# Structural optimality and neurogenetic expression mediate functional dynamics in the human brain

**DOI:** 10.1002/hbm.24942

**Published:** 2020-02-06

**Authors:** Ioannis Pappas, Michael M. Craig, David K. Menon, Emmanuel A. Stamatakis

**Affiliations:** ^1^ Division of Anaesthesia, Department of Medicine, School of Clinical Medicine University of Cambridge Cambridge UK; ^2^ Wolfson Brain Imaging Centre, Department of Clinical Neurosciences, School of Clinical Medicine University of Cambridge Cambridge UK; ^3^ Helen Wills Neuroscience Institute University of California – Berkeley Berkeley CA

**Keywords:** functional connectivity, game theory, microarray, optimality, prediction, structural connectivity

## Abstract

The human brain exhibits a rich functional repertoire in terms of complex functional connectivity patterns during rest and tasks. However, how this is developed upon a fixed structural anatomy remains poorly understood. Here we investigated the hypothesis that resting state functional connectivity and the manner in which it changes during tasks related to a set of underlying structural connections that promote optimal communication in the brain. We used a game‐theoretic model to identify such optimal connections in the structural connectome of 50 healthy individuals and subsequently used the optimal structural connections to predict resting‐state functional connectivity with high accuracy. In contrast, we found that nonoptimal connections accurately predicted functional connectivity during a working memory task. We further found that this balance between optimal and nonoptimal connections between brain regions was associated with a specific gene expression linked to neurotransmission. This multimodal evidence shows for the first time that structure–function relationships in the human brain are related to how brain networks navigate information along different white matter connections as well as the brain's underlying genetic profile.

## 1 INTRODUCTION

Optimality quantifies the extent to which a system's function is designed to maximize benefit within certain constraints (Parker & Maynard, 1990). Biological systems have adapted to attain optimality. For instance, optimal models suggest efficient energy conservation in animal behavior (Alexander, 1996), maximal information flow in transcriptional regulation (Tkacik, Callan Jr., & Bialek, 2008), balanced growth in protein expression (Dekel & Alon, 2005) and reduced wiring cost in the neuronal connectivity of the nematode *Caenorhabditis elegans* (Pérez‐Escudero & de Polavieja, 2007).

When multiple elements are interconnected as in biological networks, models can be used to assess the optimality of a network via game‐theoretic approaches such as equilibria between each element's competing strategies (Fabrikant, Luthra, Maneva, Papadimitriou, & Shenker, 2003). Unlike other biological networks, brain networks (as determined by coordinated blood‐oxygen‐level‐dependent [BOLD] activity that is, functional connectivity), emerge within the constraints imposed by their underlying structural, white‐matter network (connectome) with the purpose of serving baseline and/or cognitive function (Cole, Bassett, Power, Braver, & Petersen, 2014; Greicius, Krasnow, Reiss, & Menon, 2003; Hagmann et al., 2008; Park & Friston, 2013). Thus quantifying optimality in brain networks can lead to the understanding of the one‐to‐many relationship between structure and function at rest but also during tasks.

One approach for disentangling the relationship between structure and function is to look at how information is communicated upon fixed structural connections with models of brain communication (Laughlin & Sejnowski, 2003). Briefly, features of plausible biological models of brain communication can be summarized as follows: information communication from one region to another does not assume knowledge of the global topology of the network (as in the case of small‐world brain network hypothesis, Avena‐Koenigsberger, Misic, & Sporns, 2017; Bullmore & Sporns, 2012) but rather information is transmitted by sequentially choosing neighboring nodes along white matter paths until the target node is reached (ability to navigate information or navigability) (Seguin, van den Heuvel, & Zalesky, 2018). In addition, a minimum number of connections is used for navigation reflecting a wiring‐efficient architecture (Bullmore & Sporns, 2012).

Efficient communication of information, in terms of maximizing navigability and minimizing wiring cost, is an undeniable benefit that an optimal brain may attain (Bullmore & Sporns, 2012). Therefore, efficient communication paths in the structural connectome can serve as a low‐dimensional (compared to using the full structural connectome) structural scaffold for explaining functional connectivity during rest while demanding cognitive functions could be served by shifting from these paths to other, potentially “expensive” communication strategies (Avena‐Koenigsberger et al., 2017).

Given this assumption, we hypothesized that there is an optimal core of structural connections that promotes efficient communication and upon which functional connectivity is developed at rest. On the other hand, we hypothesized that nonoptimal connections might be recruited during task execution in the same way that BOLD activity dynamics flexibly shift from rest to task to facilitate cognition (Shine et al., 2019). Finally, we hypothesized that the difference between optimal and nonoptimal connections could be related to specific genetic signatures responsible for neurotransmission thus providing a neurobiological underpinning for efficient communication (Richiardi et al., 2015).

Methodologically for the first hypothesis, we derived an optimal network model that takes into consideration each region's navigation paths with the rest of the network while minimizing wiring cost (Gulyás, Bíró, Kőrösi, Rétvári, & Krioukov, 2016). We used this model for 50 healthy individuals and considered the overlap with their real structural networks. We termed this overlap as the level of optimality or optimality. In turn, we attempted to verify that information communicated upon optimal structural connections could explain functional connectivity. We employed an algorithm that predicted resting‐state functional connectivity by utilizing paths of a structural network (Becker et al., 2018). Using this method we showed that optimal structural connections were essential for the accurate prediction of resting‐state functional connectivity compared to using the features of the entire structural network, On the other hand, we hypothesized that task‐based connectivity (in this case during a working memory experiment) would be better predicted by nonoptimal connections. Finally, we showed that the balance of optimal and nonoptimal connections, as a potential mediator between structure, resting‐state and cognitive function, is characterized by a distinct genetic profile important for neurotransmission. To this end, we correlated the difference in the number of optimal connections and nonoptimal connections with the neurotransmitter density data obtained from post mortem brains (Hawrylycz et al., 2012).

## 2 RESULTS

### 2.1 Optimal connections in structural connectivity networks

We constructed an optimal network model where communication between brain regions was maximized with the least possible number of connections. From a graph‐theoretic perspective, describing communication involves the identification of paths form a source to a target region/node. One biologically plausible model for describing communication in the human brain is navigation via greedy routing where the closest node (in terms of spatial distance) is chosen sequentially until the target node is reached (Avena‐Koenigsberger et al., 2017). In this context, navigability was defined as the extent to which navigation can take place from one node to another. The number of connections between nodes in order to achieve navigation defined the wiring cost of navigation (Methods).

**Figure 1:**
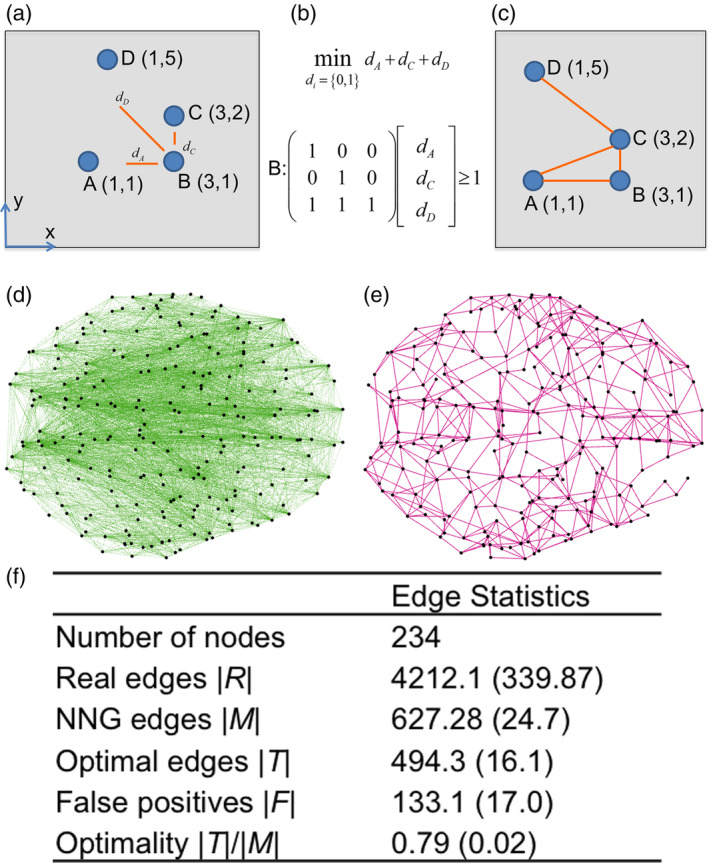
Optimality in structural brain networks (a) In this work optimality relates to finding appropriates edges that will maximize navigability at minimum wiring cost. Navigability refers to constructing paths from one node to other (target) nodes in the network by sequentially making connections to nearby (in terms of spatial distance) nodes. Each node's optimal navigation vector consists of binary decision variables indicating whether it will connect to other nodes or not in order to maximize navigability at minimum wiring cost. In the toy graph presented (we use the same layout as in Gulyás et al., 2016), node's B optimal navigation vector consists of three binary decision variables *d*_*A*_, *d*_*C*_, and *d*_*D*_ corresponding to whether node B is going to be connected to nodes A, C, and D respectively. To compute these, quantities $S_{A}^{B},$ $S_{C}^{B}$, and $S_{D}^{B}$ are calculated that represent the set of nodes that node B can navigate to using A, C, and D respectively as first steps. For example, $S_{A}^{B}$ is defined as $S_{A}^{B}=\left\{w|\text{dist}\left(w,A\right)<\text{dist}\left(w,B\right)\right\}$ (*dist* is the Euclidean distance) (b) The values of the binary decision variables are based on the minimum cover set of these three sets; here we find that *d*_*A*_ = *d*_*C*_ = 1 and *d*_*D*_ = 0. Therefore, BC and BA connections will be created. (c) By repeating calculations for the remaining nodes, it can be shown that the resulting network has maximum navigability (as a result of choosing appropriate intermediate nodes) and a minimum number of connections (as a result of the minimum cover set). This network is also the Nash equilibrium between all the nodes when it comes to maximizing navigability at minimum wiring cost (this is Nash equilibrium network game‐NNG model). Panel (d) shows an example of a whole‐brain structural network. Black dots represent the location of each ROI/node and green lines show existing connections (edges) between ROIs in the case where the general fractional anisotropy between them was nonzero. Panel (e) shows the NNG model (edges are in magenta color) obtained using the locations of ROIs shown in (d). (f) Optimality is defined as the overlap between networks D and E or in statistical terms, as the number of true positives identified by the NNG model divided by the total number NNG edges. Optimality statistics for the structural networks at the 234 resolution. Results are presented in the form of mean (*SD*) over *n* = 50 individuals

The Nash equilibrium Network Game model (NNG) takes as input each individual's topographical organization, as defined by the spatial location of brain regions/nodes, and creates a synthetic network by maximizing navigability between nodes and minimizing wiring cost (Gulyás et al., 2016). Given a node *i*, the navigation vector consists of binary variables *d*_*j* ≠ *i*_, each one representing whether node *i* is going to connect to other nodes *j* ≠ *i* or not. The value of each binary variable *d*_*j* ≠ *i*_ is derived as follows. Based on the navigability premise, node *i* will potentially connect to node *j* if information can be navigated from node *i* to the rest of the network using node *j* as a greedy next hop, that is, by transmitting information first to *j* and then from *j* to the rest of the network. This requires identifying the paths from node *i* to the rest of the network using node *j* as a greedy next‐hop and, eventually, finding the (target) nodes that can be reached from *i* using these paths. The collection of these target nodes consists of a mathematical set, denoted by $S_{j}^{i}$. After quantifying $S_{j}^{i}$ for every node *j* ≠ *i*, the collection/union of these sets $S_{j}^{i}$ represents all the nodes that *i* can efficiently navigate to using the *j* nodes as potential next hops (collection of target nodes). To which *j* nodes node *i* is going to connect to depends on the collection of the target nodes. Node *i* should be connected to as few *j* nodes as possible (based on the minimum wiring premise) but, at the same time, information should be navigated to all the target nodes (based on the navigability premise). Thus, the optimal navigation vector of node *i* is to select the minimum number of sets $S_{j}^{i}\;$ whose union still includes the collection of target nodes. This is the minimum cover set of sets $S_{j}^{i}$. If $S_{j}^{i}$ is selected in the minimum cover set of all $S_{j\neq i}^{i}$ sets then *d*_*ij*_=1 and a connection is made between *i* and *j* (Figure 1a–c, and Methods).

By repeating this process independently for all nodes, the resulting network is the Nash equilibrium of all the optimal navigation vectors. One way of thinking about the Nash equilibrium is to consider two friends (players) that try to maximize happiness by considering different eating out options. If Player 1 decides to eat lunch, then Player 1 is happy because she likes lunchtime and Player 2 is unhappy because it is too late for her. If Player 2 decides to eat breakfast she will be happy, Player 1 though will be unhappy because it is too early for her. However, if they both go for brunch, they will attain a certain level of happiness and they cannot get any happier by choosing a different option (lunch or breakfast); this renders brunch as the Nash equilibrium between the two players. The Nash equilibrium in our setup is the network configuration where each node's optimal navigation vector has been attained thus rendering any other configuration to be suboptimal for any node. From a computational perspective, the Nash equilibrium network (NNG network) comes from the minimum cover set selection process for every node as described previously and is a synthetic network that has maximal navigability between every node with a minimum number of connections (because of the definition of $S_{j}^{i}\;$ and the minimum cover set).

Given this model, our first objective was to assess the overlap between NNG and real structural networks in 50 healthy individuals. Here structural networks were defined by generalized fractional anisotropy (GFA) values for tracts connecting regions of interest (ROIs) based on a specific parcellation spanning the entire brain (see Methods). To quantify this overlap, we defined an optimal connection (*T*) between two ROIs as one that existed in both the structural network (i.e., nonzero GFA) and the NNG network (true positives). Nonoptimal connections were those that existed in the structural network but were not present in the NNG network (Figure 1d,e). False positives were defined as those connections that existed in the NNG network but not in the structural network (i.e., zero GFA) (*F*). Using notation, optimality was defined as the ratio |*T|/|M|* where |*T|* was the total number of optimal connections and |*M|* was the total number of connections in the NNG network. False positives were defined as *|F| = |M|−|T|*. We first report results for a 234‐ROI parcellation. The mean optimality score across subjects (mean = 0.79, *SD* = 0.02) suggested that brain networks follow a consistent pattern of optimal connectivity (Figure 1f). We also report the number of false positives across subjects (mean = 133.1, *SD* = 17.0) compared to the number of connections that the NNG produced (mean = 627.28, *SD* = 24.7).

To show that our results were consistent across different parcellation resolutions we applied the same model to the 129‐ROI parcellation. Optimality of structural networks using this parcellation was also high (mean = 0.7161, *SD* = 0.0430) while false positives remained low (mean = 93.8, *SD* = 15.4) compared to the number of connections that the NNG produced (mean = 334.1, *SD* = 5.4) (Appendix S1). We further wanted to assess the extent to which the results would change when using geodesic distances instead of Euclidean distances entertaining the assumption that paths along the cortical surface mesh would be preferable. Because geodesic distances are defined over closed surfaces within each hemisphere, optimality for each hemisphere was calculated separately (Methods). We observed that optimality dropped to 0.6182, *SD* = 0.0286 for the left hemisphere and 0.6454, *SD* = 0.0230 for the right hemisphere. The reduction in optimality could be attributed to the fact that communication utilizes white matter pathways that do not relate to the geodesic distances that characterize the cortical mesh.

### 2.2 Optimal connections in large‐scale networks

**Figure 2:**
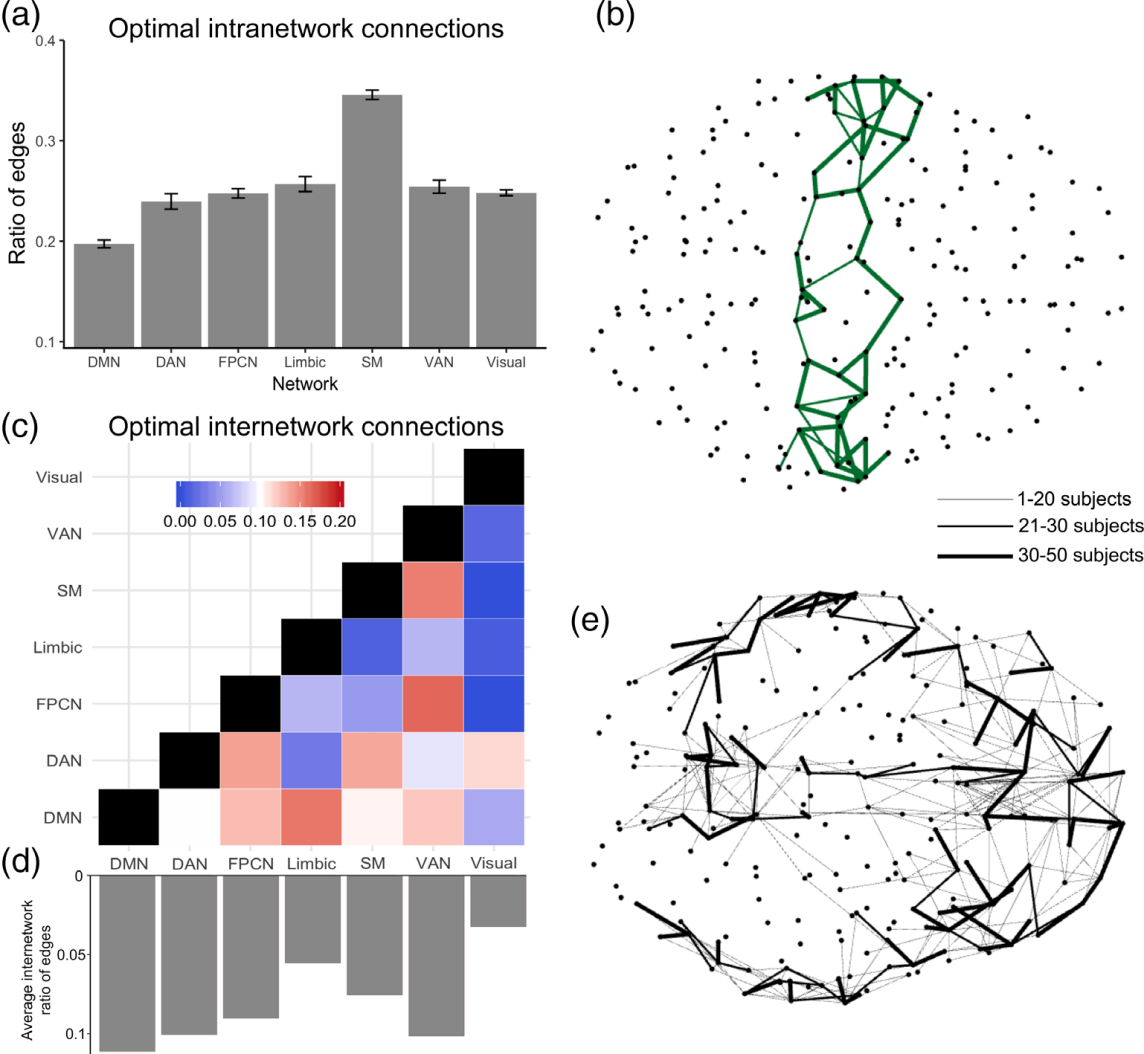
Intra‐ and internetwork optimal connections in structural brain networks (a) Optimal intranetwork connections in each of seven canonical cortical brain networks (CCNs). Bars show mean and *SE* of the mean from *n* = 50 individuals. (b) Intranetwork optimal connections in the somatomotor network, the network that showed the greatest number of intranetwork connections. (c) Optimal internetwork connections between each cortical network averaged across individuals. (d) Bars below show the mean of optimal connections from each network to the other six networks. (e) Internetwork optimal connections from the DMN, the network that showed the greatest number of internetwork connections. For (b) and (e) line emphasis corresponds to connections that exist in more than 30 (of the 50 subject sample) subjects. CCN definitions are from Yeo et al. (Yeo et al., 2011). All numbers reflect ratios of the number of optimal connections over the total number of intra‐ or internetwork connections respectively. CCN abbreviations: DMN, default mode network; DAN, dorsal attention network; FPCN, frontoparietal control network; SM, somatomotor network; VAN, ventral attention network; Visual, visual network

We next investigated whether optimal connections were distributed differentially across a set of seven canonical cortical networks (CCNs), namely the default mode network (DMN), the dorsal attention network, the frontoparietal network, the limbic network, the somatomotor network, the ventral attention network, and the visual network (Yeo et al., 2011). Hierarchical organization across these networks has been widely discussed (Margulies et al., 2016) so this was an attempt to understand whether such organization is underpinned by optimal connections. To this purpose, we first assigned each ROI from the Lausanne 234‐ROI parcellation to each CCN. We then computed the number of optimal connections between ROIs within CCNs (intranetwork optimal connections) and between ROIs of different CCNs (internetwork optimal connections). To account for differential CCN sizes we divided the number of optimal connections by the total number of intra‐ or internetwork connections (Methods). We found that the somatomotor network (SM) contained the highest proportion of optimal intranetwork connections (Figure 2a,b). This is in agreement with earlier studies proposing that the SM network has increased within‐network connectivity in relation to other networks (Power et al., 2011). The DMN showed the lowest relative optimal intranetwork connectivity, possibly reflecting the metabolically expensive frontoparietal connections linking the medial prefrontal cortex and posterior cingulate/precuneus areas (Greicius, Supekar, Menon, & Dougherty, 2009). In contrast, we found that the DMN had substantially higher internetwork optimal connectivity than any other network (Figure 2c–e), in support of the idea that the DMN might act as a global workspace, integrating information from other distributed networks (Vatansever, Menon, Manktelow, Sahakian, & Stamatakis, 2015). Furthermore, there are suggestions that the DMN occupies the top of the brain's large‐scale connectivity hierarchical organization (Margulies et al., 2016).

### 2.3 Predicting resting‐state functional connectivity using optimal structural connections

**Figure 3:**
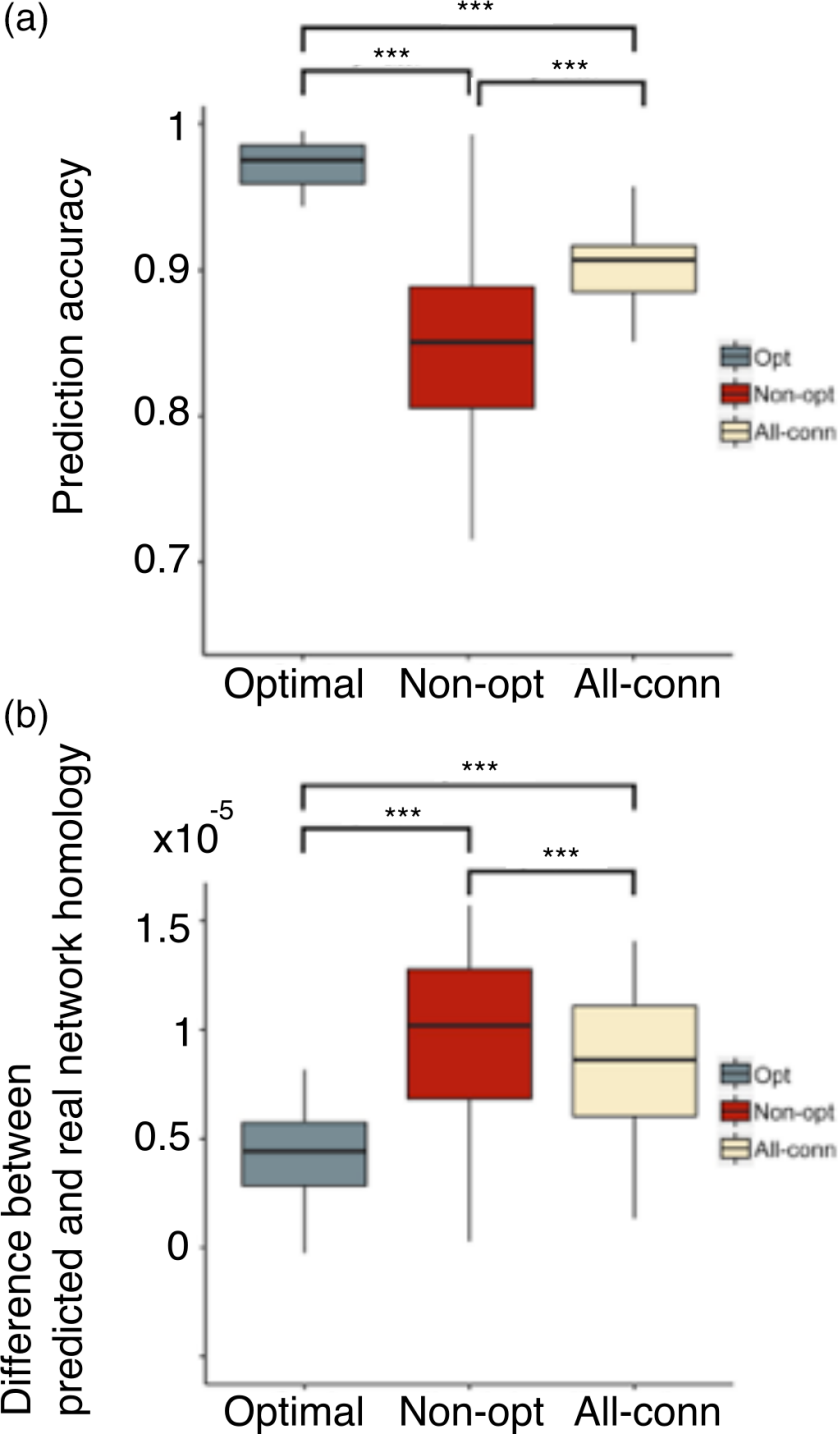
Predicting functional connectivity from structural connectivity (a) The prediction algorithm uses the eigen‐structure of a polynomial transformation of order *k* (here only for *k* = 5) of the structural connectivity matrix to predict the functional connectivity matrix (Figure 3‐Appendix S1). Each box plot shows the prediction accuracy across n = 50 individuals in terms of cross‐matrix correlation between the real and predicted matrices. Higher scores show higher predictability. See Figure 3‐Appendix S2 for different polynomial orders *k*. (b) Here we used methods from homology theory to correlate predicted and real functional matrices. Lower scores reflect a smaller difference between the real and predicted functional networks. Both prediction scores showed that using only optimal (*Opt*) connections was more predictive of whole‐brain functional connectivity than using nonoptimal (*Non‐opt*) or all structural connections (*All‐conn*). For each box, thick lines show the median value for *n* = 50 individuals while whiskers reflect the maximum and minimum values of the data. ***indicates *p* < .001 significance See Figure 3‐Appendix S3 for comparable results at the 129‐resolution parcellation

Next, we probed the capacity of the optimal connections to predict whole‐brain functional connectivity (Figure 3‐Appendix S1). We employed a recently developed algorithm that predicts resting‐state functional connectivity using the eigen‐structure of a structural connectivity matrix (Becker et al., 2018). Specifically, the predicted functional connectivity matrix was obtained by a polynomial transformation of the structural connectivity matrix utilizing the latter's path information thus allowing us to assess how navigability on paths consisting of optimal (and nonoptimal connections) related to the emerging functional connectivity (Figure 3‐Appendix S1 and Methods). The higher the order of the polynomial transformation, the longer the paths that are considered for prediction. Based on previous work (Becker et al., 2018), we chose k = 5 as our order of interest. Specifically, for this k the algorithm produces maximal correspondence between predicted and real functional connectivity matrices while the prediction accuracy plateaus when considering higher‐order (k > 5) polynomial transformations. We computed predicted functional connectivity matrices for each individual using optimal, nonoptimal and all connections in the structural matrices and we compared the predicted matrices with actual functional connectivity matrices obtained from each individual's BOLD data. For the 234‐ROI parcellation we found that predictability was superior when using only the optimal connections (correlation coefficient between predicted and real functional matrices mean r = .9688, *SD* = 0.0285) compared to using all the structural connections (mean r = .9156, *SD* = 0.0256) (Figure 3(a)). We also found that predictability dropped considerably when using only the nonoptimal structural connections (correlation coefficient between predicted and real functional network mean r = .8414, *SD* = 0.0572). A one‐way repeated‐measures analysis of variance (ANOVA) confirmed that each prediction was significantly different from the others (F[2,98] = 125.74, *p* < .0001, post hoc tests were Bonferroni corrected for multiple comparisons). For prediction results across different polynomial transformation orders, see Figure 3‐Appendix S2.

These results were further verified using a newly introduced method for predicting functional diversity from structure based on persistent homology tools (Liang & Wang, 2017). Instead of just quantifying the correlation between the fully connected predicted and real functional connectivity matrices, this method looks at the persistence of their topological differences across all possible thresholds‐from fully connected matrices to sparsely connected matrices (Methods). In this case, a higher score would imply poorer prediction. Here we observed that the differences in connected components were smaller using only optimal connections thus showing their high predictive accuracy (F[2,98] = 28.07, *p* < .0001, post hoc tests were Bonferroni corrected for multiple comparisons) (Figure 3b). To further confirm that the prediction results were a function of an optimal core of structural connections and not an artifact of the parcellation resolution, we repeated these analyses using the 129‐ROI parcellation and obtained similar results (Figure 3 **‐**Appendix S3).

Further, we conducted a similar analysis using streamlines instead of GFA, in other words, GFA values in the structural connectivity matrices were replaced by the number of streamlines. We found that the prediction results remained unchanged (Figure 3‐Appendix S4).

### 2.4 Controlling for distance

It is possible that the prediction of resting‐state functional connectivity by the NNG model could be confounded by the model's preference for short‐range connections (Hermundstad et al., 2013). We performed control analyses to see whether there was an effect of distance in the predictability (here only in terms of correlation coefficient between predicted and real functional network) of the optimal connections. We compared the predictability of resting‐state functional connectivity of the NNG model to that of an appropriate generative network model that preferentially creates edges based on short Euclidean distances and closely matches the individual structural networks (Betzel et al., 2016). We found that the predictability of the synthetic model using Euclidian distances was lower (mean r = .9494, *SD* = 0.0182, two‐sample *t* test *p* = .0042**)** compared to the NNG.

A remaining question is whether the difference in the high predictabilities between optimal connections and synthetic networks is meaningful or it is an artifact of the prediction algorithm that potentially overfits and predicts functional connectivity using only short distances. We, therefore, repeated the comparison where we tried to predict functional connectivity with distance regressed out from functional connectivity (Methods) and we found that the predictability of optimal connections was substantially higher (two‐sample *t* test *p* < .001) using optimal connections (mean = 0.6908, *SD* = 0.0764) compared to that of the synthetic networks (mean = 0.6189, *SD* = 0.0957). Thus, we concluded that distance itself could not explain the predictive power of optimal connections. It is worth noting that the predictability of the synthetic networks was higher than that of all brain connections, potentially supporting the notion that structural connectivity resembles functional connectivity at short distances (Honey et al., 2009). However, synthetic networks still underperformed compared to the NNG.

### 2.5 Predicting task‐based functional connectivity using optimal structural connections

Our findings so far indicate that optimal connections successfully predict resting‐state functional connectivity. Nonoptimal connections corresponded to connections that did not conform to the maximal navigability with minimum wiring principle. In that regard, an important question remains as to what is the functional role of nonoptimal connections. Nonoptimal connections by definition do not necessarily conform to the navigability principle. Thus, they could consist of direct long‐range connections between source and target. One possible explanation could be that these correspond to connections that support direct communication between regions when this is needed. Such configurations are believed to support integration at the whole‐brain level by connecting specialized regions (Betzel & Bassett, 2018). Parallel to this, many studies focusing on working memory have shown that functional connectivity during a working memory task becomes more integrated compared to rest (Cohen & D'Esposito, 2016; Vatansever et al., 2015). Together these results entertain the following hypothesis: nonoptimal connections would predict task‐based connectivity better compared to resting‐state data due to their role in integrating information when cognitive demands increase. We tested this hypothesis by looking at how optimal and nonoptimal connections would predict functional connectivity during a working memory paradigm (two‐back task/2bk) and compared this prediction to the previously established prediction for resting‐state functional connectivity. We observed a significant increase (difference between means = 0.0214, *p* < .001) in the predictability of nonoptimal connections during the 2bk task compared to resting‐state (Figure 3‐Appendix S5). In addition, we observed that the predictability of the optimal connections during the 2bk task was significantly reduced (difference between means = 0.1095, *p* < .001) when compared to rest. So overall much greater difference in terms of optimal and nonoptimal predictability when comparing task to rest. The difference was only apparent when separating optimal and nonoptimal connections, and not when considering all connections (Figure 3‐Appendix S5). Collectively these results propose that the balance between optimal and nonoptimal connections relates to whether the brain engages with increased cognitive demands.

### 2.6 Neurogenetic basis of optimally and nonoptimally driven functional connectivity

**Figure 4:**
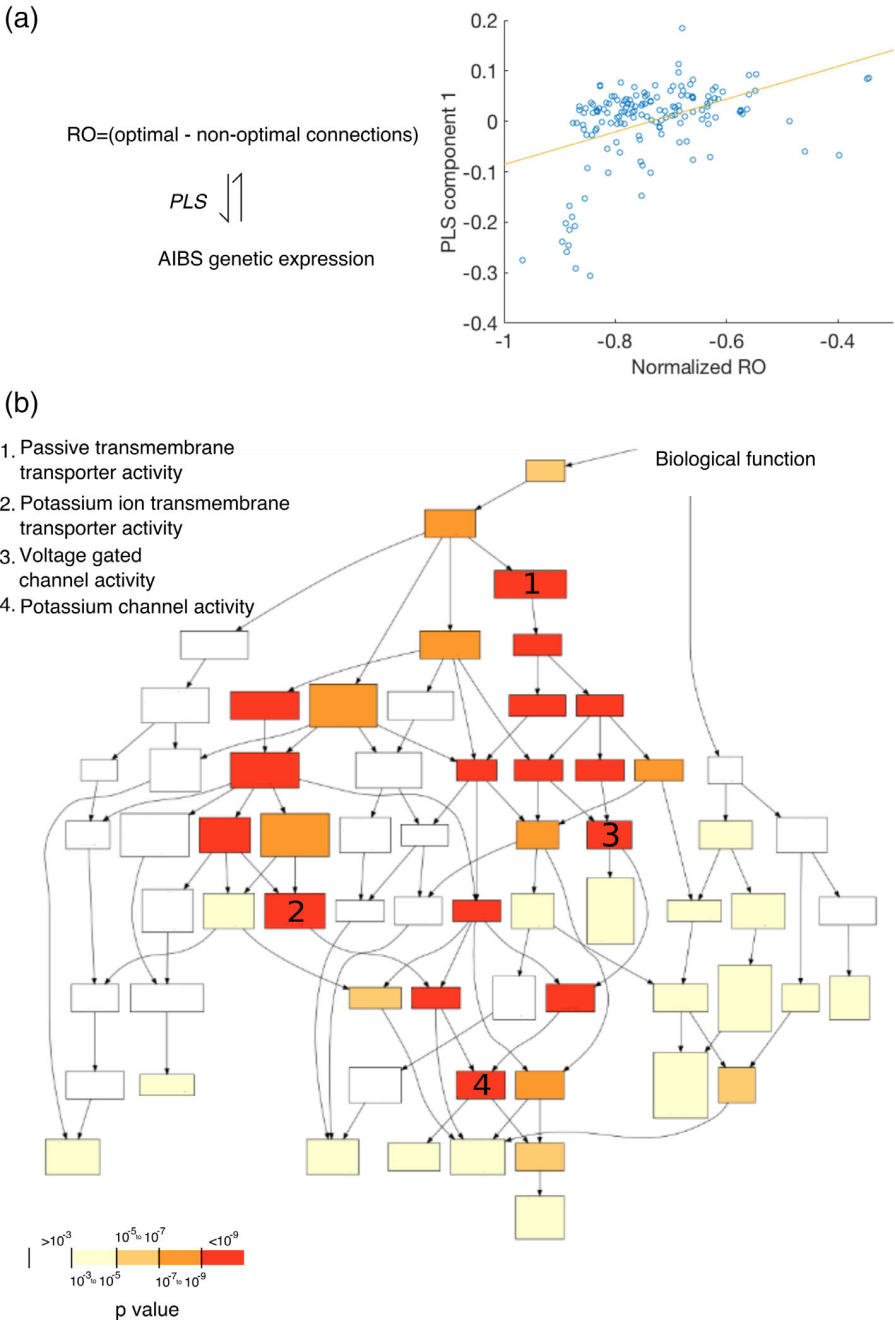
Association between gene expression and regions with higher optimal connectivity (a) We related AIBS gene expression data to the extent to which each region contained optimal connections that is, the difference between optimal and nonoptimal connections (RO). Partial least squares (PLS) analysis revealed a significant component driving the variance in the RO score (optimal–nonoptimal connections) of each ROI. (b) Using a gene ontology toolbox, we found that this PLS component was enriched for genes functional related to transmembrane transportation and channel activity. For a complete list of significant genes and their function see Appendix S2. Because differences could be due to the different number of connections in each region, RO scores were normalized by the total number of connections of each region (normalized RO)

We next sought to identify the neurobiological mechanism that allows optimal and nonoptimal connections to drive functional connectivity in a differential fashion. First, we calculated the difference between the number of optimal and nonoptimal structural connections (averaged over 50 subjects) for each region (degree of optimal connections minus degree of nonoptimal connections normalized by the total degree of each region), calling this metric each region's regional optimality (RO). Then we identified which genes were disproportionately expressed in ROIs with high RO. To do so we mapped the location of the samples from the AIBS human microarray data set to the ROIs of the Lausanne 234‐ROI parcellation to obtain their respective microarray expression (Whitaker et al., 2016). For the ROIs that were matched to sample locations, we used a regression analysis to find a relationship between RO scores and the gene expression scores (Methods). This analysis resulted in several PLS components consisting of weighted linear combinations of gene expression scores that explained most of the RO score variance and were ranked by the cumulative variance explained in both the gene expression data and (normalized) RO scores. We observed that the top PLS component, explained around 18% of the variance in RO scores and we found a positive association between the (normalized) RO score with the PLS component (Spearman's ρ = 0.2975, *p* < .0001, Figure 4a) implying a relationship between the genetic expression of each ROI and its RO score. Focusing on the gene expression profile of this PLS component, we found that its transcriptional signature was significantly enriched in genes associated with voltage gated ion channels and small amino acid neurotransmitters (Figure 4b, for the complete list see Appendix S2a; for a list of gene function categories and statistics, see Appendix S2b). These genes code for membrane bound proteins that play a role in the regulation of synaptic transmission and the initiation of action potentials. To verify that these results do not reflect some form of spatial variation in the genetic expression across the cortex, we shuffled the assignment of ROIs between the gene and the RO data and re‐computed the PLS components for 1,000 permutations. We found a rejection of the null hypothesis at *p* < .02 (Figure 4‐Appendix S1) indicating that our results were not driven by the spatial autocorrelations in the genetic and RO data.

## 3 DISCUSSION

In this work, we used the NNG model as an ideal network where communication between nodes is optimal with a minimum wiring cost and assessed its overlap with real structural networks. With the same methodology, Gulyás and colleagues showed that many real‐world networks, including the Internet, metabolic networks, roads, airports, and one human brain network are highly optimal (Gulyás et al., 2016). Using diffusion imaging data from 50 healthy volunteers, we expanded this finding to show that structural networks have consistently high optimality. Earlier work has shown that structural networks have small‐world architecture that is, their organization is characterized by high efficiency in communication usually facilitated by the presence of short paths (Bullmore & Sporns, 2012). How do these findings relate to the small‐world hypothesis? The high overlap between real and optimal networks potentially suggests that structural networks have developed to efficiently communicate information in a pattern that takes into consideration how each node differentially routes information to the rest of the network, expanding the small‐world framework where one would expect information to be routed using only shortest paths (Avena‐Koenigsberger et al., 2017). In turn, the NNG model results from the Nash equilibrium between each node's optimal navigation vectors. The only input required for deriving the NNG model is each node's position and how it is relative to its neighbors' positions. This process is different from network generative small‐world models that impose constraints (or favor) the emergence of a specific whole‐brain network structure (Vértes et al., 2012).

The NNG model uses the brain's three‐dimensional layout to produce efficient navigable networks. The notion that brain network efficiency is intertwined with the geometrical placement of brain regions has been the focus of research looking at brain organization (Cherniak, 1994; Ercsey‐Ravasz et al., 2013). One account suggests that the positioning of regions relates to the emergence of optimal brain networks in terms of efficient wiring at minimum cost (Cherniak, Mokhtarzada, Rodriguez‐Esteban, & Changizi, 2004; Chklovskii, Schikorski, & Stevens, 2002; Klyachko & Stephens, 2003; Koulakov & Chklovskii, 2001). This positioning is considered to be the result of developmental mechanisms and the increasing tensions associated with expanding brain volume and size (Van Essen, 1997). The tension hypothesis emphasizes the mechanical forces between neurons and their role in shaping brain morphology. Because the size of the cortical sheet is larger than the space needed to develop, tensions along the axons of connected neurons influence spatial morphology within this reduced space. For example, tensions would pull strongly interconnected regions towards one another, forming an outward fold (e.g., formation of a gyrus) thus reducing the distance between regions in the opposite sides of the fold. This process can explain why region positioning is related to efficient wiring at a minimum cost (Wang & Clandinin, 2016). Along these lines, Nash optimality could be regarded as a “goodness‐of‐fit” measure for individual brains that captures the extent to which efficient connectivity is developed within a layout of regions.

The discrepancy in optimality when using geodesic distances suggests that navigability is not as efficient when following paths along the surface. One explanation for this is that large‐scale signaling uses paths formed along white matter connections whose length has been shown to strongly correlate with the Euclidean distance between these regions (Roberts et al., 2016). It is worth noting that navigability might depend on a combination of white matter and cortico‐cortical surface paths. To this end, models have assessed the impact of both geodesic and Euclidean distances to communication strategies (Avena‐Koenigsberger et al., 2019). In our work, one can modify the process of finding navigation paths to depend on both geodesic and Euclidean distances, thus accounting for both of these factors when assessing optimality in the brain. We plan to explore these ideas in a future study.

We then asked how optimal structural connections were differentially distributed across CCNs. First, a high number of optimal connections were found within the SM network. This is consistent with previous studies suggesting the SM network has high local communication efficiency possibly reflective of continuous and comparatively rigid processing demands (Power et al., 2011). In contrast, the DMN had a low number of intra‐network optimal connections potentially attributed to the fact that the NNG was not able to capture direct, metabolically expensive connections linking the medial prefrontal cortex with the posterior cingulate cortex (Ongur & Price, 2010).

Despite having a low number of optimal intra‐network connections, the DMN had the highest number of optimal internetwork connections. Along these lines, studies on structural connectivity have singled out the DMN as a network extensively connected to regions of other large‐scale networks (Parvizi, Van Hoesen, Buckwalter, & Damasio, 2006). Our results show that this connectivity was supported by principles of efficient communication and minimum wiring cost reflecting flexible information exchange of the DMN with other large‐scale networks during different cognitive states (Vatansever, Menon, & Stamatakis, 2017). In a recent study by Margulies and colleagues (Margulies et al., 2016) the authors showed that DMN regions occupy ideal positions along a principal gradient, a property related to the topographic organization of large‐scale connectivity, thus promoting efficient information processing. The NNG model provides a complementary approach to understanding the DMN's unique multifunctional, integrative fingerprint in the sense that it substantiates the DMN's high number of optimal connections towards the rest of the brain. When conceptualizing the proportion of internetwork optimal connectivity in a continuous spectrum, our results place the DMN distant from the contributions of other networks, thus providing evidence for its functional heterogeneity and flexibility.

We next used structural‐to‐functional matrix association in order to identify the extent to which optimal connections can predict functional connectivity. The prediction algorithm utilized path information embedded into the structural connectivity matrix by expressing functional connectivity as a weighted combination of the powers of the matrix, each one associated with paths of different lengths (Becker et al., 2018). Remarkably, we found that optimal connections were predictive of whole‐brain resting‐state functional connectivity. Our finding proposes that paths consisting of optimal edges serve as better predictive features for explaining functional connectivity. Interregional communication using optimal paths might provide a framework for explaining flexible brain function with information efficiently navigating between functionally specialized regions (Avena‐Koenigsberger et al., 2017). Compared to using the whole structural connectome, these paths could serve as a low‐dimensional scaffold for brain communication during resting state. Indeed, a previous positron emission topography study showed uneven distribution in energy consumption with glucose metabolism distinctively higher in brain regions important for brain communication (Tomasi, Wang, & Volkow, 2013). This suggests that cortical networks attain efficient energy consumption by maintaining high metabolism in selective regions important for brain communication and, potentially shifting the metabolic demands to other regions in the case of serving cognitive function. This agrees with our finding showing that a handful of paths important for communication predict resting‐state functional connectivity while, as we discuss later, tasks require the recruitment of additional paths that potentially come with a certain metabolic and energetic cost.

It is worth noting that our comparison with a synthetic, distance‐penalizing model showed that predicting functional connectivity could not be explained solely based on interregional distance. Optimal communication considers the brain's ability to transmit information through efficient navigation paths but not necessarily through shortest paths that solely take into account the spatial proximity of brain regions. Thus, communication of information based on distance cannot fully account for predicting functional connectivity.

Nonoptimal edges were predictive of functional connectivity during a working memory task. It is possible that nonoptimal connections reflect direct and metabolically expensive connections linking and integrating information from distant parts of the brain such as the posterior and anterior parts of the DMN (Ongur & Price, 2010). Previous work has shown that functional connectivity during a working memory task becomes more integrated with the emergence of long‐range functional connections especially within the DMN (Vatansever et al., 2015). We believe that our results contribute to this notion as nonoptimal connections predicted 2bk functional connectivity better that resting‐state functional connectivity. This result, in conjunction with other studies suggesting that long‐range connections have high connectional specificity (Betzel et al., 2017), indicates that nonoptimal connections might serve a specific functional role during tasks: the integration of information between distant parts of the brain. It is crucial to investigate whether these results generalize in other tasks or cognitive domains in order to establish whether nonoptimal connections are a feature of task connectivity in general or if they are specific to working memory.

Our work on linking structure to function disentangles the contributions of optimal and nonoptimal connections to the prediction of rest and task‐based functional connectivity. We claim that optimal connections are important because they allow efficient communication at minimum cost and that this is a suitable scaffold for resting‐state functional connectivity. However, to serve cognitive demands the brain additionally recruits nonoptimal connections potentially for long‐range communication. This approach diverges from previous studies investigating structure‐to‐function relationships using correlations between all structural and functional connections (Hermundstad et al., 2013; Honey et al., 2009). In these studies, it was only speculated that the observed differences between how structure relates to resting‐state functional connectivity and how structure relates to task‐based functional connectivity would be mediated by certain structural connections (Hermundstad et al., 2013). Here we separated optimal and nonoptimal connections and thus provided direct evidence for why these differences might exist.

It is also worth noting that predicting functional connectivity using optimal connections could potentially provide useful insights in clinical settings. Alterations in the predictive capacity might suggest diversions from a normal “optimally” configured brain that can be quantified and used to classify between certain clinical populations and healthy controls.

Finally, the relationship between structural optimal, nonoptimal connections and function was complemented by the genetic analysis, where we showed that regions with an increased number of optimal connections minus nonoptimal connections (RO score) have higher expression of genes tied to regulating neurotransmission and synaptic plasticity. We found that the top PLS component correlated significantly with regions with high RO. Genes overexpressed in this component were associated with potassium and calcium gated channels. These are associated with action potential initiation and propagation that regulates neurotransmission (Lledo et al., 1995; Shull & Lingrel, 1987). These results are also in the direction of findings associating highly connected regions (hubs) with genes producing adenosine triphosphate (ATP) in the mouse brain (Fulcher & Fornito, 2015). Thus, our results provide initial evidence that optimal connections could promote efficient communication potentially by utilizing mechanisms responsible for neural communication. We could not draw similar conclusions when the gene samples' location assignments were randomly permuted; thus we concluded that the variation of genes along regions with high RO must reflect an intrinsic architecture and not just a spatial variation of the genes along the brain as it has been previously shown (Burt et al., 2018). However, it is worth noting that due to the limited information of the genetic data (approximately 20,000 genes from two postmortem brains) these results are exploratory and require further verification.

The following limitations should be considered when interpreting our findings. Despite strict preprocessing pipelines in the diffusion data, signal/GFA dropout could have biased the finding of optimal and nonoptimal connections. In future work, we plan to address this point by looking at additional diffusion data with different low signal to noise ratio and quantifying the relationship between signal dropout and optimal/nonoptimal connection identification. The model that relates structural and functional connectivity has too many degrees of freedom, which might result in overfitting. This in combination with the inherent fiber tracking difficulties due to signal dropout necessitates caution when interpreting the associations between structural and functional connectivity matrices. In addition, future work will involve further evaluation of the robustness of our results against alternative distance models that perhaps incorporate more complicated distance‐based rules for the creation of connections between nodes. Furthermore, the assignment of nodes to CCNs should be addressed with caution as imperfect overlap between regions of interest and CCNs could bias the results. Further work will re‐confirm the resting‐state prediction results with global signal regression and the task‐prediction results using the whole task period rather than blocks of task on/off.

Notwithstanding these limitations, by combining mathematical modeling techniques with multimodal imaging and genetic datasets, we identified a set of optimal structural connections, differentially distributed across the brain that were highly predictive of whole‐brain functional connectivity. Furthermore, we showed that regions with a high number of optimal connections are enriched with genes that code for the fundamental architecture of neuronal communication. Further discovery of these subtle but vital relationships will deepen our understanding of how function emerges from optimal structure and may help identify mechanisms associated with brain disease in cases of suboptimal solutions.

## 4 MATERIALS AND METHODS

### 4.1 Data

Data were downloaded from the Human Connectome Project (HCP) website: http://www.humanconnectome.org/ (Van Essen et al., 2013) Structural and functional magnetic resonance images from a total of 50 subjects between the ages of 22 and 35 were used in the analysis. Demographics are in the Appendix.

### 4.2 Individual parcellations

High‐resolution T1‐weighted scans were first segmented using *FreeSurfer*'s *recon‐all* function (Fischl, Sereno, Tootell, & Dale, 1999). These were parcellated using the Lausanne 2008 atlas (Hagmann et al., 2008) into two different resolutions of 234 or 129 regions of interest (ROIs) using the *easy_lausanne* software with settings corresponding to each resolution (http://github.com/mattcieslak/easy_lausanne). The parcellated structural images were then aligned to each individual's diffusion and fMRI images (Cammoun et al., 2012; Daducci et al., 2012). The obtained *x*, *y*, *z* coordinates for the center of mass of each ROI in each individual were utilized in the Nash Equilibrium Network game model construction described in the “Nash equilibrium Network game model (NNG)” section.

### 4.3 Structural and functional connectivity

Real structural connections were defined using High Angular Resolution Diffusion Imaging (HARDI). Preprocessing was conducted as part of the HCP pipeline and included eddy current and motion corrections, gradient non‐linearity correction, and transformation to native structural space (Sotiropoulos et al., 2013). The diffusion tensors were reconstructed in DSI‐studio (http://dsi-studio.labsolver.org) using generalized q‐sampling imaging (Yeh, Verstynen, Wang, Fernández‐Miranda, & Tseng, 2013). Using each individual's Lausanne parcellation ROIs and GFA values obtained from a deterministic fiber‐tracking algorithm, we created a 234 × 234 or 129 × 129 connectivity matrix depending on the resolution of the parcellation used. Each entry (*i*, *j*) contained either a positive number between 0 and 1 corresponding to the GFA when a nonzero GFA between each pair of brain regions was obtained or a 0 when a zero GFA value was obtained. The number of streamlines between each pair of regions was also obtained. For resting‐state functional connectivity, whole‐brain echo‐planar imaging was acquired with a 32‐channel head coil using a 3 T Siemens Skyra scanner, modified for use in the HCP. The HCP minimal preprocessing pipeline was used to preprocess functional data. This included artifact removal, motion correction, registration to the structural T1‐weighted scans, and nonlinear registration into MNI152 space (Glasser et al., 2013). Connectivity analysis was performed using the Conn functional connectivity toolbox (Whitfield‐Gabrieli & Nieto‐Castanon, 2012). Functional images were highpass filtered at 0.009 Hz to remove low‐frequency drifts due to scanner noise. Physiological noise was removed by using the anatomical *CompCor* (aCompCor) technique (Behzadi, Restom, Liau, & Liu, 2007). Motion related noise and linear drifts were also removed. Following preprocessing we computed temporal correlations between each region's BOLD signal in the Lausanne parcellation, resulting in 234 x 234 or 129 x 129 functional connectivity matrices for each individual. A 2back working memory task was acquired with similar parameters. Task‐specific time series and functional connectivity matrices were obtained using the Conn toolbox after concatenating the functional data corresponding to each task condition. Details are in the Appendix.

### 4.4 Nash equilibrium network game model

The NNG model is derived from the Nash equilibrium between all the network nodes' optimal navigation vectors coming from maximizing navigability while maintaining a minimum number of edges as in Gulyás et al. (Gulyás et al., 2016). For a node *u*, navigability refers to the ability to make a path from u to any target simply by progressing to the next node closest to the desired target that is, the ability of making a greedy path between *u* and the target. Formally in an *N*‐node graph, the navigation vector of a node *u* ∈ *V* = {1, …*N*} consists of creating connections to the rest of the nodes; this is formulated as a navigation vector *s*_*u*_ = (*s*_0_, *s*_1_, …, *s*_*N* − 1_) while G(s) is the resulting graph. The objective function can be defined as *c*_*u*_ = ∑_{∀*v* ≠ *u*}_*p*_*G*(*s*)_(*u*, *v*) +  ∣ *s*_*u*_∣ where *p*_*G*(*s*)_(*u*, *v*) is the navigability term. Specifically *p*_*G*(*s*)_(*u*, *v*) is 0 if G(s) contains a greedy (choosing neighboring nodes sequentially as intermediate steps) path from *u* to *v* and infinity otherwise. The term ∣*s*_*u*_∣ represents the number of connections. Each node's *u* optimal navigation vector is the one that minimizes the function *c*_*u*_ that is, maximizing navigability and minimizing wiring cost.

We provide more information as to how the optimal navigation vector of each node was calculated. For nodes *u* and *v* ∈ *V* if *dist*(*p*_1_, *p*_2_) is defined as the Euclidean distance using the *x*, *y*, *z* coordinates of each node/ROI (as described in the “Individual parcellations” section), then ${\;S}_{v}^{u}=\left\{w\;\right|\text{dist}\left(v,w\right)<\text{dist}\left(u,w\right)\}$ represents the set of nodes *w* to which one can navigate to from starting from *u* and using node *v* as a greedy next hop. Given this, the problem of finding the optimal navigation vector for a node *u* that would minimize *c*_*u*_ is summarized as follows. First, node *u* is associated with a collection of sets $S_{v}^{u}$, each one corresponding to the rest of the nodes *v* ∈ *V*\{*u*} that *u* is potentially going to connect to. These represent all the (target) nodes that information from *u* can reach using the *v* nodes as next greedy hops. In turn, the optimal navigation vector of node *u* consists of constructing edges to those nodes *v*′ such that their $S_{v\prime }^{u}$ sets belong to the minimum cover set of the sets $S_{v}^{u}$. For a collection of sets, the minimum cover set problem refers to selecting the minimum number of sets such that their union includes all the elements appearing in the collection of sets. Thus the minimum cover set will select the minimum number of nodes *v* that *u* can be connected to while information can still be navigated to all target nodes. This corresponds to the optimal navigation vector of node *u*. If this process is repeated independently for all nodes *u* ∈ *V*, it can be proved that the resulting network is the Nash equilibrium of all the nodes' strategies and is characterized by maximum navigability with minimum number of edges (Gulyás et al., 2016). From a computational perspective, for each ROI *u* we extracted its optimal navigation vector by coding the minimum cover set problem defined previously and solving it using the *glpk* library (https://www.gnu.org/software/glpk). This was repeated for all ROIs of each individual resulting in a subject‐specific NNG model. Instead of the Euclidean distance *dist*(*p*_1_, *p*_2_), the geodesic distance (the shortest path along the cortical surface) was also used in order to find subject‐specific NNG models. More information is provided in the Appendix.

### 4.5 Structure‐to‐function prediction algorithm

For each subject *j*, for each pair of structural and functional connectivity matrices ***S***_*j*_ and ***F***_*j*_ of dimensions *n* x *n* we used a methodology as described in Becker et al. (Becker et al., 2018) to assess the ability of structural connectivity to predict functional connectivity. The first step consisted of writing the predicted functional connectivity matrix as

$${\overset{︷}{F}}_{j}=R\;\left(\sum_{r=0}^{k}a_{r}S_{j}^{r}\right)\;R^{T}$$

The term $\sum_{r=0}^{k}\left(a_{r}S_{j}^{r}\right)$ represented a weighted sum of powers of ***S***_*j*_ up to order *k* (polynomial transformation of order *k*) and the rotation matrix ***R*** was used to transform the eigenvectors of the matrix ***S***_*j*_ in order to align to those of ***F***_*j*_. In the second step we solved the optimization problem by finding best approximation ${\overset{︷}{F}}_{j}$ that fits the original matrix ***F***_*j*_, that is, we solved the problem

$$\min_{{\left\{a_{r}\right\}}_{r=0}^{k},R}\;\left\|{\overset{︷}{F}}_{j}-F_{j}\right\|=\left\|R\;\left(\sum_{r=0}^{k}a_{r}S_{j}^{r}\right)\;R^{T}-F_{j}\right\|,R^{T}R=RR^{T}=I_{n},\det R=1\;$$

where ‖.‖ stands for the Frobenius norm, ***I***_***n***_ is the all‐ones diagonal matrix of dimension *n* and *det* stands for the determinant of the matrix. The constraints guarantee that the matrix ***R*** is a rotation matrix. Details are in Appendix. We quantified the goodness‐of‐fit between the predicted ${\overset{︷}{F}}_{j}$ and the real ***F***_*j*_ functional connectivity matrices using two methods. First we used correlation of the upper triangular entries obtained from the real matrix with those entries obtained from the predicted matrix. Second we used a homology‐based evaluation (Liang & Wang, 2017). This evaluation was based on comparing the number of connected components of the real and predicted functional connectivity matrices at different edge density levels *λ* (Betti numbers *β*_0_(*λ*) and $\hat{\beta_{0}\left(\lambda \right)}$, respectively). Here edge density referred to the percentage of correlation entries in the connectivity matrix. Therefore, zero density implied an empty matrix whereas the density of 1 indicated the existence of all correlation values in the matrix. *SSE*_*β*_ was used to evaluate the goodness‐of‐fit for the prediction. This was formulated as

$$\mathrm{SS}E_{\beta }=\frac{1}{n^{2}}\int_{0}^{1}(\beta_{0}\left(\lambda \right)-{\hat{\beta_{0}\left(\lambda \right))}}^{2}\;\mathrm{d\lambda}$$

where the integral spanned all edge densities from 0 (no correlation entries existing) to 1 (all correlation entries existing) and *n* is the dimension of the matrix. Practically, the smaller the score the better the fit of the predicted matrix to the real matrix as the number of different connected components is smaller.

Given this framework, we used optimal (NNG model), nonoptimal connections, and the entire structural connectome in place of ***S***_*j*_ and we obtained predicted functional connectivity matrices as above for each of the three cases. We then quantified the goodness of prediction using the two methods mentioned and compared the predictabilities between the three cases. More information is provided in the Appendix.

### 4.6 Controlling for distance

We conducted an additional analysis to control for the effect of distance in predicting functional connectivity. First we adopted a model for generating synthetic structural networks by penalizing the probability of a connection between pairs of regions *u* and *v* based on their distance that is, *P*(*u*, *v*) = *dist*(*u*, *v*)^−*η*^ where *η* is a parameter that regulates whether short or long range connections will be constructed and E is the Euclidean distance between *u* and *v* (Betzel et al., 2016). We fit generative models to the connectomes of individual participants following this procedure. Starting with an initial set of parameters *η* a set of synthetic networks was calculated using the previous distance rule. In turn, the parameter space was partitioned according to a Voronoi tessellation (Betzel et al., 2016). The energy of each Voronoi cell was calculated as the Kolmogorov–Smirnov distance between the real structural network and the corresponding synthetic networks. Cells were then chosen with probability inversely proportional to their energy, meaning that we chose cells with parameters that would give synthetic networks with a better fit. Next, new parameters were chosen from the selected cells and new synthetic networks were calculated. The above process was repeated twice in order to get a better fit between the distance model networks and the individual structural networks. Eventually, the synthetic network with the best fit was chosen for consequent analysis. Predicting functional connectivity matrices was conducted using these synthetic networks and this was compared to the predictability of the NNG models. Details are in Appendix.

To ensure that the comparison in the predictability between synthetic and NNG models was not driven but additional spurious distance correlates, we regressed out Euclidean distance from the predicted functional connectivity matrices *func*_*pred* in both models. For each subject we calculated the Euclidean matrix *dist*(*i*, *j*) where each entry represented the Euclidean distance between regions *i* and *j*. Then *func*_*new*_*pred* = (*func*_*pred* − *dist* * (*pinv*(*dist*) * *func*_*pred*)) was calculated where *pinv* is the pseudoinverse function. Finally, we recalculated the similarity between *func*_*new*_*pred* and the actual functional connectivity matrices for the synthetic and NNG models and treated this as the predictability number of each one of these models.

### 4.7 Gene expression samples and processing

The Allen Human Brain Atlas is a publicly available online resource of microarray‐based gene expression profiles for a set of predefined anatomical brain regions from the Allen Institute for Brain Science (AIBS) (Hawrylycz et al., 2012). The atlas is based on postmortem tissue from six donors with no known history of neurological or neuropsychiatric disease, who also passed a set of serology, toxicology, and RNA quality screens. Because only two donors had coverage in both hemispheres, we restricted our analysis to these two post mortem brains. We spatially matched the centroids of each region of the 234 Lausanne parcellation to the coordinates of the samples from the AIBS for each of the two donors. To do so, we converted the latter to voxel space and we overlapped their locations with the masks obtained from each region of the parcellation. A total of 81 regions did not match sample locations in either one of the two post mortem brains and thus were left out from the analysis to ensure robustness. As the raw expression data contained multiple probes belonging to the same gene or included unidentified gene symbols, we used the gene list as presented in Whitaker et al. (Whitaker et al., 2016). For the remaining regions, expression data were averaged across all samples from all donors across both hemispheres. To relate optimality to gene expression data, we calculated the optimal minus nonoptimal degrees for each node (RO) and we averaged across 50 individuals. We then used a Partial Least Squares‐PLS analysis to explain the variance in the optimal minus nonoptimal degree. PLS uses linear combinations of the gene expression scores (components) that predict the covariance between regional optimality and genes the most (Hastie, Tibishirani, & Friedman, 2001). We used gene enrichment analysis and visualization tools to identify significant gene ontology terms in the genes derived by the PLS component that explained the variance the most. Details are in Appendix.

### 4.8 Statistics

Statistical parameters including the definitions and exact values of sample size are reported in the main text, the figures and their corresponding legends. Prediction accuracies were compared using one‐way ANOVAs with one repeated (optimal, nonoptimal, whole‐brain connections) measure. Data were checked for normality using the Kolmogorov–Smirnov test before applying ANOVA. The obtained P values were corrected for violations of sphericity using a Greenhouse–Geisser correction. Post hoc tests were corrected for multiple comparisons using the Bonferroni test. For comparison of the predictive ability of the NNG to other connections and models, two sample t‐tests were applied. For comparison of the predictive ability of the NNG during task to the one during resting‐state, paired t‐tests were used. PLS analysis was computed using the *plsregress* function in MATLAB (The MathWorks, Inc., Natick, MA) with a 10‐fold validation setting. The *p*‐value was calculated by contrasting the variance explained in the predicted variable against a null model consisting of 1,000 permutations of the label assignment between the gene and RO data (Details are in Appendix).

## CONFLICT OF INTEREST

The authors declare no conflicts of interest.

## Supporting information


**Appendix** **S1:** Supplementary Information

## Data Availability

Data is open‐access and can be downloaded from the Human Connectome Project website. Code was built using custom‐made based scripts on top of publicly available code (cited when available). Code will be publicly available on a github repository after publication.
