# Supplementary material for: Structural optimality and neurogenetic expression mediate functional dynamics in the human brain
Source: Hum Brain Mapp. 2020 Feb 6;41(8):2229–43. doi: 10.1002/hbm.24942 (PMC7267953; doi:10.1002/hbm.24942)
Supplement: Supplementary file 1 — Appendix S1: Supplementary Information [file HBM-41-2229-s001.zip › HBM_24942_Supplementary_File2_updated.docx]

a)

| **GO term** | **Description** | **P-value** | **FDR q-value** | **Enrichment (N, B, n, b)** | **Genes** |
| --- | --- | --- | --- | --- | --- |
| GO:0022836 | gated channel activity | 1.73E-14 | 7.8E-11 | 3.05 (16750,306,1114,62) | KCNH3 - potassium voltage-gated channel, subfamily h (eag-related), member 3 CHRNA7 - cholinergic receptor, nicotinic, alpha 7 (neuronal) P2RX6 - purinergic receptor p2x, ligand-gated ion channel, 6 PTK2B - protein tyrosine kinase 2 beta TRPM2 - transient receptor potential cation channel, subfamily m, member 2 KCNS1 - potassium voltage-gated channel, delayed-rectifier, subfamily s, member 1 KCNN2 - potassium intermediate/small conductance calcium-activated channel, subfamily n, member 2 KCNMB4 - potassium large conductance calcium-activated channel, subfamily m, beta member 4 KCNMA1 - potassium large conductance calcium-activated channel, subfamily m, alpha member 1 KCNH5 - potassium voltage-gated channel, subfamily h (eag-related), member 5 KCNN1 - potassium intermediate/small conductance calcium-activated channel, subfamily n, member 1 KCNIP3 - kv channel interacting protein 3, calsenilin ASIC2 - acid-sensing (proton-gated) ion channel 2 KCNT1 - potassium channel, subfamily t, member 1 CACNB4 - calcium channel, voltage-dependent, beta 4 subunit GABRA1 - gamma-aminobutyric acid (gaba) a receptor, alpha 1 HCN1 - hyperpolarization activated cyclic nucleotide-gated potassium channel 1 SCN3B - sodium channel, voltage-gated, type iii, beta subunit CACNA2D3 - calcium channel, voltage-dependent, alpha 2/delta subunit 3 GABRA4 - gamma-aminobutyric acid (gaba) a receptor, alpha 4 SLC17A7 - solute carrier family 17 (vesicular glutamate transporter), member 7 KCNH7 - potassium voltage-gated channel, subfamily h (eag-related), member 7 SHROOM2 - shroom family member 2 CACNG2 - calcium channel, voltage-dependent, gamma subunit 2 KCNQ5 - potassium voltage-gated channel, kqt-like subfamily, member 5 MCOLN1 - mucolipin 1 CACNG3 - calcium channel, voltage-dependent, gamma subunit 3 KCNA2 - potassium voltage-gated channel, shaker-related subfamily, member 2 KCNA1 - potassium voltage-gated channel, shaker-related subfamily, member 1 (episodic ataxia with myokymia) SCN2B - sodium channel, voltage-gated, type ii, beta subunit SCN1B - sodium channel, voltage-gated, type i, beta subunit PKD2L1 - polycystic kidney disease 2-like 1 GABRG3 - gamma-aminobutyric acid (gaba) a receptor, gamma 3 KCNV1 - potassium channel, subfamily v, member 1 KCNAB3 - potassium voltage-gated channel, shaker-related subfamily, beta member 3 ANO1 - anoctamin 1, calcium activated chloride channel GRIA4 - glutamate receptor, ionotropic, ampa 4 ITPR1 - inositol 1,4,5-trisphosphate receptor, type 1 GRIN3A - glutamate receptor, ionotropic, n-methyl-d-aspartate 3a CDK5 - cyclin-dependent kinase 5 GRIN2A - glutamate receptor, ionotropic, n-methyl d-aspartate 2a KCNJ12 - potassium inwardly-rectifying channel, subfamily j, member 12 KCNJ11 - potassium inwardly-rectifying channel, subfamily j, member 11 KCNK1 - potassium channel, subfamily k, member 1 GRIK3 - glutamate receptor, ionotropic, kainate 3 GRIK4 - glutamate receptor, ionotropic, kainate 4 CLCN7 - chloride channel, voltage-sensitive 7 KCNJ4 - potassium inwardly-rectifying channel, subfamily j, member 4 KCNJ3 - potassium inwardly-rectifying channel, subfamily j, member 3 GLRA3 - glycine receptor, alpha 3 TRPM4 - transient receptor potential cation channel, subfamily m, member 4 CACNA1G - calcium channel, voltage-dependent, t type, alpha 1g subunit KCNJ9 - potassium inwardly-rectifying channel, subfamily j, member 9 KCNJ8 - potassium inwardly-rectifying channel, subfamily j, member 8 KCNJ6 - potassium inwardly-rectifying channel, subfamily j, member 6 KCNF1 - potassium voltage-gated channel, subfamily f, member 1 KCNH1 - potassium voltage-gated channel, subfamily h (eag-related), member 1 KCNB1 - potassium voltage-gated channel, shab-related subfamily, member 1 KCNC1 - potassium voltage-gated channel, shaw-related subfamily, member 1 KCNC2 - potassium voltage-gated channel, shaw-related subfamily, member 2 KCNK4 - potassium channel, subfamily k, member 4 KCNC3 - potassium voltage-gated channel, shaw-related subfamily, member 3 |
| GO:0005249 | voltage-gated potassium channel activity | 4.88E-14 | 1.1E-10 | 14.24 (16750,80,250,17) | KCNH3 - potassium voltage-gated channel, subfamily h (eag-related), member 3 KCNJ11 - potassium inwardly-rectifying channel, subfamily j, member 11 KCNJ4 - potassium inwardly-rectifying channel, subfamily j, member 4 HCN1 - hyperpolarization activated cyclic nucleotide-gated potassium channel 1 KCNJ3 - potassium inwardly-rectifying channel, subfamily j, member 3 KCNJ8 - potassium inwardly-rectifying channel, subfamily j, member 8 KCNJ6 - potassium inwardly-rectifying channel, subfamily j, member 6 KCNF1 - potassium voltage-gated channel, subfamily f, member 1 KCNS1 - potassium voltage-gated channel, delayed-rectifier, subfamily s, member 1 KCNH1 - potassium voltage-gated channel, subfamily h (eag-related), member 1 KCNC1 - potassium voltage-gated channel, shaw-related subfamily, member 1 KCNV1 - potassium channel, subfamily v, member 1 KCNAB3 - potassium voltage-gated channel, shaker-related subfamily, beta member 3 KCNH7 - potassium voltage-gated channel, subfamily h (eag-related), member 7 KCNC2 - potassium voltage-gated channel, shaw-related subfamily, member 2 KCNH5 - potassium voltage-gated channel, subfamily h (eag-related), member 5 KCNC3 - potassium voltage-gated channel, shaw-related subfamily, member 3 |
| GO:0005261 | cation channel activity | 4.94E-14 | 7.42E-11 | 3.12 (16750,292,1067,58) | KCNH3 - potassium voltage-gated channel, subfamily h (eag-related), member 3 CHRNA7 - cholinergic receptor, nicotinic, alpha 7 (neuronal) P2RX6 - purinergic receptor p2x, ligand-gated ion channel, 6 ABCC8 - atp-binding cassette, sub-family c (cftr/mrp), member 8 PTK2B - protein tyrosine kinase 2 beta TRPM2 - transient receptor potential cation channel, subfamily m, member 2 KCNS1 - potassium voltage-gated channel, delayed-rectifier, subfamily s, member 1 KCNN2 - potassium intermediate/small conductance calcium-activated channel, subfamily n, member 2 KCNMB4 - potassium large conductance calcium-activated channel, subfamily m, beta member 4 KCNMA1 - potassium large conductance calcium-activated channel, subfamily m, alpha member 1 KCNH5 - potassium voltage-gated channel, subfamily h (eag-related), member 5 KCNN1 - potassium intermediate/small conductance calcium-activated channel, subfamily n, member 1 ASIC2 - acid-sensing (proton-gated) ion channel 2 KCNIP3 - kv channel interacting protein 3, calsenilin KCNT1 - potassium channel, subfamily t, member 1 CACNB4 - calcium channel, voltage-dependent, beta 4 subunit HCN1 - hyperpolarization activated cyclic nucleotide-gated potassium channel 1 SCN3B - sodium channel, voltage-gated, type iii, beta subunit CACNA2D3 - calcium channel, voltage-dependent, alpha 2/delta subunit 3 KCNH7 - potassium voltage-gated channel, subfamily h (eag-related), member 7 SHROOM2 - shroom family member 2 CACNG2 - calcium channel, voltage-dependent, gamma subunit 2 KCNQ5 - potassium voltage-gated channel, kqt-like subfamily, member 5 KCNA2 - potassium voltage-gated channel, shaker-related subfamily, member 2 MCOLN1 - mucolipin 1 CACNG3 - calcium channel, voltage-dependent, gamma subunit 3 KCNA1 - potassium voltage-gated channel, shaker-related subfamily, member 1 (episodic ataxia with myokymia) SCN2B - sodium channel, voltage-gated, type ii, beta subunit SCN1B - sodium channel, voltage-gated, type i, beta subunit PKD2L1 - polycystic kidney disease 2-like 1 TRPV2 - transient receptor potential cation channel, subfamily v, member 2 KCNV1 - potassium channel, subfamily v, member 1 ANO1 - anoctamin 1, calcium activated chloride channel KCNAB3 - potassium voltage-gated channel, shaker-related subfamily, beta member 3 GRIA4 - glutamate receptor, ionotropic, ampa 4 ITPR1 - inositol 1,4,5-trisphosphate receptor, type 1 GRIN3A - glutamate receptor, ionotropic, n-methyl-d-aspartate 3a CDK5 - cyclin-dependent kinase 5 GRIN2A - glutamate receptor, ionotropic, n-methyl d-aspartate 2a KCNJ12 - potassium inwardly-rectifying channel, subfamily j, member 12 KCNJ11 - potassium inwardly-rectifying channel, subfamily j, member 11 KCNK1 - potassium channel, subfamily k, member 1 GRIK3 - glutamate receptor, ionotropic, kainate 3 GRIK4 - glutamate receptor, ionotropic, kainate 4 KCNJ4 - potassium inwardly-rectifying channel, subfamily j, member 4 KCNJ3 - potassium inwardly-rectifying channel, subfamily j, member 3 TRPM4 - transient receptor potential cation channel, subfamily m, member 4 KCNJ9 - potassium inwardly-rectifying channel, subfamily j, member 9 KCNJ8 - potassium inwardly-rectifying channel, subfamily j, member 8 KCNJ6 - potassium inwardly-rectifying channel, subfamily j, member 6 KCNF1 - potassium voltage-gated channel, subfamily f, member 1 PDE2A - phosphodiesterase 2a, cgmp-stimulated KCNH1 - potassium voltage-gated channel, subfamily h (eag-related), member 1 KCNB1 - potassium voltage-gated channel, shab-related subfamily, member 1 KCNC1 - potassium voltage-gated channel, shaw-related subfamily, member 1 KCNC2 - potassium voltage-gated channel, shaw-related subfamily, member 2 KCNK4 - potassium channel, subfamily k, member 4 KCNC3 - potassium voltage-gated channel, shaw-related subfamily, member 3 |
| GO:0005267 | potassium channel activity | 7.68E-14 | 8.65E-11 | 4.74 (16750,116,1067,35) | KCNH3 - potassium voltage-gated channel, subfamily h (eag-related), member 3 KCNA2 - potassium voltage-gated channel, shaker-related subfamily, member 2 KCNQ5 - potassium voltage-gated channel, kqt-like subfamily, member 5 KCNA1 - potassium voltage-gated channel, shaker-related subfamily, member 1 (episodic ataxia with myokymia) ABCC8 - atp-binding cassette, sub-family c (cftr/mrp), member 8 PKD2L1 - polycystic kidney disease 2-like 1 KCNS1 - potassium voltage-gated channel, delayed-rectifier, subfamily s, member 1 KCNN2 - potassium intermediate/small conductance calcium-activated channel, subfamily n, member 2 KCNAB3 - potassium voltage-gated channel, shaker-related subfamily, beta member 3 KCNV1 - potassium channel, subfamily v, member 1 KCNMB4 - potassium large conductance calcium-activated channel, subfamily m, beta member 4 KCNMA1 - potassium large conductance calcium-activated channel, subfamily m, alpha member 1 KCNH5 - potassium voltage-gated channel, subfamily h (eag-related), member 5 KCNN1 - potassium intermediate/small conductance calcium-activated channel, subfamily n, member 1 KCNIP3 - kv channel interacting protein 3, calsenilin KCNT1 - potassium channel, subfamily t, member 1 KCNJ12 - potassium inwardly-rectifying channel, subfamily j, member 12 KCNJ11 - potassium inwardly-rectifying channel, subfamily j, member 11 KCNK1 - potassium channel, subfamily k, member 1 GRIK3 - glutamate receptor, ionotropic, kainate 3 GRIK4 - glutamate receptor, ionotropic, kainate 4 KCNJ4 - potassium inwardly-rectifying channel, subfamily j, member 4 HCN1 - hyperpolarization activated cyclic nucleotide-gated potassium channel 1 KCNJ3 - potassium inwardly-rectifying channel, subfamily j, member 3 KCNJ9 - potassium inwardly-rectifying channel, subfamily j, member 9 KCNJ8 - potassium inwardly-rectifying channel, subfamily j, member 8 KCNJ6 - potassium inwardly-rectifying channel, subfamily j, member 6 KCNF1 - potassium voltage-gated channel, subfamily f, member 1 KCNH1 - potassium voltage-gated channel, subfamily h (eag-related), member 1 KCNB1 - potassium voltage-gated channel, shab-related subfamily, member 1 KCNC1 - potassium voltage-gated channel, shaw-related subfamily, member 1 KCNH7 - potassium voltage-gated channel, subfamily h (eag-related), member 7 KCNC2 - potassium voltage-gated channel, shaw-related subfamily, member 2 KCNC3 - potassium voltage-gated channel, shaw-related subfamily, member 3 KCNK4 - potassium channel, subfamily k, member 4 |
| GO:0022839 | ion gated channel activity | 1.14E-13 | 1.02E-10 | 3.14 (16750,297,1114,62) | KCNH3 - potassium voltage-gated channel, subfamily h (eag-related), member 3 CHRNA7 - cholinergic receptor, nicotinic, alpha 7 (neuronal) P2RX6 - purinergic receptor p2x, ligand-gated ion channel, 6 PTK2B - protein tyrosine kinase 2 beta TRPM2 - transient receptor potential cation channel, subfamily m, member 2 KCNS1 - potassium voltage-gated channel, delayed-rectifier, subfamily s, member 1 KCNN2 - potassium intermediate/small conductance calcium-activated channel, subfamily n, member 2 KCNMB4 - potassium large conductance calcium-activated channel, subfamily m, beta member 4 KCNMA1 - potassium large conductance calcium-activated channel, subfamily m, alpha member 1 KCNH5 - potassium voltage-gated channel, subfamily h (eag-related), member 5 KCNN1 - potassium intermediate/small conductance calcium-activated channel, subfamily n, member 1 KCNIP3 - kv channel interacting protein 3, calsenilin ASIC2 - acid-sensing (proton-gated) ion channel 2 KCNT1 - potassium channel, subfamily t, member 1 CACNB4 - calcium channel, voltage-dependent, beta 4 subunit GABRA1 - gamma-aminobutyric acid (gaba) a receptor, alpha 1 HCN1 - hyperpolarization activated cyclic nucleotide-gated potassium channel 1 SCN3B - sodium channel, voltage-gated, type iii, beta subunit CACNA2D3 - calcium channel, voltage-dependent, alpha 2/delta subunit 3 GABRA4 - gamma-aminobutyric acid (gaba) a receptor, alpha 4 SLC17A7 - solute carrier family 17 (vesicular glutamate transporter), member 7 KCNH7 - potassium voltage-gated channel, subfamily h (eag-related), member 7 SHROOM2 - shroom family member 2 CACNG2 - calcium channel, voltage-dependent, gamma subunit 2 KCNQ5 - potassium voltage-gated channel, kqt-like subfamily, member 5 MCOLN1 - mucolipin 1 KCNA2 - potassium voltage-gated channel, shaker-related subfamily, member 2 CACNG3 - calcium channel, voltage-dependent, gamma subunit 3 KCNA1 - potassium voltage-gated channel, shaker-related subfamily, member 1 (episodic ataxia with myokymia) SCN2B - sodium channel, voltage-gated, type ii, beta subunit SCN1B - sodium channel, voltage-gated, type i, beta subunit PKD2L1 - polycystic kidney disease 2-like 1 GABRG3 - gamma-aminobutyric acid (gaba) a receptor, gamma 3 KCNV1 - potassium channel, subfamily v, member 1 ANO1 - anoctamin 1, calcium activated chloride channel KCNAB3 - potassium voltage-gated channel, shaker-related subfamily, beta member 3 GRIA4 - glutamate receptor, ionotropic, ampa 4 ITPR1 - inositol 1,4,5-trisphosphate receptor, type 1 GRIN3A - glutamate receptor, ionotropic, n-methyl-d-aspartate 3a CDK5 - cyclin-dependent kinase 5 GRIN2A - glutamate receptor, ionotropic, n-methyl d-aspartate 2a KCNJ12 - potassium inwardly-rectifying channel, subfamily j, member 12 KCNJ11 - potassium inwardly-rectifying channel, subfamily j, member 11 KCNK1 - potassium channel, subfamily k, member 1 GRIK3 - glutamate receptor, ionotropic, kainate 3 GRIK4 - glutamate receptor, ionotropic, kainate 4 CLCN7 - chloride channel, voltage-sensitive 7 KCNJ4 - potassium inwardly-rectifying channel, subfamily j, member 4 GLRA3 - glycine receptor, alpha 3 KCNJ3 - potassium inwardly-rectifying channel, subfamily j, member 3 TRPM4 - transient receptor potential cation channel, subfamily m, member 4 CACNA1G - calcium channel, voltage-dependent, t type, alpha 1g subunit KCNJ9 - potassium inwardly-rectifying channel, subfamily j, member 9 KCNJ8 - potassium inwardly-rectifying channel, subfamily j, member 8 KCNJ6 - potassium inwardly-rectifying channel, subfamily j, member 6 KCNF1 - potassium voltage-gated channel, subfamily f, member 1 KCNH1 - potassium voltage-gated channel, subfamily h (eag-related), member 1 KCNB1 - potassium voltage-gated channel, shab-related subfamily, member 1 KCNC1 - potassium voltage-gated channel, shaw-related subfamily, member 1 KCNC2 - potassium voltage-gated channel, shaw-related subfamily, member 2 KCNK4 - potassium channel, subfamily k, member 4 KCNC3 - potassium voltage-gated channel, shaw-related subfamily, member 3 |
| GO:0022843 | voltage-gated cation channel activity | 1.64E-13 | 1.23E-10 | 10.19 (16750,126,261,20) | KCNH3 - potassium voltage-gated channel, subfamily h (eag-related), member 3 CACNG3 - calcium channel, voltage-dependent, gamma subunit 3 GRIN2A - glutamate receptor, ionotropic, n-methyl d-aspartate 2a KCNJ11 - potassium inwardly-rectifying channel, subfamily j, member 11 KCNJ4 - potassium inwardly-rectifying channel, subfamily j, member 4 HCN1 - hyperpolarization activated cyclic nucleotide-gated potassium channel 1 KCNJ3 - potassium inwardly-rectifying channel, subfamily j, member 3 KCNJ8 - potassium inwardly-rectifying channel, subfamily j, member 8 KCNJ6 - potassium inwardly-rectifying channel, subfamily j, member 6 KCNF1 - potassium voltage-gated channel, subfamily f, member 1 PTK2B - protein tyrosine kinase 2 beta KCNS1 - potassium voltage-gated channel, delayed-rectifier, subfamily s, member 1 KCNH1 - potassium voltage-gated channel, subfamily h (eag-related), member 1 KCNC1 - potassium voltage-gated channel, shaw-related subfamily, member 1 KCNAB3 - potassium voltage-gated channel, shaker-related subfamily, beta member 3 KCNV1 - potassium channel, subfamily v, member 1 KCNH7 - potassium voltage-gated channel, subfamily h (eag-related), member 7 KCNC2 - potassium voltage-gated channel, shaw-related subfamily, member 2 KCNH5 - potassium voltage-gated channel, subfamily h (eag-related), member 5 KCNC3 - potassium voltage-gated channel, shaw-related subfamily, member 3 |
| GO:0005216 | ion channel activity | 9.5E-13 | 6.11E-10 | 2.71 (16750,376,1084,66) | KCNH3 - potassium voltage-gated channel, subfamily h (eag-related), member 3 CHRNA7 - cholinergic receptor, nicotinic, alpha 7 (neuronal) P2RX6 - purinergic receptor p2x, ligand-gated ion channel, 6 ABCC8 - atp-binding cassette, sub-family c (cftr/mrp), member 8 PTK2B - protein tyrosine kinase 2 beta TRPM2 - transient receptor potential cation channel, subfamily m, member 2 KCNS1 - potassium voltage-gated channel, delayed-rectifier, subfamily s, member 1 KCNN2 - potassium intermediate/small conductance calcium-activated channel, subfamily n, member 2 KCNMB4 - potassium large conductance calcium-activated channel, subfamily m, beta member 4 KCNMA1 - potassium large conductance calcium-activated channel, subfamily m, alpha member 1 KCNH5 - potassium voltage-gated channel, subfamily h (eag-related), member 5 KCNN1 - potassium intermediate/small conductance calcium-activated channel, subfamily n, member 1 KCNIP3 - kv channel interacting protein 3, calsenilin ASIC2 - acid-sensing (proton-gated) ion channel 2 KCNT1 - potassium channel, subfamily t, member 1 CACNB4 - calcium channel, voltage-dependent, beta 4 subunit GABRA1 - gamma-aminobutyric acid (gaba) a receptor, alpha 1 HCN1 - hyperpolarization activated cyclic nucleotide-gated potassium channel 1 SCN3B - sodium channel, voltage-gated, type iii, beta subunit CACNA2D3 - calcium channel, voltage-dependent, alpha 2/delta subunit 3 GABRA4 - gamma-aminobutyric acid (gaba) a receptor, alpha 4 SLC17A7 - solute carrier family 17 (vesicular glutamate transporter), member 7 KCNH7 - potassium voltage-gated channel, subfamily h (eag-related), member 7 SHROOM2 - shroom family member 2 CACNG2 - calcium channel, voltage-dependent, gamma subunit 2 KCNQ5 - potassium voltage-gated channel, kqt-like subfamily, member 5 MCOLN1 - mucolipin 1 CACNG3 - calcium channel, voltage-dependent, gamma subunit 3 KCNA2 - potassium voltage-gated channel, shaker-related subfamily, member 2 KCNA1 - potassium voltage-gated channel, shaker-related subfamily, member 1 (episodic ataxia with myokymia) LRRC8B - leucine rich repeat containing 8 family, member b SCN2B - sodium channel, voltage-gated, type ii, beta subunit SCN1B - sodium channel, voltage-gated, type i, beta subunit PKD2L1 - polycystic kidney disease 2-like 1 GABRG3 - gamma-aminobutyric acid (gaba) a receptor, gamma 3 PIDD - p53-induced death domain protein TRPV2 - transient receptor potential cation channel, subfamily v, member 2 KCNV1 - potassium channel, subfamily v, member 1 KCNAB3 - potassium voltage-gated channel, shaker-related subfamily, beta member 3 ANO1 - anoctamin 1, calcium activated chloride channel GRIA4 - glutamate receptor, ionotropic, ampa 4 ITPR1 - inositol 1,4,5-trisphosphate receptor, type 1 GRIN3A - glutamate receptor, ionotropic, n-methyl-d-aspartate 3a CDK5 - cyclin-dependent kinase 5 GRIN2A - glutamate receptor, ionotropic, n-methyl d-aspartate 2a KCNJ12 - potassium inwardly-rectifying channel, subfamily j, member 12 KCNJ11 - potassium inwardly-rectifying channel, subfamily j, member 11 KCNK1 - potassium channel, subfamily k, member 1 GRIK3 - glutamate receptor, ionotropic, kainate 3 CLCN7 - chloride channel, voltage-sensitive 7 GRIK4 - glutamate receptor, ionotropic, kainate 4 KCNJ4 - potassium inwardly-rectifying channel, subfamily j, member 4 GLRA3 - glycine receptor, alpha 3 KCNJ3 - potassium inwardly-rectifying channel, subfamily j, member 3 TRPM4 - transient receptor potential cation channel, subfamily m, member 4 KCNJ9 - potassium inwardly-rectifying channel, subfamily j, member 9 KCNJ8 - potassium inwardly-rectifying channel, subfamily j, member 8 KCNJ6 - potassium inwardly-rectifying channel, subfamily j, member 6 KCNF1 - potassium voltage-gated channel, subfamily f, member 1 PDE2A - phosphodiesterase 2a, cgmp-stimulated KCNH1 - potassium voltage-gated channel, subfamily h (eag-related), member 1 KCNB1 - potassium voltage-gated channel, shab-related subfamily, member 1 KCNC1 - potassium voltage-gated channel, shaw-related subfamily, member 1 KCNC2 - potassium voltage-gated channel, shaw-related subfamily, member 2 KCNK4 - potassium channel, subfamily k, member 4 KCNC3 - potassium voltage-gated channel, shaw-related subfamily, member 3 |
| GO:0015079 | potassium ion transmembrane transporter activity | 1.05E-12 | 5.92E-10 | 6.01 (16750,143,507,26) | KCNH3 - potassium voltage-gated channel, subfamily h (eag-related), member 3 ATP1A1 - atpase, na+/k+ transporting, alpha 1 polypeptide ABCC8 - atp-binding cassette, sub-family c (cftr/mrp), member 8 PKD2L1 - polycystic kidney disease 2-like 1 KCNS1 - potassium voltage-gated channel, delayed-rectifier, subfamily s, member 1 KCNV1 - potassium channel, subfamily v, member 1 KCNAB3 - potassium voltage-gated channel, shaker-related subfamily, beta member 3 KCNMA1 - potassium large conductance calcium-activated channel, subfamily m, alpha member 1 KCNH5 - potassium voltage-gated channel, subfamily h (eag-related), member 5 KCNIP3 - kv channel interacting protein 3, calsenilin KCNT1 - potassium channel, subfamily t, member 1 KCNJ11 - potassium inwardly-rectifying channel, subfamily j, member 11 GRIK3 - glutamate receptor, ionotropic, kainate 3 GRIK4 - glutamate receptor, ionotropic, kainate 4 KCNJ4 - potassium inwardly-rectifying channel, subfamily j, member 4 HCN1 - hyperpolarization activated cyclic nucleotide-gated potassium channel 1 KCNJ3 - potassium inwardly-rectifying channel, subfamily j, member 3 KCNJ9 - potassium inwardly-rectifying channel, subfamily j, member 9 KCNJ8 - potassium inwardly-rectifying channel, subfamily j, member 8 KCNJ6 - potassium inwardly-rectifying channel, subfamily j, member 6 KCNF1 - potassium voltage-gated channel, subfamily f, member 1 KCNH1 - potassium voltage-gated channel, subfamily h (eag-related), member 1 KCNC1 - potassium voltage-gated channel, shaw-related subfamily, member 1 KCNH7 - potassium voltage-gated channel, subfamily h (eag-related), member 7 KCNC2 - potassium voltage-gated channel, shaw-related subfamily, member 2 KCNC3 - potassium voltage-gated channel, shaw-related subfamily, member 3 |
| GO:0022832 | voltage-gated channel activity | 1.57E-12 | 7.88E-10 | 3.59 (16750,176,1114,42) | KCNH3 - potassium voltage-gated channel, subfamily h (eag-related), member 3 CACNG2 - calcium channel, voltage-dependent, gamma subunit 2 CACNG3 - calcium channel, voltage-dependent, gamma subunit 3 KCNQ5 - potassium voltage-gated channel, kqt-like subfamily, member 5 KCNA2 - potassium voltage-gated channel, shaker-related subfamily, member 2 KCNA1 - potassium voltage-gated channel, shaker-related subfamily, member 1 (episodic ataxia with myokymia) SCN2B - sodium channel, voltage-gated, type ii, beta subunit SCN1B - sodium channel, voltage-gated, type i, beta subunit PTK2B - protein tyrosine kinase 2 beta KCNS1 - potassium voltage-gated channel, delayed-rectifier, subfamily s, member 1 ANO1 - anoctamin 1, calcium activated chloride channel KCNAB3 - potassium voltage-gated channel, shaker-related subfamily, beta member 3 KCNV1 - potassium channel, subfamily v, member 1 KCNMA1 - potassium large conductance calcium-activated channel, subfamily m, alpha member 1 KCNH5 - potassium voltage-gated channel, subfamily h (eag-related), member 5 GRIN3A - glutamate receptor, ionotropic, n-methyl-d-aspartate 3a KCNIP3 - kv channel interacting protein 3, calsenilin CDK5 - cyclin-dependent kinase 5 GRIN2A - glutamate receptor, ionotropic, n-methyl d-aspartate 2a KCNJ12 - potassium inwardly-rectifying channel, subfamily j, member 12 KCNT1 - potassium channel, subfamily t, member 1 KCNJ11 - potassium inwardly-rectifying channel, subfamily j, member 11 KCNK1 - potassium channel, subfamily k, member 1 CACNB4 - calcium channel, voltage-dependent, beta 4 subunit CLCN7 - chloride channel, voltage-sensitive 7 KCNJ4 - potassium inwardly-rectifying channel, subfamily j, member 4 KCNJ3 - potassium inwardly-rectifying channel, subfamily j, member 3 SCN3B - sodium channel, voltage-gated, type iii, beta subunit HCN1 - hyperpolarization activated cyclic nucleotide-gated potassium channel 1 CACNA2D3 - calcium channel, voltage-dependent, alpha 2/delta subunit 3 KCNJ9 - potassium inwardly-rectifying channel, subfamily j, member 9 CACNA1G - calcium channel, voltage-dependent, t type, alpha 1g subunit KCNJ8 - potassium inwardly-rectifying channel, subfamily j, member 8 KCNJ6 - potassium inwardly-rectifying channel, subfamily j, member 6 KCNF1 - potassium voltage-gated channel, subfamily f, member 1 KCNH1 - potassium voltage-gated channel, subfamily h (eag-related), member 1 KCNB1 - potassium voltage-gated channel, shab-related subfamily, member 1 KCNC1 - potassium voltage-gated channel, shaw-related subfamily, member 1 KCNH7 - potassium voltage-gated channel, subfamily h (eag-related), member 7 KCNC2 - potassium voltage-gated channel, shaw-related subfamily, member 2 KCNK4 - potassium channel, subfamily k, member 4 KCNC3 - potassium voltage-gated channel, shaw-related subfamily, member 3 |
| GO:0005244 | voltage-gated ion channel activity | 1.57E-12 | 7.09E-10 | 3.59 (16750,176,1114,42) | KCNH3 - potassium voltage-gated channel, subfamily h (eag-related), member 3 CACNG2 - calcium channel, voltage-dependent, gamma subunit 2 KCNA2 - potassium voltage-gated channel, shaker-related subfamily, member 2 KCNQ5 - potassium voltage-gated channel, kqt-like subfamily, member 5 CACNG3 - calcium channel, voltage-dependent, gamma subunit 3 KCNA1 - potassium voltage-gated channel, shaker-related subfamily, member 1 (episodic ataxia with myokymia) SCN2B - sodium channel, voltage-gated, type ii, beta subunit SCN1B - sodium channel, voltage-gated, type i, beta subunit PTK2B - protein tyrosine kinase 2 beta KCNS1 - potassium voltage-gated channel, delayed-rectifier, subfamily s, member 1 KCNAB3 - potassium voltage-gated channel, shaker-related subfamily, beta member 3 ANO1 - anoctamin 1, calcium activated chloride channel KCNV1 - potassium channel, subfamily v, member 1 KCNMA1 - potassium large conductance calcium-activated channel, subfamily m, alpha member 1 KCNH5 - potassium voltage-gated channel, subfamily h (eag-related), member 5 GRIN3A - glutamate receptor, ionotropic, n-methyl-d-aspartate 3a KCNIP3 - kv channel interacting protein 3, calsenilin CDK5 - cyclin-dependent kinase 5 KCNJ12 - potassium inwardly-rectifying channel, subfamily j, member 12 GRIN2A - glutamate receptor, ionotropic, n-methyl d-aspartate 2a KCNT1 - potassium channel, subfamily t, member 1 KCNJ11 - potassium inwardly-rectifying channel, subfamily j, member 11 KCNK1 - potassium channel, subfamily k, member 1 CACNB4 - calcium channel, voltage-dependent, beta 4 subunit CLCN7 - chloride channel, voltage-sensitive 7 KCNJ4 - potassium inwardly-rectifying channel, subfamily j, member 4 KCNJ3 - potassium inwardly-rectifying channel, subfamily j, member 3 SCN3B - sodium channel, voltage-gated, type iii, beta subunit HCN1 - hyperpolarization activated cyclic nucleotide-gated potassium channel 1 CACNA2D3 - calcium channel, voltage-dependent, alpha 2/delta subunit 3 KCNJ9 - potassium inwardly-rectifying channel, subfamily j, member 9 CACNA1G - calcium channel, voltage-dependent, t type, alpha 1g subunit KCNJ8 - potassium inwardly-rectifying channel, subfamily j, member 8 KCNJ6 - potassium inwardly-rectifying channel, subfamily j, member 6 KCNF1 - potassium voltage-gated channel, subfamily f, member 1 KCNH1 - potassium voltage-gated channel, subfamily h (eag-related), member 1 KCNB1 - potassium voltage-gated channel, shab-related subfamily, member 1 KCNC1 - potassium voltage-gated channel, shaw-related subfamily, member 1 KCNH7 - potassium voltage-gated channel, subfamily h (eag-related), member 7 KCNC2 - potassium voltage-gated channel, shaw-related subfamily, member 2 KCNC3 - potassium voltage-gated channel, shaw-related subfamily, member 3 KCNK4 - potassium channel, subfamily k, member 4 |
| GO:0022838 | substrate-specific channel activity | 2.06E-12 | 8.41E-10 | 2.66 (16750,383,1084,66) | KCNH3 - potassium voltage-gated channel, subfamily h (eag-related), member 3 CHRNA7 - cholinergic receptor, nicotinic, alpha 7 (neuronal) P2RX6 - purinergic receptor p2x, ligand-gated ion channel, 6 ABCC8 - atp-binding cassette, sub-family c (cftr/mrp), member 8 PTK2B - protein tyrosine kinase 2 beta TRPM2 - transient receptor potential cation channel, subfamily m, member 2 KCNS1 - potassium voltage-gated channel, delayed-rectifier, subfamily s, member 1 KCNN2 - potassium intermediate/small conductance calcium-activated channel, subfamily n, member 2 KCNMB4 - potassium large conductance calcium-activated channel, subfamily m, beta member 4 KCNMA1 - potassium large conductance calcium-activated channel, subfamily m, alpha member 1 KCNH5 - potassium voltage-gated channel, subfamily h (eag-related), member 5 KCNN1 - potassium intermediate/small conductance calcium-activated channel, subfamily n, member 1 KCNIP3 - kv channel interacting protein 3, calsenilin ASIC2 - acid-sensing (proton-gated) ion channel 2 KCNT1 - potassium channel, subfamily t, member 1 CACNB4 - calcium channel, voltage-dependent, beta 4 subunit GABRA1 - gamma-aminobutyric acid (gaba) a receptor, alpha 1 HCN1 - hyperpolarization activated cyclic nucleotide-gated potassium channel 1 SCN3B - sodium channel, voltage-gated, type iii, beta subunit CACNA2D3 - calcium channel, voltage-dependent, alpha 2/delta subunit 3 GABRA4 - gamma-aminobutyric acid (gaba) a receptor, alpha 4 SLC17A7 - solute carrier family 17 (vesicular glutamate transporter), member 7 KCNH7 - potassium voltage-gated channel, subfamily h (eag-related), member 7 SHROOM2 - shroom family member 2 CACNG2 - calcium channel, voltage-dependent, gamma subunit 2 KCNQ5 - potassium voltage-gated channel, kqt-like subfamily, member 5 MCOLN1 - mucolipin 1 CACNG3 - calcium channel, voltage-dependent, gamma subunit 3 KCNA2 - potassium voltage-gated channel, shaker-related subfamily, member 2 KCNA1 - potassium voltage-gated channel, shaker-related subfamily, member 1 (episodic ataxia with myokymia) LRRC8B - leucine rich repeat containing 8 family, member b SCN2B - sodium channel, voltage-gated, type ii, beta subunit SCN1B - sodium channel, voltage-gated, type i, beta subunit PKD2L1 - polycystic kidney disease 2-like 1 GABRG3 - gamma-aminobutyric acid (gaba) a receptor, gamma 3 PIDD - p53-induced death domain protein TRPV2 - transient receptor potential cation channel, subfamily v, member 2 KCNV1 - potassium channel, subfamily v, member 1 ANO1 - anoctamin 1, calcium activated chloride channel KCNAB3 - potassium voltage-gated channel, shaker-related subfamily, beta member 3 GRIA4 - glutamate receptor, ionotropic, ampa 4 ITPR1 - inositol 1,4,5-trisphosphate receptor, type 1 GRIN3A - glutamate receptor, ionotropic, n-methyl-d-aspartate 3a CDK5 - cyclin-dependent kinase 5 GRIN2A - glutamate receptor, ionotropic, n-methyl d-aspartate 2a KCNJ12 - potassium inwardly-rectifying channel, subfamily j, member 12 KCNJ11 - potassium inwardly-rectifying channel, subfamily j, member 11 KCNK1 - potassium channel, subfamily k, member 1 GRIK3 - glutamate receptor, ionotropic, kainate 3 GRIK4 - glutamate receptor, ionotropic, kainate 4 CLCN7 - chloride channel, voltage-sensitive 7 KCNJ4 - potassium inwardly-rectifying channel, subfamily j, member 4 GLRA3 - glycine receptor, alpha 3 KCNJ3 - potassium inwardly-rectifying channel, subfamily j, member 3 TRPM4 - transient receptor potential cation channel, subfamily m, member 4 KCNJ9 - potassium inwardly-rectifying channel, subfamily j, member 9 KCNJ8 - potassium inwardly-rectifying channel, subfamily j, member 8 KCNJ6 - potassium inwardly-rectifying channel, subfamily j, member 6 KCNF1 - potassium voltage-gated channel, subfamily f, member 1 PDE2A - phosphodiesterase 2a, cgmp-stimulated KCNH1 - potassium voltage-gated channel, subfamily h (eag-related), member 1 KCNB1 - potassium voltage-gated channel, shab-related subfamily, member 1 KCNC1 - potassium voltage-gated channel, shaw-related subfamily, member 1 KCNC2 - potassium voltage-gated channel, shaw-related subfamily, member 2 KCNK4 - potassium channel, subfamily k, member 4 KCNC3 - potassium voltage-gated channel, shaw-related subfamily, member 3 |
| GO:0046873 | metal ion transmembrane transporter activity | 1.97E-11 | 7.41E-9 | 2.56 (16750,398,1067,65) | KCNH3 - potassium voltage-gated channel, subfamily h (eag-related), member 3 SLC4A10 - solute carrier family 4, sodium bicarbonate transporter, member 10 SLC39A3 - solute carrier family 39 (zinc transporter), member 3 CHRNA7 - cholinergic receptor, nicotinic, alpha 7 (neuronal) SLC6A7 - solute carrier family 6 (neurotransmitter transporter), member 7 ATP1A1 - atpase, na+/k+ transporting, alpha 1 polypeptide SLC8A2 - solute carrier family 8 (sodium/calcium exchanger), member 2 ABCC8 - atp-binding cassette, sub-family c (cftr/mrp), member 8 KCNS1 - potassium voltage-gated channel, delayed-rectifier, subfamily s, member 1 TRPM2 - transient receptor potential cation channel, subfamily m, member 2 KCNN2 - potassium intermediate/small conductance calcium-activated channel, subfamily n, member 2 KCNMB4 - potassium large conductance calcium-activated channel, subfamily m, beta member 4 KCNMA1 - potassium large conductance calcium-activated channel, subfamily m, alpha member 1 KCNH5 - potassium voltage-gated channel, subfamily h (eag-related), member 5 KCNN1 - potassium intermediate/small conductance calcium-activated channel, subfamily n, member 1 KCNIP3 - kv channel interacting protein 3, calsenilin ASIC2 - acid-sensing (proton-gated) ion channel 2 KCNT1 - potassium channel, subfamily t, member 1 CACNB4 - calcium channel, voltage-dependent, beta 4 subunit SLC1A6 - solute carrier family 1 (high affinity aspartate/glutamate transporter), member 6 SCN3B - sodium channel, voltage-gated, type iii, beta subunit HCN1 - hyperpolarization activated cyclic nucleotide-gated potassium channel 1 CACNA2D3 - calcium channel, voltage-dependent, alpha 2/delta subunit 3 ATP2B3 - atpase, ca++ transporting, plasma membrane 3 KCNH7 - potassium voltage-gated channel, subfamily h (eag-related), member 7 SLC17A7 - solute carrier family 17 (vesicular glutamate transporter), member 7 SHROOM2 - shroom family member 2 CACNG2 - calcium channel, voltage-dependent, gamma subunit 2 KCNQ5 - potassium voltage-gated channel, kqt-like subfamily, member 5 MCOLN1 - mucolipin 1 CACNG3 - calcium channel, voltage-dependent, gamma subunit 3 KCNA2 - potassium voltage-gated channel, shaker-related subfamily, member 2 KCNA1 - potassium voltage-gated channel, shaker-related subfamily, member 1 (episodic ataxia with myokymia) SCN2B - sodium channel, voltage-gated, type ii, beta subunit SCN1B - sodium channel, voltage-gated, type i, beta subunit SLC39A14 - solute carrier family 39 (zinc transporter), member 14 PKD2L1 - polycystic kidney disease 2-like 1 TRPV2 - transient receptor potential cation channel, subfamily v, member 2 KCNV1 - potassium channel, subfamily v, member 1 TFR2 - transferrin receptor 2 KCNAB3 - potassium voltage-gated channel, shaker-related subfamily, beta member 3 ITPR1 - inositol 1,4,5-trisphosphate receptor, type 1 GRIN3A - glutamate receptor, ionotropic, n-methyl-d-aspartate 3a CDK5 - cyclin-dependent kinase 5 KCNJ12 - potassium inwardly-rectifying channel, subfamily j, member 12 GRIN2A - glutamate receptor, ionotropic, n-methyl d-aspartate 2a KCNJ11 - potassium inwardly-rectifying channel, subfamily j, member 11 KCNK1 - potassium channel, subfamily k, member 1 GRIK3 - glutamate receptor, ionotropic, kainate 3 GRIK4 - glutamate receptor, ionotropic, kainate 4 SLC30A3 - solute carrier family 30 (zinc transporter), member 3 KCNJ4 - potassium inwardly-rectifying channel, subfamily j, member 4 KCNJ3 - potassium inwardly-rectifying channel, subfamily j, member 3 TRPM4 - transient receptor potential cation channel, subfamily m, member 4 KCNJ9 - potassium inwardly-rectifying channel, subfamily j, member 9 KCNJ8 - potassium inwardly-rectifying channel, subfamily j, member 8 KCNJ6 - potassium inwardly-rectifying channel, subfamily j, member 6 KCNF1 - potassium voltage-gated channel, subfamily f, member 1 PDE2A - phosphodiesterase 2a, cgmp-stimulated KCNH1 - potassium voltage-gated channel, subfamily h (eag-related), member 1 KCNB1 - potassium voltage-gated channel, shab-related subfamily, member 1 KCNC1 - potassium voltage-gated channel, shaw-related subfamily, member 1 KCNC2 - potassium voltage-gated channel, shaw-related subfamily, member 2 KCNK4 - potassium channel, subfamily k, member 4 KCNC3 - potassium voltage-gated channel, shaw-related subfamily, member 3 |
| GO:0015267 | channel activity | 4.88E-11 | 1.69E-8 | 3.38 (16750,410,507,42) | KCNH3 - potassium voltage-gated channel, subfamily h (eag-related), member 3 CACNG3 - calcium channel, voltage-dependent, gamma subunit 3 CHRNA7 - cholinergic receptor, nicotinic, alpha 7 (neuronal) LRRC8B - leucine rich repeat containing 8 family, member b SCN1B - sodium channel, voltage-gated, type i, beta subunit P2RX6 - purinergic receptor p2x, ligand-gated ion channel, 6 ABCC8 - atp-binding cassette, sub-family c (cftr/mrp), member 8 PKD2L1 - polycystic kidney disease 2-like 1 PTK2B - protein tyrosine kinase 2 beta TRPM2 - transient receptor potential cation channel, subfamily m, member 2 KCNS1 - potassium voltage-gated channel, delayed-rectifier, subfamily s, member 1 TRPV2 - transient receptor potential cation channel, subfamily v, member 2 KCNV1 - potassium channel, subfamily v, member 1 KCNAB3 - potassium voltage-gated channel, shaker-related subfamily, beta member 3 ANO1 - anoctamin 1, calcium activated chloride channel KCNMA1 - potassium large conductance calcium-activated channel, subfamily m, alpha member 1 KCNH5 - potassium voltage-gated channel, subfamily h (eag-related), member 5 ITPR1 - inositol 1,4,5-trisphosphate receptor, type 1 KCNIP3 - kv channel interacting protein 3, calsenilin CDK5 - cyclin-dependent kinase 5 GRIN2A - glutamate receptor, ionotropic, n-methyl d-aspartate 2a KCNT1 - potassium channel, subfamily t, member 1 KCNJ11 - potassium inwardly-rectifying channel, subfamily j, member 11 GRIK3 - glutamate receptor, ionotropic, kainate 3 GRIK4 - glutamate receptor, ionotropic, kainate 4 KCNJ4 - potassium inwardly-rectifying channel, subfamily j, member 4 GABRA1 - gamma-aminobutyric acid (gaba) a receptor, alpha 1 HCN1 - hyperpolarization activated cyclic nucleotide-gated potassium channel 1 KCNJ3 - potassium inwardly-rectifying channel, subfamily j, member 3 KCNJ9 - potassium inwardly-rectifying channel, subfamily j, member 9 KCNJ8 - potassium inwardly-rectifying channel, subfamily j, member 8 GABRA4 - gamma-aminobutyric acid (gaba) a receptor, alpha 4 KCNJ6 - potassium inwardly-rectifying channel, subfamily j, member 6 KCNF1 - potassium voltage-gated channel, subfamily f, member 1 PDE2A - phosphodiesterase 2a, cgmp-stimulated KCNH1 - potassium voltage-gated channel, subfamily h (eag-related), member 1 KCNC1 - potassium voltage-gated channel, shaw-related subfamily, member 1 SLC17A7 - solute carrier family 17 (vesicular glutamate transporter), member 7 KCNH7 - potassium voltage-gated channel, subfamily h (eag-related), member 7 SHROOM2 - shroom family member 2 KCNC2 - potassium voltage-gated channel, shaw-related subfamily, member 2 KCNC3 - potassium voltage-gated channel, shaw-related subfamily, member 3 |
| GO:0022803 | passive transmembrane transporter activity | 5.07E-11 | 1.63E-8 | 3.38 (16750,411,507,42) | KCNH3 - potassium voltage-gated channel, subfamily h (eag-related), member 3 CACNG3 - calcium channel, voltage-dependent, gamma subunit 3 CHRNA7 - cholinergic receptor, nicotinic, alpha 7 (neuronal) LRRC8B - leucine rich repeat containing 8 family, member b SCN1B - sodium channel, voltage-gated, type i, beta subunit P2RX6 - purinergic receptor p2x, ligand-gated ion channel, 6 ABCC8 - atp-binding cassette, sub-family c (cftr/mrp), member 8 PKD2L1 - polycystic kidney disease 2-like 1 PTK2B - protein tyrosine kinase 2 beta TRPM2 - transient receptor potential cation channel, subfamily m, member 2 KCNS1 - potassium voltage-gated channel, delayed-rectifier, subfamily s, member 1 TRPV2 - transient receptor potential cation channel, subfamily v, member 2 KCNV1 - potassium channel, subfamily v, member 1 ANO1 - anoctamin 1, calcium activated chloride channel KCNAB3 - potassium voltage-gated channel, shaker-related subfamily, beta member 3 KCNMA1 - potassium large conductance calcium-activated channel, subfamily m, alpha member 1 KCNH5 - potassium voltage-gated channel, subfamily h (eag-related), member 5 ITPR1 - inositol 1,4,5-trisphosphate receptor, type 1 KCNIP3 - kv channel interacting protein 3, calsenilin CDK5 - cyclin-dependent kinase 5 GRIN2A - glutamate receptor, ionotropic, n-methyl d-aspartate 2a KCNT1 - potassium channel, subfamily t, member 1 KCNJ11 - potassium inwardly-rectifying channel, subfamily j, member 11 GRIK3 - glutamate receptor, ionotropic, kainate 3 GRIK4 - glutamate receptor, ionotropic, kainate 4 KCNJ4 - potassium inwardly-rectifying channel, subfamily j, member 4 GABRA1 - gamma-aminobutyric acid (gaba) a receptor, alpha 1 HCN1 - hyperpolarization activated cyclic nucleotide-gated potassium channel 1 KCNJ3 - potassium inwardly-rectifying channel, subfamily j, member 3 KCNJ9 - potassium inwardly-rectifying channel, subfamily j, member 9 KCNJ8 - potassium inwardly-rectifying channel, subfamily j, member 8 GABRA4 - gamma-aminobutyric acid (gaba) a receptor, alpha 4 KCNJ6 - potassium inwardly-rectifying channel, subfamily j, member 6 KCNF1 - potassium voltage-gated channel, subfamily f, member 1 PDE2A - phosphodiesterase 2a, cgmp-stimulated KCNH1 - potassium voltage-gated channel, subfamily h (eag-related), member 1 KCNC1 - potassium voltage-gated channel, shaw-related subfamily, member 1 SLC17A7 - solute carrier family 17 (vesicular glutamate transporter), member 7 KCNH7 - potassium voltage-gated channel, subfamily h (eag-related), member 7 SHROOM2 - shroom family member 2 KCNC2 - potassium voltage-gated channel, shaw-related subfamily, member 2 KCNC3 - potassium voltage-gated channel, shaw-related subfamily, member 3 |
| GO:0022890 | inorganic cation transmembrane transporter activity | 5.25E-10 | 1.58E-7 | 2.92 (16750,531,507,47) | KCNH3 - potassium voltage-gated channel, subfamily h (eag-related), member 3 CACNG3 - calcium channel, voltage-dependent, gamma subunit 3 CHRNA7 - cholinergic receptor, nicotinic, alpha 7 (neuronal) SLC6A7 - solute carrier family 6 (neurotransmitter transporter), member 7 SCN1B - sodium channel, voltage-gated, type i, beta subunit ATP1A1 - atpase, na+/k+ transporting, alpha 1 polypeptide P2RX6 - purinergic receptor p2x, ligand-gated ion channel, 6 SLC45A4 - solute carrier family 45, member 4 SLC8A2 - solute carrier family 8 (sodium/calcium exchanger), member 2 ABCC8 - atp-binding cassette, sub-family c (cftr/mrp), member 8 PKD2L1 - polycystic kidney disease 2-like 1 PTK2B - protein tyrosine kinase 2 beta TRPM2 - transient receptor potential cation channel, subfamily m, member 2 ATP6V0D1 - atpase, h+ transporting, lysosomal 38kda, v0 subunit d1 KCNS1 - potassium voltage-gated channel, delayed-rectifier, subfamily s, member 1 TRPV2 - transient receptor potential cation channel, subfamily v, member 2 KCNV1 - potassium channel, subfamily v, member 1 TFR2 - transferrin receptor 2 ANO1 - anoctamin 1, calcium activated chloride channel KCNAB3 - potassium voltage-gated channel, shaker-related subfamily, beta member 3 KCNMA1 - potassium large conductance calcium-activated channel, subfamily m, alpha member 1 KCNH5 - potassium voltage-gated channel, subfamily h (eag-related), member 5 ITPR1 - inositol 1,4,5-trisphosphate receptor, type 1 KCNIP3 - kv channel interacting protein 3, calsenilin CDK5 - cyclin-dependent kinase 5 GRIN2A - glutamate receptor, ionotropic, n-methyl d-aspartate 2a KCNT1 - potassium channel, subfamily t, member 1 KCNJ11 - potassium inwardly-rectifying channel, subfamily j, member 11 GRIK3 - glutamate receptor, ionotropic, kainate 3 GRIK4 - glutamate receptor, ionotropic, kainate 4 SLC30A3 - solute carrier family 30 (zinc transporter), member 3 KCNJ4 - potassium inwardly-rectifying channel, subfamily j, member 4 HCN1 - hyperpolarization activated cyclic nucleotide-gated potassium channel 1 KCNJ3 - potassium inwardly-rectifying channel, subfamily j, member 3 ATP6V1B1 - atpase, h+ transporting, lysosomal 56/58kda, v1 subunit b1 KCNJ9 - potassium inwardly-rectifying channel, subfamily j, member 9 KCNJ8 - potassium inwardly-rectifying channel, subfamily j, member 8 KCNJ6 - potassium inwardly-rectifying channel, subfamily j, member 6 KCNF1 - potassium voltage-gated channel, subfamily f, member 1 PDE2A - phosphodiesterase 2a, cgmp-stimulated KCNH1 - potassium voltage-gated channel, subfamily h (eag-related), member 1 KCNC1 - potassium voltage-gated channel, shaw-related subfamily, member 1 SLC17A7 - solute carrier family 17 (vesicular glutamate transporter), member 7 KCNH7 - potassium voltage-gated channel, subfamily h (eag-related), member 7 SHROOM2 - shroom family member 2 KCNC2 - potassium voltage-gated channel, shaw-related subfamily, member 2 KCNC3 - potassium voltage-gated channel, shaw-related subfamily, member 3 |
| GO:0008324 | cation transmembrane transporter activity | 5.98E-10 | 1.68E-7 | 2.27 (16750,575,924,72) | KCNH3 - potassium voltage-gated channel, subfamily h (eag-related), member 3 SLC4A10 - solute carrier family 4, sodium bicarbonate transporter, member 10 CHRNA7 - cholinergic receptor, nicotinic, alpha 7 (neuronal) SLC39A3 - solute carrier family 39 (zinc transporter), member 3 SLC6A7 - solute carrier family 6 (neurotransmitter transporter), member 7 P2RX6 - purinergic receptor p2x, ligand-gated ion channel, 6 ATP1A1 - atpase, na+/k+ transporting, alpha 1 polypeptide SLC8A2 - solute carrier family 8 (sodium/calcium exchanger), member 2 ABCC8 - atp-binding cassette, sub-family c (cftr/mrp), member 8 PTK2B - protein tyrosine kinase 2 beta TRPM2 - transient receptor potential cation channel, subfamily m, member 2 KCNS1 - potassium voltage-gated channel, delayed-rectifier, subfamily s, member 1 ATP6V0D1 - atpase, h+ transporting, lysosomal 38kda, v0 subunit d1 KCNMA1 - potassium large conductance calcium-activated channel, subfamily m, alpha member 1 KCNH5 - potassium voltage-gated channel, subfamily h (eag-related), member 5 SLC36A1 - solute carrier family 36 (proton/amino acid symporter), member 1 KCNIP3 - kv channel interacting protein 3, calsenilin ASIC2 - acid-sensing (proton-gated) ion channel 2 KCNT1 - potassium channel, subfamily t, member 1 RHCG - rh family, c glycoprotein SLC1A6 - solute carrier family 1 (high affinity aspartate/glutamate transporter), member 6 HCN1 - hyperpolarization activated cyclic nucleotide-gated potassium channel 1 ATP6V1B1 - atpase, h+ transporting, lysosomal 56/58kda, v1 subunit b1 ATP2B3 - atpase, ca++ transporting, plasma membrane 3 SLC17A7 - solute carrier family 17 (vesicular glutamate transporter), member 7 KCNH7 - potassium voltage-gated channel, subfamily h (eag-related), member 7 SHROOM2 - shroom family member 2 SLC25A19 - solute carrier family 25 (mitochondrial thiamine pyrophosphate carrier), member 19 CACNG2 - calcium channel, voltage-dependent, gamma subunit 2 KCNQ5 - potassium voltage-gated channel, kqt-like subfamily, member 5 CACNG3 - calcium channel, voltage-dependent, gamma subunit 3 MCOLN1 - mucolipin 1 KCNA2 - potassium voltage-gated channel, shaker-related subfamily, member 2 KCNA1 - potassium voltage-gated channel, shaker-related subfamily, member 1 (episodic ataxia with myokymia) SCN2B - sodium channel, voltage-gated, type ii, beta subunit SCN1B - sodium channel, voltage-gated, type i, beta subunit SLC45A4 - solute carrier family 45, member 4 SLC39A14 - solute carrier family 39 (zinc transporter), member 14 PKD2L1 - polycystic kidney disease 2-like 1 TRPV2 - transient receptor potential cation channel, subfamily v, member 2 TFR2 - transferrin receptor 2 KCNV1 - potassium channel, subfamily v, member 1 ANO1 - anoctamin 1, calcium activated chloride channel KCNAB3 - potassium voltage-gated channel, shaker-related subfamily, beta member 3 GRIA4 - glutamate receptor, ionotropic, ampa 4 ITPR1 - inositol 1,4,5-trisphosphate receptor, type 1 SLC38A7 - solute carrier family 38, member 7 GRIN3A - glutamate receptor, ionotropic, n-methyl-d-aspartate 3a CDK5 - cyclin-dependent kinase 5 KCNJ12 - potassium inwardly-rectifying channel, subfamily j, member 12 GRIN2A - glutamate receptor, ionotropic, n-methyl d-aspartate 2a KCNJ11 - potassium inwardly-rectifying channel, subfamily j, member 11 SLC45A1 - solute carrier family 45, member 1 SLC41A3 - solute carrier family 41, member 3 KCNK1 - potassium channel, subfamily k, member 1 GRIK3 - glutamate receptor, ionotropic, kainate 3 GRIK4 - glutamate receptor, ionotropic, kainate 4 SLC30A3 - solute carrier family 30 (zinc transporter), member 3 KCNJ4 - potassium inwardly-rectifying channel, subfamily j, member 4 KCNJ3 - potassium inwardly-rectifying channel, subfamily j, member 3 TRPM4 - transient receptor potential cation channel, subfamily m, member 4 KCNJ9 - potassium inwardly-rectifying channel, subfamily j, member 9 KCNJ8 - potassium inwardly-rectifying channel, subfamily j, member 8 KCNJ6 - potassium inwardly-rectifying channel, subfamily j, member 6 KCNF1 - potassium voltage-gated channel, subfamily f, member 1 PDE2A - phosphodiesterase 2a, cgmp-stimulated KCNH1 - potassium voltage-gated channel, subfamily h (eag-related), member 1 KCNB1 - potassium voltage-gated channel, shab-related subfamily, member 1 KCNC1 - potassium voltage-gated channel, shaw-related subfamily, member 1 KCNC2 - potassium voltage-gated channel, shaw-related subfamily, member 2 KCNK4 - potassium channel, subfamily k, member 4 KCNC3 - potassium voltage-gated channel, shaw-related subfamily, member 3 |
| GO:0015077 | monovalent inorganic cation transmembrane transporter activity | 2.31E-9 | 6.12E-7 | 3.40 (16750,340,507,35) | KCNH3 - potassium voltage-gated channel, subfamily h (eag-related), member 3 SLC6A7 - solute carrier family 6 (neurotransmitter transporter), member 7 SCN1B - sodium channel, voltage-gated, type i, beta subunit ATP1A1 - atpase, na+/k+ transporting, alpha 1 polypeptide SLC45A4 - solute carrier family 45, member 4 SLC8A2 - solute carrier family 8 (sodium/calcium exchanger), member 2 ABCC8 - atp-binding cassette, sub-family c (cftr/mrp), member 8 PKD2L1 - polycystic kidney disease 2-like 1 ATP6V0D1 - atpase, h+ transporting, lysosomal 38kda, v0 subunit d1 KCNS1 - potassium voltage-gated channel, delayed-rectifier, subfamily s, member 1 TRPM2 - transient receptor potential cation channel, subfamily m, member 2 KCNV1 - potassium channel, subfamily v, member 1 KCNAB3 - potassium voltage-gated channel, shaker-related subfamily, beta member 3 KCNMA1 - potassium large conductance calcium-activated channel, subfamily m, alpha member 1 KCNH5 - potassium voltage-gated channel, subfamily h (eag-related), member 5 KCNIP3 - kv channel interacting protein 3, calsenilin KCNT1 - potassium channel, subfamily t, member 1 KCNJ11 - potassium inwardly-rectifying channel, subfamily j, member 11 GRIK3 - glutamate receptor, ionotropic, kainate 3 GRIK4 - glutamate receptor, ionotropic, kainate 4 KCNJ4 - potassium inwardly-rectifying channel, subfamily j, member 4 HCN1 - hyperpolarization activated cyclic nucleotide-gated potassium channel 1 KCNJ3 - potassium inwardly-rectifying channel, subfamily j, member 3 ATP6V1B1 - atpase, h+ transporting, lysosomal 56/58kda, v1 subunit b1 KCNJ9 - potassium inwardly-rectifying channel, subfamily j, member 9 KCNJ8 - potassium inwardly-rectifying channel, subfamily j, member 8 KCNJ6 - potassium inwardly-rectifying channel, subfamily j, member 6 KCNF1 - potassium voltage-gated channel, subfamily f, member 1 KCNH1 - potassium voltage-gated channel, subfamily h (eag-related), member 1 KCNC1 - potassium voltage-gated channel, shaw-related subfamily, member 1 SLC17A7 - solute carrier family 17 (vesicular glutamate transporter), member 7 KCNH7 - potassium voltage-gated channel, subfamily h (eag-related), member 7 SHROOM2 - shroom family member 2 KCNC2 - potassium voltage-gated channel, shaw-related subfamily, member 2 KCNC3 - potassium voltage-gated channel, shaw-related subfamily, member 3 |
| GO:0015075 | ion transmembrane transporter activity | 3.2E-9 | 7.99E-7 | 1.95 (16750,782,1046,95) | KCNH3 - potassium voltage-gated channel, subfamily h (eag-related), member 3 SLC4A10 - solute carrier family 4, sodium bicarbonate transporter, member 10 SLC39A3 - solute carrier family 39 (zinc transporter), member 3 CHRNA7 - cholinergic receptor, nicotinic, alpha 7 (neuronal) SLC6A7 - solute carrier family 6 (neurotransmitter transporter), member 7 P2RX6 - purinergic receptor p2x, ligand-gated ion channel, 6 ATP1A1 - atpase, na+/k+ transporting, alpha 1 polypeptide SLC8A2 - solute carrier family 8 (sodium/calcium exchanger), member 2 ADAMTS8 - adam metallopeptidase with thrombospondin type 1 motif, 8 ABCC8 - atp-binding cassette, sub-family c (cftr/mrp), member 8 PTK2B - protein tyrosine kinase 2 beta TRPM2 - transient receptor potential cation channel, subfamily m, member 2 ATP6V0D1 - atpase, h+ transporting, lysosomal 38kda, v0 subunit d1 KCNS1 - potassium voltage-gated channel, delayed-rectifier, subfamily s, member 1 KCNMB4 - potassium large conductance calcium-activated channel, subfamily m, beta member 4 KCNMA1 - potassium large conductance calcium-activated channel, subfamily m, alpha member 1 SLC36A1 - solute carrier family 36 (proton/amino acid symporter), member 1 KCNH5 - potassium voltage-gated channel, subfamily h (eag-related), member 5 KCNN1 - potassium intermediate/small conductance calcium-activated channel, subfamily n, member 1 KCNIP3 - kv channel interacting protein 3, calsenilin ASIC2 - acid-sensing (proton-gated) ion channel 2 SLC26A10 - solute carrier family 26, member 10 KCNT1 - potassium channel, subfamily t, member 1 SLC37A4 - solute carrier family 37 (glucose-6-phosphate transporter), member 4 SLCO4A1 - solute carrier organic anion transporter family, member 4a1 CACNB4 - calcium channel, voltage-dependent, beta 4 subunit SLC25A11 - solute carrier family 25 (mitochondrial carrier; oxoglutarate carrier), member 11 RHCG - rh family, c glycoprotein SLC1A6 - solute carrier family 1 (high affinity aspartate/glutamate transporter), member 6 GABRA1 - gamma-aminobutyric acid (gaba) a receptor, alpha 1 SCN3B - sodium channel, voltage-gated, type iii, beta subunit HCN1 - hyperpolarization activated cyclic nucleotide-gated potassium channel 1 CACNA2D3 - calcium channel, voltage-dependent, alpha 2/delta subunit 3 SLC38A11 - solute carrier family 38, member 11 ATP6V1B1 - atpase, h+ transporting, lysosomal 56/58kda, v1 subunit b1 GABRA4 - gamma-aminobutyric acid (gaba) a receptor, alpha 4 ATP2B3 - atpase, ca++ transporting, plasma membrane 3 SLC17A7 - solute carrier family 17 (vesicular glutamate transporter), member 7 KCNH7 - potassium voltage-gated channel, subfamily h (eag-related), member 7 SHROOM2 - shroom family member 2 SLC25A19 - solute carrier family 25 (mitochondrial thiamine pyrophosphate carrier), member 19 CACNG2 - calcium channel, voltage-dependent, gamma subunit 2 KCNQ5 - potassium voltage-gated channel, kqt-like subfamily, member 5 MCOLN1 - mucolipin 1 SLC25A38 - solute carrier family 25, member 38 CACNG3 - calcium channel, voltage-dependent, gamma subunit 3 KCNA2 - potassium voltage-gated channel, shaker-related subfamily, member 2 KCNA1 - potassium voltage-gated channel, shaker-related subfamily, member 1 (episodic ataxia with myokymia) LRRC8B - leucine rich repeat containing 8 family, member b SCN2B - sodium channel, voltage-gated, type ii, beta subunit SCN1B - sodium channel, voltage-gated, type i, beta subunit SLC45A4 - solute carrier family 45, member 4 SLC39A14 - solute carrier family 39 (zinc transporter), member 14 PKD2L1 - polycystic kidney disease 2-like 1 GABRG3 - gamma-aminobutyric acid (gaba) a receptor, gamma 3 PIDD - p53-induced death domain protein TRPV2 - transient receptor potential cation channel, subfamily v, member 2 SLC16A7 - solute carrier family 16 (monocarboxylate transporter), member 7 TFR2 - transferrin receptor 2 KCNAB3 - potassium voltage-gated channel, shaker-related subfamily, beta member 3 ANO1 - anoctamin 1, calcium activated chloride channel KCNV1 - potassium channel, subfamily v, member 1 GRIA4 - glutamate receptor, ionotropic, ampa 4 SLC38A7 - solute carrier family 38, member 7 ITPR1 - inositol 1,4,5-trisphosphate receptor, type 1 SLC26A4 - solute carrier family 26 (anion exchanger), member 4 GRIN3A - glutamate receptor, ionotropic, n-methyl-d-aspartate 3a CDK5 - cyclin-dependent kinase 5 GRIN2A - glutamate receptor, ionotropic, n-methyl d-aspartate 2a KCNJ12 - potassium inwardly-rectifying channel, subfamily j, member 12 KCNJ11 - potassium inwardly-rectifying channel, subfamily j, member 11 SLC45A1 - solute carrier family 45, member 1 SLC41A3 - solute carrier family 41, member 3 KCNK1 - potassium channel, subfamily k, member 1 GRIK3 - glutamate receptor, ionotropic, kainate 3 GRIK4 - glutamate receptor, ionotropic, kainate 4 SLC30A3 - solute carrier family 30 (zinc transporter), member 3 KCNJ4 - potassium inwardly-rectifying channel, subfamily j, member 4 KCNJ3 - potassium inwardly-rectifying channel, subfamily j, member 3 GLRA3 - glycine receptor, alpha 3 TRPM4 - transient receptor potential cation channel, subfamily m, member 4 SLC25A41 - solute carrier family 25, member 41 KCNJ9 - potassium inwardly-rectifying channel, subfamily j, member 9 KCNJ8 - potassium inwardly-rectifying channel, subfamily j, member 8 KCNJ6 - potassium inwardly-rectifying channel, subfamily j, member 6 KCNF1 - potassium voltage-gated channel, subfamily f, member 1 PDE2A - phosphodiesterase 2a, cgmp-stimulated KCNH1 - potassium voltage-gated channel, subfamily h (eag-related), member 1 KCNB1 - potassium voltage-gated channel, shab-related subfamily, member 1 SLC27A2 - solute carrier family 27 (fatty acid transporter), member 2 KCNC1 - potassium voltage-gated channel, shaw-related subfamily, member 1 SLC25A5 - solute carrier family 25 (mitochondrial carrier; adenine nucleotide translocator), member 5 KCNC2 - potassium voltage-gated channel, shaw-related subfamily, member 2 KCNK4 - potassium channel, subfamily k, member 4 KCNC3 - potassium voltage-gated channel, shaw-related subfamily, member 3 |
| GO:0022834 | ligand-gated channel activity | 3.22E-9 | 7.62E-7 | 4.53 (16750,120,770,25) | MCOLN1 - mucolipin 1 CHRNA7 - cholinergic receptor, nicotinic, alpha 7 (neuronal) P2RX6 - purinergic receptor p2x, ligand-gated ion channel, 6 GABRG3 - gamma-aminobutyric acid (gaba) a receptor, gamma 3 PTK2B - protein tyrosine kinase 2 beta TRPM2 - transient receptor potential cation channel, subfamily m, member 2 GRIA4 - glutamate receptor, ionotropic, ampa 4 ITPR1 - inositol 1,4,5-trisphosphate receptor, type 1 GRIN3A - glutamate receptor, ionotropic, n-methyl-d-aspartate 3a KCNJ12 - potassium inwardly-rectifying channel, subfamily j, member 12 GRIN2A - glutamate receptor, ionotropic, n-methyl d-aspartate 2a KCNJ11 - potassium inwardly-rectifying channel, subfamily j, member 11 GRIK3 - glutamate receptor, ionotropic, kainate 3 GRIK4 - glutamate receptor, ionotropic, kainate 4 GABRA1 - gamma-aminobutyric acid (gaba) a receptor, alpha 1 KCNJ4 - potassium inwardly-rectifying channel, subfamily j, member 4 HCN1 - hyperpolarization activated cyclic nucleotide-gated potassium channel 1 KCNJ3 - potassium inwardly-rectifying channel, subfamily j, member 3 GLRA3 - glycine receptor, alpha 3 KCNJ9 - potassium inwardly-rectifying channel, subfamily j, member 9 GABRA4 - gamma-aminobutyric acid (gaba) a receptor, alpha 4 KCNJ8 - potassium inwardly-rectifying channel, subfamily j, member 8 KCNJ6 - potassium inwardly-rectifying channel, subfamily j, member 6 SLC17A7 - solute carrier family 17 (vesicular glutamate transporter), member 7 SHROOM2 - shroom family member 2 |
| GO:0015276 | ligand-gated ion channel activity | 3.22E-9 | 7.24E-7 | 4.53 (16750,120,770,25) | MCOLN1 - mucolipin 1 CHRNA7 - cholinergic receptor, nicotinic, alpha 7 (neuronal) P2RX6 - purinergic receptor p2x, ligand-gated ion channel, 6 GABRG3 - gamma-aminobutyric acid (gaba) a receptor, gamma 3 PTK2B - protein tyrosine kinase 2 beta TRPM2 - transient receptor potential cation channel, subfamily m, member 2 GRIA4 - glutamate receptor, ionotropic, ampa 4 ITPR1 - inositol 1,4,5-trisphosphate receptor, type 1 GRIN3A - glutamate receptor, ionotropic, n-methyl-d-aspartate 3a KCNJ12 - potassium inwardly-rectifying channel, subfamily j, member 12 GRIN2A - glutamate receptor, ionotropic, n-methyl d-aspartate 2a KCNJ11 - potassium inwardly-rectifying channel, subfamily j, member 11 GRIK3 - glutamate receptor, ionotropic, kainate 3 GRIK4 - glutamate receptor, ionotropic, kainate 4 GABRA1 - gamma-aminobutyric acid (gaba) a receptor, alpha 1 KCNJ4 - potassium inwardly-rectifying channel, subfamily j, member 4 HCN1 - hyperpolarization activated cyclic nucleotide-gated potassium channel 1 GLRA3 - glycine receptor, alpha 3 KCNJ3 - potassium inwardly-rectifying channel, subfamily j, member 3 KCNJ9 - potassium inwardly-rectifying channel, subfamily j, member 9 GABRA4 - gamma-aminobutyric acid (gaba) a receptor, alpha 4 KCNJ8 - potassium inwardly-rectifying channel, subfamily j, member 8 KCNJ6 - potassium inwardly-rectifying channel, subfamily j, member 6 SLC17A7 - solute carrier family 17 (vesicular glutamate transporter), member 7 SHROOM2 - shroom family member 2 |
| GO:0022857 | transmembrane transporter activity | 4.54E-9 | 9.74E-7 | 1.68 (16750,937,1513,142) | SLC4A10 - solute carrier family 4, sodium bicarbonate transporter, member 10 SLC22A18 - solute carrier family 22, member 18 SLC22A10 - solute carrier family 22, member 10 SLC29A1 - solute carrier family 29 (equilibrative nucleoside transporter), member 1 ABCA4 - atp-binding cassette, sub-family a (abc1), member 4 TRPM2 - transient receptor potential cation channel, subfamily m, member 2 ADC - arginine decarboxylase KCNMB4 - potassium large conductance calcium-activated channel, subfamily m, beta member 4 ASIC2 - acid-sensing (proton-gated) ion channel 2 SV2B - synaptic vesicle glycoprotein 2b SLC46A2 - solute carrier family 46, member 2 OCA2 - oculocutaneous albinism ii SLCO4A1 - solute carrier organic anion transporter family, member 4a1 STEAP1 - six transmembrane epithelial antigen of the prostate 1 SCN3B - sodium channel, voltage-gated, type iii, beta subunit CACNA2D3 - calcium channel, voltage-dependent, alpha 2/delta subunit 3 SLC38A11 - solute carrier family 38, member 11 SLC35C2 - solute carrier family 35 (gdp-fucose transporter), member c2 KCNH7 - potassium voltage-gated channel, subfamily h (eag-related), member 7 SHROOM2 - shroom family member 2 SCN1A - sodium channel, voltage-gated, type i, alpha subunit SCN2B - sodium channel, voltage-gated, type ii, beta subunit SCN1B - sodium channel, voltage-gated, type i, beta subunit ABCB8 - atp-binding cassette, sub-family b (mdr/tap), member 8 PIDD - p53-induced death domain protein TRPV2 - transient receptor potential cation channel, subfamily v, member 2 MFSD4 - major facilitator superfamily domain containing 4 SLC16A7 - solute carrier family 16 (monocarboxylate transporter), member 7 ANO1 - anoctamin 1, calcium activated chloride channel KCNAB3 - potassium voltage-gated channel, shaker-related subfamily, beta member 3 GRIN3A - glutamate receptor, ionotropic, n-methyl-d-aspartate 3a SLC26A4 - solute carrier family 26 (anion exchanger), member 4 TIMM17B - translocase of inner mitochondrial membrane 17 homolog b (yeast) ASNA1 - arsa arsenite transporter, atp-binding, homolog 1 (bacterial) CDK5 - cyclin-dependent kinase 5 SVOP - sv2 related protein homolog (rat) SLC2A11 - solute carrier family 2 (facilitated glucose transporter), member 11 SLC30A3 - solute carrier family 30 (zinc transporter), member 3 TRPM4 - transient receptor potential cation channel, subfamily m, member 4 CACNA1G - calcium channel, voltage-dependent, t type, alpha 1g subunit SLC2A13 - solute carrier family 2 (facilitated glucose transporter), member 13 PDE2A - phosphodiesterase 2a, cgmp-stimulated TIMM22 - translocase of inner mitochondrial membrane 22 homolog (yeast) SLC4A8 - solute carrier family 4, sodium bicarbonate cotransporter, member 8 SLC4A7 - solute carrier family 4, sodium bicarbonate cotransporter, member 7 KCNH3 - potassium voltage-gated channel, subfamily h (eag-related), member 3 SLC25A26 - solute carrier family 25 (s-adenosylmethionine carrier), member 26 SLC39A3 - solute carrier family 39 (zinc transporter), member 3 CHRNA7 - cholinergic receptor, nicotinic, alpha 7 (neuronal) SLC6A7 - solute carrier family 6 (neurotransmitter transporter), member 7 RTBDN - retbindin KCNMB3 - potassium large conductance calcium-activated channel, subfamily m beta member 3 ATP12A - atpase, h+/k+ transporting, nongastric, alpha polypeptide P2RX6 - purinergic receptor p2x, ligand-gated ion channel, 6 ATP1A1 - atpase, na+/k+ transporting, alpha 1 polypeptide TIMM17A - translocase of inner mitochondrial membrane 17 homolog a (yeast) SLC8A2 - solute carrier family 8 (sodium/calcium exchanger), member 2 ADAMTS8 - adam metallopeptidase with thrombospondin type 1 motif, 8 SLC6A17 - solute carrier family 6 (neutral amino acid transporter), member 17 ABCC8 - atp-binding cassette, sub-family c (cftr/mrp), member 8 CACNG7 - calcium channel, voltage-dependent, gamma subunit 7 SLC9A5 - solute carrier family 9, subfamily a (nhe5, cation proton antiporter 5), member 5 SLC35F1 - solute carrier family 35, member f1 PTK2B - protein tyrosine kinase 2 beta ATP6V0D1 - atpase, h+ transporting, lysosomal 38kda, v0 subunit d1 KCNS1 - potassium voltage-gated channel, delayed-rectifier, subfamily s, member 1 KCNN2 - potassium intermediate/small conductance calcium-activated channel, subfamily n, member 2 KCNK3 - potassium channel, subfamily k, member 3 KCNMA1 - potassium large conductance calcium-activated channel, subfamily m, alpha member 1 SLC36A1 - solute carrier family 36 (proton/amino acid symporter), member 1 KCNH5 - potassium voltage-gated channel, subfamily h (eag-related), member 5 SLC6A20 - solute carrier family 6 (proline imino transporter), member 20 KCNN1 - potassium intermediate/small conductance calcium-activated channel, subfamily n, member 1 KCNIP3 - kv channel interacting protein 3, calsenilin SLC26A10 - solute carrier family 26, member 10 KCNT1 - potassium channel, subfamily t, member 1 SLC37A4 - solute carrier family 37 (glucose-6-phosphate transporter), member 4 CACNB4 - calcium channel, voltage-dependent, beta 4 subunit SLC26A6 - solute carrier family 26 (anion exchanger), member 6 SLC29A4 - solute carrier family 29 (equilibrative nucleoside transporter), member 4 SLC25A11 - solute carrier family 25 (mitochondrial carrier; oxoglutarate carrier), member 11 SLC1A6 - solute carrier family 1 (high affinity aspartate/glutamate transporter), member 6 RHCG - rh family, c glycoprotein GABRA1 - gamma-aminobutyric acid (gaba) a receptor, alpha 1 HCN1 - hyperpolarization activated cyclic nucleotide-gated potassium channel 1 ATP6V1B1 - atpase, h+ transporting, lysosomal 56/58kda, v1 subunit b1 GABRA4 - gamma-aminobutyric acid (gaba) a receptor, alpha 4 GABRA3 - gamma-aminobutyric acid (gaba) a receptor, alpha 3 ATP2B3 - atpase, ca++ transporting, plasma membrane 3 SLC17A7 - solute carrier family 17 (vesicular glutamate transporter), member 7 SLC25A19 - solute carrier family 25 (mitochondrial thiamine pyrophosphate carrier), member 19 MFSD12 - major facilitator superfamily domain containing 12 CACNG2 - calcium channel, voltage-dependent, gamma subunit 2 KCNQ5 - potassium voltage-gated channel, kqt-like subfamily, member 5 MCOLN1 - mucolipin 1 SLC25A38 - solute carrier family 25, member 38 CACNG3 - calcium channel, voltage-dependent, gamma subunit 3 KCNA2 - potassium voltage-gated channel, shaker-related subfamily, member 2 KCNA1 - potassium voltage-gated channel, shaker-related subfamily, member 1 (episodic ataxia with myokymia) LRRC8B - leucine rich repeat containing 8 family, member b SLC25A39 - solute carrier family 25, member 39 SLC45A4 - solute carrier family 45, member 4 SLC39A14 - solute carrier family 39 (zinc transporter), member 14 SLC25A25 - solute carrier family 25 (mitochondrial carrier; phosphate carrier), member 25 PKD2L1 - polycystic kidney disease 2-like 1 GABRG3 - gamma-aminobutyric acid (gaba) a receptor, gamma 3 TFR2 - transferrin receptor 2 KCNV1 - potassium channel, subfamily v, member 1 GRIA4 - glutamate receptor, ionotropic, ampa 4 GRIA3 - glutamate receptor, ionotropic, ampa 3 SLC38A7 - solute carrier family 38, member 7 ITPR1 - inositol 1,4,5-trisphosphate receptor, type 1 ABCC10 - atp-binding cassette, sub-family c (cftr/mrp), member 10 GRIN2A - glutamate receptor, ionotropic, n-methyl d-aspartate 2a KCNJ12 - potassium inwardly-rectifying channel, subfamily j, member 12 KCNJ11 - potassium inwardly-rectifying channel, subfamily j, member 11 SLC45A1 - solute carrier family 45, member 1 GRIK1 - glutamate receptor, ionotropic, kainate 1 SLC41A3 - solute carrier family 41, member 3 GRIK2 - glutamate receptor, ionotropic, kainate 2 KCNK1 - potassium channel, subfamily k, member 1 GRIK3 - glutamate receptor, ionotropic, kainate 3 GRIK4 - glutamate receptor, ionotropic, kainate 4 CLCN7 - chloride channel, voltage-sensitive 7 SLC25A42 - solute carrier family 25, member 42 KCNJ4 - potassium inwardly-rectifying channel, subfamily j, member 4 GLRA3 - glycine receptor, alpha 3 KCNJ3 - potassium inwardly-rectifying channel, subfamily j, member 3 SLC25A41 - solute carrier family 25, member 41 KCNJ9 - potassium inwardly-rectifying channel, subfamily j, member 9 KCNJ8 - potassium inwardly-rectifying channel, subfamily j, member 8 KCNJ6 - potassium inwardly-rectifying channel, subfamily j, member 6 KCNF1 - potassium voltage-gated channel, subfamily f, member 1 ATP13A1 - atpase type 13a1 KCNH1 - potassium voltage-gated channel, subfamily h (eag-related), member 1 SLC27A2 - solute carrier family 27 (fatty acid transporter), member 2 KCNB1 - potassium voltage-gated channel, shab-related subfamily, member 1 SLC25A5 - solute carrier family 25 (mitochondrial carrier; adenine nucleotide translocator), member 5 KCNC1 - potassium voltage-gated channel, shaw-related subfamily, member 1 KCNC2 - potassium voltage-gated channel, shaw-related subfamily, member 2 KCNK4 - potassium channel, subfamily k, member 4 KCNC3 - potassium voltage-gated channel, shaw-related subfamily, member 3 |
| GO:0015318 | inorganic molecular entity transmembrane transporter activity | 7.66E-9 | 1.57E-6 | 1.96 (16750,727,1046,89) | KCNH3 - potassium voltage-gated channel, subfamily h (eag-related), member 3 SLC4A10 - solute carrier family 4, sodium bicarbonate transporter, member 10 CHRNA7 - cholinergic receptor, nicotinic, alpha 7 (neuronal) SLC39A3 - solute carrier family 39 (zinc transporter), member 3 SLC6A7 - solute carrier family 6 (neurotransmitter transporter), member 7 ATP1A1 - atpase, na+/k+ transporting, alpha 1 polypeptide P2RX6 - purinergic receptor p2x, ligand-gated ion channel, 6 ADAMTS8 - adam metallopeptidase with thrombospondin type 1 motif, 8 SLC8A2 - solute carrier family 8 (sodium/calcium exchanger), member 2 ABCC8 - atp-binding cassette, sub-family c (cftr/mrp), member 8 PTK2B - protein tyrosine kinase 2 beta TRPM2 - transient receptor potential cation channel, subfamily m, member 2 KCNS1 - potassium voltage-gated channel, delayed-rectifier, subfamily s, member 1 ATP6V0D1 - atpase, h+ transporting, lysosomal 38kda, v0 subunit d1 KCNMB4 - potassium large conductance calcium-activated channel, subfamily m, beta member 4 KCNMA1 - potassium large conductance calcium-activated channel, subfamily m, alpha member 1 KCNH5 - potassium voltage-gated channel, subfamily h (eag-related), member 5 SLC36A1 - solute carrier family 36 (proton/amino acid symporter), member 1 KCNN1 - potassium intermediate/small conductance calcium-activated channel, subfamily n, member 1 KCNIP3 - kv channel interacting protein 3, calsenilin ASIC2 - acid-sensing (proton-gated) ion channel 2 SLC26A10 - solute carrier family 26, member 10 SLC37A4 - solute carrier family 37 (glucose-6-phosphate transporter), member 4 KCNT1 - potassium channel, subfamily t, member 1 CACNB4 - calcium channel, voltage-dependent, beta 4 subunit SLC25A11 - solute carrier family 25 (mitochondrial carrier; oxoglutarate carrier), member 11 SLC1A6 - solute carrier family 1 (high affinity aspartate/glutamate transporter), member 6 GABRA1 - gamma-aminobutyric acid (gaba) a receptor, alpha 1 SCN3B - sodium channel, voltage-gated, type iii, beta subunit HCN1 - hyperpolarization activated cyclic nucleotide-gated potassium channel 1 CACNA2D3 - calcium channel, voltage-dependent, alpha 2/delta subunit 3 SLC38A11 - solute carrier family 38, member 11 ATP6V1B1 - atpase, h+ transporting, lysosomal 56/58kda, v1 subunit b1 GABRA4 - gamma-aminobutyric acid (gaba) a receptor, alpha 4 ATP2B3 - atpase, ca++ transporting, plasma membrane 3 SLC17A7 - solute carrier family 17 (vesicular glutamate transporter), member 7 KCNH7 - potassium voltage-gated channel, subfamily h (eag-related), member 7 SHROOM2 - shroom family member 2 CACNG2 - calcium channel, voltage-dependent, gamma subunit 2 KCNQ5 - potassium voltage-gated channel, kqt-like subfamily, member 5 SLC25A38 - solute carrier family 25, member 38 CACNG3 - calcium channel, voltage-dependent, gamma subunit 3 MCOLN1 - mucolipin 1 KCNA2 - potassium voltage-gated channel, shaker-related subfamily, member 2 KCNA1 - potassium voltage-gated channel, shaker-related subfamily, member 1 (episodic ataxia with myokymia) LRRC8B - leucine rich repeat containing 8 family, member b SCN2B - sodium channel, voltage-gated, type ii, beta subunit SCN1B - sodium channel, voltage-gated, type i, beta subunit SLC45A4 - solute carrier family 45, member 4 SLC39A14 - solute carrier family 39 (zinc transporter), member 14 PKD2L1 - polycystic kidney disease 2-like 1 GABRG3 - gamma-aminobutyric acid (gaba) a receptor, gamma 3 PIDD - p53-induced death domain protein TRPV2 - transient receptor potential cation channel, subfamily v, member 2 SLC16A7 - solute carrier family 16 (monocarboxylate transporter), member 7 TFR2 - transferrin receptor 2 KCNV1 - potassium channel, subfamily v, member 1 KCNAB3 - potassium voltage-gated channel, shaker-related subfamily, beta member 3 ANO1 - anoctamin 1, calcium activated chloride channel GRIA4 - glutamate receptor, ionotropic, ampa 4 SLC38A7 - solute carrier family 38, member 7 ITPR1 - inositol 1,4,5-trisphosphate receptor, type 1 GRIN3A - glutamate receptor, ionotropic, n-methyl-d-aspartate 3a SLC26A4 - solute carrier family 26 (anion exchanger), member 4 CDK5 - cyclin-dependent kinase 5 GRIN2A - glutamate receptor, ionotropic, n-methyl d-aspartate 2a KCNJ12 - potassium inwardly-rectifying channel, subfamily j, member 12 KCNJ11 - potassium inwardly-rectifying channel, subfamily j, member 11 SLC45A1 - solute carrier family 45, member 1 KCNK1 - potassium channel, subfamily k, member 1 GRIK3 - glutamate receptor, ionotropic, kainate 3 GRIK4 - glutamate receptor, ionotropic, kainate 4 SLC30A3 - solute carrier family 30 (zinc transporter), member 3 KCNJ4 - potassium inwardly-rectifying channel, subfamily j, member 4 KCNJ3 - potassium inwardly-rectifying channel, subfamily j, member 3 GLRA3 - glycine receptor, alpha 3 TRPM4 - transient receptor potential cation channel, subfamily m, member 4 KCNJ9 - potassium inwardly-rectifying channel, subfamily j, member 9 KCNJ8 - potassium inwardly-rectifying channel, subfamily j, member 8 KCNJ6 - potassium inwardly-rectifying channel, subfamily j, member 6 KCNF1 - potassium voltage-gated channel, subfamily f, member 1 PDE2A - phosphodiesterase 2a, cgmp-stimulated KCNH1 - potassium voltage-gated channel, subfamily h (eag-related), member 1 KCNB1 - potassium voltage-gated channel, shab-related subfamily, member 1 SLC27A2 - solute carrier family 27 (fatty acid transporter), member 2 KCNC1 - potassium voltage-gated channel, shaw-related subfamily, member 1 KCNC2 - potassium voltage-gated channel, shaw-related subfamily, member 2 KCNK4 - potassium channel, subfamily k, member 4 KCNC3 - potassium voltage-gated channel, shaw-related subfamily, member 3 |
| GO:0016772 | transferase activity, transferring phosphorus-containing groups | 9.74E-9 | 1.91E-6 | 1.68 (16750,821,1655,136) | MTPAP - mitochondrial poly(a) polymerase FN3KRP - fructosamine 3 kinase related protein FLT3 - fms-related tyrosine kinase 3 BCKDK - branched chain ketoacid dehydrogenase kinase LIMK1 - lim domain kinase 1 MAP3K9 - mitogen-activated protein kinase kinase kinase 9 RET - ret proto-oncogene POLL - polymerase (dna directed), lambda CCNO - cyclin o CDKL1 - cyclin-dependent kinase-like 1 (cdc2-related kinase) UAP1 - udp-n-acteylglucosamine pyrophosphorylase 1 POLN - polymerase (dna directed) nu BRSK1 - br serine/threonine kinase 1 LRRK1 - leucine-rich repeat kinase 1 PIP5KL1 - phosphatidylinositol-4-phosphate 5-kinase-like 1 SPHK2 - sphingosine kinase 2 NLK - nemo-like kinase YRDC - yrdc domain containing (e. coli) MAGI3 - membrane associated guanylate kinase, ww and pdz domain containing 3 STK19 - serine/threonine kinase 19 NME6 - nme/nm23 nucleoside diphosphate kinase 6 TBRG4 - transforming growth factor beta regulator 4 NUAK1 - nuak family, snf1-like kinase, 1 PAK1 - p21 protein (cdc42/rac)-activated kinase 1 MAST1 - microtubule associated serine/threonine kinase 1 DGKZ - diacylglycerol kinase, zeta FGFRL1 - fibroblast growth factor receptor-like 1 CTU2 - cytosolic thiouridylase subunit 2 homolog (s. pombe) CCNA1 - cyclin a1 PDPK1 - 3-phosphoinositide dependent protein kinase-1 FES - feline sarcoma oncogene MAST3 - microtubule associated serine/threonine kinase 3 RPAP1 - rna polymerase ii associated protein 1 PIM3 - pim-3 oncogene DCLK1 - doublecortin-like kinase 1 EFNA3 - ephrin-a3 CDK5 - cyclin-dependent kinase 5 DAPK3 - death-associated protein kinase 3 CDK9 - cyclin-dependent kinase 9 PNCK - pregnancy up-regulated non-ubiquitously expressed cam kinase DGKA - diacylglycerol kinase, alpha 80kda MED20 - mediator complex subunit 20 MET - met proto-oncogene MAP3K13 - mitogen-activated protein kinase kinase kinase 13 MAPK12 - mitogen-activated protein kinase 12 TNNI3K - tnni3 interacting kinase GMPPA - gdp-mannose pyrophosphorylase a POLD2 - polymerase (dna directed), delta 2, accessory subunit GMPPB - gdp-mannose pyrophosphorylase b POLD1 - polymerase (dna directed), delta 1, catalytic subunit PGS1 - phosphatidylglycerophosphate synthase 1 POLR2H - polymerase (rna) ii (dna directed) polypeptide h POLR2E - polymerase (rna) ii (dna directed) polypeptide e, 25kda PAK6 - p21 protein (cdc42/rac)-activated kinase 6 PRKAA2 - protein kinase, amp-activated, alpha 2 catalytic subunit PRKAB1 - protein kinase, amp-activated, beta 1 non-catalytic subunit CAMK1D - calcium/calmodulin-dependent protein kinase id CDK14 - cyclin-dependent kinase 14 NME1 - nme/nm23 nucleoside diphosphate kinase 1 CAMK2G - calcium/calmodulin-dependent protein kinase ii gamma AGK - acylglycerol kinase ADRBK2 - adrenergic, beta, receptor kinase 2 ADRBK1 - adrenergic, beta, receptor kinase 1 STK32C - serine/threonine kinase 32c CAMK2A - calcium/calmodulin-dependent protein kinase ii alpha KHK - ketohexokinase (fructokinase) PTK2B - protein tyrosine kinase 2 beta DPAGT1 - dolichyl-phosphate (udp-n-acetylglucosamine) n-acetylglucosaminephosphotransferase 1 (glcnac-1-p transferase) CIT - citron (rho-interacting, serine/threonine kinase 21) CHKB - choline kinase beta CAD - carbamoyl-phosphate synthetase 2, aspartate transcarbamylase, and dihydroorotase PINK1 - pten induced putative kinase 1 HK1 - hexokinase 1 ADCK2 - aarf domain containing kinase 2 PIP5K1B - phosphatidylinositol-4-phosphate 5-kinase, type i, beta MATK - megakaryocyte-associated tyrosine kinase STYK1 - serine/threonine/tyrosine kinase 1 CAMKK1 - calcium/calmodulin-dependent protein kinase kinase 1, alpha PFKP - phosphofructokinase, platelet STK25 - serine/threonine kinase 25 STK11 - serine/threonine kinase 11 MAP3K6 - mitogen-activated protein kinase kinase kinase 6 GRK6 - g protein-coupled receptor kinase 6 ADCK5 - aarf domain containing kinase 5 PGM2L1 - phosphoglucomutase 2-like 1 PANK4 - pantothenate kinase 4 PAPSS2 - 3'-phosphoadenosine 5'-phosphosulfate synthase 2 GAK - cyclin g associated kinase PRPS2 - phosphoribosyl pyrophosphate synthetase 2 PRPS1 - phosphoribosyl pyrophosphate synthetase 1 CCNE1 - cyclin e1 TERF2 - telomeric repeat binding factor 2 PI4KA - phosphatidylinositol 4-kinase, catalytic, alpha CCNG2 - cyclin g2 TESK1 - testis-specific kinase 1 PRKY - protein kinase, y-linked, pseudogene CAMK1G - calcium/calmodulin-dependent protein kinase ig XYLB - xylulokinase homolog (h. influenzae) TPK1 - thiamin pyrophosphokinase 1 UCKL1 - uridine-cytidine kinase 1-like 1 TYRO3 - tyro3 protein tyrosine kinase FLAD1 - flavin adenine dinucleotide synthetase 1 BRSK2 - br serine/threonine kinase 2 TYK2 - tyrosine kinase 2 ITPKA - inositol-trisphosphate 3-kinase a LTK - leukocyte receptor tyrosine kinase MAP2K1 - mitogen-activated protein kinase kinase 1 CLK2 - cdc-like kinase 2 MAP2K2 - mitogen-activated protein kinase kinase 2 MAPK10 - mitogen-activated protein kinase 10 EPHA3 - eph receptor a3 MAPK11 - mitogen-activated protein kinase 11 PKDCC - protein kinase domain containing, cytoplasmic MAPK3 - mitogen-activated protein kinase 3 CCNI - cyclin i PRKG2 - protein kinase, cgmp-dependent, type ii EPHB3 - eph receptor b3 KALRN - kalirin, rhogef kinase EPHA5 - eph receptor a5 PHKG2 - phosphorylase kinase, gamma 2 (testis) EPHA4 - eph receptor a4 RPS6KA4 - ribosomal protein s6 kinase, 90kda, polypeptide 4 CSNK1E - casein kinase 1, epsilon PPIP5K1 - diphosphoinositol pentakisphosphate kinase 1 PIK3CD - phosphatidylinositol-4,5-bisphosphate 3-kinase, catalytic subunit delta CKMT1B - creatine kinase, mitochondrial 1b PIM1 - pim-1 oncogene EPHB6 - eph receptor b6 PRKCE - protein kinase c, epsilon PRKCB - protein kinase c, beta CCND2 - cyclin d2 ADCK1 - aarf domain containing kinase 1 PAK7 - p21 protein (cdc42/rac)-activated kinase 7 ERCC2 - excision repair cross-complementing rodent repair deficiency, complementation group 2 GSK3A - glycogen synthase kinase 3 alpha CDIPT - cdp-diacylglycerol--inositol 3-phosphatidyltransferase |
| GO:0016301 | kinase activity | 1.68E-8 | 3.14E-6 | 1.74 (16750,681,1655,117) | FN3KRP - fructosamine 3 kinase related protein FLT3 - fms-related tyrosine kinase 3 LIMK1 - lim domain kinase 1 BCKDK - branched chain ketoacid dehydrogenase kinase MAP3K9 - mitogen-activated protein kinase kinase kinase 9 RET - ret proto-oncogene CCNO - cyclin o CDKL1 - cyclin-dependent kinase-like 1 (cdc2-related kinase) BRSK1 - br serine/threonine kinase 1 LRRK1 - leucine-rich repeat kinase 1 PIP5KL1 - phosphatidylinositol-4-phosphate 5-kinase-like 1 NLK - nemo-like kinase SPHK2 - sphingosine kinase 2 MAGI3 - membrane associated guanylate kinase, ww and pdz domain containing 3 STK19 - serine/threonine kinase 19 NME6 - nme/nm23 nucleoside diphosphate kinase 6 TBRG4 - transforming growth factor beta regulator 4 NUAK1 - nuak family, snf1-like kinase, 1 PAK1 - p21 protein (cdc42/rac)-activated kinase 1 MAST1 - microtubule associated serine/threonine kinase 1 DGKZ - diacylglycerol kinase, zeta FGFRL1 - fibroblast growth factor receptor-like 1 CCNA1 - cyclin a1 PDPK1 - 3-phosphoinositide dependent protein kinase-1 FES - feline sarcoma oncogene MAST3 - microtubule associated serine/threonine kinase 3 PIM3 - pim-3 oncogene DCLK1 - doublecortin-like kinase 1 EFNA3 - ephrin-a3 CDK5 - cyclin-dependent kinase 5 DAPK3 - death-associated protein kinase 3 CDK9 - cyclin-dependent kinase 9 PNCK - pregnancy up-regulated non-ubiquitously expressed cam kinase DGKA - diacylglycerol kinase, alpha 80kda MET - met proto-oncogene MAP3K13 - mitogen-activated protein kinase kinase kinase 13 MAPK12 - mitogen-activated protein kinase 12 TNNI3K - tnni3 interacting kinase PAK6 - p21 protein (cdc42/rac)-activated kinase 6 PRKAA2 - protein kinase, amp-activated, alpha 2 catalytic subunit PRKAB1 - protein kinase, amp-activated, beta 1 non-catalytic subunit CAMK1D - calcium/calmodulin-dependent protein kinase id CDK14 - cyclin-dependent kinase 14 NME1 - nme/nm23 nucleoside diphosphate kinase 1 ADRBK2 - adrenergic, beta, receptor kinase 2 AGK - acylglycerol kinase CAMK2G - calcium/calmodulin-dependent protein kinase ii gamma ADRBK1 - adrenergic, beta, receptor kinase 1 STK32C - serine/threonine kinase 32c CAMK2A - calcium/calmodulin-dependent protein kinase ii alpha KHK - ketohexokinase (fructokinase) PTK2B - protein tyrosine kinase 2 beta CHKB - choline kinase beta CIT - citron (rho-interacting, serine/threonine kinase 21) CAD - carbamoyl-phosphate synthetase 2, aspartate transcarbamylase, and dihydroorotase PINK1 - pten induced putative kinase 1 HK1 - hexokinase 1 ADCK2 - aarf domain containing kinase 2 PIP5K1B - phosphatidylinositol-4-phosphate 5-kinase, type i, beta MATK - megakaryocyte-associated tyrosine kinase STYK1 - serine/threonine/tyrosine kinase 1 CAMKK1 - calcium/calmodulin-dependent protein kinase kinase 1, alpha PFKP - phosphofructokinase, platelet STK25 - serine/threonine kinase 25 STK11 - serine/threonine kinase 11 MAP3K6 - mitogen-activated protein kinase kinase kinase 6 GRK6 - g protein-coupled receptor kinase 6 ADCK5 - aarf domain containing kinase 5 PGM2L1 - phosphoglucomutase 2-like 1 PAPSS2 - 3'-phosphoadenosine 5'-phosphosulfate synthase 2 PANK4 - pantothenate kinase 4 PRPS2 - phosphoribosyl pyrophosphate synthetase 2 GAK - cyclin g associated kinase PRPS1 - phosphoribosyl pyrophosphate synthetase 1 CCNE1 - cyclin e1 PI4KA - phosphatidylinositol 4-kinase, catalytic, alpha CCNG2 - cyclin g2 TESK1 - testis-specific kinase 1 PRKY - protein kinase, y-linked, pseudogene CAMK1G - calcium/calmodulin-dependent protein kinase ig XYLB - xylulokinase homolog (h. influenzae) TPK1 - thiamin pyrophosphokinase 1 UCKL1 - uridine-cytidine kinase 1-like 1 TYRO3 - tyro3 protein tyrosine kinase BRSK2 - br serine/threonine kinase 2 ITPKA - inositol-trisphosphate 3-kinase a TYK2 - tyrosine kinase 2 CLK2 - cdc-like kinase 2 MAP2K1 - mitogen-activated protein kinase kinase 1 LTK - leukocyte receptor tyrosine kinase MAP2K2 - mitogen-activated protein kinase kinase 2 MAPK10 - mitogen-activated protein kinase 10 MAPK11 - mitogen-activated protein kinase 11 EPHA3 - eph receptor a3 PKDCC - protein kinase domain containing, cytoplasmic MAPK3 - mitogen-activated protein kinase 3 CCNI - cyclin i PRKG2 - protein kinase, cgmp-dependent, type ii KALRN - kalirin, rhogef kinase EPHB3 - eph receptor b3 PHKG2 - phosphorylase kinase, gamma 2 (testis) EPHA5 - eph receptor a5 EPHA4 - eph receptor a4 CSNK1E - casein kinase 1, epsilon RPS6KA4 - ribosomal protein s6 kinase, 90kda, polypeptide 4 PPIP5K1 - diphosphoinositol pentakisphosphate kinase 1 CKMT1B - creatine kinase, mitochondrial 1b PIK3CD - phosphatidylinositol-4,5-bisphosphate 3-kinase, catalytic subunit delta PIM1 - pim-1 oncogene PRKCE - protein kinase c, epsilon EPHB6 - eph receptor b6 PRKCB - protein kinase c, beta CCND2 - cyclin d2 ADCK1 - aarf domain containing kinase 1 PAK7 - p21 protein (cdc42/rac)-activated kinase 7 ERCC2 - excision repair cross-complementing rodent repair deficiency, complementation group 2 GSK3A - glycogen synthase kinase 3 alpha |
| GO:0043168 | anion binding | 6.86E-8 | 1.24E-5 | 1.33 (16750,2495,1722,341) | TRIB3 - tribbles homolog 3 (drosophila) RFC2 - replication factor c (activator 1) 2, 40kda AGAP3 - arfgap with gtpase domain, ankyrin repeat and ph domain 3 LIMK1 - lim domain kinase 1 BCKDK - branched chain ketoacid dehydrogenase kinase DHX30 - deah (asp-glu-ala-his) box helicase 30 SEPT5 - septin 5 ABCA4 - atp-binding cassette, sub-family a (abc1), member 4 SPG7 - spastic paraplegia 7 (pure and complicated autosomal recessive) HRG - histidine-rich glycoprotein TRPM2 - transient receptor potential cation channel, subfamily m, member 2 RET - ret proto-oncogene GCKR - glucokinase (hexokinase 4) regulator CDKL1 - cyclin-dependent kinase-like 1 (cdc2-related kinase) TUBA4A - tubulin, alpha 4a TUBA3C - tubulin, alpha 3c RAB3D - rab3d, member ras oncogene family BRSK1 - br serine/threonine kinase 1 CCT5 - chaperonin containing tcp1, subunit 5 (epsilon) PIP5KL1 - phosphatidylinositol-4-phosphate 5-kinase-like 1 SPHK2 - sphingosine kinase 2 UBE2J2 - ubiquitin-conjugating enzyme e2, j2 RAB37 - rab37, member ras oncogene family LIPC - lipase, hepatic MAGI3 - membrane associated guanylate kinase, ww and pdz domain containing 3 STK19 - serine/threonine kinase 19 TUBA4B - tubulin, alpha 4b (pseudogene) RAB40C - rab40c, member ras oncogene family SYTL2 - synaptotagmin-like 2 NME6 - nme/nm23 nucleoside diphosphate kinase 6 RIMKLA - ribosomal modification protein rimk-like family member a MYO5B - myosin vb MAST1 - microtubule associated serine/threonine kinase 1 EEF1A2 - eukaryotic translation elongation factor 1 alpha 2 GLDC - glycine dehydrogenase (decarboxylating) MAST3 - microtubule associated serine/threonine kinase 3 ADCY6 - adenylate cyclase 6 MYO16 - myosin xvi PIM3 - pim-3 oncogene UNC13A - unc-13 homolog a (c. elegans) GRIN3A - glutamate receptor, ionotropic, n-methyl-d-aspartate 3a ADCY2 - adenylate cyclase 2 (brain) SNRNP200 - small nuclear ribonucleoprotein 200kda (u5) DIRAS2 - diras family, gtp-binding ras-like 2 FIGNL2 - fidgetin-like 2 PNCK - pregnancy up-regulated non-ubiquitously expressed cam kinase NRBP1 - nuclear receptor binding protein 1 KIF21B - kinesin family member 21b MVD - mevalonate (diphospho) decarboxylase TRPM4 - transient receptor potential cation channel, subfamily m, member 4 CSAD - cysteine sulfinic acid decarboxylase LCN1 - lipocalin 1 GNA15 - guanine nucleotide binding protein (g protein), alpha 15 (gq class) GMPPB - gdp-mannose pyrophosphorylase b GNA11 - guanine nucleotide binding protein (g protein), alpha 11 (gq class) ARL4A - adp-ribosylation factor-like 4a ARL4C - adp-ribosylation factor-like 4c PGS1 - phosphatidylglycerophosphate synthase 1 PAK6 - p21 protein (cdc42/rac)-activated kinase 6 BAIAP3 - bai1-associated protein 3 TRAP1 - tnf receptor-associated protein 1 RUVBL2 - ruvb-like 2 (e. coli) COL13A1 - collagen, type xiii, alpha 1 PRKAA2 - protein kinase, amp-activated, alpha 2 catalytic subunit MYO15A - myosin xva NAT6 - n-acetyltransferase 6 (gcn5-related) FABP3 - fatty acid binding protein 3, muscle and heart (mammary-derived growth inhibitor) CAMK1D - calcium/calmodulin-dependent protein kinase id KIF3C - kinesin family member 3c CAMK2G - calcium/calmodulin-dependent protein kinase ii gamma AGK - acylglycerol kinase ADRBK2 - adrenergic, beta, receptor kinase 2 SYT1 - synaptotagmin i PLEKHB2 - pleckstrin homology domain containing, family b (evectins) member 2 ADRBK1 - adrenergic, beta, receptor kinase 1 ADAMTS8 - adam metallopeptidase with thrombospondin type 1 motif, 8 KIF5A - kinesin family member 5a CHRD - chordin CAMK2A - calcium/calmodulin-dependent protein kinase ii alpha PICK1 - protein interacting with prkca 1 ABCC8 - atp-binding cassette, sub-family c (cftr/mrp), member 8 KHK - ketohexokinase (fructokinase) TIMM44 - translocase of inner mitochondrial membrane 44 homolog (yeast) PTK2B - protein tyrosine kinase 2 beta CIT - citron (rho-interacting, serine/threonine kinase 21) PPP5C - protein phosphatase 5, catalytic subunit CUL9 - cullin 9 CARS - cysteinyl-trna synthetase FARSA - phenylalanyl-trna synthetase, alpha subunit AACS - acetoacetyl-coa synthetase FASN - fatty acid synthase CAD - carbamoyl-phosphate synthetase 2, aspartate transcarbamylase, and dihydroorotase PINK1 - pten induced putative kinase 1 HK1 - hexokinase 1 XRCC6 - x-ray repair complementing defective repair in chinese hamster cells 6 UBE2E3 - ubiquitin-conjugating enzyme e2e 3 ADCK2 - aarf domain containing kinase 2 KIF25 - kinesin family member 25 KIFC2 - kinesin family member c2 DDX51 - dead (asp-glu-ala-asp) box polypeptide 51 CAMKK1 - calcium/calmodulin-dependent protein kinase kinase 1, alpha VARS - valyl-trna synthetase STK25 - serine/threonine kinase 25 RAPGEF4 - rap guanine nucleotide exchange factor (gef) 4 ALAS1 - aminolevulinate, delta-, synthase 1 STK11 - serine/threonine kinase 11 RABL6 - rab, member ras oncogene family-like 6 TTLL12 - tubulin tyrosine ligase-like family, member 12 LONP1 - lon peptidase 1, mitochondrial EEFSEC - eukaryotic elongation factor, selenocysteine-trna-specific GAK - cyclin g associated kinase TUBA1B - tubulin, alpha 1b PRPS2 - phosphoribosyl pyrophosphate synthetase 2 PRPS1 - phosphoribosyl pyrophosphate synthetase 1 PRODH - proline dehydrogenase (oxidase) 1 RAB26 - rab26, member ras oncogene family SLIT2 - slit homolog 2 (drosophila) GPAA1 - glycosylphosphatidylinositol anchor attachment 1 GBF1 - golgi brefeldin a resistant guanine nucleotide exchange factor 1 PRKY - protein kinase, y-linked, pseudogene TECPR1 - tectonin beta-propeller repeat containing 1 CAMK1G - calcium/calmodulin-dependent protein kinase ig XYLB - xylulokinase homolog (h. influenzae) ACAD9 - acyl-coa dehydrogenase family, member 9 UCKL1 - uridine-cytidine kinase 1-like 1 TYRO3 - tyro3 protein tyrosine kinase ITPKA - inositol-trisphosphate 3-kinase a TYK2 - tyrosine kinase 2 RSPO2 - r-spondin 2 MAP2K1 - mitogen-activated protein kinase kinase 1 DHX35 - deah (asp-glu-ala-his) box polypeptide 35 ITPR1 - inositol 1,4,5-trisphosphate receptor, type 1 MAP2K2 - mitogen-activated protein kinase kinase 2 GAD1 - glutamate decarboxylase 1 (brain, 67kda) MAPK10 - mitogen-activated protein kinase 10 MAPK11 - mitogen-activated protein kinase 11 EPHA3 - eph receptor a3 KCNJ11 - potassium inwardly-rectifying channel, subfamily j, member 11 PKDCC - protein kinase domain containing, cytoplasmic MAPK3 - mitogen-activated protein kinase 3 PRKG2 - protein kinase, cgmp-dependent, type ii GLRA3 - glycine receptor, alpha 3 OSBPL10 - oxysterol binding protein-like 10 AMPH - amphiphysin EPHB3 - eph receptor b3 EPHA5 - eph receptor a5 EPHA4 - eph receptor a4 KCNJ8 - potassium inwardly-rectifying channel, subfamily j, member 8 OSBPL5 - oxysterol binding protein-like 5 CSNK1E - casein kinase 1, epsilon ATP13A1 - atpase type 13a1 PRKCE - protein kinase c, epsilon EPHB6 - eph receptor b6 PRKCB - protein kinase c, beta KCNH1 - potassium voltage-gated channel, subfamily h (eag-related), member 1 GAP43 - growth associated protein 43 SLC27A2 - solute carrier family 27 (fatty acid transporter), member 2 ADCK1 - aarf domain containing kinase 1 UBE2O - ubiquitin-conjugating enzyme e2o PAK7 - p21 protein (cdc42/rac)-activated kinase 7 ERCC2 - excision repair cross-complementing rodent repair deficiency, complementation group 2 SYT12 - synaptotagmin xii CDC6 - cell division cycle 6 MTPAP - mitochondrial poly(a) polymerase DHX8 - deah (asp-glu-ala-his) box polypeptide 8 FLT3 - fms-related tyrosine kinase 3 TUBA3D - tubulin, alpha 3d SYT17 - synaptotagmin xvii CPNE9 - copine family member ix GSDMB - gasdermin b MAP3K9 - mitogen-activated protein kinase kinase kinase 9 ANXA6 - annexin a6 PSD - pleckstrin and sec7 domain containing CYTH3 - cytohesin 3 PLCB1 - phospholipase c, beta 1 (phosphoinositide-specific) STARD5 - star-related lipid transfer (start) domain containing 5 TUBA8 - tubulin, alpha 8 RAB15 - rab15, member ras oncogene family FRMPD2 - ferm and pdz domain containing 2 PCLO - piccolo presynaptic cytomatrix protein DFNA5 - deafness, autosomal dominant 5 MTHFD1L - methylenetetrahydrofolate dehydrogenase (nadp+ dependent) 1-like LRRK1 - leucine-rich repeat kinase 1 NLK - nemo-like kinase UBE2QL1 - ubiquitin-conjugating enzyme e2q family-like 1 MAGI1 - membrane associated guanylate kinase, ww and pdz domain containing 1 NUAK1 - nuak family, snf1-like kinase, 1 PAK1 - p21 protein (cdc42/rac)-activated kinase 1 RASL10A - ras-like, family 10, member a POSTN - periostin, osteoblast specific factor DGKZ - diacylglycerol kinase, zeta NISCH - nischarin FGFRL1 - fibroblast growth factor receptor-like 1 LEPREL2 - leprecan-like 2 ESYT1 - extended synaptotagmin-like protein 1 TUBA1A - tubulin, alpha 1a FGF9 - fibroblast growth factor 9 ABCB8 - atp-binding cassette, sub-family b (mdr/tap), member 8 CHD5 - chromodomain helicase dna binding protein 5 PDPK1 - 3-phosphoinositide dependent protein kinase-1 RAB24 - rab24, member ras oncogene family FES - feline sarcoma oncogene SNX17 - sorting nexin 17 DHX38 - deah (asp-glu-ala-his) box polypeptide 38 DCLK1 - doublecortin-like kinase 1 CPNE8 - copine viii SCN8A - sodium channel, voltage gated, type viii, alpha subunit KMO - kynurenine 3-monooxygenase (kynurenine 3-hydroxylase) HIST1H1A - histone cluster 1, h1a CDK5 - cyclin-dependent kinase 5 ASNA1 - arsa arsenite transporter, atp-binding, homolog 1 (bacterial) DAPK3 - death-associated protein kinase 3 RND1 - rho family gtpase 1 CDK9 - cyclin-dependent kinase 9 DBH - dopamine beta-hydroxylase (dopamine beta-monooxygenase) DGKA - diacylglycerol kinase, alpha 80kda MET - met proto-oncogene MAP3K13 - mitogen-activated protein kinase kinase kinase 13 ARL9 - adp-ribosylation factor-like 9 ASS1 - argininosuccinate synthase 1 MFGE8 - milk fat globule-egf factor 8 protein MAPK12 - mitogen-activated protein kinase 12 TNNI3K - tnni3 interacting kinase SARS - seryl-trna synthetase PDE2A - phosphodiesterase 2a, cgmp-stimulated NRGN - neurogranin (protein kinase c substrate, rc3) ARHGAP9 - rho gtpase activating protein 9 TUBB8 - tubulin, beta 8 class viii PDE4A - phosphodiesterase 4a, camp-specific CYP26A1 - cytochrome p450, family 26, subfamily a, polypeptide 1 PTPRF - protein tyrosine phosphatase, receptor type, f GPN2 - gpn-loop gtpase 2 GTPBP2 - gtp binding protein 2 RTBDN - retbindin RHEBL1 - ras homolog enriched in brain like 1 NOS2 - nitric oxide synthase 2, inducible GNL1 - guanine nucleotide binding protein-like 1 P4HTM - prolyl 4-hydroxylase, transmembrane (endoplasmic reticulum) ATP12A - atpase, h+/k+ transporting, nongastric, alpha polypeptide P2RX6 - purinergic receptor p2x, ligand-gated ion channel, 6 ATP1A1 - atpase, na+/k+ transporting, alpha 1 polypeptide CDK14 - cyclin-dependent kinase 14 NME1 - nme/nm23 nucleoside diphosphate kinase 1 RPH3A - rabphilin 3a homolog (mouse) GOT1 - glutamic-oxaloacetic transaminase 1, soluble ATP1A4 - atpase, na+/k+ transporting, alpha 4 polypeptide SDCBP2 - syndecan binding protein (syntenin) 2 STK32C - serine/threonine kinase 32c NOV - nephroblastoma overexpressed RAB6B - rab6b, member ras oncogene family MORC2 - morc family cw-type zinc finger 2 MAPT - microtubule-associated protein tau MYO19 - myosin xix SIRT6 - sirtuin 6 SIRT7 - sirtuin 7 PACSIN1 - protein kinase c and casein kinase substrate in neurons 1 CHKB - choline kinase beta SIRT4 - sirtuin 4 CNGB3 - cyclic nucleotide gated channel beta 3 MAP4K2 - mitogen-activated protein kinase kinase kinase kinase 2 ABCB9 - atp-binding cassette, sub-family b (mdr/tap), member 9 CPNE4 - copine iv HAPLN4 - hyaluronan and proteoglycan link protein 4 ARL10 - adp-ribosylation factor-like 10 RAB3A - rab3a, member ras oncogene family SKIV2L - superkiller viralicidic activity 2-like (s. cerevisiae) HCN1 - hyperpolarization activated cyclic nucleotide-gated potassium channel 1 ATP6V1B1 - atpase, h+ transporting, lysosomal 56/58kda, v1 subunit b1 FRMPD4 - ferm and pdz domain containing 4 PIP5K1B - phosphatidylinositol-4-phosphate 5-kinase, type i, beta SYT13 - synaptotagmin xiii PTGES2 - prostaglandin e synthase 2 MATK - megakaryocyte-associated tyrosine kinase STYK1 - serine/threonine/tyrosine kinase 1 PIF1 - pif1 5'-to-3' dna helicase TUBG2 - tubulin, gamma 2 BAIAP2L2 - bai1-associated protein 2-like 2 STOML2 - stomatin (epb72)-like 2 PFKP - phosphofructokinase, platelet RAC2 - ras-related c3 botulinum toxin substrate 2 (rho family, small gtp binding protein rac2) ATP2B4 - atpase, ca++ transporting, plasma membrane 4 PFAS - phosphoribosylformylglycinamidine synthase ATP2B3 - atpase, ca++ transporting, plasma membrane 3 EHD2 - eh-domain containing 2 MAP3K6 - mitogen-activated protein kinase kinase kinase 6 INO80 - ino80 complex subunit PITPNM2 - phosphatidylinositol transfer protein, membrane-associated 2 EHD3 - eh-domain containing 3 DDX56 - dead (asp-glu-ala-asp) box helicase 56 RAB27B - rab27b, member ras oncogene family ATP8B1 - atpase, aminophospholipid transporter, class i, type 8b, member 1 GRK6 - g protein-coupled receptor kinase 6 COL5A3 - collagen, type v, alpha 3 PAPSS2 - 3'-phosphoadenosine 5'-phosphosulfate synthase 2 PANK4 - pantothenate kinase 4 EGLN2 - egl-9 family hypoxia-inducible factor 2 RASAL1 - ras protein activator like 1 (gap1 like) BAD - bcl2-associated agonist of cell death MICAL2 - microtubule associated monooxygenase, calponin and lim domain containing 2 PI4KA - phosphatidylinositol 4-kinase, catalytic, alpha CYP26B1 - cytochrome p450, family 26, subfamily b, polypeptide 1 TESK1 - testis-specific kinase 1 TPK1 - thiamin pyrophosphokinase 1 PLA2G4A - phospholipase a2, group iva (cytosolic, calcium-dependent) MYH7B - myosin, heavy chain 7b, cardiac muscle, beta RASD1 - ras, dexamethasone-induced 1 SESN2 - sestrin 2 KIF1A - kinesin family member 1a FLAD1 - flavin adenine dinucleotide synthetase 1 BRSK2 - br serine/threonine kinase 2 LTK - leukocyte receptor tyrosine kinase FBLN7 - fibulin 7 CLK2 - cdc-like kinase 2 RHOV - ras homolog family member v TWF2 - twinfilin actin-binding protein 2 ACAD8 - acyl-coa dehydrogenase family, member 8 ABCC10 - atp-binding cassette, sub-family c (cftr/mrp), member 10 CLCN7 - chloride channel, voltage-sensitive 7 RTN4RL1 - reticulon 4 receptor-like 1 ACOT7 - acyl-coa thioesterase 7 KALRN - kalirin, rhogef kinase ATAD3B - atpase family, aaa domain containing 3b PHKG2 - phosphorylase kinase, gamma 2 (testis) UNC13C - unc-13 homolog c (c. elegans) RPS6KA4 - ribosomal protein s6 kinase, 90kda, polypeptide 4 DHX16 - deah (asp-glu-ala-his) box polypeptide 16 PPIP5K1 - diphosphoinositol pentakisphosphate kinase 1 DOC2A - double c2-like domains, alpha PIK3CD - phosphatidylinositol-4,5-bisphosphate 3-kinase, catalytic subunit delta CKMT1B - creatine kinase, mitochondrial 1b PIM1 - pim-1 oncogene SYT16 - synaptotagmin xvi THBS3 - thrombospondin 3 ESYT3 - extended synaptotagmin-like protein 3 DNM1 - dynamin 1 UBE2D4 - ubiquitin-conjugating enzyme e2d 4 (putative) TARS2 - threonyl-trna synthetase 2, mitochondrial (putative) GSK3A - glycogen synthase kinase 3 alpha NAV2 - neuron navigator 2 P4HA2 - prolyl 4-hydroxylase, alpha polypeptide ii RHOF - ras homolog family member f (in filopodia) |
| GO:0099094 | ligand-gated cation channel activity | 7.83E-8 | 1.36E-5 | 4.29 (16750,93,924,22) | ASIC2 - acid-sensing (proton-gated) ion channel 2 MCOLN1 - mucolipin 1 KCNJ12 - potassium inwardly-rectifying channel, subfamily j, member 12 GRIN2A - glutamate receptor, ionotropic, n-methyl d-aspartate 2a KCNJ11 - potassium inwardly-rectifying channel, subfamily j, member 11 CHRNA7 - cholinergic receptor, nicotinic, alpha 7 (neuronal) KCNK1 - potassium channel, subfamily k, member 1 GRIK3 - glutamate receptor, ionotropic, kainate 3 GRIK4 - glutamate receptor, ionotropic, kainate 4 KCNJ4 - potassium inwardly-rectifying channel, subfamily j, member 4 KCNJ3 - potassium inwardly-rectifying channel, subfamily j, member 3 P2RX6 - purinergic receptor p2x, ligand-gated ion channel, 6 HCN1 - hyperpolarization activated cyclic nucleotide-gated potassium channel 1 KCNJ9 - potassium inwardly-rectifying channel, subfamily j, member 9 KCNJ8 - potassium inwardly-rectifying channel, subfamily j, member 8 KCNJ6 - potassium inwardly-rectifying channel, subfamily j, member 6 PTK2B - protein tyrosine kinase 2 beta TRPM2 - transient receptor potential cation channel, subfamily m, member 2 GRIA4 - glutamate receptor, ionotropic, ampa 4 SHROOM2 - shroom family member 2 ITPR1 - inositol 1,4,5-trisphosphate receptor, type 1 GRIN3A - glutamate receptor, ionotropic, n-methyl-d-aspartate 3a |
| GO:0016773 | phosphotransferase activity, alcohol group as acceptor | 8.61E-8 | 1.44E-5 | 1.74 (16750,624,1655,107) | FLT3 - fms-related tyrosine kinase 3 LIMK1 - lim domain kinase 1 BCKDK - branched chain ketoacid dehydrogenase kinase MAP3K9 - mitogen-activated protein kinase kinase kinase 9 RET - ret proto-oncogene CCNO - cyclin o CDKL1 - cyclin-dependent kinase-like 1 (cdc2-related kinase) BRSK1 - br serine/threonine kinase 1 LRRK1 - leucine-rich repeat kinase 1 PIP5KL1 - phosphatidylinositol-4-phosphate 5-kinase-like 1 NLK - nemo-like kinase SPHK2 - sphingosine kinase 2 STK19 - serine/threonine kinase 19 TBRG4 - transforming growth factor beta regulator 4 NUAK1 - nuak family, snf1-like kinase, 1 PAK1 - p21 protein (cdc42/rac)-activated kinase 1 MAST1 - microtubule associated serine/threonine kinase 1 DGKZ - diacylglycerol kinase, zeta FGFRL1 - fibroblast growth factor receptor-like 1 CCNA1 - cyclin a1 PDPK1 - 3-phosphoinositide dependent protein kinase-1 FES - feline sarcoma oncogene MAST3 - microtubule associated serine/threonine kinase 3 PIM3 - pim-3 oncogene DCLK1 - doublecortin-like kinase 1 EFNA3 - ephrin-a3 CDK5 - cyclin-dependent kinase 5 DAPK3 - death-associated protein kinase 3 CDK9 - cyclin-dependent kinase 9 PNCK - pregnancy up-regulated non-ubiquitously expressed cam kinase DGKA - diacylglycerol kinase, alpha 80kda MET - met proto-oncogene MAP3K13 - mitogen-activated protein kinase kinase kinase 13 MAPK12 - mitogen-activated protein kinase 12 TNNI3K - tnni3 interacting kinase PAK6 - p21 protein (cdc42/rac)-activated kinase 6 PRKAA2 - protein kinase, amp-activated, alpha 2 catalytic subunit PRKAB1 - protein kinase, amp-activated, beta 1 non-catalytic subunit CAMK1D - calcium/calmodulin-dependent protein kinase id CDK14 - cyclin-dependent kinase 14 ADRBK2 - adrenergic, beta, receptor kinase 2 AGK - acylglycerol kinase CAMK2G - calcium/calmodulin-dependent protein kinase ii gamma ADRBK1 - adrenergic, beta, receptor kinase 1 STK32C - serine/threonine kinase 32c CAMK2A - calcium/calmodulin-dependent protein kinase ii alpha KHK - ketohexokinase (fructokinase) PTK2B - protein tyrosine kinase 2 beta CHKB - choline kinase beta CIT - citron (rho-interacting, serine/threonine kinase 21) CAD - carbamoyl-phosphate synthetase 2, aspartate transcarbamylase, and dihydroorotase PINK1 - pten induced putative kinase 1 HK1 - hexokinase 1 ADCK2 - aarf domain containing kinase 2 PIP5K1B - phosphatidylinositol-4-phosphate 5-kinase, type i, beta MATK - megakaryocyte-associated tyrosine kinase STYK1 - serine/threonine/tyrosine kinase 1 CAMKK1 - calcium/calmodulin-dependent protein kinase kinase 1, alpha PFKP - phosphofructokinase, platelet STK25 - serine/threonine kinase 25 STK11 - serine/threonine kinase 11 MAP3K6 - mitogen-activated protein kinase kinase kinase 6 GRK6 - g protein-coupled receptor kinase 6 ADCK5 - aarf domain containing kinase 5 PGM2L1 - phosphoglucomutase 2-like 1 PANK4 - pantothenate kinase 4 PAPSS2 - 3'-phosphoadenosine 5'-phosphosulfate synthase 2 GAK - cyclin g associated kinase CCNE1 - cyclin e1 PI4KA - phosphatidylinositol 4-kinase, catalytic, alpha CCNG2 - cyclin g2 TESK1 - testis-specific kinase 1 PRKY - protein kinase, y-linked, pseudogene CAMK1G - calcium/calmodulin-dependent protein kinase ig XYLB - xylulokinase homolog (h. influenzae) TYRO3 - tyro3 protein tyrosine kinase BRSK2 - br serine/threonine kinase 2 ITPKA - inositol-trisphosphate 3-kinase a TYK2 - tyrosine kinase 2 CLK2 - cdc-like kinase 2 MAP2K1 - mitogen-activated protein kinase kinase 1 LTK - leukocyte receptor tyrosine kinase MAP2K2 - mitogen-activated protein kinase kinase 2 MAPK10 - mitogen-activated protein kinase 10 MAPK11 - mitogen-activated protein kinase 11 EPHA3 - eph receptor a3 PKDCC - protein kinase domain containing, cytoplasmic MAPK3 - mitogen-activated protein kinase 3 CCNI - cyclin i PRKG2 - protein kinase, cgmp-dependent, type ii KALRN - kalirin, rhogef kinase EPHB3 - eph receptor b3 PHKG2 - phosphorylase kinase, gamma 2 (testis) EPHA5 - eph receptor a5 EPHA4 - eph receptor a4 CSNK1E - casein kinase 1, epsilon RPS6KA4 - ribosomal protein s6 kinase, 90kda, polypeptide 4 PIK3CD - phosphatidylinositol-4,5-bisphosphate 3-kinase, catalytic subunit delta PIM1 - pim-1 oncogene PRKCE - protein kinase c, epsilon EPHB6 - eph receptor b6 PRKCB - protein kinase c, beta CCND2 - cyclin d2 ADCK1 - aarf domain containing kinase 1 GSK3A - glycogen synthase kinase 3 alpha ERCC2 - excision repair cross-complementing rodent repair deficiency, complementation group 2 PAK7 - p21 protein (cdc42/rac)-activated kinase 7 |
| GO:0005215 | transporter activity | 1.05E-7 | 1.69E-5 | 1.56 (16750,1123,1513,158) | SLC4A10 - solute carrier family 4, sodium bicarbonate transporter, member 10 SLC22A18 - solute carrier family 22, member 18 SLC22A10 - solute carrier family 22, member 10 SLC29A1 - solute carrier family 29 (equilibrative nucleoside transporter), member 1 ABCA4 - atp-binding cassette, sub-family a (abc1), member 4 TRPM2 - transient receptor potential cation channel, subfamily m, member 2 ADC - arginine decarboxylase KCNMB4 - potassium large conductance calcium-activated channel, subfamily m, beta member 4 STARD5 - star-related lipid transfer (start) domain containing 5 ASIC2 - acid-sensing (proton-gated) ion channel 2 SV2B - synaptic vesicle glycoprotein 2b SLC46A2 - solute carrier family 46, member 2 OCA2 - oculocutaneous albinism ii SLCO4A1 - solute carrier organic anion transporter family, member 4a1 STEAP1 - six transmembrane epithelial antigen of the prostate 1 SCN3B - sodium channel, voltage-gated, type iii, beta subunit STEAP2 - steap family member 2, metalloreductase CACNA2D3 - calcium channel, voltage-dependent, alpha 2/delta subunit 3 SLC38A11 - solute carrier family 38, member 11 SLC35C2 - solute carrier family 35 (gdp-fucose transporter), member c2 KCNH7 - potassium voltage-gated channel, subfamily h (eag-related), member 7 SHROOM2 - shroom family member 2 SCN1A - sodium channel, voltage-gated, type i, alpha subunit C14orf79 - chromosome 14 open reading frame 79 SCN2B - sodium channel, voltage-gated, type ii, beta subunit SCN1B - sodium channel, voltage-gated, type i, beta subunit ABCB8 - atp-binding cassette, sub-family b (mdr/tap), member 8 PIDD - p53-induced death domain protein OSBPL3 - oxysterol binding protein-like 3 TRPV2 - transient receptor potential cation channel, subfamily v, member 2 MFSD4 - major facilitator superfamily domain containing 4 SLC16A7 - solute carrier family 16 (monocarboxylate transporter), member 7 KCNAB3 - potassium voltage-gated channel, shaker-related subfamily, beta member 3 ANO1 - anoctamin 1, calcium activated chloride channel GRIN3A - glutamate receptor, ionotropic, n-methyl-d-aspartate 3a SLC26A4 - solute carrier family 26 (anion exchanger), member 4 TIMM17B - translocase of inner mitochondrial membrane 17 homolog b (yeast) ASNA1 - arsa arsenite transporter, atp-binding, homolog 1 (bacterial) CDK5 - cyclin-dependent kinase 5 SVOP - sv2 related protein homolog (rat) SLC2A11 - solute carrier family 2 (facilitated glucose transporter), member 11 SLC30A3 - solute carrier family 30 (zinc transporter), member 3 TRPM4 - transient receptor potential cation channel, subfamily m, member 4 CACNA1G - calcium channel, voltage-dependent, t type, alpha 1g subunit SLC2A13 - solute carrier family 2 (facilitated glucose transporter), member 13 PDE2A - phosphodiesterase 2a, cgmp-stimulated RAMP3 - receptor (g protein-coupled) activity modifying protein 3 CPLX3 - complexin 3 TIMM22 - translocase of inner mitochondrial membrane 22 homolog (yeast) SLC4A8 - solute carrier family 4, sodium bicarbonate cotransporter, member 8 SLC4A7 - solute carrier family 4, sodium bicarbonate cotransporter, member 7 KCNH3 - potassium voltage-gated channel, subfamily h (eag-related), member 3 SLC25A26 - solute carrier family 25 (s-adenosylmethionine carrier), member 26 AP1B1 - adaptor-related protein complex 1, beta 1 subunit SLC39A3 - solute carrier family 39 (zinc transporter), member 3 CHRNA7 - cholinergic receptor, nicotinic, alpha 7 (neuronal) SLC6A7 - solute carrier family 6 (neurotransmitter transporter), member 7 RTBDN - retbindin KCNMB3 - potassium large conductance calcium-activated channel, subfamily m beta member 3 FABP3 - fatty acid binding protein 3, muscle and heart (mammary-derived growth inhibitor) ATP12A - atpase, h+/k+ transporting, nongastric, alpha polypeptide P2RX6 - purinergic receptor p2x, ligand-gated ion channel, 6 ATP1A1 - atpase, na+/k+ transporting, alpha 1 polypeptide TIMM17A - translocase of inner mitochondrial membrane 17 homolog a (yeast) SLC8A2 - solute carrier family 8 (sodium/calcium exchanger), member 2 ADAMTS8 - adam metallopeptidase with thrombospondin type 1 motif, 8 SLC6A17 - solute carrier family 6 (neutral amino acid transporter), member 17 ABCC8 - atp-binding cassette, sub-family c (cftr/mrp), member 8 CACNG7 - calcium channel, voltage-dependent, gamma subunit 7 SLC9A5 - solute carrier family 9, subfamily a (nhe5, cation proton antiporter 5), member 5 SLC35F1 - solute carrier family 35, member f1 PTK2B - protein tyrosine kinase 2 beta ATP6V0D1 - atpase, h+ transporting, lysosomal 38kda, v0 subunit d1 KCNS1 - potassium voltage-gated channel, delayed-rectifier, subfamily s, member 1 KCNN2 - potassium intermediate/small conductance calcium-activated channel, subfamily n, member 2 KCNK3 - potassium channel, subfamily k, member 3 KCNMA1 - potassium large conductance calcium-activated channel, subfamily m, alpha member 1 SLC36A1 - solute carrier family 36 (proton/amino acid symporter), member 1 SLC6A20 - solute carrier family 6 (proline imino transporter), member 20 KCNH5 - potassium voltage-gated channel, subfamily h (eag-related), member 5 KCNN1 - potassium intermediate/small conductance calcium-activated channel, subfamily n, member 1 KCNIP3 - kv channel interacting protein 3, calsenilin SLC26A10 - solute carrier family 26, member 10 KCNT1 - potassium channel, subfamily t, member 1 SLC37A4 - solute carrier family 37 (glucose-6-phosphate transporter), member 4 CACNB4 - calcium channel, voltage-dependent, beta 4 subunit SLC26A6 - solute carrier family 26 (anion exchanger), member 6 SLC29A4 - solute carrier family 29 (equilibrative nucleoside transporter), member 4 SLC25A11 - solute carrier family 25 (mitochondrial carrier; oxoglutarate carrier), member 11 SLC1A6 - solute carrier family 1 (high affinity aspartate/glutamate transporter), member 6 RHCG - rh family, c glycoprotein GABRA1 - gamma-aminobutyric acid (gaba) a receptor, alpha 1 HCN1 - hyperpolarization activated cyclic nucleotide-gated potassium channel 1 ATP6V1B1 - atpase, h+ transporting, lysosomal 56/58kda, v1 subunit b1 GABRA4 - gamma-aminobutyric acid (gaba) a receptor, alpha 4 GABRA3 - gamma-aminobutyric acid (gaba) a receptor, alpha 3 ATP2B3 - atpase, ca++ transporting, plasma membrane 3 SLC17A7 - solute carrier family 17 (vesicular glutamate transporter), member 7 PITPNM2 - phosphatidylinositol transfer protein, membrane-associated 2 SLC25A19 - solute carrier family 25 (mitochondrial thiamine pyrophosphate carrier), member 19 ATP8B1 - atpase, aminophospholipid transporter, class i, type 8b, member 1 MFSD12 - major facilitator superfamily domain containing 12 EIF4ENIF1 - eukaryotic translation initiation factor 4e nuclear import factor 1 CACNG2 - calcium channel, voltage-dependent, gamma subunit 2 KCNQ5 - potassium voltage-gated channel, kqt-like subfamily, member 5 MCOLN1 - mucolipin 1 SLC25A38 - solute carrier family 25, member 38 CACNG3 - calcium channel, voltage-dependent, gamma subunit 3 KCNA2 - potassium voltage-gated channel, shaker-related subfamily, member 2 KCNA1 - potassium voltage-gated channel, shaker-related subfamily, member 1 (episodic ataxia with myokymia) LRRC8B - leucine rich repeat containing 8 family, member b SLC25A39 - solute carrier family 25, member 39 SLC45A4 - solute carrier family 45, member 4 SLC39A14 - solute carrier family 39 (zinc transporter), member 14 SLC25A25 - solute carrier family 25 (mitochondrial carrier; phosphate carrier), member 25 PKD2L1 - polycystic kidney disease 2-like 1 GABRG3 - gamma-aminobutyric acid (gaba) a receptor, gamma 3 TFR2 - transferrin receptor 2 KCNV1 - potassium channel, subfamily v, member 1 GRIA4 - glutamate receptor, ionotropic, ampa 4 GRIA3 - glutamate receptor, ionotropic, ampa 3 GRAMD1A - gram domain containing 1a SLC38A7 - solute carrier family 38, member 7 ITPR1 - inositol 1,4,5-trisphosphate receptor, type 1 ABCC10 - atp-binding cassette, sub-family c (cftr/mrp), member 10 GRIN2A - glutamate receptor, ionotropic, n-methyl d-aspartate 2a KCNJ12 - potassium inwardly-rectifying channel, subfamily j, member 12 KCNJ11 - potassium inwardly-rectifying channel, subfamily j, member 11 SLC45A1 - solute carrier family 45, member 1 GRIK1 - glutamate receptor, ionotropic, kainate 1 SLC41A3 - solute carrier family 41, member 3 GRIK2 - glutamate receptor, ionotropic, kainate 2 KCNK1 - potassium channel, subfamily k, member 1 GRIK3 - glutamate receptor, ionotropic, kainate 3 GRIK4 - glutamate receptor, ionotropic, kainate 4 CLCN7 - chloride channel, voltage-sensitive 7 SLC25A42 - solute carrier family 25, member 42 KCNJ4 - potassium inwardly-rectifying channel, subfamily j, member 4 GLRA3 - glycine receptor, alpha 3 KCNJ3 - potassium inwardly-rectifying channel, subfamily j, member 3 XPO6 - exportin 6 OSBPL10 - oxysterol binding protein-like 10 SLC25A41 - solute carrier family 25, member 41 KCNJ9 - potassium inwardly-rectifying channel, subfamily j, member 9 OSBPL5 - oxysterol binding protein-like 5 KCNJ8 - potassium inwardly-rectifying channel, subfamily j, member 8 KCNJ6 - potassium inwardly-rectifying channel, subfamily j, member 6 KCNF1 - potassium voltage-gated channel, subfamily f, member 1 ATP13A1 - atpase type 13a1 KCNH1 - potassium voltage-gated channel, subfamily h (eag-related), member 1 SLC27A2 - solute carrier family 27 (fatty acid transporter), member 2 KCNB1 - potassium voltage-gated channel, shab-related subfamily, member 1 SLC25A5 - solute carrier family 25 (mitochondrial carrier; adenine nucleotide translocator), member 5 KCNC1 - potassium voltage-gated channel, shaw-related subfamily, member 1 SLC27A4 - solute carrier family 27 (fatty acid transporter), member 4 KCNC2 - potassium voltage-gated channel, shaw-related subfamily, member 2 KCNK4 - potassium channel, subfamily k, member 4 KCNC3 - potassium voltage-gated channel, shaw-related subfamily, member 3 |
| GO:0032555 | purine ribonucleotide binding | 2.8E-7 | 4.35E-5 | 1.40 (16750,1693,1722,243) | TRIB3 - tribbles homolog 3 (drosophila) RFC2 - replication factor c (activator 1) 2, 40kda AGAP3 - arfgap with gtpase domain, ankyrin repeat and ph domain 3 BCKDK - branched chain ketoacid dehydrogenase kinase DHX30 - deah (asp-glu-ala-his) box helicase 30 LIMK1 - lim domain kinase 1 SEPT5 - septin 5 ABCA4 - atp-binding cassette, sub-family a (abc1), member 4 SPG7 - spastic paraplegia 7 (pure and complicated autosomal recessive) RET - ret proto-oncogene CDKL1 - cyclin-dependent kinase-like 1 (cdc2-related kinase) TUBA4A - tubulin, alpha 4a TUBA3C - tubulin, alpha 3c RAB3D - rab3d, member ras oncogene family BRSK1 - br serine/threonine kinase 1 CCT5 - chaperonin containing tcp1, subunit 5 (epsilon) PIP5KL1 - phosphatidylinositol-4-phosphate 5-kinase-like 1 SPHK2 - sphingosine kinase 2 UBE2J2 - ubiquitin-conjugating enzyme e2, j2 RAB37 - rab37, member ras oncogene family MAGI3 - membrane associated guanylate kinase, ww and pdz domain containing 3 TUBA4B - tubulin, alpha 4b (pseudogene) STK19 - serine/threonine kinase 19 RAB40C - rab40c, member ras oncogene family NME6 - nme/nm23 nucleoside diphosphate kinase 6 RIMKLA - ribosomal modification protein rimk-like family member a MYO5B - myosin vb MAST1 - microtubule associated serine/threonine kinase 1 EEF1A2 - eukaryotic translation elongation factor 1 alpha 2 MAST3 - microtubule associated serine/threonine kinase 3 ADCY6 - adenylate cyclase 6 MYO16 - myosin xvi PIM3 - pim-3 oncogene ADCY2 - adenylate cyclase 2 (brain) SNRNP200 - small nuclear ribonucleoprotein 200kda (u5) DIRAS2 - diras family, gtp-binding ras-like 2 FIGNL2 - fidgetin-like 2 PNCK - pregnancy up-regulated non-ubiquitously expressed cam kinase NRBP1 - nuclear receptor binding protein 1 KIF21B - kinesin family member 21b MVD - mevalonate (diphospho) decarboxylase TRPM4 - transient receptor potential cation channel, subfamily m, member 4 GNA15 - guanine nucleotide binding protein (g protein), alpha 15 (gq class) GMPPB - gdp-mannose pyrophosphorylase b GNA11 - guanine nucleotide binding protein (g protein), alpha 11 (gq class) ARL4A - adp-ribosylation factor-like 4a ARL4C - adp-ribosylation factor-like 4c PGS1 - phosphatidylglycerophosphate synthase 1 PAK6 - p21 protein (cdc42/rac)-activated kinase 6 TRAP1 - tnf receptor-associated protein 1 RUVBL2 - ruvb-like 2 (e. coli) PRKAA2 - protein kinase, amp-activated, alpha 2 catalytic subunit MYO15A - myosin xva NAT6 - n-acetyltransferase 6 (gcn5-related) CAMK1D - calcium/calmodulin-dependent protein kinase id CAMK2G - calcium/calmodulin-dependent protein kinase ii gamma AGK - acylglycerol kinase ADRBK2 - adrenergic, beta, receptor kinase 2 KIF3C - kinesin family member 3c ADRBK1 - adrenergic, beta, receptor kinase 1 CAMK2A - calcium/calmodulin-dependent protein kinase ii alpha KIF5A - kinesin family member 5a ABCC8 - atp-binding cassette, sub-family c (cftr/mrp), member 8 TIMM44 - translocase of inner mitochondrial membrane 44 homolog (yeast) KHK - ketohexokinase (fructokinase) PTK2B - protein tyrosine kinase 2 beta CIT - citron (rho-interacting, serine/threonine kinase 21) PPP5C - protein phosphatase 5, catalytic subunit CUL9 - cullin 9 CARS - cysteinyl-trna synthetase FARSA - phenylalanyl-trna synthetase, alpha subunit AACS - acetoacetyl-coa synthetase CAD - carbamoyl-phosphate synthetase 2, aspartate transcarbamylase, and dihydroorotase PINK1 - pten induced putative kinase 1 HK1 - hexokinase 1 XRCC6 - x-ray repair complementing defective repair in chinese hamster cells 6 UBE2E3 - ubiquitin-conjugating enzyme e2e 3 ADCK2 - aarf domain containing kinase 2 KIF25 - kinesin family member 25 KIFC2 - kinesin family member c2 DDX51 - dead (asp-glu-ala-asp) box polypeptide 51 CAMKK1 - calcium/calmodulin-dependent protein kinase kinase 1, alpha VARS - valyl-trna synthetase STK25 - serine/threonine kinase 25 RAPGEF4 - rap guanine nucleotide exchange factor (gef) 4 STK11 - serine/threonine kinase 11 RABL6 - rab, member ras oncogene family-like 6 TTLL12 - tubulin tyrosine ligase-like family, member 12 LONP1 - lon peptidase 1, mitochondrial EEFSEC - eukaryotic elongation factor, selenocysteine-trna-specific TUBA1B - tubulin, alpha 1b PRPS2 - phosphoribosyl pyrophosphate synthetase 2 GAK - cyclin g associated kinase PRPS1 - phosphoribosyl pyrophosphate synthetase 1 RAB26 - rab26, member ras oncogene family PRKY - protein kinase, y-linked, pseudogene CAMK1G - calcium/calmodulin-dependent protein kinase ig XYLB - xylulokinase homolog (h. influenzae) ACAD9 - acyl-coa dehydrogenase family, member 9 UCKL1 - uridine-cytidine kinase 1-like 1 TYRO3 - tyro3 protein tyrosine kinase ITPKA - inositol-trisphosphate 3-kinase a TYK2 - tyrosine kinase 2 MAP2K1 - mitogen-activated protein kinase kinase 1 DHX35 - deah (asp-glu-ala-his) box polypeptide 35 MAP2K2 - mitogen-activated protein kinase kinase 2 MAPK10 - mitogen-activated protein kinase 10 MAPK11 - mitogen-activated protein kinase 11 EPHA3 - eph receptor a3 KCNJ11 - potassium inwardly-rectifying channel, subfamily j, member 11 PKDCC - protein kinase domain containing, cytoplasmic MAPK3 - mitogen-activated protein kinase 3 PRKG2 - protein kinase, cgmp-dependent, type ii EPHB3 - eph receptor b3 EPHA5 - eph receptor a5 EPHA4 - eph receptor a4 KCNJ8 - potassium inwardly-rectifying channel, subfamily j, member 8 CSNK1E - casein kinase 1, epsilon ATP13A1 - atpase type 13a1 PRKCE - protein kinase c, epsilon EPHB6 - eph receptor b6 PRKCB - protein kinase c, beta SLC27A2 - solute carrier family 27 (fatty acid transporter), member 2 ADCK1 - aarf domain containing kinase 1 UBE2O - ubiquitin-conjugating enzyme e2o ERCC2 - excision repair cross-complementing rodent repair deficiency, complementation group 2 PAK7 - p21 protein (cdc42/rac)-activated kinase 7 CDC6 - cell division cycle 6 MTPAP - mitochondrial poly(a) polymerase DHX8 - deah (asp-glu-ala-his) box polypeptide 8 TUBA3D - tubulin, alpha 3d FLT3 - fms-related tyrosine kinase 3 MAP3K9 - mitogen-activated protein kinase kinase kinase 9 ANXA6 - annexin a6 TUBA8 - tubulin, alpha 8 RAB15 - rab15, member ras oncogene family MTHFD1L - methylenetetrahydrofolate dehydrogenase (nadp+ dependent) 1-like LRRK1 - leucine-rich repeat kinase 1 NLK - nemo-like kinase UBE2QL1 - ubiquitin-conjugating enzyme e2q family-like 1 MAGI1 - membrane associated guanylate kinase, ww and pdz domain containing 1 NUAK1 - nuak family, snf1-like kinase, 1 PAK1 - p21 protein (cdc42/rac)-activated kinase 1 RASL10A - ras-like, family 10, member a DGKZ - diacylglycerol kinase, zeta TUBA1A - tubulin, alpha 1a ABCB8 - atp-binding cassette, sub-family b (mdr/tap), member 8 CHD5 - chromodomain helicase dna binding protein 5 PDPK1 - 3-phosphoinositide dependent protein kinase-1 RAB24 - rab24, member ras oncogene family FES - feline sarcoma oncogene DHX38 - deah (asp-glu-ala-his) box polypeptide 38 DCLK1 - doublecortin-like kinase 1 SCN8A - sodium channel, voltage gated, type viii, alpha subunit CDK5 - cyclin-dependent kinase 5 ASNA1 - arsa arsenite transporter, atp-binding, homolog 1 (bacterial) DAPK3 - death-associated protein kinase 3 CDK9 - cyclin-dependent kinase 9 RND1 - rho family gtpase 1 DGKA - diacylglycerol kinase, alpha 80kda MET - met proto-oncogene MAP3K13 - mitogen-activated protein kinase kinase kinase 13 ARL9 - adp-ribosylation factor-like 9 ASS1 - argininosuccinate synthase 1 MAPK12 - mitogen-activated protein kinase 12 TNNI3K - tnni3 interacting kinase SARS - seryl-trna synthetase PDE2A - phosphodiesterase 2a, cgmp-stimulated TUBB8 - tubulin, beta 8 class viii PDE4A - phosphodiesterase 4a, camp-specific GPN2 - gpn-loop gtpase 2 GTPBP2 - gtp binding protein 2 RHEBL1 - ras homolog enriched in brain like 1 GNL1 - guanine nucleotide binding protein-like 1 ATP12A - atpase, h+/k+ transporting, nongastric, alpha polypeptide P2RX6 - purinergic receptor p2x, ligand-gated ion channel, 6 ATP1A1 - atpase, na+/k+ transporting, alpha 1 polypeptide CDK14 - cyclin-dependent kinase 14 NME1 - nme/nm23 nucleoside diphosphate kinase 1 ATP1A4 - atpase, na+/k+ transporting, alpha 4 polypeptide STK32C - serine/threonine kinase 32c RAB6B - rab6b, member ras oncogene family MORC2 - morc family cw-type zinc finger 2 MYO19 - myosin xix CHKB - choline kinase beta CNGB3 - cyclic nucleotide gated channel beta 3 MAP4K2 - mitogen-activated protein kinase kinase kinase kinase 2 ABCB9 - atp-binding cassette, sub-family b (mdr/tap), member 9 RAB3A - rab3a, member ras oncogene family ARL10 - adp-ribosylation factor-like 10 SKIV2L - superkiller viralicidic activity 2-like (s. cerevisiae) HCN1 - hyperpolarization activated cyclic nucleotide-gated potassium channel 1 ATP6V1B1 - atpase, h+ transporting, lysosomal 56/58kda, v1 subunit b1 PIP5K1B - phosphatidylinositol-4-phosphate 5-kinase, type i, beta MATK - megakaryocyte-associated tyrosine kinase STYK1 - serine/threonine/tyrosine kinase 1 PIF1 - pif1 5'-to-3' dna helicase TUBG2 - tubulin, gamma 2 RAC2 - ras-related c3 botulinum toxin substrate 2 (rho family, small gtp binding protein rac2) PFKP - phosphofructokinase, platelet ATP2B4 - atpase, ca++ transporting, plasma membrane 4 PFAS - phosphoribosylformylglycinamidine synthase ATP2B3 - atpase, ca++ transporting, plasma membrane 3 EHD2 - eh-domain containing 2 MAP3K6 - mitogen-activated protein kinase kinase kinase 6 INO80 - ino80 complex subunit EHD3 - eh-domain containing 3 DDX56 - dead (asp-glu-ala-asp) box helicase 56 RAB27B - rab27b, member ras oncogene family ATP8B1 - atpase, aminophospholipid transporter, class i, type 8b, member 1 GRK6 - g protein-coupled receptor kinase 6 PAPSS2 - 3'-phosphoadenosine 5'-phosphosulfate synthase 2 PANK4 - pantothenate kinase 4 PI4KA - phosphatidylinositol 4-kinase, catalytic, alpha TESK1 - testis-specific kinase 1 MYH7B - myosin, heavy chain 7b, cardiac muscle, beta TPK1 - thiamin pyrophosphokinase 1 RASD1 - ras, dexamethasone-induced 1 KIF1A - kinesin family member 1a FLAD1 - flavin adenine dinucleotide synthetase 1 BRSK2 - br serine/threonine kinase 2 LTK - leukocyte receptor tyrosine kinase CLK2 - cdc-like kinase 2 TWF2 - twinfilin actin-binding protein 2 RHOV - ras homolog family member v ABCC10 - atp-binding cassette, sub-family c (cftr/mrp), member 10 CLCN7 - chloride channel, voltage-sensitive 7 ACOT7 - acyl-coa thioesterase 7 KALRN - kalirin, rhogef kinase ATAD3B - atpase family, aaa domain containing 3b PHKG2 - phosphorylase kinase, gamma 2 (testis) RPS6KA4 - ribosomal protein s6 kinase, 90kda, polypeptide 4 DHX16 - deah (asp-glu-ala-his) box polypeptide 16 PPIP5K1 - diphosphoinositol pentakisphosphate kinase 1 CKMT1B - creatine kinase, mitochondrial 1b PIK3CD - phosphatidylinositol-4,5-bisphosphate 3-kinase, catalytic subunit delta PIM1 - pim-1 oncogene DNM1 - dynamin 1 UBE2D4 - ubiquitin-conjugating enzyme e2d 4 (putative) TARS2 - threonyl-trna synthetase 2, mitochondrial (putative) NAV2 - neuron navigator 2 GSK3A - glycogen synthase kinase 3 alpha RHOF - ras homolog family member f (in filopodia) |
| GO:0032553 | ribonucleotide binding | 3.75E-7 | 5.63E-5 | 1.39 (16750,1708,1722,244) | TRIB3 - tribbles homolog 3 (drosophila) RFC2 - replication factor c (activator 1) 2, 40kda AGAP3 - arfgap with gtpase domain, ankyrin repeat and ph domain 3 BCKDK - branched chain ketoacid dehydrogenase kinase DHX30 - deah (asp-glu-ala-his) box helicase 30 LIMK1 - lim domain kinase 1 SEPT5 - septin 5 ABCA4 - atp-binding cassette, sub-family a (abc1), member 4 SPG7 - spastic paraplegia 7 (pure and complicated autosomal recessive) RET - ret proto-oncogene CDKL1 - cyclin-dependent kinase-like 1 (cdc2-related kinase) TUBA4A - tubulin, alpha 4a TUBA3C - tubulin, alpha 3c RAB3D - rab3d, member ras oncogene family BRSK1 - br serine/threonine kinase 1 CCT5 - chaperonin containing tcp1, subunit 5 (epsilon) PIP5KL1 - phosphatidylinositol-4-phosphate 5-kinase-like 1 SPHK2 - sphingosine kinase 2 RAB37 - rab37, member ras oncogene family UBE2J2 - ubiquitin-conjugating enzyme e2, j2 MAGI3 - membrane associated guanylate kinase, ww and pdz domain containing 3 TUBA4B - tubulin, alpha 4b (pseudogene) STK19 - serine/threonine kinase 19 RAB40C - rab40c, member ras oncogene family NME6 - nme/nm23 nucleoside diphosphate kinase 6 RIMKLA - ribosomal modification protein rimk-like family member a MYO5B - myosin vb MAST1 - microtubule associated serine/threonine kinase 1 EEF1A2 - eukaryotic translation elongation factor 1 alpha 2 MAST3 - microtubule associated serine/threonine kinase 3 ADCY6 - adenylate cyclase 6 MYO16 - myosin xvi PIM3 - pim-3 oncogene ADCY2 - adenylate cyclase 2 (brain) SNRNP200 - small nuclear ribonucleoprotein 200kda (u5) DIRAS2 - diras family, gtp-binding ras-like 2 FIGNL2 - fidgetin-like 2 PNCK - pregnancy up-regulated non-ubiquitously expressed cam kinase NRBP1 - nuclear receptor binding protein 1 KIF21B - kinesin family member 21b MVD - mevalonate (diphospho) decarboxylase TRPM4 - transient receptor potential cation channel, subfamily m, member 4 GNA15 - guanine nucleotide binding protein (g protein), alpha 15 (gq class) GMPPB - gdp-mannose pyrophosphorylase b GNA11 - guanine nucleotide binding protein (g protein), alpha 11 (gq class) ARL4A - adp-ribosylation factor-like 4a ARL4C - adp-ribosylation factor-like 4c PGS1 - phosphatidylglycerophosphate synthase 1 PAK6 - p21 protein (cdc42/rac)-activated kinase 6 TRAP1 - tnf receptor-associated protein 1 RUVBL2 - ruvb-like 2 (e. coli) PRKAA2 - protein kinase, amp-activated, alpha 2 catalytic subunit MYO15A - myosin xva NAT6 - n-acetyltransferase 6 (gcn5-related) CAMK1D - calcium/calmodulin-dependent protein kinase id CAMK2G - calcium/calmodulin-dependent protein kinase ii gamma AGK - acylglycerol kinase ADRBK2 - adrenergic, beta, receptor kinase 2 KIF3C - kinesin family member 3c ADRBK1 - adrenergic, beta, receptor kinase 1 CAMK2A - calcium/calmodulin-dependent protein kinase ii alpha KIF5A - kinesin family member 5a ABCC8 - atp-binding cassette, sub-family c (cftr/mrp), member 8 TIMM44 - translocase of inner mitochondrial membrane 44 homolog (yeast) KHK - ketohexokinase (fructokinase) PTK2B - protein tyrosine kinase 2 beta CIT - citron (rho-interacting, serine/threonine kinase 21) PPP5C - protein phosphatase 5, catalytic subunit CUL9 - cullin 9 CARS - cysteinyl-trna synthetase FARSA - phenylalanyl-trna synthetase, alpha subunit AACS - acetoacetyl-coa synthetase CAD - carbamoyl-phosphate synthetase 2, aspartate transcarbamylase, and dihydroorotase PINK1 - pten induced putative kinase 1 HK1 - hexokinase 1 XRCC6 - x-ray repair complementing defective repair in chinese hamster cells 6 UBE2E3 - ubiquitin-conjugating enzyme e2e 3 ADCK2 - aarf domain containing kinase 2 KIF25 - kinesin family member 25 KIFC2 - kinesin family member c2 DDX51 - dead (asp-glu-ala-asp) box polypeptide 51 CAMKK1 - calcium/calmodulin-dependent protein kinase kinase 1, alpha VARS - valyl-trna synthetase STK25 - serine/threonine kinase 25 RAPGEF4 - rap guanine nucleotide exchange factor (gef) 4 STK11 - serine/threonine kinase 11 RABL6 - rab, member ras oncogene family-like 6 TTLL12 - tubulin tyrosine ligase-like family, member 12 LONP1 - lon peptidase 1, mitochondrial EEFSEC - eukaryotic elongation factor, selenocysteine-trna-specific TUBA1B - tubulin, alpha 1b PRPS2 - phosphoribosyl pyrophosphate synthetase 2 GAK - cyclin g associated kinase PRPS1 - phosphoribosyl pyrophosphate synthetase 1 RAB26 - rab26, member ras oncogene family PRKY - protein kinase, y-linked, pseudogene CAMK1G - calcium/calmodulin-dependent protein kinase ig XYLB - xylulokinase homolog (h. influenzae) ACAD9 - acyl-coa dehydrogenase family, member 9 UCKL1 - uridine-cytidine kinase 1-like 1 TYRO3 - tyro3 protein tyrosine kinase ITPKA - inositol-trisphosphate 3-kinase a TYK2 - tyrosine kinase 2 MAP2K1 - mitogen-activated protein kinase kinase 1 DHX35 - deah (asp-glu-ala-his) box polypeptide 35 MAP2K2 - mitogen-activated protein kinase kinase 2 MAPK10 - mitogen-activated protein kinase 10 MAPK11 - mitogen-activated protein kinase 11 EPHA3 - eph receptor a3 KCNJ11 - potassium inwardly-rectifying channel, subfamily j, member 11 PKDCC - protein kinase domain containing, cytoplasmic MAPK3 - mitogen-activated protein kinase 3 PRKG2 - protein kinase, cgmp-dependent, type ii EPHB3 - eph receptor b3 EPHA5 - eph receptor a5 EPHA4 - eph receptor a4 KCNJ8 - potassium inwardly-rectifying channel, subfamily j, member 8 CSNK1E - casein kinase 1, epsilon ATP13A1 - atpase type 13a1 PRKCE - protein kinase c, epsilon EPHB6 - eph receptor b6 PRKCB - protein kinase c, beta SLC27A2 - solute carrier family 27 (fatty acid transporter), member 2 ADCK1 - aarf domain containing kinase 1 UBE2O - ubiquitin-conjugating enzyme e2o ERCC2 - excision repair cross-complementing rodent repair deficiency, complementation group 2 PAK7 - p21 protein (cdc42/rac)-activated kinase 7 CDC6 - cell division cycle 6 MTPAP - mitochondrial poly(a) polymerase DHX8 - deah (asp-glu-ala-his) box polypeptide 8 TUBA3D - tubulin, alpha 3d FLT3 - fms-related tyrosine kinase 3 MAP3K9 - mitogen-activated protein kinase kinase kinase 9 ANXA6 - annexin a6 TUBA8 - tubulin, alpha 8 RAB15 - rab15, member ras oncogene family MTHFD1L - methylenetetrahydrofolate dehydrogenase (nadp+ dependent) 1-like LRRK1 - leucine-rich repeat kinase 1 NLK - nemo-like kinase UBE2QL1 - ubiquitin-conjugating enzyme e2q family-like 1 MAGI1 - membrane associated guanylate kinase, ww and pdz domain containing 1 NUAK1 - nuak family, snf1-like kinase, 1 PAK1 - p21 protein (cdc42/rac)-activated kinase 1 RASL10A - ras-like, family 10, member a DGKZ - diacylglycerol kinase, zeta TUBA1A - tubulin, alpha 1a ABCB8 - atp-binding cassette, sub-family b (mdr/tap), member 8 CHD5 - chromodomain helicase dna binding protein 5 PDPK1 - 3-phosphoinositide dependent protein kinase-1 RAB24 - rab24, member ras oncogene family FES - feline sarcoma oncogene DHX38 - deah (asp-glu-ala-his) box polypeptide 38 DCLK1 - doublecortin-like kinase 1 SCN8A - sodium channel, voltage gated, type viii, alpha subunit CDK5 - cyclin-dependent kinase 5 ASNA1 - arsa arsenite transporter, atp-binding, homolog 1 (bacterial) DAPK3 - death-associated protein kinase 3 CDK9 - cyclin-dependent kinase 9 RND1 - rho family gtpase 1 DGKA - diacylglycerol kinase, alpha 80kda MET - met proto-oncogene MAP3K13 - mitogen-activated protein kinase kinase kinase 13 ARL9 - adp-ribosylation factor-like 9 ASS1 - argininosuccinate synthase 1 MAPK12 - mitogen-activated protein kinase 12 TNNI3K - tnni3 interacting kinase SARS - seryl-trna synthetase PDE2A - phosphodiesterase 2a, cgmp-stimulated TUBB8 - tubulin, beta 8 class viii PDE4A - phosphodiesterase 4a, camp-specific GPN2 - gpn-loop gtpase 2 GTPBP2 - gtp binding protein 2 RHEBL1 - ras homolog enriched in brain like 1 NOS2 - nitric oxide synthase 2, inducible GNL1 - guanine nucleotide binding protein-like 1 ATP12A - atpase, h+/k+ transporting, nongastric, alpha polypeptide P2RX6 - purinergic receptor p2x, ligand-gated ion channel, 6 ATP1A1 - atpase, na+/k+ transporting, alpha 1 polypeptide CDK14 - cyclin-dependent kinase 14 NME1 - nme/nm23 nucleoside diphosphate kinase 1 ATP1A4 - atpase, na+/k+ transporting, alpha 4 polypeptide STK32C - serine/threonine kinase 32c RAB6B - rab6b, member ras oncogene family MORC2 - morc family cw-type zinc finger 2 MYO19 - myosin xix CHKB - choline kinase beta CNGB3 - cyclic nucleotide gated channel beta 3 MAP4K2 - mitogen-activated protein kinase kinase kinase kinase 2 ABCB9 - atp-binding cassette, sub-family b (mdr/tap), member 9 RAB3A - rab3a, member ras oncogene family ARL10 - adp-ribosylation factor-like 10 SKIV2L - superkiller viralicidic activity 2-like (s. cerevisiae) HCN1 - hyperpolarization activated cyclic nucleotide-gated potassium channel 1 ATP6V1B1 - atpase, h+ transporting, lysosomal 56/58kda, v1 subunit b1 PIP5K1B - phosphatidylinositol-4-phosphate 5-kinase, type i, beta MATK - megakaryocyte-associated tyrosine kinase STYK1 - serine/threonine/tyrosine kinase 1 PIF1 - pif1 5'-to-3' dna helicase TUBG2 - tubulin, gamma 2 RAC2 - ras-related c3 botulinum toxin substrate 2 (rho family, small gtp binding protein rac2) PFKP - phosphofructokinase, platelet ATP2B4 - atpase, ca++ transporting, plasma membrane 4 PFAS - phosphoribosylformylglycinamidine synthase ATP2B3 - atpase, ca++ transporting, plasma membrane 3 EHD2 - eh-domain containing 2 MAP3K6 - mitogen-activated protein kinase kinase kinase 6 INO80 - ino80 complex subunit EHD3 - eh-domain containing 3 RAB27B - rab27b, member ras oncogene family DDX56 - dead (asp-glu-ala-asp) box helicase 56 ATP8B1 - atpase, aminophospholipid transporter, class i, type 8b, member 1 GRK6 - g protein-coupled receptor kinase 6 PAPSS2 - 3'-phosphoadenosine 5'-phosphosulfate synthase 2 PANK4 - pantothenate kinase 4 PI4KA - phosphatidylinositol 4-kinase, catalytic, alpha TESK1 - testis-specific kinase 1 MYH7B - myosin, heavy chain 7b, cardiac muscle, beta TPK1 - thiamin pyrophosphokinase 1 RASD1 - ras, dexamethasone-induced 1 KIF1A - kinesin family member 1a FLAD1 - flavin adenine dinucleotide synthetase 1 BRSK2 - br serine/threonine kinase 2 LTK - leukocyte receptor tyrosine kinase CLK2 - cdc-like kinase 2 TWF2 - twinfilin actin-binding protein 2 RHOV - ras homolog family member v ABCC10 - atp-binding cassette, sub-family c (cftr/mrp), member 10 CLCN7 - chloride channel, voltage-sensitive 7 ACOT7 - acyl-coa thioesterase 7 KALRN - kalirin, rhogef kinase ATAD3B - atpase family, aaa domain containing 3b PHKG2 - phosphorylase kinase, gamma 2 (testis) RPS6KA4 - ribosomal protein s6 kinase, 90kda, polypeptide 4 DHX16 - deah (asp-glu-ala-his) box polypeptide 16 PPIP5K1 - diphosphoinositol pentakisphosphate kinase 1 CKMT1B - creatine kinase, mitochondrial 1b PIK3CD - phosphatidylinositol-4,5-bisphosphate 3-kinase, catalytic subunit delta PIM1 - pim-1 oncogene DNM1 - dynamin 1 UBE2D4 - ubiquitin-conjugating enzyme e2d 4 (putative) TARS2 - threonyl-trna synthetase 2, mitochondrial (putative) NAV2 - neuron navigator 2 GSK3A - glycogen synthase kinase 3 alpha RHOF - ras homolog family member f (in filopodia) |
| GO:0035639 | purine ribonucleoside triphosphate binding | 4.27E-7 | 6.2E-5 | 1.40 (16750,1635,1722,235) | TRIB3 - tribbles homolog 3 (drosophila) RFC2 - replication factor c (activator 1) 2, 40kda AGAP3 - arfgap with gtpase domain, ankyrin repeat and ph domain 3 BCKDK - branched chain ketoacid dehydrogenase kinase DHX30 - deah (asp-glu-ala-his) box helicase 30 LIMK1 - lim domain kinase 1 SEPT5 - septin 5 ABCA4 - atp-binding cassette, sub-family a (abc1), member 4 SPG7 - spastic paraplegia 7 (pure and complicated autosomal recessive) RET - ret proto-oncogene CDKL1 - cyclin-dependent kinase-like 1 (cdc2-related kinase) TUBA4A - tubulin, alpha 4a TUBA3C - tubulin, alpha 3c RAB3D - rab3d, member ras oncogene family BRSK1 - br serine/threonine kinase 1 CCT5 - chaperonin containing tcp1, subunit 5 (epsilon) PIP5KL1 - phosphatidylinositol-4-phosphate 5-kinase-like 1 SPHK2 - sphingosine kinase 2 UBE2J2 - ubiquitin-conjugating enzyme e2, j2 RAB37 - rab37, member ras oncogene family MAGI3 - membrane associated guanylate kinase, ww and pdz domain containing 3 TUBA4B - tubulin, alpha 4b (pseudogene) STK19 - serine/threonine kinase 19 RAB40C - rab40c, member ras oncogene family NME6 - nme/nm23 nucleoside diphosphate kinase 6 RIMKLA - ribosomal modification protein rimk-like family member a MYO5B - myosin vb MAST1 - microtubule associated serine/threonine kinase 1 EEF1A2 - eukaryotic translation elongation factor 1 alpha 2 MAST3 - microtubule associated serine/threonine kinase 3 ADCY6 - adenylate cyclase 6 MYO16 - myosin xvi PIM3 - pim-3 oncogene ADCY2 - adenylate cyclase 2 (brain) SNRNP200 - small nuclear ribonucleoprotein 200kda (u5) DIRAS2 - diras family, gtp-binding ras-like 2 FIGNL2 - fidgetin-like 2 PNCK - pregnancy up-regulated non-ubiquitously expressed cam kinase NRBP1 - nuclear receptor binding protein 1 KIF21B - kinesin family member 21b MVD - mevalonate (diphospho) decarboxylase TRPM4 - transient receptor potential cation channel, subfamily m, member 4 GNA15 - guanine nucleotide binding protein (g protein), alpha 15 (gq class) GMPPB - gdp-mannose pyrophosphorylase b GNA11 - guanine nucleotide binding protein (g protein), alpha 11 (gq class) ARL4A - adp-ribosylation factor-like 4a ARL4C - adp-ribosylation factor-like 4c PGS1 - phosphatidylglycerophosphate synthase 1 PAK6 - p21 protein (cdc42/rac)-activated kinase 6 TRAP1 - tnf receptor-associated protein 1 RUVBL2 - ruvb-like 2 (e. coli) PRKAA2 - protein kinase, amp-activated, alpha 2 catalytic subunit MYO15A - myosin xva CAMK1D - calcium/calmodulin-dependent protein kinase id CAMK2G - calcium/calmodulin-dependent protein kinase ii gamma AGK - acylglycerol kinase ADRBK2 - adrenergic, beta, receptor kinase 2 KIF3C - kinesin family member 3c ADRBK1 - adrenergic, beta, receptor kinase 1 CAMK2A - calcium/calmodulin-dependent protein kinase ii alpha KIF5A - kinesin family member 5a ABCC8 - atp-binding cassette, sub-family c (cftr/mrp), member 8 TIMM44 - translocase of inner mitochondrial membrane 44 homolog (yeast) KHK - ketohexokinase (fructokinase) PTK2B - protein tyrosine kinase 2 beta CIT - citron (rho-interacting, serine/threonine kinase 21) PPP5C - protein phosphatase 5, catalytic subunit CUL9 - cullin 9 CARS - cysteinyl-trna synthetase FARSA - phenylalanyl-trna synthetase, alpha subunit AACS - acetoacetyl-coa synthetase CAD - carbamoyl-phosphate synthetase 2, aspartate transcarbamylase, and dihydroorotase PINK1 - pten induced putative kinase 1 HK1 - hexokinase 1 XRCC6 - x-ray repair complementing defective repair in chinese hamster cells 6 UBE2E3 - ubiquitin-conjugating enzyme e2e 3 ADCK2 - aarf domain containing kinase 2 KIFC2 - kinesin family member c2 KIF25 - kinesin family member 25 DDX51 - dead (asp-glu-ala-asp) box polypeptide 51 CAMKK1 - calcium/calmodulin-dependent protein kinase kinase 1, alpha VARS - valyl-trna synthetase STK25 - serine/threonine kinase 25 STK11 - serine/threonine kinase 11 RABL6 - rab, member ras oncogene family-like 6 TTLL12 - tubulin tyrosine ligase-like family, member 12 LONP1 - lon peptidase 1, mitochondrial EEFSEC - eukaryotic elongation factor, selenocysteine-trna-specific TUBA1B - tubulin, alpha 1b PRPS2 - phosphoribosyl pyrophosphate synthetase 2 GAK - cyclin g associated kinase PRPS1 - phosphoribosyl pyrophosphate synthetase 1 RAB26 - rab26, member ras oncogene family PRKY - protein kinase, y-linked, pseudogene CAMK1G - calcium/calmodulin-dependent protein kinase ig XYLB - xylulokinase homolog (h. influenzae) UCKL1 - uridine-cytidine kinase 1-like 1 TYRO3 - tyro3 protein tyrosine kinase TYK2 - tyrosine kinase 2 ITPKA - inositol-trisphosphate 3-kinase a MAP2K1 - mitogen-activated protein kinase kinase 1 DHX35 - deah (asp-glu-ala-his) box polypeptide 35 MAP2K2 - mitogen-activated protein kinase kinase 2 MAPK10 - mitogen-activated protein kinase 10 MAPK11 - mitogen-activated protein kinase 11 EPHA3 - eph receptor a3 KCNJ11 - potassium inwardly-rectifying channel, subfamily j, member 11 PKDCC - protein kinase domain containing, cytoplasmic MAPK3 - mitogen-activated protein kinase 3 PRKG2 - protein kinase, cgmp-dependent, type ii EPHB3 - eph receptor b3 EPHA5 - eph receptor a5 EPHA4 - eph receptor a4 KCNJ8 - potassium inwardly-rectifying channel, subfamily j, member 8 CSNK1E - casein kinase 1, epsilon ATP13A1 - atpase type 13a1 PRKCE - protein kinase c, epsilon EPHB6 - eph receptor b6 PRKCB - protein kinase c, beta SLC27A2 - solute carrier family 27 (fatty acid transporter), member 2 ADCK1 - aarf domain containing kinase 1 UBE2O - ubiquitin-conjugating enzyme e2o PAK7 - p21 protein (cdc42/rac)-activated kinase 7 ERCC2 - excision repair cross-complementing rodent repair deficiency, complementation group 2 CDC6 - cell division cycle 6 MTPAP - mitochondrial poly(a) polymerase DHX8 - deah (asp-glu-ala-his) box polypeptide 8 FLT3 - fms-related tyrosine kinase 3 TUBA3D - tubulin, alpha 3d MAP3K9 - mitogen-activated protein kinase kinase kinase 9 ANXA6 - annexin a6 TUBA8 - tubulin, alpha 8 RAB15 - rab15, member ras oncogene family MTHFD1L - methylenetetrahydrofolate dehydrogenase (nadp+ dependent) 1-like LRRK1 - leucine-rich repeat kinase 1 NLK - nemo-like kinase UBE2QL1 - ubiquitin-conjugating enzyme e2q family-like 1 MAGI1 - membrane associated guanylate kinase, ww and pdz domain containing 1 NUAK1 - nuak family, snf1-like kinase, 1 PAK1 - p21 protein (cdc42/rac)-activated kinase 1 RASL10A - ras-like, family 10, member a DGKZ - diacylglycerol kinase, zeta TUBA1A - tubulin, alpha 1a ABCB8 - atp-binding cassette, sub-family b (mdr/tap), member 8 CHD5 - chromodomain helicase dna binding protein 5 PDPK1 - 3-phosphoinositide dependent protein kinase-1 RAB24 - rab24, member ras oncogene family FES - feline sarcoma oncogene DHX38 - deah (asp-glu-ala-his) box polypeptide 38 DCLK1 - doublecortin-like kinase 1 SCN8A - sodium channel, voltage gated, type viii, alpha subunit CDK5 - cyclin-dependent kinase 5 ASNA1 - arsa arsenite transporter, atp-binding, homolog 1 (bacterial) DAPK3 - death-associated protein kinase 3 CDK9 - cyclin-dependent kinase 9 RND1 - rho family gtpase 1 DGKA - diacylglycerol kinase, alpha 80kda MET - met proto-oncogene MAP3K13 - mitogen-activated protein kinase kinase kinase 13 ARL9 - adp-ribosylation factor-like 9 ASS1 - argininosuccinate synthase 1 MAPK12 - mitogen-activated protein kinase 12 TNNI3K - tnni3 interacting kinase SARS - seryl-trna synthetase TUBB8 - tubulin, beta 8 class viii GPN2 - gpn-loop gtpase 2 GTPBP2 - gtp binding protein 2 RHEBL1 - ras homolog enriched in brain like 1 GNL1 - guanine nucleotide binding protein-like 1 ATP12A - atpase, h+/k+ transporting, nongastric, alpha polypeptide P2RX6 - purinergic receptor p2x, ligand-gated ion channel, 6 ATP1A1 - atpase, na+/k+ transporting, alpha 1 polypeptide CDK14 - cyclin-dependent kinase 14 NME1 - nme/nm23 nucleoside diphosphate kinase 1 ATP1A4 - atpase, na+/k+ transporting, alpha 4 polypeptide STK32C - serine/threonine kinase 32c RAB6B - rab6b, member ras oncogene family MORC2 - morc family cw-type zinc finger 2 MYO19 - myosin xix CHKB - choline kinase beta MAP4K2 - mitogen-activated protein kinase kinase kinase kinase 2 ABCB9 - atp-binding cassette, sub-family b (mdr/tap), member 9 RAB3A - rab3a, member ras oncogene family ARL10 - adp-ribosylation factor-like 10 SKIV2L - superkiller viralicidic activity 2-like (s. cerevisiae) ATP6V1B1 - atpase, h+ transporting, lysosomal 56/58kda, v1 subunit b1 PIP5K1B - phosphatidylinositol-4-phosphate 5-kinase, type i, beta MATK - megakaryocyte-associated tyrosine kinase STYK1 - serine/threonine/tyrosine kinase 1 PIF1 - pif1 5'-to-3' dna helicase TUBG2 - tubulin, gamma 2 RAC2 - ras-related c3 botulinum toxin substrate 2 (rho family, small gtp binding protein rac2) PFKP - phosphofructokinase, platelet ATP2B4 - atpase, ca++ transporting, plasma membrane 4 PFAS - phosphoribosylformylglycinamidine synthase ATP2B3 - atpase, ca++ transporting, plasma membrane 3 EHD2 - eh-domain containing 2 MAP3K6 - mitogen-activated protein kinase kinase kinase 6 INO80 - ino80 complex subunit EHD3 - eh-domain containing 3 DDX56 - dead (asp-glu-ala-asp) box helicase 56 RAB27B - rab27b, member ras oncogene family ATP8B1 - atpase, aminophospholipid transporter, class i, type 8b, member 1 GRK6 - g protein-coupled receptor kinase 6 PAPSS2 - 3'-phosphoadenosine 5'-phosphosulfate synthase 2 PANK4 - pantothenate kinase 4 PI4KA - phosphatidylinositol 4-kinase, catalytic, alpha TESK1 - testis-specific kinase 1 MYH7B - myosin, heavy chain 7b, cardiac muscle, beta TPK1 - thiamin pyrophosphokinase 1 RASD1 - ras, dexamethasone-induced 1 KIF1A - kinesin family member 1a FLAD1 - flavin adenine dinucleotide synthetase 1 BRSK2 - br serine/threonine kinase 2 LTK - leukocyte receptor tyrosine kinase CLK2 - cdc-like kinase 2 TWF2 - twinfilin actin-binding protein 2 RHOV - ras homolog family member v ABCC10 - atp-binding cassette, sub-family c (cftr/mrp), member 10 CLCN7 - chloride channel, voltage-sensitive 7 KALRN - kalirin, rhogef kinase ATAD3B - atpase family, aaa domain containing 3b PHKG2 - phosphorylase kinase, gamma 2 (testis) RPS6KA4 - ribosomal protein s6 kinase, 90kda, polypeptide 4 DHX16 - deah (asp-glu-ala-his) box polypeptide 16 PPIP5K1 - diphosphoinositol pentakisphosphate kinase 1 CKMT1B - creatine kinase, mitochondrial 1b PIK3CD - phosphatidylinositol-4,5-bisphosphate 3-kinase, catalytic subunit delta PIM1 - pim-1 oncogene DNM1 - dynamin 1 UBE2D4 - ubiquitin-conjugating enzyme e2d 4 (putative) TARS2 - threonyl-trna synthetase 2, mitochondrial (putative) NAV2 - neuron navigator 2 GSK3A - glycogen synthase kinase 3 alpha RHOF - ras homolog family member f (in filopodia) |
| GO:0017076 | purine nucleotide binding | 4.81E-7 | 6.77E-5 | 1.39 (16750,1704,1722,243) | TRIB3 - tribbles homolog 3 (drosophila) RFC2 - replication factor c (activator 1) 2, 40kda AGAP3 - arfgap with gtpase domain, ankyrin repeat and ph domain 3 BCKDK - branched chain ketoacid dehydrogenase kinase DHX30 - deah (asp-glu-ala-his) box helicase 30 LIMK1 - lim domain kinase 1 SEPT5 - septin 5 ABCA4 - atp-binding cassette, sub-family a (abc1), member 4 SPG7 - spastic paraplegia 7 (pure and complicated autosomal recessive) RET - ret proto-oncogene CDKL1 - cyclin-dependent kinase-like 1 (cdc2-related kinase) TUBA4A - tubulin, alpha 4a TUBA3C - tubulin, alpha 3c RAB3D - rab3d, member ras oncogene family BRSK1 - br serine/threonine kinase 1 CCT5 - chaperonin containing tcp1, subunit 5 (epsilon) PIP5KL1 - phosphatidylinositol-4-phosphate 5-kinase-like 1 SPHK2 - sphingosine kinase 2 RAB37 - rab37, member ras oncogene family UBE2J2 - ubiquitin-conjugating enzyme e2, j2 MAGI3 - membrane associated guanylate kinase, ww and pdz domain containing 3 TUBA4B - tubulin, alpha 4b (pseudogene) STK19 - serine/threonine kinase 19 RAB40C - rab40c, member ras oncogene family NME6 - nme/nm23 nucleoside diphosphate kinase 6 RIMKLA - ribosomal modification protein rimk-like family member a MYO5B - myosin vb MAST1 - microtubule associated serine/threonine kinase 1 EEF1A2 - eukaryotic translation elongation factor 1 alpha 2 MAST3 - microtubule associated serine/threonine kinase 3 ADCY6 - adenylate cyclase 6 MYO16 - myosin xvi PIM3 - pim-3 oncogene ADCY2 - adenylate cyclase 2 (brain) SNRNP200 - small nuclear ribonucleoprotein 200kda (u5) DIRAS2 - diras family, gtp-binding ras-like 2 FIGNL2 - fidgetin-like 2 PNCK - pregnancy up-regulated non-ubiquitously expressed cam kinase NRBP1 - nuclear receptor binding protein 1 KIF21B - kinesin family member 21b MVD - mevalonate (diphospho) decarboxylase TRPM4 - transient receptor potential cation channel, subfamily m, member 4 GNA15 - guanine nucleotide binding protein (g protein), alpha 15 (gq class) GMPPB - gdp-mannose pyrophosphorylase b GNA11 - guanine nucleotide binding protein (g protein), alpha 11 (gq class) ARL4A - adp-ribosylation factor-like 4a ARL4C - adp-ribosylation factor-like 4c PGS1 - phosphatidylglycerophosphate synthase 1 PAK6 - p21 protein (cdc42/rac)-activated kinase 6 TRAP1 - tnf receptor-associated protein 1 RUVBL2 - ruvb-like 2 (e. coli) PRKAA2 - protein kinase, amp-activated, alpha 2 catalytic subunit MYO15A - myosin xva NAT6 - n-acetyltransferase 6 (gcn5-related) CAMK1D - calcium/calmodulin-dependent protein kinase id CAMK2G - calcium/calmodulin-dependent protein kinase ii gamma AGK - acylglycerol kinase ADRBK2 - adrenergic, beta, receptor kinase 2 KIF3C - kinesin family member 3c ADRBK1 - adrenergic, beta, receptor kinase 1 CAMK2A - calcium/calmodulin-dependent protein kinase ii alpha KIF5A - kinesin family member 5a ABCC8 - atp-binding cassette, sub-family c (cftr/mrp), member 8 TIMM44 - translocase of inner mitochondrial membrane 44 homolog (yeast) KHK - ketohexokinase (fructokinase) PTK2B - protein tyrosine kinase 2 beta CIT - citron (rho-interacting, serine/threonine kinase 21) PPP5C - protein phosphatase 5, catalytic subunit CUL9 - cullin 9 CARS - cysteinyl-trna synthetase FARSA - phenylalanyl-trna synthetase, alpha subunit AACS - acetoacetyl-coa synthetase CAD - carbamoyl-phosphate synthetase 2, aspartate transcarbamylase, and dihydroorotase PINK1 - pten induced putative kinase 1 HK1 - hexokinase 1 XRCC6 - x-ray repair complementing defective repair in chinese hamster cells 6 UBE2E3 - ubiquitin-conjugating enzyme e2e 3 ADCK2 - aarf domain containing kinase 2 KIF25 - kinesin family member 25 KIFC2 - kinesin family member c2 DDX51 - dead (asp-glu-ala-asp) box polypeptide 51 CAMKK1 - calcium/calmodulin-dependent protein kinase kinase 1, alpha VARS - valyl-trna synthetase STK25 - serine/threonine kinase 25 RAPGEF4 - rap guanine nucleotide exchange factor (gef) 4 STK11 - serine/threonine kinase 11 RABL6 - rab, member ras oncogene family-like 6 TTLL12 - tubulin tyrosine ligase-like family, member 12 LONP1 - lon peptidase 1, mitochondrial EEFSEC - eukaryotic elongation factor, selenocysteine-trna-specific TUBA1B - tubulin, alpha 1b PRPS2 - phosphoribosyl pyrophosphate synthetase 2 GAK - cyclin g associated kinase PRPS1 - phosphoribosyl pyrophosphate synthetase 1 RAB26 - rab26, member ras oncogene family PRKY - protein kinase, y-linked, pseudogene CAMK1G - calcium/calmodulin-dependent protein kinase ig XYLB - xylulokinase homolog (h. influenzae) ACAD9 - acyl-coa dehydrogenase family, member 9 UCKL1 - uridine-cytidine kinase 1-like 1 TYRO3 - tyro3 protein tyrosine kinase ITPKA - inositol-trisphosphate 3-kinase a TYK2 - tyrosine kinase 2 MAP2K1 - mitogen-activated protein kinase kinase 1 DHX35 - deah (asp-glu-ala-his) box polypeptide 35 MAP2K2 - mitogen-activated protein kinase kinase 2 MAPK10 - mitogen-activated protein kinase 10 MAPK11 - mitogen-activated protein kinase 11 EPHA3 - eph receptor a3 KCNJ11 - potassium inwardly-rectifying channel, subfamily j, member 11 PKDCC - protein kinase domain containing, cytoplasmic MAPK3 - mitogen-activated protein kinase 3 PRKG2 - protein kinase, cgmp-dependent, type ii EPHB3 - eph receptor b3 EPHA5 - eph receptor a5 EPHA4 - eph receptor a4 KCNJ8 - potassium inwardly-rectifying channel, subfamily j, member 8 CSNK1E - casein kinase 1, epsilon ATP13A1 - atpase type 13a1 PRKCE - protein kinase c, epsilon EPHB6 - eph receptor b6 PRKCB - protein kinase c, beta SLC27A2 - solute carrier family 27 (fatty acid transporter), member 2 ADCK1 - aarf domain containing kinase 1 UBE2O - ubiquitin-conjugating enzyme e2o ERCC2 - excision repair cross-complementing rodent repair deficiency, complementation group 2 PAK7 - p21 protein (cdc42/rac)-activated kinase 7 CDC6 - cell division cycle 6 MTPAP - mitochondrial poly(a) polymerase DHX8 - deah (asp-glu-ala-his) box polypeptide 8 TUBA3D - tubulin, alpha 3d FLT3 - fms-related tyrosine kinase 3 MAP3K9 - mitogen-activated protein kinase kinase kinase 9 ANXA6 - annexin a6 TUBA8 - tubulin, alpha 8 RAB15 - rab15, member ras oncogene family MTHFD1L - methylenetetrahydrofolate dehydrogenase (nadp+ dependent) 1-like LRRK1 - leucine-rich repeat kinase 1 NLK - nemo-like kinase UBE2QL1 - ubiquitin-conjugating enzyme e2q family-like 1 MAGI1 - membrane associated guanylate kinase, ww and pdz domain containing 1 NUAK1 - nuak family, snf1-like kinase, 1 PAK1 - p21 protein (cdc42/rac)-activated kinase 1 RASL10A - ras-like, family 10, member a DGKZ - diacylglycerol kinase, zeta TUBA1A - tubulin, alpha 1a ABCB8 - atp-binding cassette, sub-family b (mdr/tap), member 8 CHD5 - chromodomain helicase dna binding protein 5 PDPK1 - 3-phosphoinositide dependent protein kinase-1 RAB24 - rab24, member ras oncogene family FES - feline sarcoma oncogene DHX38 - deah (asp-glu-ala-his) box polypeptide 38 DCLK1 - doublecortin-like kinase 1 SCN8A - sodium channel, voltage gated, type viii, alpha subunit CDK5 - cyclin-dependent kinase 5 ASNA1 - arsa arsenite transporter, atp-binding, homolog 1 (bacterial) DAPK3 - death-associated protein kinase 3 CDK9 - cyclin-dependent kinase 9 RND1 - rho family gtpase 1 DGKA - diacylglycerol kinase, alpha 80kda MET - met proto-oncogene MAP3K13 - mitogen-activated protein kinase kinase kinase 13 ARL9 - adp-ribosylation factor-like 9 ASS1 - argininosuccinate synthase 1 MAPK12 - mitogen-activated protein kinase 12 TNNI3K - tnni3 interacting kinase SARS - seryl-trna synthetase PDE2A - phosphodiesterase 2a, cgmp-stimulated TUBB8 - tubulin, beta 8 class viii PDE4A - phosphodiesterase 4a, camp-specific GPN2 - gpn-loop gtpase 2 GTPBP2 - gtp binding protein 2 RHEBL1 - ras homolog enriched in brain like 1 GNL1 - guanine nucleotide binding protein-like 1 ATP12A - atpase, h+/k+ transporting, nongastric, alpha polypeptide P2RX6 - purinergic receptor p2x, ligand-gated ion channel, 6 ATP1A1 - atpase, na+/k+ transporting, alpha 1 polypeptide CDK14 - cyclin-dependent kinase 14 NME1 - nme/nm23 nucleoside diphosphate kinase 1 ATP1A4 - atpase, na+/k+ transporting, alpha 4 polypeptide STK32C - serine/threonine kinase 32c RAB6B - rab6b, member ras oncogene family MORC2 - morc family cw-type zinc finger 2 MYO19 - myosin xix CHKB - choline kinase beta CNGB3 - cyclic nucleotide gated channel beta 3 MAP4K2 - mitogen-activated protein kinase kinase kinase kinase 2 ABCB9 - atp-binding cassette, sub-family b (mdr/tap), member 9 RAB3A - rab3a, member ras oncogene family ARL10 - adp-ribosylation factor-like 10 SKIV2L - superkiller viralicidic activity 2-like (s. cerevisiae) HCN1 - hyperpolarization activated cyclic nucleotide-gated potassium channel 1 ATP6V1B1 - atpase, h+ transporting, lysosomal 56/58kda, v1 subunit b1 PIP5K1B - phosphatidylinositol-4-phosphate 5-kinase, type i, beta MATK - megakaryocyte-associated tyrosine kinase STYK1 - serine/threonine/tyrosine kinase 1 PIF1 - pif1 5'-to-3' dna helicase TUBG2 - tubulin, gamma 2 RAC2 - ras-related c3 botulinum toxin substrate 2 (rho family, small gtp binding protein rac2) PFKP - phosphofructokinase, platelet ATP2B4 - atpase, ca++ transporting, plasma membrane 4 PFAS - phosphoribosylformylglycinamidine synthase ATP2B3 - atpase, ca++ transporting, plasma membrane 3 EHD2 - eh-domain containing 2 MAP3K6 - mitogen-activated protein kinase kinase kinase 6 INO80 - ino80 complex subunit EHD3 - eh-domain containing 3 RAB27B - rab27b, member ras oncogene family DDX56 - dead (asp-glu-ala-asp) box helicase 56 ATP8B1 - atpase, aminophospholipid transporter, class i, type 8b, member 1 GRK6 - g protein-coupled receptor kinase 6 PAPSS2 - 3'-phosphoadenosine 5'-phosphosulfate synthase 2 PANK4 - pantothenate kinase 4 PI4KA - phosphatidylinositol 4-kinase, catalytic, alpha TESK1 - testis-specific kinase 1 MYH7B - myosin, heavy chain 7b, cardiac muscle, beta TPK1 - thiamin pyrophosphokinase 1 RASD1 - ras, dexamethasone-induced 1 KIF1A - kinesin family member 1a FLAD1 - flavin adenine dinucleotide synthetase 1 BRSK2 - br serine/threonine kinase 2 LTK - leukocyte receptor tyrosine kinase CLK2 - cdc-like kinase 2 TWF2 - twinfilin actin-binding protein 2 RHOV - ras homolog family member v ABCC10 - atp-binding cassette, sub-family c (cftr/mrp), member 10 CLCN7 - chloride channel, voltage-sensitive 7 ACOT7 - acyl-coa thioesterase 7 KALRN - kalirin, rhogef kinase ATAD3B - atpase family, aaa domain containing 3b PHKG2 - phosphorylase kinase, gamma 2 (testis) RPS6KA4 - ribosomal protein s6 kinase, 90kda, polypeptide 4 DHX16 - deah (asp-glu-ala-his) box polypeptide 16 PPIP5K1 - diphosphoinositol pentakisphosphate kinase 1 CKMT1B - creatine kinase, mitochondrial 1b PIK3CD - phosphatidylinositol-4,5-bisphosphate 3-kinase, catalytic subunit delta PIM1 - pim-1 oncogene DNM1 - dynamin 1 UBE2D4 - ubiquitin-conjugating enzyme e2d 4 (putative) TARS2 - threonyl-trna synthetase 2, mitochondrial (putative) NAV2 - neuron navigator 2 GSK3A - glycogen synthase kinase 3 alpha RHOF - ras homolog family member f (in filopodia) |
| GO:0004672 | protein kinase activity | 9.19E-7 | 1.25E-4 | 1.74 (16750,530,1651,91) | PRKAA2 - protein kinase, amp-activated, alpha 2 catalytic subunit PRKAB1 - protein kinase, amp-activated, beta 1 non-catalytic subunit FLT3 - fms-related tyrosine kinase 3 CAMK1D - calcium/calmodulin-dependent protein kinase id LIMK1 - lim domain kinase 1 CDK14 - cyclin-dependent kinase 14 BCKDK - branched chain ketoacid dehydrogenase kinase CAMK2G - calcium/calmodulin-dependent protein kinase ii gamma ADRBK2 - adrenergic, beta, receptor kinase 2 ADRBK1 - adrenergic, beta, receptor kinase 1 STK32C - serine/threonine kinase 32c CAMK2A - calcium/calmodulin-dependent protein kinase ii alpha MAP3K9 - mitogen-activated protein kinase kinase kinase 9 PTK2B - protein tyrosine kinase 2 beta RET - ret proto-oncogene CIT - citron (rho-interacting, serine/threonine kinase 21) CCNO - cyclin o CDKL1 - cyclin-dependent kinase-like 1 (cdc2-related kinase) CAD - carbamoyl-phosphate synthetase 2, aspartate transcarbamylase, and dihydroorotase PINK1 - pten induced putative kinase 1 BRSK1 - br serine/threonine kinase 1 LRRK1 - leucine-rich repeat kinase 1 ADCK2 - aarf domain containing kinase 2 NLK - nemo-like kinase MATK - megakaryocyte-associated tyrosine kinase STYK1 - serine/threonine/tyrosine kinase 1 CAMKK1 - calcium/calmodulin-dependent protein kinase kinase 1, alpha STK19 - serine/threonine kinase 19 TBRG4 - transforming growth factor beta regulator 4 NUAK1 - nuak family, snf1-like kinase, 1 STK25 - serine/threonine kinase 25 PAK1 - p21 protein (cdc42/rac)-activated kinase 1 STK11 - serine/threonine kinase 11 MAP3K6 - mitogen-activated protein kinase kinase kinase 6 MAST1 - microtubule associated serine/threonine kinase 1 ADCK5 - aarf domain containing kinase 5 GRK6 - g protein-coupled receptor kinase 6 FGFRL1 - fibroblast growth factor receptor-like 1 GAK - cyclin g associated kinase CCNE1 - cyclin e1 CCNG2 - cyclin g2 TESK1 - testis-specific kinase 1 CCNA1 - cyclin a1 PRKY - protein kinase, y-linked, pseudogene PDPK1 - 3-phosphoinositide dependent protein kinase-1 CAMK1G - calcium/calmodulin-dependent protein kinase ig FES - feline sarcoma oncogene MAST3 - microtubule associated serine/threonine kinase 3 TYRO3 - tyro3 protein tyrosine kinase PIM3 - pim-3 oncogene BRSK2 - br serine/threonine kinase 2 TYK2 - tyrosine kinase 2 ITPKA - inositol-trisphosphate 3-kinase a CLK2 - cdc-like kinase 2 MAP2K1 - mitogen-activated protein kinase kinase 1 DCLK1 - doublecortin-like kinase 1 LTK - leukocyte receptor tyrosine kinase EFNA3 - ephrin-a3 MAP2K2 - mitogen-activated protein kinase kinase 2 MAPK10 - mitogen-activated protein kinase 10 CDK5 - cyclin-dependent kinase 5 DAPK3 - death-associated protein kinase 3 EPHA3 - eph receptor a3 MAPK11 - mitogen-activated protein kinase 11 CDK9 - cyclin-dependent kinase 9 PKDCC - protein kinase domain containing, cytoplasmic MAPK3 - mitogen-activated protein kinase 3 PNCK - pregnancy up-regulated non-ubiquitously expressed cam kinase CCNI - cyclin i PRKG2 - protein kinase, cgmp-dependent, type ii MET - met proto-oncogene MAP3K13 - mitogen-activated protein kinase kinase kinase 13 EPHB3 - eph receptor b3 KALRN - kalirin, rhogef kinase EPHA5 - eph receptor a5 PHKG2 - phosphorylase kinase, gamma 2 (testis) EPHA4 - eph receptor a4 CSNK1E - casein kinase 1, epsilon RPS6KA4 - ribosomal protein s6 kinase, 90kda, polypeptide 4 MAPK12 - mitogen-activated protein kinase 12 TNNI3K - tnni3 interacting kinase PIM1 - pim-1 oncogene EPHB6 - eph receptor b6 PRKCE - protein kinase c, epsilon CCND2 - cyclin d2 PRKCB - protein kinase c, beta ADCK1 - aarf domain containing kinase 1 ERCC2 - excision repair cross-complementing rodent repair deficiency, complementation group 2 PAK7 - p21 protein (cdc42/rac)-activated kinase 7 GSK3A - glycogen synthase kinase 3 alpha PAK6 - p21 protein (cdc42/rac)-activated kinase 6 |
| GO:0005516 | calmodulin binding | 1.72E-6 | 2.28E-4 | 4.74 (16750,184,307,16) | MYO15A - myosin xva CNN1 - calponin 1, basic, smooth muscle NOS2 - nitric oxide synthase 2, inducible ARPP21 - camp-regulated phosphoprotein, 21kda RASGRF2 - ras protein-specific guanine nucleotide-releasing factor 2 CAMK2G - calcium/calmodulin-dependent protein kinase ii gamma SLC8A2 - solute carrier family 8 (sodium/calcium exchanger), member 2 CAMK2A - calcium/calmodulin-dependent protein kinase ii alpha CAMK1G - calcium/calmodulin-dependent protein kinase ig RGS4 - regulator of g-protein signaling 4 KCNH1 - potassium voltage-gated channel, subfamily h (eag-related), member 1 NRGN - neurogranin (protein kinase c substrate, rc3) UNC13A - unc-13 homolog a (c. elegans) ITPKA - inositol-trisphosphate 3-kinase a MYO5B - myosin vb KCNH5 - potassium voltage-gated channel, subfamily h (eag-related), member 5 |
| GO:0005272 | sodium channel activity | 3.36E-6 | 4.32E-4 | 4.77 (16750,35,1405,14) | SCN1A - sodium channel, voltage-gated, type i, alpha subunit ASIC2 - acid-sensing (proton-gated) ion channel 2 GRIK1 - glutamate receptor, ionotropic, kainate 1 SCN2B - sodium channel, voltage-gated, type ii, beta subunit KCNK1 - potassium channel, subfamily k, member 1 GRIK2 - glutamate receptor, ionotropic, kainate 2 GRIK3 - glutamate receptor, ionotropic, kainate 3 SCN1B - sodium channel, voltage-gated, type i, beta subunit GRIK4 - glutamate receptor, ionotropic, kainate 4 HCN1 - hyperpolarization activated cyclic nucleotide-gated potassium channel 1 SCN3B - sodium channel, voltage-gated, type iii, beta subunit PKD2L1 - polycystic kidney disease 2-like 1 TRPM2 - transient receptor potential cation channel, subfamily m, member 2 SHROOM2 - shroom family member 2 |
| GO:0032559 | adenyl ribonucleotide binding | 3.75E-6 | 4.69E-4 | 1.40 (16750,1383,1717,199) | TRIB3 - tribbles homolog 3 (drosophila) RFC2 - replication factor c (activator 1) 2, 40kda LIMK1 - lim domain kinase 1 BCKDK - branched chain ketoacid dehydrogenase kinase DHX30 - deah (asp-glu-ala-his) box helicase 30 ABCA4 - atp-binding cassette, sub-family a (abc1), member 4 SPG7 - spastic paraplegia 7 (pure and complicated autosomal recessive) RET - ret proto-oncogene CDKL1 - cyclin-dependent kinase-like 1 (cdc2-related kinase) BRSK1 - br serine/threonine kinase 1 CCT5 - chaperonin containing tcp1, subunit 5 (epsilon) PIP5KL1 - phosphatidylinositol-4-phosphate 5-kinase-like 1 SPHK2 - sphingosine kinase 2 UBE2J2 - ubiquitin-conjugating enzyme e2, j2 MAGI3 - membrane associated guanylate kinase, ww and pdz domain containing 3 STK19 - serine/threonine kinase 19 NME6 - nme/nm23 nucleoside diphosphate kinase 6 RIMKLA - ribosomal modification protein rimk-like family member a MYO5B - myosin vb MAST1 - microtubule associated serine/threonine kinase 1 MAST3 - microtubule associated serine/threonine kinase 3 ADCY6 - adenylate cyclase 6 MYO16 - myosin xvi PIM3 - pim-3 oncogene ADCY2 - adenylate cyclase 2 (brain) SNRNP200 - small nuclear ribonucleoprotein 200kda (u5) FIGNL2 - fidgetin-like 2 PNCK - pregnancy up-regulated non-ubiquitously expressed cam kinase NRBP1 - nuclear receptor binding protein 1 KIF21B - kinesin family member 21b TRPM4 - transient receptor potential cation channel, subfamily m, member 4 MVD - mevalonate (diphospho) decarboxylase PGS1 - phosphatidylglycerophosphate synthase 1 PAK6 - p21 protein (cdc42/rac)-activated kinase 6 RUVBL2 - ruvb-like 2 (e. coli) TRAP1 - tnf receptor-associated protein 1 PRKAA2 - protein kinase, amp-activated, alpha 2 catalytic subunit MYO15A - myosin xva NAT6 - n-acetyltransferase 6 (gcn5-related) CAMK1D - calcium/calmodulin-dependent protein kinase id CAMK2G - calcium/calmodulin-dependent protein kinase ii gamma AGK - acylglycerol kinase ADRBK2 - adrenergic, beta, receptor kinase 2 KIF3C - kinesin family member 3c ADRBK1 - adrenergic, beta, receptor kinase 1 CAMK2A - calcium/calmodulin-dependent protein kinase ii alpha KIF5A - kinesin family member 5a ABCC8 - atp-binding cassette, sub-family c (cftr/mrp), member 8 TIMM44 - translocase of inner mitochondrial membrane 44 homolog (yeast) KHK - ketohexokinase (fructokinase) PTK2B - protein tyrosine kinase 2 beta CIT - citron (rho-interacting, serine/threonine kinase 21) PPP5C - protein phosphatase 5, catalytic subunit CUL9 - cullin 9 CARS - cysteinyl-trna synthetase FARSA - phenylalanyl-trna synthetase, alpha subunit AACS - acetoacetyl-coa synthetase CAD - carbamoyl-phosphate synthetase 2, aspartate transcarbamylase, and dihydroorotase PINK1 - pten induced putative kinase 1 HK1 - hexokinase 1 XRCC6 - x-ray repair complementing defective repair in chinese hamster cells 6 UBE2E3 - ubiquitin-conjugating enzyme e2e 3 ADCK2 - aarf domain containing kinase 2 KIF25 - kinesin family member 25 KIFC2 - kinesin family member c2 DDX51 - dead (asp-glu-ala-asp) box polypeptide 51 CAMKK1 - calcium/calmodulin-dependent protein kinase kinase 1, alpha VARS - valyl-trna synthetase STK25 - serine/threonine kinase 25 RAPGEF4 - rap guanine nucleotide exchange factor (gef) 4 STK11 - serine/threonine kinase 11 TTLL12 - tubulin tyrosine ligase-like family, member 12 LONP1 - lon peptidase 1, mitochondrial GAK - cyclin g associated kinase PRPS2 - phosphoribosyl pyrophosphate synthetase 2 PRPS1 - phosphoribosyl pyrophosphate synthetase 1 PRKY - protein kinase, y-linked, pseudogene CAMK1G - calcium/calmodulin-dependent protein kinase ig XYLB - xylulokinase homolog (h. influenzae) UCKL1 - uridine-cytidine kinase 1-like 1 ACAD9 - acyl-coa dehydrogenase family, member 9 TYRO3 - tyro3 protein tyrosine kinase ITPKA - inositol-trisphosphate 3-kinase a TYK2 - tyrosine kinase 2 MAP2K1 - mitogen-activated protein kinase kinase 1 DHX35 - deah (asp-glu-ala-his) box polypeptide 35 MAP2K2 - mitogen-activated protein kinase kinase 2 MAPK10 - mitogen-activated protein kinase 10 MAPK11 - mitogen-activated protein kinase 11 EPHA3 - eph receptor a3 KCNJ11 - potassium inwardly-rectifying channel, subfamily j, member 11 PKDCC - protein kinase domain containing, cytoplasmic MAPK3 - mitogen-activated protein kinase 3 PRKG2 - protein kinase, cgmp-dependent, type ii EPHB3 - eph receptor b3 EPHA5 - eph receptor a5 EPHA4 - eph receptor a4 KCNJ8 - potassium inwardly-rectifying channel, subfamily j, member 8 CSNK1E - casein kinase 1, epsilon ATP13A1 - atpase type 13a1 PRKCE - protein kinase c, epsilon EPHB6 - eph receptor b6 PRKCB - protein kinase c, beta SLC27A2 - solute carrier family 27 (fatty acid transporter), member 2 ADCK1 - aarf domain containing kinase 1 UBE2O - ubiquitin-conjugating enzyme e2o ERCC2 - excision repair cross-complementing rodent repair deficiency, complementation group 2 PAK7 - p21 protein (cdc42/rac)-activated kinase 7 CDC6 - cell division cycle 6 MTPAP - mitochondrial poly(a) polymerase DHX8 - deah (asp-glu-ala-his) box polypeptide 8 FLT3 - fms-related tyrosine kinase 3 MAP3K9 - mitogen-activated protein kinase kinase kinase 9 MTHFD1L - methylenetetrahydrofolate dehydrogenase (nadp+ dependent) 1-like LRRK1 - leucine-rich repeat kinase 1 NLK - nemo-like kinase UBE2QL1 - ubiquitin-conjugating enzyme e2q family-like 1 MAGI1 - membrane associated guanylate kinase, ww and pdz domain containing 1 NUAK1 - nuak family, snf1-like kinase, 1 PAK1 - p21 protein (cdc42/rac)-activated kinase 1 DGKZ - diacylglycerol kinase, zeta ABCB8 - atp-binding cassette, sub-family b (mdr/tap), member 8 CHD5 - chromodomain helicase dna binding protein 5 PDPK1 - 3-phosphoinositide dependent protein kinase-1 FES - feline sarcoma oncogene DHX38 - deah (asp-glu-ala-his) box polypeptide 38 DCLK1 - doublecortin-like kinase 1 SCN8A - sodium channel, voltage gated, type viii, alpha subunit CDK5 - cyclin-dependent kinase 5 ASNA1 - arsa arsenite transporter, atp-binding, homolog 1 (bacterial) DAPK3 - death-associated protein kinase 3 CDK9 - cyclin-dependent kinase 9 DGKA - diacylglycerol kinase, alpha 80kda MET - met proto-oncogene MAP3K13 - mitogen-activated protein kinase kinase kinase 13 ASS1 - argininosuccinate synthase 1 MAPK12 - mitogen-activated protein kinase 12 SARS - seryl-trna synthetase TNNI3K - tnni3 interacting kinase PDE2A - phosphodiesterase 2a, cgmp-stimulated PDE4A - phosphodiesterase 4a, camp-specific ATP12A - atpase, h+/k+ transporting, nongastric, alpha polypeptide ATP1A1 - atpase, na+/k+ transporting, alpha 1 polypeptide P2RX6 - purinergic receptor p2x, ligand-gated ion channel, 6 CDK14 - cyclin-dependent kinase 14 NME1 - nme/nm23 nucleoside diphosphate kinase 1 ATP1A4 - atpase, na+/k+ transporting, alpha 4 polypeptide STK32C - serine/threonine kinase 32c MORC2 - morc family cw-type zinc finger 2 MYO19 - myosin xix CHKB - choline kinase beta MAP4K2 - mitogen-activated protein kinase kinase kinase kinase 2 ABCB9 - atp-binding cassette, sub-family b (mdr/tap), member 9 SKIV2L - superkiller viralicidic activity 2-like (s. cerevisiae) HCN1 - hyperpolarization activated cyclic nucleotide-gated potassium channel 1 ATP6V1B1 - atpase, h+ transporting, lysosomal 56/58kda, v1 subunit b1 PIP5K1B - phosphatidylinositol-4-phosphate 5-kinase, type i, beta MATK - megakaryocyte-associated tyrosine kinase STYK1 - serine/threonine/tyrosine kinase 1 PIF1 - pif1 5'-to-3' dna helicase PFKP - phosphofructokinase, platelet ATP2B4 - atpase, ca++ transporting, plasma membrane 4 ATP2B3 - atpase, ca++ transporting, plasma membrane 3 PFAS - phosphoribosylformylglycinamidine synthase EHD2 - eh-domain containing 2 INO80 - ino80 complex subunit MAP3K6 - mitogen-activated protein kinase kinase kinase 6 EHD3 - eh-domain containing 3 DDX56 - dead (asp-glu-ala-asp) box helicase 56 ATP8B1 - atpase, aminophospholipid transporter, class i, type 8b, member 1 GRK6 - g protein-coupled receptor kinase 6 PANK4 - pantothenate kinase 4 PAPSS2 - 3'-phosphoadenosine 5'-phosphosulfate synthase 2 PI4KA - phosphatidylinositol 4-kinase, catalytic, alpha TESK1 - testis-specific kinase 1 TPK1 - thiamin pyrophosphokinase 1 MYH7B - myosin, heavy chain 7b, cardiac muscle, beta KIF1A - kinesin family member 1a FLAD1 - flavin adenine dinucleotide synthetase 1 BRSK2 - br serine/threonine kinase 2 CLK2 - cdc-like kinase 2 LTK - leukocyte receptor tyrosine kinase TWF2 - twinfilin actin-binding protein 2 ABCC10 - atp-binding cassette, sub-family c (cftr/mrp), member 10 CLCN7 - chloride channel, voltage-sensitive 7 ACOT7 - acyl-coa thioesterase 7 KALRN - kalirin, rhogef kinase PHKG2 - phosphorylase kinase, gamma 2 (testis) ATAD3B - atpase family, aaa domain containing 3b RPS6KA4 - ribosomal protein s6 kinase, 90kda, polypeptide 4 DHX16 - deah (asp-glu-ala-his) box polypeptide 16 PPIP5K1 - diphosphoinositol pentakisphosphate kinase 1 CKMT1B - creatine kinase, mitochondrial 1b PIK3CD - phosphatidylinositol-4,5-bisphosphate 3-kinase, catalytic subunit delta PIM1 - pim-1 oncogene UBE2D4 - ubiquitin-conjugating enzyme e2d 4 (putative) TARS2 - threonyl-trna synthetase 2, mitochondrial (putative) GSK3A - glycogen synthase kinase 3 alpha NAV2 - neuron navigator 2 |
| GO:0004970 | ionotropic glutamate receptor activity | 4.01E-6 | 4.87E-4 | 7.15 (16750,15,1405,9) | GRIN2A - glutamate receptor, ionotropic, n-methyl d-aspartate 2a GRIK1 - glutamate receptor, ionotropic, kainate 1 PTK2B - protein tyrosine kinase 2 beta GRIK2 - glutamate receptor, ionotropic, kainate 2 GRIK3 - glutamate receptor, ionotropic, kainate 3 GRIK4 - glutamate receptor, ionotropic, kainate 4 GRIA4 - glutamate receptor, ionotropic, ampa 4 GRIA3 - glutamate receptor, ionotropic, ampa 3 GRIN3A - glutamate receptor, ionotropic, n-methyl-d-aspartate 3a |
| GO:0005524 | ATP binding | 5.01E-6 | 5.94E-4 | 1.41 (16750,1331,1717,192) | CDC6 - cell division cycle 6 TRIB3 - tribbles homolog 3 (drosophila) DHX8 - deah (asp-glu-ala-his) box polypeptide 8 MTPAP - mitochondrial poly(a) polymerase RFC2 - replication factor c (activator 1) 2, 40kda FLT3 - fms-related tyrosine kinase 3 DHX30 - deah (asp-glu-ala-his) box helicase 30 BCKDK - branched chain ketoacid dehydrogenase kinase LIMK1 - lim domain kinase 1 MAP3K9 - mitogen-activated protein kinase kinase kinase 9 ABCA4 - atp-binding cassette, sub-family a (abc1), member 4 SPG7 - spastic paraplegia 7 (pure and complicated autosomal recessive) RET - ret proto-oncogene CDKL1 - cyclin-dependent kinase-like 1 (cdc2-related kinase) BRSK1 - br serine/threonine kinase 1 MTHFD1L - methylenetetrahydrofolate dehydrogenase (nadp+ dependent) 1-like CCT5 - chaperonin containing tcp1, subunit 5 (epsilon) LRRK1 - leucine-rich repeat kinase 1 PIP5KL1 - phosphatidylinositol-4-phosphate 5-kinase-like 1 SPHK2 - sphingosine kinase 2 NLK - nemo-like kinase UBE2J2 - ubiquitin-conjugating enzyme e2, j2 UBE2QL1 - ubiquitin-conjugating enzyme e2q family-like 1 MAGI3 - membrane associated guanylate kinase, ww and pdz domain containing 3 STK19 - serine/threonine kinase 19 NME6 - nme/nm23 nucleoside diphosphate kinase 6 RIMKLA - ribosomal modification protein rimk-like family member a MAGI1 - membrane associated guanylate kinase, ww and pdz domain containing 1 NUAK1 - nuak family, snf1-like kinase, 1 PAK1 - p21 protein (cdc42/rac)-activated kinase 1 MYO5B - myosin vb MAST1 - microtubule associated serine/threonine kinase 1 DGKZ - diacylglycerol kinase, zeta ABCB8 - atp-binding cassette, sub-family b (mdr/tap), member 8 CHD5 - chromodomain helicase dna binding protein 5 PDPK1 - 3-phosphoinositide dependent protein kinase-1 FES - feline sarcoma oncogene MAST3 - microtubule associated serine/threonine kinase 3 ADCY6 - adenylate cyclase 6 MYO16 - myosin xvi DHX38 - deah (asp-glu-ala-his) box polypeptide 38 PIM3 - pim-3 oncogene DCLK1 - doublecortin-like kinase 1 SCN8A - sodium channel, voltage gated, type viii, alpha subunit SNRNP200 - small nuclear ribonucleoprotein 200kda (u5) ADCY2 - adenylate cyclase 2 (brain) DAPK3 - death-associated protein kinase 3 ASNA1 - arsa arsenite transporter, atp-binding, homolog 1 (bacterial) CDK5 - cyclin-dependent kinase 5 CDK9 - cyclin-dependent kinase 9 FIGNL2 - fidgetin-like 2 PNCK - pregnancy up-regulated non-ubiquitously expressed cam kinase DGKA - diacylglycerol kinase, alpha 80kda NRBP1 - nuclear receptor binding protein 1 MET - met proto-oncogene KIF21B - kinesin family member 21b MAP3K13 - mitogen-activated protein kinase kinase kinase 13 MVD - mevalonate (diphospho) decarboxylase TRPM4 - transient receptor potential cation channel, subfamily m, member 4 ASS1 - argininosuccinate synthase 1 MAPK12 - mitogen-activated protein kinase 12 TNNI3K - tnni3 interacting kinase SARS - seryl-trna synthetase PGS1 - phosphatidylglycerophosphate synthase 1 PAK6 - p21 protein (cdc42/rac)-activated kinase 6 TRAP1 - tnf receptor-associated protein 1 RUVBL2 - ruvb-like 2 (e. coli) PRKAA2 - protein kinase, amp-activated, alpha 2 catalytic subunit MYO15A - myosin xva CAMK1D - calcium/calmodulin-dependent protein kinase id ATP12A - atpase, h+/k+ transporting, nongastric, alpha polypeptide P2RX6 - purinergic receptor p2x, ligand-gated ion channel, 6 ATP1A1 - atpase, na+/k+ transporting, alpha 1 polypeptide CDK14 - cyclin-dependent kinase 14 NME1 - nme/nm23 nucleoside diphosphate kinase 1 ADRBK2 - adrenergic, beta, receptor kinase 2 AGK - acylglycerol kinase CAMK2G - calcium/calmodulin-dependent protein kinase ii gamma KIF3C - kinesin family member 3c ADRBK1 - adrenergic, beta, receptor kinase 1 ATP1A4 - atpase, na+/k+ transporting, alpha 4 polypeptide STK32C - serine/threonine kinase 32c KIF5A - kinesin family member 5a CAMK2A - calcium/calmodulin-dependent protein kinase ii alpha ABCC8 - atp-binding cassette, sub-family c (cftr/mrp), member 8 MORC2 - morc family cw-type zinc finger 2 KHK - ketohexokinase (fructokinase) TIMM44 - translocase of inner mitochondrial membrane 44 homolog (yeast) MYO19 - myosin xix PTK2B - protein tyrosine kinase 2 beta CIT - citron (rho-interacting, serine/threonine kinase 21) CHKB - choline kinase beta CUL9 - cullin 9 PPP5C - protein phosphatase 5, catalytic subunit FARSA - phenylalanyl-trna synthetase, alpha subunit CARS - cysteinyl-trna synthetase AACS - acetoacetyl-coa synthetase CAD - carbamoyl-phosphate synthetase 2, aspartate transcarbamylase, and dihydroorotase MAP4K2 - mitogen-activated protein kinase kinase kinase kinase 2 ABCB9 - atp-binding cassette, sub-family b (mdr/tap), member 9 PINK1 - pten induced putative kinase 1 HK1 - hexokinase 1 SKIV2L - superkiller viralicidic activity 2-like (s. cerevisiae) XRCC6 - x-ray repair complementing defective repair in chinese hamster cells 6 UBE2E3 - ubiquitin-conjugating enzyme e2e 3 ADCK2 - aarf domain containing kinase 2 ATP6V1B1 - atpase, h+ transporting, lysosomal 56/58kda, v1 subunit b1 PIP5K1B - phosphatidylinositol-4-phosphate 5-kinase, type i, beta KIFC2 - kinesin family member c2 MATK - megakaryocyte-associated tyrosine kinase KIF25 - kinesin family member 25 STYK1 - serine/threonine/tyrosine kinase 1 DDX51 - dead (asp-glu-ala-asp) box polypeptide 51 PIF1 - pif1 5'-to-3' dna helicase CAMKK1 - calcium/calmodulin-dependent protein kinase kinase 1, alpha VARS - valyl-trna synthetase PFKP - phosphofructokinase, platelet ATP2B4 - atpase, ca++ transporting, plasma membrane 4 ATP2B3 - atpase, ca++ transporting, plasma membrane 3 PFAS - phosphoribosylformylglycinamidine synthase STK25 - serine/threonine kinase 25 STK11 - serine/threonine kinase 11 EHD2 - eh-domain containing 2 MAP3K6 - mitogen-activated protein kinase kinase kinase 6 INO80 - ino80 complex subunit EHD3 - eh-domain containing 3 DDX56 - dead (asp-glu-ala-asp) box helicase 56 ATP8B1 - atpase, aminophospholipid transporter, class i, type 8b, member 1 TTLL12 - tubulin tyrosine ligase-like family, member 12 LONP1 - lon peptidase 1, mitochondrial GRK6 - g protein-coupled receptor kinase 6 PAPSS2 - 3'-phosphoadenosine 5'-phosphosulfate synthase 2 PANK4 - pantothenate kinase 4 GAK - cyclin g associated kinase PRPS2 - phosphoribosyl pyrophosphate synthetase 2 PRPS1 - phosphoribosyl pyrophosphate synthetase 1 PI4KA - phosphatidylinositol 4-kinase, catalytic, alpha TESK1 - testis-specific kinase 1 PRKY - protein kinase, y-linked, pseudogene CAMK1G - calcium/calmodulin-dependent protein kinase ig XYLB - xylulokinase homolog (h. influenzae) MYH7B - myosin, heavy chain 7b, cardiac muscle, beta TPK1 - thiamin pyrophosphokinase 1 UCKL1 - uridine-cytidine kinase 1-like 1 TYRO3 - tyro3 protein tyrosine kinase KIF1A - kinesin family member 1a FLAD1 - flavin adenine dinucleotide synthetase 1 BRSK2 - br serine/threonine kinase 2 TYK2 - tyrosine kinase 2 ITPKA - inositol-trisphosphate 3-kinase a LTK - leukocyte receptor tyrosine kinase DHX35 - deah (asp-glu-ala-his) box polypeptide 35 MAP2K1 - mitogen-activated protein kinase kinase 1 CLK2 - cdc-like kinase 2 MAP2K2 - mitogen-activated protein kinase kinase 2 TWF2 - twinfilin actin-binding protein 2 MAPK10 - mitogen-activated protein kinase 10 EPHA3 - eph receptor a3 ABCC10 - atp-binding cassette, sub-family c (cftr/mrp), member 10 MAPK11 - mitogen-activated protein kinase 11 KCNJ11 - potassium inwardly-rectifying channel, subfamily j, member 11 PKDCC - protein kinase domain containing, cytoplasmic MAPK3 - mitogen-activated protein kinase 3 CLCN7 - chloride channel, voltage-sensitive 7 PRKG2 - protein kinase, cgmp-dependent, type ii EPHB3 - eph receptor b3 KALRN - kalirin, rhogef kinase EPHA5 - eph receptor a5 ATAD3B - atpase family, aaa domain containing 3b PHKG2 - phosphorylase kinase, gamma 2 (testis) EPHA4 - eph receptor a4 KCNJ8 - potassium inwardly-rectifying channel, subfamily j, member 8 RPS6KA4 - ribosomal protein s6 kinase, 90kda, polypeptide 4 CSNK1E - casein kinase 1, epsilon DHX16 - deah (asp-glu-ala-his) box polypeptide 16 PPIP5K1 - diphosphoinositol pentakisphosphate kinase 1 ATP13A1 - atpase type 13a1 PIK3CD - phosphatidylinositol-4,5-bisphosphate 3-kinase, catalytic subunit delta CKMT1B - creatine kinase, mitochondrial 1b PIM1 - pim-1 oncogene EPHB6 - eph receptor b6 PRKCE - protein kinase c, epsilon PRKCB - protein kinase c, beta UBE2D4 - ubiquitin-conjugating enzyme e2d 4 (putative) SLC27A2 - solute carrier family 27 (fatty acid transporter), member 2 TARS2 - threonyl-trna synthetase 2, mitochondrial (putative) ADCK1 - aarf domain containing kinase 1 ERCC2 - excision repair cross-complementing rodent repair deficiency, complementation group 2 PAK7 - p21 protein (cdc42/rac)-activated kinase 7 NAV2 - neuron navigator 2 UBE2O - ubiquitin-conjugating enzyme e2o GSK3A - glycogen synthase kinase 3 alpha |
| GO:0008144 | drug binding | 5.55E-6 | 6.41E-4 | 1.38 (16750,1552,1668,213) | TRIB3 - tribbles homolog 3 (drosophila) RFC2 - replication factor c (activator 1) 2, 40kda BCKDK - branched chain ketoacid dehydrogenase kinase DHX30 - deah (asp-glu-ala-his) box helicase 30 LIMK1 - lim domain kinase 1 ABCA4 - atp-binding cassette, sub-family a (abc1), member 4 RET - ret proto-oncogene CDKL1 - cyclin-dependent kinase-like 1 (cdc2-related kinase) BRSK1 - br serine/threonine kinase 1 CCT5 - chaperonin containing tcp1, subunit 5 (epsilon) PIP5KL1 - phosphatidylinositol-4-phosphate 5-kinase-like 1 SPHK2 - sphingosine kinase 2 UBE2J2 - ubiquitin-conjugating enzyme e2, j2 MAGI3 - membrane associated guanylate kinase, ww and pdz domain containing 3 STK19 - serine/threonine kinase 19 NME6 - nme/nm23 nucleoside diphosphate kinase 6 RIMKLA - ribosomal modification protein rimk-like family member a MYO5B - myosin vb MAST1 - microtubule associated serine/threonine kinase 1 PPID - peptidylprolyl isomerase d PPIA - peptidylprolyl isomerase a (cyclophilin a) GLDC - glycine dehydrogenase (decarboxylating) MAST3 - microtubule associated serine/threonine kinase 3 ADCY6 - adenylate cyclase 6 NGB - neuroglobin MYO16 - myosin xvi PIM3 - pim-3 oncogene HTR2A - 5-hydroxytryptamine (serotonin) receptor 2a, g protein-coupled ADCY2 - adenylate cyclase 2 (brain) GRIN3A - glutamate receptor, ionotropic, n-methyl-d-aspartate 3a SNRNP200 - small nuclear ribonucleoprotein 200kda (u5) FIGNL2 - fidgetin-like 2 PNCK - pregnancy up-regulated non-ubiquitously expressed cam kinase NRBP1 - nuclear receptor binding protein 1 KIF21B - kinesin family member 21b MVD - mevalonate (diphospho) decarboxylase TRPM4 - transient receptor potential cation channel, subfamily m, member 4 CSAD - cysteine sulfinic acid decarboxylase PGS1 - phosphatidylglycerophosphate synthase 1 PAK6 - p21 protein (cdc42/rac)-activated kinase 6 TRAP1 - tnf receptor-associated protein 1 RUVBL2 - ruvb-like 2 (e. coli) PRKAA2 - protein kinase, amp-activated, alpha 2 catalytic subunit MYO15A - myosin xva MTR - 5-methyltetrahydrofolate-homocysteine methyltransferase PPIE - peptidylprolyl isomerase e (cyclophilin e) CAMK1D - calcium/calmodulin-dependent protein kinase id CAMK2G - calcium/calmodulin-dependent protein kinase ii gamma ADRBK2 - adrenergic, beta, receptor kinase 2 AGK - acylglycerol kinase KIF3C - kinesin family member 3c ADRBK1 - adrenergic, beta, receptor kinase 1 CAMK2A - calcium/calmodulin-dependent protein kinase ii alpha KIF5A - kinesin family member 5a ABCC8 - atp-binding cassette, sub-family c (cftr/mrp), member 8 TIMM44 - translocase of inner mitochondrial membrane 44 homolog (yeast) KHK - ketohexokinase (fructokinase) PTK2B - protein tyrosine kinase 2 beta PPP3R1 - protein phosphatase 3, regulatory subunit b, alpha CIT - citron (rho-interacting, serine/threonine kinase 21) PPP5C - protein phosphatase 5, catalytic subunit CUL9 - cullin 9 CARS - cysteinyl-trna synthetase FARSA - phenylalanyl-trna synthetase, alpha subunit FASN - fatty acid synthase AACS - acetoacetyl-coa synthetase CAD - carbamoyl-phosphate synthetase 2, aspartate transcarbamylase, and dihydroorotase PINK1 - pten induced putative kinase 1 HK1 - hexokinase 1 XRCC6 - x-ray repair complementing defective repair in chinese hamster cells 6 UBE2E3 - ubiquitin-conjugating enzyme e2e 3 GABRA1 - gamma-aminobutyric acid (gaba) a receptor, alpha 1 ADCK2 - aarf domain containing kinase 2 KIFC2 - kinesin family member c2 KIF25 - kinesin family member 25 DDX51 - dead (asp-glu-ala-asp) box polypeptide 51 CAMKK1 - calcium/calmodulin-dependent protein kinase kinase 1, alpha VARS - valyl-trna synthetase STK25 - serine/threonine kinase 25 STK11 - serine/threonine kinase 11 ALAS1 - aminolevulinate, delta-, synthase 1 TTLL12 - tubulin tyrosine ligase-like family, member 12 LONP1 - lon peptidase 1, mitochondrial PRPS2 - phosphoribosyl pyrophosphate synthetase 2 GAK - cyclin g associated kinase PRPS1 - phosphoribosyl pyrophosphate synthetase 1 PRKY - protein kinase, y-linked, pseudogene CAMK1G - calcium/calmodulin-dependent protein kinase ig XYLB - xylulokinase homolog (h. influenzae) UCKL1 - uridine-cytidine kinase 1-like 1 TYRO3 - tyro3 protein tyrosine kinase TYK2 - tyrosine kinase 2 ITPKA - inositol-trisphosphate 3-kinase a DHX35 - deah (asp-glu-ala-his) box polypeptide 35 MAP2K1 - mitogen-activated protein kinase kinase 1 MAP2K2 - mitogen-activated protein kinase kinase 2 GAD1 - glutamate decarboxylase 1 (brain, 67kda) MAPK10 - mitogen-activated protein kinase 10 MAPK11 - mitogen-activated protein kinase 11 EPHA3 - eph receptor a3 KCNJ11 - potassium inwardly-rectifying channel, subfamily j, member 11 PKDCC - protein kinase domain containing, cytoplasmic MAPK3 - mitogen-activated protein kinase 3 PRKG2 - protein kinase, cgmp-dependent, type ii GLRA3 - glycine receptor, alpha 3 EPHB3 - eph receptor b3 EPHA5 - eph receptor a5 EPHA4 - eph receptor a4 KCNJ8 - potassium inwardly-rectifying channel, subfamily j, member 8 CSNK1E - casein kinase 1, epsilon ATP13A1 - atpase type 13a1 PRKCE - protein kinase c, epsilon EPHB6 - eph receptor b6 PRKCB - protein kinase c, beta SLC27A2 - solute carrier family 27 (fatty acid transporter), member 2 ADCK1 - aarf domain containing kinase 1 PAK7 - p21 protein (cdc42/rac)-activated kinase 7 ERCC2 - excision repair cross-complementing rodent repair deficiency, complementation group 2 CDC6 - cell division cycle 6 DHX8 - deah (asp-glu-ala-his) box polypeptide 8 MTPAP - mitochondrial poly(a) polymerase FLT3 - fms-related tyrosine kinase 3 MAP3K9 - mitogen-activated protein kinase kinase kinase 9 MTHFD1L - methylenetetrahydrofolate dehydrogenase (nadp+ dependent) 1-like LRRK1 - leucine-rich repeat kinase 1 NLK - nemo-like kinase UBE2QL1 - ubiquitin-conjugating enzyme e2q family-like 1 MAGI1 - membrane associated guanylate kinase, ww and pdz domain containing 1 NUAK1 - nuak family, snf1-like kinase, 1 PAK1 - p21 protein (cdc42/rac)-activated kinase 1 DGKZ - diacylglycerol kinase, zeta CYP2E1 - cytochrome p450, family 2, subfamily e, polypeptide 1 ABCB8 - atp-binding cassette, sub-family b (mdr/tap), member 8 CHD5 - chromodomain helicase dna binding protein 5 PDPK1 - 3-phosphoinositide dependent protein kinase-1 FES - feline sarcoma oncogene DHX38 - deah (asp-glu-ala-his) box polypeptide 38 DCLK1 - doublecortin-like kinase 1 DAPK3 - death-associated protein kinase 3 CDK5 - cyclin-dependent kinase 5 ASNA1 - arsa arsenite transporter, atp-binding, homolog 1 (bacterial) CDK9 - cyclin-dependent kinase 9 DGKA - diacylglycerol kinase, alpha 80kda MET - met proto-oncogene MAP3K13 - mitogen-activated protein kinase kinase kinase 13 ASS1 - argininosuccinate synthase 1 MAPK12 - mitogen-activated protein kinase 12 TNNI3K - tnni3 interacting kinase SARS - seryl-trna synthetase PDE2A - phosphodiesterase 2a, cgmp-stimulated CYP26A1 - cytochrome p450, family 26, subfamily a, polypeptide 1 CHRNA7 - cholinergic receptor, nicotinic, alpha 7 (neuronal) RTBDN - retbindin NOS2 - nitric oxide synthase 2, inducible ATP12A - atpase, h+/k+ transporting, nongastric, alpha polypeptide ATP1A1 - atpase, na+/k+ transporting, alpha 1 polypeptide P2RX6 - purinergic receptor p2x, ligand-gated ion channel, 6 CDK14 - cyclin-dependent kinase 14 NME1 - nme/nm23 nucleoside diphosphate kinase 1 CHRM3 - cholinergic receptor, muscarinic 3 GOT1 - glutamic-oxaloacetic transaminase 1, soluble STK32C - serine/threonine kinase 32c MORC2 - morc family cw-type zinc finger 2 MYO19 - myosin xix DRD1 - dopamine receptor d1 CHKB - choline kinase beta ABCB9 - atp-binding cassette, sub-family b (mdr/tap), member 9 SKIV2L - superkiller viralicidic activity 2-like (s. cerevisiae) ATP6V1B1 - atpase, h+ transporting, lysosomal 56/58kda, v1 subunit b1 PIP5K1B - phosphatidylinositol-4-phosphate 5-kinase, type i, beta MATK - megakaryocyte-associated tyrosine kinase STYK1 - serine/threonine/tyrosine kinase 1 PIF1 - pif1 5'-to-3' dna helicase PFKP - phosphofructokinase, platelet ATP2B4 - atpase, ca++ transporting, plasma membrane 4 PFAS - phosphoribosylformylglycinamidine synthase ATP2B3 - atpase, ca++ transporting, plasma membrane 3 EHD2 - eh-domain containing 2 INO80 - ino80 complex subunit MAP3K6 - mitogen-activated protein kinase kinase kinase 6 EHD3 - eh-domain containing 3 DDX56 - dead (asp-glu-ala-asp) box helicase 56 ATP8B1 - atpase, aminophospholipid transporter, class i, type 8b, member 1 GRK6 - g protein-coupled receptor kinase 6 PANK4 - pantothenate kinase 4 PAPSS2 - 3'-phosphoadenosine 5'-phosphosulfate synthase 2 EGLN2 - egl-9 family hypoxia-inducible factor 2 PI4KA - phosphatidylinositol 4-kinase, catalytic, alpha TESK1 - testis-specific kinase 1 RARB - retinoic acid receptor, beta MYH7B - myosin, heavy chain 7b, cardiac muscle, beta TPK1 - thiamin pyrophosphokinase 1 KIF1A - kinesin family member 1a FLAD1 - flavin adenine dinucleotide synthetase 1 BRSK2 - br serine/threonine kinase 2 CLK2 - cdc-like kinase 2 LTK - leukocyte receptor tyrosine kinase TWF2 - twinfilin actin-binding protein 2 ABCC10 - atp-binding cassette, sub-family c (cftr/mrp), member 10 CLCN7 - chloride channel, voltage-sensitive 7 KALRN - kalirin, rhogef kinase PHKG2 - phosphorylase kinase, gamma 2 (testis) ATAD3B - atpase family, aaa domain containing 3b RPS6KA4 - ribosomal protein s6 kinase, 90kda, polypeptide 4 DHX16 - deah (asp-glu-ala-his) box polypeptide 16 PPIP5K1 - diphosphoinositol pentakisphosphate kinase 1 CKMT1B - creatine kinase, mitochondrial 1b PIK3CD - phosphatidylinositol-4,5-bisphosphate 3-kinase, catalytic subunit delta PIM1 - pim-1 oncogene UBE2D4 - ubiquitin-conjugating enzyme e2d 4 (putative) TARS2 - threonyl-trna synthetase 2, mitochondrial (putative) NAV2 - neuron navigator 2 GSK3A - glycogen synthase kinase 3 alpha |
| GO:0030554 | adenyl nucleotide binding | 6.02E-6 | 6.78E-4 | 1.39 (16750,1392,1717,199) | TRIB3 - tribbles homolog 3 (drosophila) RFC2 - replication factor c (activator 1) 2, 40kda LIMK1 - lim domain kinase 1 BCKDK - branched chain ketoacid dehydrogenase kinase DHX30 - deah (asp-glu-ala-his) box helicase 30 ABCA4 - atp-binding cassette, sub-family a (abc1), member 4 SPG7 - spastic paraplegia 7 (pure and complicated autosomal recessive) RET - ret proto-oncogene CDKL1 - cyclin-dependent kinase-like 1 (cdc2-related kinase) BRSK1 - br serine/threonine kinase 1 CCT5 - chaperonin containing tcp1, subunit 5 (epsilon) PIP5KL1 - phosphatidylinositol-4-phosphate 5-kinase-like 1 SPHK2 - sphingosine kinase 2 UBE2J2 - ubiquitin-conjugating enzyme e2, j2 MAGI3 - membrane associated guanylate kinase, ww and pdz domain containing 3 STK19 - serine/threonine kinase 19 RIMKLA - ribosomal modification protein rimk-like family member a NME6 - nme/nm23 nucleoside diphosphate kinase 6 MAST1 - microtubule associated serine/threonine kinase 1 MYO5B - myosin vb MAST3 - microtubule associated serine/threonine kinase 3 ADCY6 - adenylate cyclase 6 MYO16 - myosin xvi PIM3 - pim-3 oncogene ADCY2 - adenylate cyclase 2 (brain) SNRNP200 - small nuclear ribonucleoprotein 200kda (u5) FIGNL2 - fidgetin-like 2 PNCK - pregnancy up-regulated non-ubiquitously expressed cam kinase NRBP1 - nuclear receptor binding protein 1 KIF21B - kinesin family member 21b TRPM4 - transient receptor potential cation channel, subfamily m, member 4 MVD - mevalonate (diphospho) decarboxylase PGS1 - phosphatidylglycerophosphate synthase 1 PAK6 - p21 protein (cdc42/rac)-activated kinase 6 RUVBL2 - ruvb-like 2 (e. coli) TRAP1 - tnf receptor-associated protein 1 PRKAA2 - protein kinase, amp-activated, alpha 2 catalytic subunit MYO15A - myosin xva NAT6 - n-acetyltransferase 6 (gcn5-related) CAMK1D - calcium/calmodulin-dependent protein kinase id CAMK2G - calcium/calmodulin-dependent protein kinase ii gamma ADRBK2 - adrenergic, beta, receptor kinase 2 AGK - acylglycerol kinase KIF3C - kinesin family member 3c ADRBK1 - adrenergic, beta, receptor kinase 1 CAMK2A - calcium/calmodulin-dependent protein kinase ii alpha KIF5A - kinesin family member 5a ABCC8 - atp-binding cassette, sub-family c (cftr/mrp), member 8 TIMM44 - translocase of inner mitochondrial membrane 44 homolog (yeast) KHK - ketohexokinase (fructokinase) PTK2B - protein tyrosine kinase 2 beta CIT - citron (rho-interacting, serine/threonine kinase 21) PPP5C - protein phosphatase 5, catalytic subunit CUL9 - cullin 9 CARS - cysteinyl-trna synthetase FARSA - phenylalanyl-trna synthetase, alpha subunit AACS - acetoacetyl-coa synthetase CAD - carbamoyl-phosphate synthetase 2, aspartate transcarbamylase, and dihydroorotase PINK1 - pten induced putative kinase 1 HK1 - hexokinase 1 XRCC6 - x-ray repair complementing defective repair in chinese hamster cells 6 UBE2E3 - ubiquitin-conjugating enzyme e2e 3 ADCK2 - aarf domain containing kinase 2 KIF25 - kinesin family member 25 KIFC2 - kinesin family member c2 DDX51 - dead (asp-glu-ala-asp) box polypeptide 51 CAMKK1 - calcium/calmodulin-dependent protein kinase kinase 1, alpha VARS - valyl-trna synthetase STK25 - serine/threonine kinase 25 RAPGEF4 - rap guanine nucleotide exchange factor (gef) 4 STK11 - serine/threonine kinase 11 TTLL12 - tubulin tyrosine ligase-like family, member 12 LONP1 - lon peptidase 1, mitochondrial GAK - cyclin g associated kinase PRPS2 - phosphoribosyl pyrophosphate synthetase 2 PRPS1 - phosphoribosyl pyrophosphate synthetase 1 PRKY - protein kinase, y-linked, pseudogene CAMK1G - calcium/calmodulin-dependent protein kinase ig XYLB - xylulokinase homolog (h. influenzae) UCKL1 - uridine-cytidine kinase 1-like 1 ACAD9 - acyl-coa dehydrogenase family, member 9 TYRO3 - tyro3 protein tyrosine kinase ITPKA - inositol-trisphosphate 3-kinase a TYK2 - tyrosine kinase 2 MAP2K1 - mitogen-activated protein kinase kinase 1 DHX35 - deah (asp-glu-ala-his) box polypeptide 35 MAP2K2 - mitogen-activated protein kinase kinase 2 MAPK10 - mitogen-activated protein kinase 10 MAPK11 - mitogen-activated protein kinase 11 EPHA3 - eph receptor a3 KCNJ11 - potassium inwardly-rectifying channel, subfamily j, member 11 PKDCC - protein kinase domain containing, cytoplasmic MAPK3 - mitogen-activated protein kinase 3 PRKG2 - protein kinase, cgmp-dependent, type ii EPHB3 - eph receptor b3 EPHA5 - eph receptor a5 EPHA4 - eph receptor a4 KCNJ8 - potassium inwardly-rectifying channel, subfamily j, member 8 CSNK1E - casein kinase 1, epsilon ATP13A1 - atpase type 13a1 PRKCE - protein kinase c, epsilon EPHB6 - eph receptor b6 PRKCB - protein kinase c, beta SLC27A2 - solute carrier family 27 (fatty acid transporter), member 2 ADCK1 - aarf domain containing kinase 1 UBE2O - ubiquitin-conjugating enzyme e2o PAK7 - p21 protein (cdc42/rac)-activated kinase 7 ERCC2 - excision repair cross-complementing rodent repair deficiency, complementation group 2 CDC6 - cell division cycle 6 MTPAP - mitochondrial poly(a) polymerase DHX8 - deah (asp-glu-ala-his) box polypeptide 8 FLT3 - fms-related tyrosine kinase 3 MAP3K9 - mitogen-activated protein kinase kinase kinase 9 MTHFD1L - methylenetetrahydrofolate dehydrogenase (nadp+ dependent) 1-like LRRK1 - leucine-rich repeat kinase 1 NLK - nemo-like kinase UBE2QL1 - ubiquitin-conjugating enzyme e2q family-like 1 MAGI1 - membrane associated guanylate kinase, ww and pdz domain containing 1 NUAK1 - nuak family, snf1-like kinase, 1 PAK1 - p21 protein (cdc42/rac)-activated kinase 1 DGKZ - diacylglycerol kinase, zeta ABCB8 - atp-binding cassette, sub-family b (mdr/tap), member 8 CHD5 - chromodomain helicase dna binding protein 5 PDPK1 - 3-phosphoinositide dependent protein kinase-1 FES - feline sarcoma oncogene DHX38 - deah (asp-glu-ala-his) box polypeptide 38 DCLK1 - doublecortin-like kinase 1 SCN8A - sodium channel, voltage gated, type viii, alpha subunit CDK5 - cyclin-dependent kinase 5 ASNA1 - arsa arsenite transporter, atp-binding, homolog 1 (bacterial) DAPK3 - death-associated protein kinase 3 CDK9 - cyclin-dependent kinase 9 DGKA - diacylglycerol kinase, alpha 80kda MET - met proto-oncogene MAP3K13 - mitogen-activated protein kinase kinase kinase 13 ASS1 - argininosuccinate synthase 1 MAPK12 - mitogen-activated protein kinase 12 SARS - seryl-trna synthetase TNNI3K - tnni3 interacting kinase PDE2A - phosphodiesterase 2a, cgmp-stimulated PDE4A - phosphodiesterase 4a, camp-specific ATP12A - atpase, h+/k+ transporting, nongastric, alpha polypeptide ATP1A1 - atpase, na+/k+ transporting, alpha 1 polypeptide P2RX6 - purinergic receptor p2x, ligand-gated ion channel, 6 CDK14 - cyclin-dependent kinase 14 NME1 - nme/nm23 nucleoside diphosphate kinase 1 ATP1A4 - atpase, na+/k+ transporting, alpha 4 polypeptide STK32C - serine/threonine kinase 32c MORC2 - morc family cw-type zinc finger 2 MYO19 - myosin xix CHKB - choline kinase beta MAP4K2 - mitogen-activated protein kinase kinase kinase kinase 2 ABCB9 - atp-binding cassette, sub-family b (mdr/tap), member 9 SKIV2L - superkiller viralicidic activity 2-like (s. cerevisiae) HCN1 - hyperpolarization activated cyclic nucleotide-gated potassium channel 1 ATP6V1B1 - atpase, h+ transporting, lysosomal 56/58kda, v1 subunit b1 PIP5K1B - phosphatidylinositol-4-phosphate 5-kinase, type i, beta MATK - megakaryocyte-associated tyrosine kinase STYK1 - serine/threonine/tyrosine kinase 1 PIF1 - pif1 5'-to-3' dna helicase PFKP - phosphofructokinase, platelet ATP2B4 - atpase, ca++ transporting, plasma membrane 4 PFAS - phosphoribosylformylglycinamidine synthase ATP2B3 - atpase, ca++ transporting, plasma membrane 3 EHD2 - eh-domain containing 2 INO80 - ino80 complex subunit MAP3K6 - mitogen-activated protein kinase kinase kinase 6 EHD3 - eh-domain containing 3 DDX56 - dead (asp-glu-ala-asp) box helicase 56 ATP8B1 - atpase, aminophospholipid transporter, class i, type 8b, member 1 GRK6 - g protein-coupled receptor kinase 6 PANK4 - pantothenate kinase 4 PAPSS2 - 3'-phosphoadenosine 5'-phosphosulfate synthase 2 PI4KA - phosphatidylinositol 4-kinase, catalytic, alpha TESK1 - testis-specific kinase 1 TPK1 - thiamin pyrophosphokinase 1 MYH7B - myosin, heavy chain 7b, cardiac muscle, beta KIF1A - kinesin family member 1a FLAD1 - flavin adenine dinucleotide synthetase 1 BRSK2 - br serine/threonine kinase 2 CLK2 - cdc-like kinase 2 LTK - leukocyte receptor tyrosine kinase TWF2 - twinfilin actin-binding protein 2 ABCC10 - atp-binding cassette, sub-family c (cftr/mrp), member 10 CLCN7 - chloride channel, voltage-sensitive 7 ACOT7 - acyl-coa thioesterase 7 KALRN - kalirin, rhogef kinase PHKG2 - phosphorylase kinase, gamma 2 (testis) ATAD3B - atpase family, aaa domain containing 3b RPS6KA4 - ribosomal protein s6 kinase, 90kda, polypeptide 4 DHX16 - deah (asp-glu-ala-his) box polypeptide 16 PPIP5K1 - diphosphoinositol pentakisphosphate kinase 1 CKMT1B - creatine kinase, mitochondrial 1b PIK3CD - phosphatidylinositol-4,5-bisphosphate 3-kinase, catalytic subunit delta PIM1 - pim-1 oncogene UBE2D4 - ubiquitin-conjugating enzyme e2d 4 (putative) TARS2 - threonyl-trna synthetase 2, mitochondrial (putative) GSK3A - glycogen synthase kinase 3 alpha NAV2 - neuron navigator 2 |
| GO:0005242 | inward rectifier potassium channel activity | 7.15E-6 | 7.85E-4 | 16.58 (16750,19,319,6) | KCNJ11 - potassium inwardly-rectifying channel, subfamily j, member 11 KCNJ4 - potassium inwardly-rectifying channel, subfamily j, member 4 KCNJ3 - potassium inwardly-rectifying channel, subfamily j, member 3 KCNJ9 - potassium inwardly-rectifying channel, subfamily j, member 9 KCNJ8 - potassium inwardly-rectifying channel, subfamily j, member 8 KCNJ6 - potassium inwardly-rectifying channel, subfamily j, member 6 |
| GO:0000166 | nucleotide binding | 7.54E-6 | 8.08E-4 | 1.33 (16750,1908,1722,260) | TRIB3 - tribbles homolog 3 (drosophila) RFC2 - replication factor c (activator 1) 2, 40kda AGAP3 - arfgap with gtpase domain, ankyrin repeat and ph domain 3 LIMK1 - lim domain kinase 1 BCKDK - branched chain ketoacid dehydrogenase kinase DHX30 - deah (asp-glu-ala-his) box helicase 30 SEPT5 - septin 5 DHRS11 - dehydrogenase/reductase (sdr family) member 11 ABCA4 - atp-binding cassette, sub-family a (abc1), member 4 SPG7 - spastic paraplegia 7 (pure and complicated autosomal recessive) RET - ret proto-oncogene CDKL1 - cyclin-dependent kinase-like 1 (cdc2-related kinase) TUBA4A - tubulin, alpha 4a TUBA3C - tubulin, alpha 3c RAB3D - rab3d, member ras oncogene family BRSK1 - br serine/threonine kinase 1 CCT5 - chaperonin containing tcp1, subunit 5 (epsilon) PIP5KL1 - phosphatidylinositol-4-phosphate 5-kinase-like 1 SPHK2 - sphingosine kinase 2 RAB37 - rab37, member ras oncogene family UBE2J2 - ubiquitin-conjugating enzyme e2, j2 MAGI3 - membrane associated guanylate kinase, ww and pdz domain containing 3 TUBA4B - tubulin, alpha 4b (pseudogene) STK19 - serine/threonine kinase 19 RAB40C - rab40c, member ras oncogene family NME6 - nme/nm23 nucleoside diphosphate kinase 6 RIMKLA - ribosomal modification protein rimk-like family member a MYO5B - myosin vb MAST1 - microtubule associated serine/threonine kinase 1 EEF1A2 - eukaryotic translation elongation factor 1 alpha 2 MAST3 - microtubule associated serine/threonine kinase 3 ADCY6 - adenylate cyclase 6 MYO16 - myosin xvi PIM3 - pim-3 oncogene NT5C1A - 5'-nucleotidase, cytosolic ia ADCY2 - adenylate cyclase 2 (brain) SNRNP200 - small nuclear ribonucleoprotein 200kda (u5) DIRAS2 - diras family, gtp-binding ras-like 2 NT5M - 5',3'-nucleotidase, mitochondrial FIGNL2 - fidgetin-like 2 PNCK - pregnancy up-regulated non-ubiquitously expressed cam kinase NRBP1 - nuclear receptor binding protein 1 KIF21B - kinesin family member 21b MVD - mevalonate (diphospho) decarboxylase TRPM4 - transient receptor potential cation channel, subfamily m, member 4 GNA15 - guanine nucleotide binding protein (g protein), alpha 15 (gq class) GMPPB - gdp-mannose pyrophosphorylase b POLD1 - polymerase (dna directed), delta 1, catalytic subunit GNA11 - guanine nucleotide binding protein (g protein), alpha 11 (gq class) ARL4A - adp-ribosylation factor-like 4a ARL4C - adp-ribosylation factor-like 4c PGS1 - phosphatidylglycerophosphate synthase 1 PAK6 - p21 protein (cdc42/rac)-activated kinase 6 TRAP1 - tnf receptor-associated protein 1 RUVBL2 - ruvb-like 2 (e. coli) PRKAA2 - protein kinase, amp-activated, alpha 2 catalytic subunit MYO15A - myosin xva NAT6 - n-acetyltransferase 6 (gcn5-related) CAMK1D - calcium/calmodulin-dependent protein kinase id KIF3C - kinesin family member 3c CAMK2G - calcium/calmodulin-dependent protein kinase ii gamma ADRBK2 - adrenergic, beta, receptor kinase 2 AGK - acylglycerol kinase ADRBK1 - adrenergic, beta, receptor kinase 1 KIF5A - kinesin family member 5a CAMK2A - calcium/calmodulin-dependent protein kinase ii alpha ABCC8 - atp-binding cassette, sub-family c (cftr/mrp), member 8 KHK - ketohexokinase (fructokinase) TIMM44 - translocase of inner mitochondrial membrane 44 homolog (yeast) PTK2B - protein tyrosine kinase 2 beta CIT - citron (rho-interacting, serine/threonine kinase 21) PPP5C - protein phosphatase 5, catalytic subunit CUL9 - cullin 9 CARS - cysteinyl-trna synthetase FARSA - phenylalanyl-trna synthetase, alpha subunit FASN - fatty acid synthase AACS - acetoacetyl-coa synthetase CAD - carbamoyl-phosphate synthetase 2, aspartate transcarbamylase, and dihydroorotase PINK1 - pten induced putative kinase 1 HK1 - hexokinase 1 XRCC6 - x-ray repair complementing defective repair in chinese hamster cells 6 UBE2E3 - ubiquitin-conjugating enzyme e2e 3 ADCK2 - aarf domain containing kinase 2 KIF25 - kinesin family member 25 KIFC2 - kinesin family member c2 DDX51 - dead (asp-glu-ala-asp) box polypeptide 51 CAMKK1 - calcium/calmodulin-dependent protein kinase kinase 1, alpha VARS - valyl-trna synthetase STK25 - serine/threonine kinase 25 RAPGEF4 - rap guanine nucleotide exchange factor (gef) 4 STK11 - serine/threonine kinase 11 RABL6 - rab, member ras oncogene family-like 6 TTLL12 - tubulin tyrosine ligase-like family, member 12 LONP1 - lon peptidase 1, mitochondrial EEFSEC - eukaryotic elongation factor, selenocysteine-trna-specific TUBA1B - tubulin, alpha 1b PRPS2 - phosphoribosyl pyrophosphate synthetase 2 GAK - cyclin g associated kinase PRPS1 - phosphoribosyl pyrophosphate synthetase 1 PRODH - proline dehydrogenase (oxidase) 1 RAB26 - rab26, member ras oncogene family PRKY - protein kinase, y-linked, pseudogene CAMK1G - calcium/calmodulin-dependent protein kinase ig XYLB - xylulokinase homolog (h. influenzae) ACAD9 - acyl-coa dehydrogenase family, member 9 UCKL1 - uridine-cytidine kinase 1-like 1 TYRO3 - tyro3 protein tyrosine kinase ITPKA - inositol-trisphosphate 3-kinase a TYK2 - tyrosine kinase 2 MAP2K1 - mitogen-activated protein kinase kinase 1 DHX35 - deah (asp-glu-ala-his) box polypeptide 35 MAP2K2 - mitogen-activated protein kinase kinase 2 MAPK10 - mitogen-activated protein kinase 10 MAPK11 - mitogen-activated protein kinase 11 EPHA3 - eph receptor a3 KCNJ11 - potassium inwardly-rectifying channel, subfamily j, member 11 PKDCC - protein kinase domain containing, cytoplasmic MAPK3 - mitogen-activated protein kinase 3 PRKG2 - protein kinase, cgmp-dependent, type ii EPHB3 - eph receptor b3 EPHA5 - eph receptor a5 EPHA4 - eph receptor a4 KCNJ8 - potassium inwardly-rectifying channel, subfamily j, member 8 CSNK1E - casein kinase 1, epsilon ATP13A1 - atpase type 13a1 PRKCE - protein kinase c, epsilon EPHB6 - eph receptor b6 PRKCB - protein kinase c, beta SLC27A2 - solute carrier family 27 (fatty acid transporter), member 2 SLC27A4 - solute carrier family 27 (fatty acid transporter), member 4 ADCK1 - aarf domain containing kinase 1 UBE2O - ubiquitin-conjugating enzyme e2o ERCC2 - excision repair cross-complementing rodent repair deficiency, complementation group 2 PAK7 - p21 protein (cdc42/rac)-activated kinase 7 CDC6 - cell division cycle 6 MTPAP - mitochondrial poly(a) polymerase DHX8 - deah (asp-glu-ala-his) box polypeptide 8 TUBA3D - tubulin, alpha 3d FLT3 - fms-related tyrosine kinase 3 MAP3K9 - mitogen-activated protein kinase kinase kinase 9 ANXA6 - annexin a6 TUBA8 - tubulin, alpha 8 RAB15 - rab15, member ras oncogene family MTHFD1L - methylenetetrahydrofolate dehydrogenase (nadp+ dependent) 1-like LRRK1 - leucine-rich repeat kinase 1 NLK - nemo-like kinase UBE2QL1 - ubiquitin-conjugating enzyme e2q family-like 1 MAGI1 - membrane associated guanylate kinase, ww and pdz domain containing 1 NUAK1 - nuak family, snf1-like kinase, 1 PAK1 - p21 protein (cdc42/rac)-activated kinase 1 RASL10A - ras-like, family 10, member a DGKZ - diacylglycerol kinase, zeta TUBA1A - tubulin, alpha 1a ABCB8 - atp-binding cassette, sub-family b (mdr/tap), member 8 CHD5 - chromodomain helicase dna binding protein 5 PDPK1 - 3-phosphoinositide dependent protein kinase-1 RAB24 - rab24, member ras oncogene family FES - feline sarcoma oncogene DHX38 - deah (asp-glu-ala-his) box polypeptide 38 DCLK1 - doublecortin-like kinase 1 SCN8A - sodium channel, voltage gated, type viii, alpha subunit KMO - kynurenine 3-monooxygenase (kynurenine 3-hydroxylase) CDK5 - cyclin-dependent kinase 5 ASNA1 - arsa arsenite transporter, atp-binding, homolog 1 (bacterial) DAPK3 - death-associated protein kinase 3 CDK9 - cyclin-dependent kinase 9 RND1 - rho family gtpase 1 DGKA - diacylglycerol kinase, alpha 80kda MET - met proto-oncogene MAP3K13 - mitogen-activated protein kinase kinase kinase 13 ARL9 - adp-ribosylation factor-like 9 ASS1 - argininosuccinate synthase 1 MAPK12 - mitogen-activated protein kinase 12 TNNI3K - tnni3 interacting kinase SARS - seryl-trna synthetase PDE2A - phosphodiesterase 2a, cgmp-stimulated MBD3 - methyl-cpg binding domain protein 3 TUBB8 - tubulin, beta 8 class viii PDE4A - phosphodiesterase 4a, camp-specific GPN2 - gpn-loop gtpase 2 GTPBP2 - gtp binding protein 2 RHEBL1 - ras homolog enriched in brain like 1 NOS2 - nitric oxide synthase 2, inducible GNL1 - guanine nucleotide binding protein-like 1 ATP12A - atpase, h+/k+ transporting, nongastric, alpha polypeptide P2RX6 - purinergic receptor p2x, ligand-gated ion channel, 6 ATP1A1 - atpase, na+/k+ transporting, alpha 1 polypeptide CDK14 - cyclin-dependent kinase 14 NME1 - nme/nm23 nucleoside diphosphate kinase 1 PGD - phosphogluconate dehydrogenase ATP1A4 - atpase, na+/k+ transporting, alpha 4 polypeptide STK32C - serine/threonine kinase 32c RAB6B - rab6b, member ras oncogene family MORC2 - morc family cw-type zinc finger 2 MYO19 - myosin xix SIRT6 - sirtuin 6 SIRT7 - sirtuin 7 CHKB - choline kinase beta SIRT4 - sirtuin 4 CNGB3 - cyclic nucleotide gated channel beta 3 MAP4K2 - mitogen-activated protein kinase kinase kinase kinase 2 ABCB9 - atp-binding cassette, sub-family b (mdr/tap), member 9 RAB3A - rab3a, member ras oncogene family ARL10 - adp-ribosylation factor-like 10 SKIV2L - superkiller viralicidic activity 2-like (s. cerevisiae) HCN1 - hyperpolarization activated cyclic nucleotide-gated potassium channel 1 ATP6V1B1 - atpase, h+ transporting, lysosomal 56/58kda, v1 subunit b1 PIP5K1B - phosphatidylinositol-4-phosphate 5-kinase, type i, beta MATK - megakaryocyte-associated tyrosine kinase STYK1 - serine/threonine/tyrosine kinase 1 PIF1 - pif1 5'-to-3' dna helicase TUBG2 - tubulin, gamma 2 RAC2 - ras-related c3 botulinum toxin substrate 2 (rho family, small gtp binding protein rac2) PFKP - phosphofructokinase, platelet ATP2B4 - atpase, ca++ transporting, plasma membrane 4 PFAS - phosphoribosylformylglycinamidine synthase ATP2B3 - atpase, ca++ transporting, plasma membrane 3 EHD2 - eh-domain containing 2 MAP3K6 - mitogen-activated protein kinase kinase kinase 6 INO80 - ino80 complex subunit EHD3 - eh-domain containing 3 DDX56 - dead (asp-glu-ala-asp) box helicase 56 RAB27B - rab27b, member ras oncogene family ATP8B1 - atpase, aminophospholipid transporter, class i, type 8b, member 1 GRK6 - g protein-coupled receptor kinase 6 PAPSS2 - 3'-phosphoadenosine 5'-phosphosulfate synthase 2 PANK4 - pantothenate kinase 4 MICAL2 - microtubule associated monooxygenase, calponin and lim domain containing 2 PI4KA - phosphatidylinositol 4-kinase, catalytic, alpha TESK1 - testis-specific kinase 1 DHCR7 - 7-dehydrocholesterol reductase MYH7B - myosin, heavy chain 7b, cardiac muscle, beta TPK1 - thiamin pyrophosphokinase 1 RASD1 - ras, dexamethasone-induced 1 KIF1A - kinesin family member 1a FLAD1 - flavin adenine dinucleotide synthetase 1 BRSK2 - br serine/threonine kinase 2 LTK - leukocyte receptor tyrosine kinase CLK2 - cdc-like kinase 2 TWF2 - twinfilin actin-binding protein 2 RHOV - ras homolog family member v ACAD8 - acyl-coa dehydrogenase family, member 8 ABCC10 - atp-binding cassette, sub-family c (cftr/mrp), member 10 CLCN7 - chloride channel, voltage-sensitive 7 ACOT7 - acyl-coa thioesterase 7 KALRN - kalirin, rhogef kinase ATAD3B - atpase family, aaa domain containing 3b PHKG2 - phosphorylase kinase, gamma 2 (testis) RPS6KA4 - ribosomal protein s6 kinase, 90kda, polypeptide 4 DHX16 - deah (asp-glu-ala-his) box polypeptide 16 PPIP5K1 - diphosphoinositol pentakisphosphate kinase 1 CKMT1B - creatine kinase, mitochondrial 1b PIK3CD - phosphatidylinositol-4,5-bisphosphate 3-kinase, catalytic subunit delta PIM1 - pim-1 oncogene DNM1 - dynamin 1 UBE2D4 - ubiquitin-conjugating enzyme e2d 4 (putative) TARS2 - threonyl-trna synthetase 2, mitochondrial (putative) NAV2 - neuron navigator 2 GSK3A - glycogen synthase kinase 3 alpha RHOF - ras homolog family member f (in filopodia) |
| GO:1901265 | nucleoside phosphate binding | 8.33E-6 | 8.73E-4 | 1.32 (16750,1909,1722,260) | TRIB3 - tribbles homolog 3 (drosophila) RFC2 - replication factor c (activator 1) 2, 40kda AGAP3 - arfgap with gtpase domain, ankyrin repeat and ph domain 3 LIMK1 - lim domain kinase 1 BCKDK - branched chain ketoacid dehydrogenase kinase DHX30 - deah (asp-glu-ala-his) box helicase 30 SEPT5 - septin 5 DHRS11 - dehydrogenase/reductase (sdr family) member 11 ABCA4 - atp-binding cassette, sub-family a (abc1), member 4 SPG7 - spastic paraplegia 7 (pure and complicated autosomal recessive) RET - ret proto-oncogene CDKL1 - cyclin-dependent kinase-like 1 (cdc2-related kinase) TUBA4A - tubulin, alpha 4a TUBA3C - tubulin, alpha 3c RAB3D - rab3d, member ras oncogene family BRSK1 - br serine/threonine kinase 1 CCT5 - chaperonin containing tcp1, subunit 5 (epsilon) PIP5KL1 - phosphatidylinositol-4-phosphate 5-kinase-like 1 SPHK2 - sphingosine kinase 2 RAB37 - rab37, member ras oncogene family UBE2J2 - ubiquitin-conjugating enzyme e2, j2 MAGI3 - membrane associated guanylate kinase, ww and pdz domain containing 3 TUBA4B - tubulin, alpha 4b (pseudogene) STK19 - serine/threonine kinase 19 RAB40C - rab40c, member ras oncogene family NME6 - nme/nm23 nucleoside diphosphate kinase 6 RIMKLA - ribosomal modification protein rimk-like family member a MYO5B - myosin vb MAST1 - microtubule associated serine/threonine kinase 1 EEF1A2 - eukaryotic translation elongation factor 1 alpha 2 MAST3 - microtubule associated serine/threonine kinase 3 ADCY6 - adenylate cyclase 6 MYO16 - myosin xvi PIM3 - pim-3 oncogene NT5C1A - 5'-nucleotidase, cytosolic ia ADCY2 - adenylate cyclase 2 (brain) SNRNP200 - small nuclear ribonucleoprotein 200kda (u5) DIRAS2 - diras family, gtp-binding ras-like 2 NT5M - 5',3'-nucleotidase, mitochondrial FIGNL2 - fidgetin-like 2 PNCK - pregnancy up-regulated non-ubiquitously expressed cam kinase NRBP1 - nuclear receptor binding protein 1 KIF21B - kinesin family member 21b MVD - mevalonate (diphospho) decarboxylase TRPM4 - transient receptor potential cation channel, subfamily m, member 4 GNA15 - guanine nucleotide binding protein (g protein), alpha 15 (gq class) GMPPB - gdp-mannose pyrophosphorylase b POLD1 - polymerase (dna directed), delta 1, catalytic subunit GNA11 - guanine nucleotide binding protein (g protein), alpha 11 (gq class) ARL4A - adp-ribosylation factor-like 4a ARL4C - adp-ribosylation factor-like 4c PGS1 - phosphatidylglycerophosphate synthase 1 PAK6 - p21 protein (cdc42/rac)-activated kinase 6 TRAP1 - tnf receptor-associated protein 1 RUVBL2 - ruvb-like 2 (e. coli) PRKAA2 - protein kinase, amp-activated, alpha 2 catalytic subunit MYO15A - myosin xva NAT6 - n-acetyltransferase 6 (gcn5-related) CAMK1D - calcium/calmodulin-dependent protein kinase id KIF3C - kinesin family member 3c CAMK2G - calcium/calmodulin-dependent protein kinase ii gamma AGK - acylglycerol kinase ADRBK2 - adrenergic, beta, receptor kinase 2 ADRBK1 - adrenergic, beta, receptor kinase 1 KIF5A - kinesin family member 5a CAMK2A - calcium/calmodulin-dependent protein kinase ii alpha ABCC8 - atp-binding cassette, sub-family c (cftr/mrp), member 8 KHK - ketohexokinase (fructokinase) TIMM44 - translocase of inner mitochondrial membrane 44 homolog (yeast) PTK2B - protein tyrosine kinase 2 beta CIT - citron (rho-interacting, serine/threonine kinase 21) PPP5C - protein phosphatase 5, catalytic subunit CUL9 - cullin 9 CARS - cysteinyl-trna synthetase FARSA - phenylalanyl-trna synthetase, alpha subunit FASN - fatty acid synthase AACS - acetoacetyl-coa synthetase CAD - carbamoyl-phosphate synthetase 2, aspartate transcarbamylase, and dihydroorotase PINK1 - pten induced putative kinase 1 HK1 - hexokinase 1 XRCC6 - x-ray repair complementing defective repair in chinese hamster cells 6 UBE2E3 - ubiquitin-conjugating enzyme e2e 3 ADCK2 - aarf domain containing kinase 2 KIF25 - kinesin family member 25 KIFC2 - kinesin family member c2 DDX51 - dead (asp-glu-ala-asp) box polypeptide 51 CAMKK1 - calcium/calmodulin-dependent protein kinase kinase 1, alpha VARS - valyl-trna synthetase STK25 - serine/threonine kinase 25 RAPGEF4 - rap guanine nucleotide exchange factor (gef) 4 STK11 - serine/threonine kinase 11 RABL6 - rab, member ras oncogene family-like 6 TTLL12 - tubulin tyrosine ligase-like family, member 12 LONP1 - lon peptidase 1, mitochondrial EEFSEC - eukaryotic elongation factor, selenocysteine-trna-specific TUBA1B - tubulin, alpha 1b PRPS2 - phosphoribosyl pyrophosphate synthetase 2 GAK - cyclin g associated kinase PRPS1 - phosphoribosyl pyrophosphate synthetase 1 PRODH - proline dehydrogenase (oxidase) 1 RAB26 - rab26, member ras oncogene family PRKY - protein kinase, y-linked, pseudogene CAMK1G - calcium/calmodulin-dependent protein kinase ig XYLB - xylulokinase homolog (h. influenzae) ACAD9 - acyl-coa dehydrogenase family, member 9 UCKL1 - uridine-cytidine kinase 1-like 1 TYRO3 - tyro3 protein tyrosine kinase ITPKA - inositol-trisphosphate 3-kinase a TYK2 - tyrosine kinase 2 MAP2K1 - mitogen-activated protein kinase kinase 1 DHX35 - deah (asp-glu-ala-his) box polypeptide 35 MAP2K2 - mitogen-activated protein kinase kinase 2 MAPK10 - mitogen-activated protein kinase 10 MAPK11 - mitogen-activated protein kinase 11 EPHA3 - eph receptor a3 KCNJ11 - potassium inwardly-rectifying channel, subfamily j, member 11 PKDCC - protein kinase domain containing, cytoplasmic MAPK3 - mitogen-activated protein kinase 3 PRKG2 - protein kinase, cgmp-dependent, type ii EPHB3 - eph receptor b3 EPHA5 - eph receptor a5 EPHA4 - eph receptor a4 KCNJ8 - potassium inwardly-rectifying channel, subfamily j, member 8 CSNK1E - casein kinase 1, epsilon ATP13A1 - atpase type 13a1 PRKCE - protein kinase c, epsilon EPHB6 - eph receptor b6 PRKCB - protein kinase c, beta SLC27A2 - solute carrier family 27 (fatty acid transporter), member 2 SLC27A4 - solute carrier family 27 (fatty acid transporter), member 4 ADCK1 - aarf domain containing kinase 1 UBE2O - ubiquitin-conjugating enzyme e2o PAK7 - p21 protein (cdc42/rac)-activated kinase 7 ERCC2 - excision repair cross-complementing rodent repair deficiency, complementation group 2 CDC6 - cell division cycle 6 MTPAP - mitochondrial poly(a) polymerase DHX8 - deah (asp-glu-ala-his) box polypeptide 8 FLT3 - fms-related tyrosine kinase 3 TUBA3D - tubulin, alpha 3d MAP3K9 - mitogen-activated protein kinase kinase kinase 9 ANXA6 - annexin a6 TUBA8 - tubulin, alpha 8 RAB15 - rab15, member ras oncogene family MTHFD1L - methylenetetrahydrofolate dehydrogenase (nadp+ dependent) 1-like LRRK1 - leucine-rich repeat kinase 1 NLK - nemo-like kinase UBE2QL1 - ubiquitin-conjugating enzyme e2q family-like 1 MAGI1 - membrane associated guanylate kinase, ww and pdz domain containing 1 NUAK1 - nuak family, snf1-like kinase, 1 PAK1 - p21 protein (cdc42/rac)-activated kinase 1 RASL10A - ras-like, family 10, member a DGKZ - diacylglycerol kinase, zeta TUBA1A - tubulin, alpha 1a ABCB8 - atp-binding cassette, sub-family b (mdr/tap), member 8 CHD5 - chromodomain helicase dna binding protein 5 PDPK1 - 3-phosphoinositide dependent protein kinase-1 RAB24 - rab24, member ras oncogene family FES - feline sarcoma oncogene DHX38 - deah (asp-glu-ala-his) box polypeptide 38 DCLK1 - doublecortin-like kinase 1 SCN8A - sodium channel, voltage gated, type viii, alpha subunit KMO - kynurenine 3-monooxygenase (kynurenine 3-hydroxylase) CDK5 - cyclin-dependent kinase 5 ASNA1 - arsa arsenite transporter, atp-binding, homolog 1 (bacterial) DAPK3 - death-associated protein kinase 3 CDK9 - cyclin-dependent kinase 9 RND1 - rho family gtpase 1 DGKA - diacylglycerol kinase, alpha 80kda MET - met proto-oncogene MAP3K13 - mitogen-activated protein kinase kinase kinase 13 ARL9 - adp-ribosylation factor-like 9 ASS1 - argininosuccinate synthase 1 MAPK12 - mitogen-activated protein kinase 12 TNNI3K - tnni3 interacting kinase SARS - seryl-trna synthetase PDE2A - phosphodiesterase 2a, cgmp-stimulated MBD3 - methyl-cpg binding domain protein 3 TUBB8 - tubulin, beta 8 class viii PDE4A - phosphodiesterase 4a, camp-specific GPN2 - gpn-loop gtpase 2 GTPBP2 - gtp binding protein 2 RHEBL1 - ras homolog enriched in brain like 1 NOS2 - nitric oxide synthase 2, inducible GNL1 - guanine nucleotide binding protein-like 1 ATP12A - atpase, h+/k+ transporting, nongastric, alpha polypeptide P2RX6 - purinergic receptor p2x, ligand-gated ion channel, 6 ATP1A1 - atpase, na+/k+ transporting, alpha 1 polypeptide CDK14 - cyclin-dependent kinase 14 NME1 - nme/nm23 nucleoside diphosphate kinase 1 PGD - phosphogluconate dehydrogenase ATP1A4 - atpase, na+/k+ transporting, alpha 4 polypeptide STK32C - serine/threonine kinase 32c RAB6B - rab6b, member ras oncogene family MORC2 - morc family cw-type zinc finger 2 MYO19 - myosin xix SIRT6 - sirtuin 6 SIRT7 - sirtuin 7 CHKB - choline kinase beta SIRT4 - sirtuin 4 CNGB3 - cyclic nucleotide gated channel beta 3 MAP4K2 - mitogen-activated protein kinase kinase kinase kinase 2 ABCB9 - atp-binding cassette, sub-family b (mdr/tap), member 9 RAB3A - rab3a, member ras oncogene family ARL10 - adp-ribosylation factor-like 10 SKIV2L - superkiller viralicidic activity 2-like (s. cerevisiae) HCN1 - hyperpolarization activated cyclic nucleotide-gated potassium channel 1 ATP6V1B1 - atpase, h+ transporting, lysosomal 56/58kda, v1 subunit b1 PIP5K1B - phosphatidylinositol-4-phosphate 5-kinase, type i, beta MATK - megakaryocyte-associated tyrosine kinase STYK1 - serine/threonine/tyrosine kinase 1 PIF1 - pif1 5'-to-3' dna helicase TUBG2 - tubulin, gamma 2 RAC2 - ras-related c3 botulinum toxin substrate 2 (rho family, small gtp binding protein rac2) PFKP - phosphofructokinase, platelet ATP2B4 - atpase, ca++ transporting, plasma membrane 4 ATP2B3 - atpase, ca++ transporting, plasma membrane 3 PFAS - phosphoribosylformylglycinamidine synthase EHD2 - eh-domain containing 2 MAP3K6 - mitogen-activated protein kinase kinase kinase 6 INO80 - ino80 complex subunit EHD3 - eh-domain containing 3 RAB27B - rab27b, member ras oncogene family DDX56 - dead (asp-glu-ala-asp) box helicase 56 ATP8B1 - atpase, aminophospholipid transporter, class i, type 8b, member 1 GRK6 - g protein-coupled receptor kinase 6 PAPSS2 - 3'-phosphoadenosine 5'-phosphosulfate synthase 2 PANK4 - pantothenate kinase 4 MICAL2 - microtubule associated monooxygenase, calponin and lim domain containing 2 PI4KA - phosphatidylinositol 4-kinase, catalytic, alpha TESK1 - testis-specific kinase 1 DHCR7 - 7-dehydrocholesterol reductase MYH7B - myosin, heavy chain 7b, cardiac muscle, beta TPK1 - thiamin pyrophosphokinase 1 RASD1 - ras, dexamethasone-induced 1 KIF1A - kinesin family member 1a FLAD1 - flavin adenine dinucleotide synthetase 1 BRSK2 - br serine/threonine kinase 2 LTK - leukocyte receptor tyrosine kinase CLK2 - cdc-like kinase 2 TWF2 - twinfilin actin-binding protein 2 RHOV - ras homolog family member v ACAD8 - acyl-coa dehydrogenase family, member 8 ABCC10 - atp-binding cassette, sub-family c (cftr/mrp), member 10 CLCN7 - chloride channel, voltage-sensitive 7 ACOT7 - acyl-coa thioesterase 7 KALRN - kalirin, rhogef kinase ATAD3B - atpase family, aaa domain containing 3b PHKG2 - phosphorylase kinase, gamma 2 (testis) RPS6KA4 - ribosomal protein s6 kinase, 90kda, polypeptide 4 DHX16 - deah (asp-glu-ala-his) box polypeptide 16 PPIP5K1 - diphosphoinositol pentakisphosphate kinase 1 CKMT1B - creatine kinase, mitochondrial 1b PIK3CD - phosphatidylinositol-4,5-bisphosphate 3-kinase, catalytic subunit delta PIM1 - pim-1 oncogene DNM1 - dynamin 1 UBE2D4 - ubiquitin-conjugating enzyme e2d 4 (putative) TARS2 - threonyl-trna synthetase 2, mitochondrial (putative) NAV2 - neuron navigator 2 GSK3A - glycogen synthase kinase 3 alpha RHOF - ras homolog family member f (in filopodia) |
| GO:0097367 | carbohydrate derivative binding | 9.57E-6 | 9.79E-4 | 1.31 (16750,1999,1744,273) | TRIB3 - tribbles homolog 3 (drosophila) GFPT1 - glutamine--fructose-6-phosphate transaminase 1 RFC2 - replication factor c (activator 1) 2, 40kda AGAP3 - arfgap with gtpase domain, ankyrin repeat and ph domain 3 LIMK1 - lim domain kinase 1 BCKDK - branched chain ketoacid dehydrogenase kinase DHX30 - deah (asp-glu-ala-his) box helicase 30 SEPT5 - septin 5 ABCA4 - atp-binding cassette, sub-family a (abc1), member 4 SPG7 - spastic paraplegia 7 (pure and complicated autosomal recessive) HRG - histidine-rich glycoprotein TRPM2 - transient receptor potential cation channel, subfamily m, member 2 RET - ret proto-oncogene GCKR - glucokinase (hexokinase 4) regulator CDKL1 - cyclin-dependent kinase-like 1 (cdc2-related kinase) TUBA4A - tubulin, alpha 4a TUBA3C - tubulin, alpha 3c TPX2 - tpx2, microtubule-associated RAB3D - rab3d, member ras oncogene family BRSK1 - br serine/threonine kinase 1 GLA - galactosidase, alpha CCT5 - chaperonin containing tcp1, subunit 5 (epsilon) PIP5KL1 - phosphatidylinositol-4-phosphate 5-kinase-like 1 SPHK2 - sphingosine kinase 2 RAB37 - rab37, member ras oncogene family UBE2J2 - ubiquitin-conjugating enzyme e2, j2 LIPC - lipase, hepatic MAGI3 - membrane associated guanylate kinase, ww and pdz domain containing 3 STK19 - serine/threonine kinase 19 TUBA4B - tubulin, alpha 4b (pseudogene) RAB40C - rab40c, member ras oncogene family NME6 - nme/nm23 nucleoside diphosphate kinase 6 RIMKLA - ribosomal modification protein rimk-like family member a MYO5B - myosin vb MAST1 - microtubule associated serine/threonine kinase 1 EEF1A2 - eukaryotic translation elongation factor 1 alpha 2 MAST3 - microtubule associated serine/threonine kinase 3 ADCY6 - adenylate cyclase 6 MYO16 - myosin xvi PIM3 - pim-3 oncogene ADCY2 - adenylate cyclase 2 (brain) SNRNP200 - small nuclear ribonucleoprotein 200kda (u5) DIRAS2 - diras family, gtp-binding ras-like 2 FIGNL2 - fidgetin-like 2 PNCK - pregnancy up-regulated non-ubiquitously expressed cam kinase NRBP1 - nuclear receptor binding protein 1 KIF21B - kinesin family member 21b MVD - mevalonate (diphospho) decarboxylase TRPM4 - transient receptor potential cation channel, subfamily m, member 4 GNA15 - guanine nucleotide binding protein (g protein), alpha 15 (gq class) GMPPB - gdp-mannose pyrophosphorylase b GNA11 - guanine nucleotide binding protein (g protein), alpha 11 (gq class) ARL4A - adp-ribosylation factor-like 4a ADORA1 - adenosine a1 receptor ARL4C - adp-ribosylation factor-like 4c PGS1 - phosphatidylglycerophosphate synthase 1 PAK6 - p21 protein (cdc42/rac)-activated kinase 6 TRAP1 - tnf receptor-associated protein 1 RUVBL2 - ruvb-like 2 (e. coli) COL13A1 - collagen, type xiii, alpha 1 PRKAA2 - protein kinase, amp-activated, alpha 2 catalytic subunit MYO15A - myosin xva NAT6 - n-acetyltransferase 6 (gcn5-related) CAMK1D - calcium/calmodulin-dependent protein kinase id KIF3C - kinesin family member 3c CAMK2G - calcium/calmodulin-dependent protein kinase ii gamma ADRBK2 - adrenergic, beta, receptor kinase 2 AGK - acylglycerol kinase ADRBK1 - adrenergic, beta, receptor kinase 1 ADAMTS8 - adam metallopeptidase with thrombospondin type 1 motif, 8 KIF5A - kinesin family member 5a CHRD - chordin CAMK2A - calcium/calmodulin-dependent protein kinase ii alpha ABCC8 - atp-binding cassette, sub-family c (cftr/mrp), member 8 KHK - ketohexokinase (fructokinase) TIMM44 - translocase of inner mitochondrial membrane 44 homolog (yeast) PTK2B - protein tyrosine kinase 2 beta CIT - citron (rho-interacting, serine/threonine kinase 21) PPP5C - protein phosphatase 5, catalytic subunit CUL9 - cullin 9 CARS - cysteinyl-trna synthetase FARSA - phenylalanyl-trna synthetase, alpha subunit AACS - acetoacetyl-coa synthetase CAD - carbamoyl-phosphate synthetase 2, aspartate transcarbamylase, and dihydroorotase CTSK - cathepsin k PINK1 - pten induced putative kinase 1 HK1 - hexokinase 1 XRCC6 - x-ray repair complementing defective repair in chinese hamster cells 6 UBE2E3 - ubiquitin-conjugating enzyme e2e 3 ADCK2 - aarf domain containing kinase 2 KIF25 - kinesin family member 25 KIFC2 - kinesin family member c2 DDX51 - dead (asp-glu-ala-asp) box polypeptide 51 CAMKK1 - calcium/calmodulin-dependent protein kinase kinase 1, alpha VARS - valyl-trna synthetase STK25 - serine/threonine kinase 25 RAPGEF4 - rap guanine nucleotide exchange factor (gef) 4 STK11 - serine/threonine kinase 11 RABL6 - rab, member ras oncogene family-like 6 TTLL12 - tubulin tyrosine ligase-like family, member 12 LONP1 - lon peptidase 1, mitochondrial EEFSEC - eukaryotic elongation factor, selenocysteine-trna-specific TUBA1B - tubulin, alpha 1b PRPS2 - phosphoribosyl pyrophosphate synthetase 2 GAK - cyclin g associated kinase PRPS1 - phosphoribosyl pyrophosphate synthetase 1 RAB26 - rab26, member ras oncogene family SLIT2 - slit homolog 2 (drosophila) GPAA1 - glycosylphosphatidylinositol anchor attachment 1 PRKY - protein kinase, y-linked, pseudogene GFPT2 - glutamine-fructose-6-phosphate transaminase 2 CAMK1G - calcium/calmodulin-dependent protein kinase ig XYLB - xylulokinase homolog (h. influenzae) ACAD9 - acyl-coa dehydrogenase family, member 9 UCKL1 - uridine-cytidine kinase 1-like 1 TYRO3 - tyro3 protein tyrosine kinase ITPKA - inositol-trisphosphate 3-kinase a TYK2 - tyrosine kinase 2 RSPO2 - r-spondin 2 MAP2K1 - mitogen-activated protein kinase kinase 1 DHX35 - deah (asp-glu-ala-his) box polypeptide 35 MAP2K2 - mitogen-activated protein kinase kinase 2 MAPK10 - mitogen-activated protein kinase 10 MAPK11 - mitogen-activated protein kinase 11 EPHA3 - eph receptor a3 KCNJ11 - potassium inwardly-rectifying channel, subfamily j, member 11 PKDCC - protein kinase domain containing, cytoplasmic MAPK3 - mitogen-activated protein kinase 3 PRKG2 - protein kinase, cgmp-dependent, type ii EPHB3 - eph receptor b3 EPHA5 - eph receptor a5 EPHA4 - eph receptor a4 KCNJ8 - potassium inwardly-rectifying channel, subfamily j, member 8 CSNK1E - casein kinase 1, epsilon ATP13A1 - atpase type 13a1 PRKCE - protein kinase c, epsilon EPHB6 - eph receptor b6 PRKCB - protein kinase c, beta GAP43 - growth associated protein 43 SPON2 - spondin 2, extracellular matrix protein SLC27A2 - solute carrier family 27 (fatty acid transporter), member 2 ADCK1 - aarf domain containing kinase 1 UBE2O - ubiquitin-conjugating enzyme e2o ERCC2 - excision repair cross-complementing rodent repair deficiency, complementation group 2 PAK7 - p21 protein (cdc42/rac)-activated kinase 7 CDC6 - cell division cycle 6 MTPAP - mitochondrial poly(a) polymerase DHX8 - deah (asp-glu-ala-his) box polypeptide 8 TUBA3D - tubulin, alpha 3d FLT3 - fms-related tyrosine kinase 3 MAP3K9 - mitogen-activated protein kinase kinase kinase 9 ANXA6 - annexin a6 TUBA8 - tubulin, alpha 8 RAB15 - rab15, member ras oncogene family MTHFD1L - methylenetetrahydrofolate dehydrogenase (nadp+ dependent) 1-like LRRK1 - leucine-rich repeat kinase 1 NLK - nemo-like kinase UBE2QL1 - ubiquitin-conjugating enzyme e2q family-like 1 MAGI1 - membrane associated guanylate kinase, ww and pdz domain containing 1 NUAK1 - nuak family, snf1-like kinase, 1 PAK1 - p21 protein (cdc42/rac)-activated kinase 1 RASL10A - ras-like, family 10, member a POSTN - periostin, osteoblast specific factor DGKZ - diacylglycerol kinase, zeta FGFRL1 - fibroblast growth factor receptor-like 1 FGF9 - fibroblast growth factor 9 TUBA1A - tubulin, alpha 1a ABCB8 - atp-binding cassette, sub-family b (mdr/tap), member 8 CHD5 - chromodomain helicase dna binding protein 5 PDPK1 - 3-phosphoinositide dependent protein kinase-1 RAB24 - rab24, member ras oncogene family FES - feline sarcoma oncogene DHX38 - deah (asp-glu-ala-his) box polypeptide 38 DCLK1 - doublecortin-like kinase 1 SCN8A - sodium channel, voltage gated, type viii, alpha subunit HIST1H1A - histone cluster 1, h1a CDK5 - cyclin-dependent kinase 5 ASNA1 - arsa arsenite transporter, atp-binding, homolog 1 (bacterial) DAPK3 - death-associated protein kinase 3 RND1 - rho family gtpase 1 CDK9 - cyclin-dependent kinase 9 DGKA - diacylglycerol kinase, alpha 80kda MET - met proto-oncogene MAP3K13 - mitogen-activated protein kinase kinase kinase 13 ARL9 - adp-ribosylation factor-like 9 ASS1 - argininosuccinate synthase 1 MAPK12 - mitogen-activated protein kinase 12 TNNI3K - tnni3 interacting kinase SARS - seryl-trna synthetase PDE2A - phosphodiesterase 2a, cgmp-stimulated TUBB8 - tubulin, beta 8 class viii PDE4A - phosphodiesterase 4a, camp-specific PTPRF - protein tyrosine phosphatase, receptor type, f GPN2 - gpn-loop gtpase 2 GTPBP2 - gtp binding protein 2 RHEBL1 - ras homolog enriched in brain like 1 NOS2 - nitric oxide synthase 2, inducible GNL1 - guanine nucleotide binding protein-like 1 ATP12A - atpase, h+/k+ transporting, nongastric, alpha polypeptide P2RX6 - purinergic receptor p2x, ligand-gated ion channel, 6 ATP1A1 - atpase, na+/k+ transporting, alpha 1 polypeptide CDK14 - cyclin-dependent kinase 14 NME1 - nme/nm23 nucleoside diphosphate kinase 1 ATP1A4 - atpase, na+/k+ transporting, alpha 4 polypeptide STK32C - serine/threonine kinase 32c NOV - nephroblastoma overexpressed RAB6B - rab6b, member ras oncogene family MORC2 - morc family cw-type zinc finger 2 MYO19 - myosin xix CHKB - choline kinase beta CNGB3 - cyclic nucleotide gated channel beta 3 MAP4K2 - mitogen-activated protein kinase kinase kinase kinase 2 ABCB9 - atp-binding cassette, sub-family b (mdr/tap), member 9 RAB3A - rab3a, member ras oncogene family ARL10 - adp-ribosylation factor-like 10 SKIV2L - superkiller viralicidic activity 2-like (s. cerevisiae) HAPLN4 - hyaluronan and proteoglycan link protein 4 HCN1 - hyperpolarization activated cyclic nucleotide-gated potassium channel 1 ATP6V1B1 - atpase, h+ transporting, lysosomal 56/58kda, v1 subunit b1 PIP5K1B - phosphatidylinositol-4-phosphate 5-kinase, type i, beta MATK - megakaryocyte-associated tyrosine kinase STYK1 - serine/threonine/tyrosine kinase 1 PIF1 - pif1 5'-to-3' dna helicase TUBG2 - tubulin, gamma 2 RAC2 - ras-related c3 botulinum toxin substrate 2 (rho family, small gtp binding protein rac2) PFKP - phosphofructokinase, platelet ATP2B4 - atpase, ca++ transporting, plasma membrane 4 PFAS - phosphoribosylformylglycinamidine synthase ATP2B3 - atpase, ca++ transporting, plasma membrane 3 EHD2 - eh-domain containing 2 MAP3K6 - mitogen-activated protein kinase kinase kinase 6 INO80 - ino80 complex subunit EHD3 - eh-domain containing 3 DDX56 - dead (asp-glu-ala-asp) box helicase 56 RAB27B - rab27b, member ras oncogene family ATP8B1 - atpase, aminophospholipid transporter, class i, type 8b, member 1 GRK6 - g protein-coupled receptor kinase 6 COL5A3 - collagen, type v, alpha 3 PAPSS2 - 3'-phosphoadenosine 5'-phosphosulfate synthase 2 PANK4 - pantothenate kinase 4 PI4KA - phosphatidylinositol 4-kinase, catalytic, alpha TESK1 - testis-specific kinase 1 TPK1 - thiamin pyrophosphokinase 1 MYH7B - myosin, heavy chain 7b, cardiac muscle, beta RASD1 - ras, dexamethasone-induced 1 KIF1A - kinesin family member 1a FLAD1 - flavin adenine dinucleotide synthetase 1 BRSK2 - br serine/threonine kinase 2 LTK - leukocyte receptor tyrosine kinase FBLN7 - fibulin 7 CLK2 - cdc-like kinase 2 TWF2 - twinfilin actin-binding protein 2 RHOV - ras homolog family member v ABCC10 - atp-binding cassette, sub-family c (cftr/mrp), member 10 CLCN7 - chloride channel, voltage-sensitive 7 RTN4RL1 - reticulon 4 receptor-like 1 ACOT7 - acyl-coa thioesterase 7 KALRN - kalirin, rhogef kinase ATAD3B - atpase family, aaa domain containing 3b PHKG2 - phosphorylase kinase, gamma 2 (testis) RPS6KA4 - ribosomal protein s6 kinase, 90kda, polypeptide 4 DHX16 - deah (asp-glu-ala-his) box polypeptide 16 PPIP5K1 - diphosphoinositol pentakisphosphate kinase 1 PIK3CD - phosphatidylinositol-4,5-bisphosphate 3-kinase, catalytic subunit delta CKMT1B - creatine kinase, mitochondrial 1b PIM1 - pim-1 oncogene THBS3 - thrombospondin 3 DNM1 - dynamin 1 UBE2D4 - ubiquitin-conjugating enzyme e2d 4 (putative) TARS2 - threonyl-trna synthetase 2, mitochondrial (putative) NAV2 - neuron navigator 2 GSK3A - glycogen synthase kinase 3 alpha RHOF - ras homolog family member f (in filopodia) |
| GO:0005230 | extracellular ligand-gated ion channel activity | 1.58E-5 | 1.58E-3 | 4.99 (16750,61,716,13) | GRIN2A - glutamate receptor, ionotropic, n-methyl d-aspartate 2a CHRNA7 - cholinergic receptor, nicotinic, alpha 7 (neuronal) GRIK3 - glutamate receptor, ionotropic, kainate 3 GRIK4 - glutamate receptor, ionotropic, kainate 4 GABRA1 - gamma-aminobutyric acid (gaba) a receptor, alpha 1 P2RX6 - purinergic receptor p2x, ligand-gated ion channel, 6 GLRA3 - glycine receptor, alpha 3 GABRA4 - gamma-aminobutyric acid (gaba) a receptor, alpha 4 GABRG3 - gamma-aminobutyric acid (gaba) a receptor, gamma 3 PTK2B - protein tyrosine kinase 2 beta GRIA4 - glutamate receptor, ionotropic, ampa 4 SLC17A7 - solute carrier family 17 (vesicular glutamate transporter), member 7 GRIN3A - glutamate receptor, ionotropic, n-methyl-d-aspartate 3a |
| GO:0036094 | small molecule binding | 1.76E-5 | 1.73E-3 | 1.28 (16750,2249,1807,310) | TRIB3 - tribbles homolog 3 (drosophila) RFC2 - replication factor c (activator 1) 2, 40kda AGAP3 - arfgap with gtpase domain, ankyrin repeat and ph domain 3 LIMK1 - lim domain kinase 1 BCKDK - branched chain ketoacid dehydrogenase kinase DHX30 - deah (asp-glu-ala-his) box helicase 30 SEPT5 - septin 5 DHRS11 - dehydrogenase/reductase (sdr family) member 11 ABCA4 - atp-binding cassette, sub-family a (abc1), member 4 SPG7 - spastic paraplegia 7 (pure and complicated autosomal recessive) RET - ret proto-oncogene CDKL1 - cyclin-dependent kinase-like 1 (cdc2-related kinase) RLBP1 - retinaldehyde binding protein 1 TUBA4A - tubulin, alpha 4a TUBA3C - tubulin, alpha 3c TPX2 - tpx2, microtubule-associated RAB3D - rab3d, member ras oncogene family BRSK1 - br serine/threonine kinase 1 CCT5 - chaperonin containing tcp1, subunit 5 (epsilon) PIP5KL1 - phosphatidylinositol-4-phosphate 5-kinase-like 1 SPHK2 - sphingosine kinase 2 UBE2J2 - ubiquitin-conjugating enzyme e2, j2 RAB37 - rab37, member ras oncogene family MAGI3 - membrane associated guanylate kinase, ww and pdz domain containing 3 STK19 - serine/threonine kinase 19 TUBA4B - tubulin, alpha 4b (pseudogene) RAB40C - rab40c, member ras oncogene family NME6 - nme/nm23 nucleoside diphosphate kinase 6 RIMKLA - ribosomal modification protein rimk-like family member a DCTPP1 - dctp pyrophosphatase 1 RHO - rhodopsin MYO5B - myosin vb MAST1 - microtubule associated serine/threonine kinase 1 EEF1A2 - eukaryotic translation elongation factor 1 alpha 2 GLDC - glycine dehydrogenase (decarboxylating) MAST3 - microtubule associated serine/threonine kinase 3 ADCY6 - adenylate cyclase 6 NGB - neuroglobin MYO16 - myosin xvi PIM3 - pim-3 oncogene NT5C1A - 5'-nucleotidase, cytosolic ia GRIN3A - glutamate receptor, ionotropic, n-methyl-d-aspartate 3a ADCY2 - adenylate cyclase 2 (brain) SNRNP200 - small nuclear ribonucleoprotein 200kda (u5) DIRAS2 - diras family, gtp-binding ras-like 2 NT5M - 5',3'-nucleotidase, mitochondrial FIGNL2 - fidgetin-like 2 PNCK - pregnancy up-regulated non-ubiquitously expressed cam kinase NRBP1 - nuclear receptor binding protein 1 KIF21B - kinesin family member 21b MVD - mevalonate (diphospho) decarboxylase TRPM4 - transient receptor potential cation channel, subfamily m, member 4 CSAD - cysteine sulfinic acid decarboxylase LCN1 - lipocalin 1 GNA15 - guanine nucleotide binding protein (g protein), alpha 15 (gq class) GMPPB - gdp-mannose pyrophosphorylase b GNA11 - guanine nucleotide binding protein (g protein), alpha 11 (gq class) POLD1 - polymerase (dna directed), delta 1, catalytic subunit ARL4A - adp-ribosylation factor-like 4a ADORA1 - adenosine a1 receptor ARL4C - adp-ribosylation factor-like 4c PGS1 - phosphatidylglycerophosphate synthase 1 PAK6 - p21 protein (cdc42/rac)-activated kinase 6 TRAP1 - tnf receptor-associated protein 1 C8G - complement component 8, gamma polypeptide RUVBL2 - ruvb-like 2 (e. coli) PRKAA2 - protein kinase, amp-activated, alpha 2 catalytic subunit MYO15A - myosin xva MTR - 5-methyltetrahydrofolate-homocysteine methyltransferase SYP - synaptophysin SYN2 - synapsin ii NAT6 - n-acetyltransferase 6 (gcn5-related) FABP3 - fatty acid binding protein 3, muscle and heart (mammary-derived growth inhibitor) CAMK1D - calcium/calmodulin-dependent protein kinase id UBE2Z - ubiquitin-conjugating enzyme e2z KIF3C - kinesin family member 3c CAMK2G - calcium/calmodulin-dependent protein kinase ii gamma ADRBK2 - adrenergic, beta, receptor kinase 2 AGK - acylglycerol kinase ADRBK1 - adrenergic, beta, receptor kinase 1 KIF5A - kinesin family member 5a CAMK2A - calcium/calmodulin-dependent protein kinase ii alpha ABCC8 - atp-binding cassette, sub-family c (cftr/mrp), member 8 KHK - ketohexokinase (fructokinase) TIMM44 - translocase of inner mitochondrial membrane 44 homolog (yeast) PTK2B - protein tyrosine kinase 2 beta CIT - citron (rho-interacting, serine/threonine kinase 21) PPP5C - protein phosphatase 5, catalytic subunit CUL9 - cullin 9 CARS - cysteinyl-trna synthetase FARSA - phenylalanyl-trna synthetase, alpha subunit AACS - acetoacetyl-coa synthetase FASN - fatty acid synthase CAD - carbamoyl-phosphate synthetase 2, aspartate transcarbamylase, and dihydroorotase PINK1 - pten induced putative kinase 1 HK1 - hexokinase 1 XRCC6 - x-ray repair complementing defective repair in chinese hamster cells 6 UBE2E3 - ubiquitin-conjugating enzyme e2e 3 ADCK2 - aarf domain containing kinase 2 KIF25 - kinesin family member 25 KIFC2 - kinesin family member c2 DDX51 - dead (asp-glu-ala-asp) box polypeptide 51 CAMKK1 - calcium/calmodulin-dependent protein kinase kinase 1, alpha VARS - valyl-trna synthetase STK25 - serine/threonine kinase 25 RAPGEF4 - rap guanine nucleotide exchange factor (gef) 4 ALAS1 - aminolevulinate, delta-, synthase 1 STK11 - serine/threonine kinase 11 RABL6 - rab, member ras oncogene family-like 6 TTLL12 - tubulin tyrosine ligase-like family, member 12 LONP1 - lon peptidase 1, mitochondrial EEFSEC - eukaryotic elongation factor, selenocysteine-trna-specific GAK - cyclin g associated kinase TUBA1B - tubulin, alpha 1b PRPS2 - phosphoribosyl pyrophosphate synthetase 2 PRPS1 - phosphoribosyl pyrophosphate synthetase 1 PRODH - proline dehydrogenase (oxidase) 1 RAB26 - rab26, member ras oncogene family PRKY - protein kinase, y-linked, pseudogene CAMK1G - calcium/calmodulin-dependent protein kinase ig XYLB - xylulokinase homolog (h. influenzae) ACAD9 - acyl-coa dehydrogenase family, member 9 UCKL1 - uridine-cytidine kinase 1-like 1 TYRO3 - tyro3 protein tyrosine kinase ITPKA - inositol-trisphosphate 3-kinase a TYK2 - tyrosine kinase 2 MAP2K1 - mitogen-activated protein kinase kinase 1 DHX35 - deah (asp-glu-ala-his) box polypeptide 35 ITPR1 - inositol 1,4,5-trisphosphate receptor, type 1 MAP2K2 - mitogen-activated protein kinase kinase 2 GAD1 - glutamate decarboxylase 1 (brain, 67kda) MAPK10 - mitogen-activated protein kinase 10 MAPK11 - mitogen-activated protein kinase 11 EPHA3 - eph receptor a3 KCNJ11 - potassium inwardly-rectifying channel, subfamily j, member 11 PKDCC - protein kinase domain containing, cytoplasmic MAPK3 - mitogen-activated protein kinase 3 PRKG2 - protein kinase, cgmp-dependent, type ii GLRA3 - glycine receptor, alpha 3 OSBPL10 - oxysterol binding protein-like 10 EPHB3 - eph receptor b3 EPHA5 - eph receptor a5 EPHA4 - eph receptor a4 KCNJ8 - potassium inwardly-rectifying channel, subfamily j, member 8 OSBPL5 - oxysterol binding protein-like 5 CSNK1E - casein kinase 1, epsilon ATP13A1 - atpase type 13a1 PRKCE - protein kinase c, epsilon EPHB6 - eph receptor b6 PRKCB - protein kinase c, beta SLC27A2 - solute carrier family 27 (fatty acid transporter), member 2 CBS - cystathionine-beta-synthase SLC27A4 - solute carrier family 27 (fatty acid transporter), member 4 ADCK1 - aarf domain containing kinase 1 UBE2O - ubiquitin-conjugating enzyme e2o ERCC2 - excision repair cross-complementing rodent repair deficiency, complementation group 2 PAK7 - p21 protein (cdc42/rac)-activated kinase 7 CDIPT - cdp-diacylglycerol--inositol 3-phosphatidyltransferase CCBL1 - cysteine conjugate-beta lyase, cytoplasmic CDC6 - cell division cycle 6 MTPAP - mitochondrial poly(a) polymerase DHX8 - deah (asp-glu-ala-his) box polypeptide 8 TUBA3D - tubulin, alpha 3d FLT3 - fms-related tyrosine kinase 3 MAP3K9 - mitogen-activated protein kinase kinase kinase 9 ANXA6 - annexin a6 STARD5 - star-related lipid transfer (start) domain containing 5 TUBA8 - tubulin, alpha 8 RAB15 - rab15, member ras oncogene family LCN15 - lipocalin 15 MTHFD1L - methylenetetrahydrofolate dehydrogenase (nadp+ dependent) 1-like LRRK1 - leucine-rich repeat kinase 1 NLK - nemo-like kinase TDO2 - tryptophan 2,3-dioxygenase UBE2QL1 - ubiquitin-conjugating enzyme e2q family-like 1 FKBP1B - fk506 binding protein 1b, 12.6 kda MAGI1 - membrane associated guanylate kinase, ww and pdz domain containing 1 NUAK1 - nuak family, snf1-like kinase, 1 PAK1 - p21 protein (cdc42/rac)-activated kinase 1 RASL10A - ras-like, family 10, member a DGKZ - diacylglycerol kinase, zeta LEPREL2 - leprecan-like 2 TUBA1A - tubulin, alpha 1a CYP2E1 - cytochrome p450, family 2, subfamily e, polypeptide 1 ABCB8 - atp-binding cassette, sub-family b (mdr/tap), member 8 CHD5 - chromodomain helicase dna binding protein 5 PDPK1 - 3-phosphoinositide dependent protein kinase-1 RAB4B - rab4b, member ras oncogene family RAB24 - rab24, member ras oncogene family FES - feline sarcoma oncogene OSBPL3 - oxysterol binding protein-like 3 DHX38 - deah (asp-glu-ala-his) box polypeptide 38 DCLK1 - doublecortin-like kinase 1 SCN8A - sodium channel, voltage gated, type viii, alpha subunit KMO - kynurenine 3-monooxygenase (kynurenine 3-hydroxylase) CDK5 - cyclin-dependent kinase 5 ASNA1 - arsa arsenite transporter, atp-binding, homolog 1 (bacterial) DAPK3 - death-associated protein kinase 3 RND1 - rho family gtpase 1 CDK9 - cyclin-dependent kinase 9 DBH - dopamine beta-hydroxylase (dopamine beta-monooxygenase) DGKA - diacylglycerol kinase, alpha 80kda MET - met proto-oncogene MAP3K13 - mitogen-activated protein kinase kinase kinase 13 ARL9 - adp-ribosylation factor-like 9 ASS1 - argininosuccinate synthase 1 MAPK12 - mitogen-activated protein kinase 12 TNNI3K - tnni3 interacting kinase SARS - seryl-trna synthetase PDE2A - phosphodiesterase 2a, cgmp-stimulated MBD3 - methyl-cpg binding domain protein 3 TUBB8 - tubulin, beta 8 class viii PDE4A - phosphodiesterase 4a, camp-specific CYP26A1 - cytochrome p450, family 26, subfamily a, polypeptide 1 GPN2 - gpn-loop gtpase 2 GTPBP2 - gtp binding protein 2 RTBDN - retbindin SLC2A8 - solute carrier family 2 (facilitated glucose transporter), member 8 RHEBL1 - ras homolog enriched in brain like 1 NOS2 - nitric oxide synthase 2, inducible GNL1 - guanine nucleotide binding protein-like 1 P4HTM - prolyl 4-hydroxylase, transmembrane (endoplasmic reticulum) ATP12A - atpase, h+/k+ transporting, nongastric, alpha polypeptide P2RX6 - purinergic receptor p2x, ligand-gated ion channel, 6 ATP1A1 - atpase, na+/k+ transporting, alpha 1 polypeptide CDK14 - cyclin-dependent kinase 14 MICAL3 - microtubule associated monooxygenase, calponin and lim domain containing 3 NME1 - nme/nm23 nucleoside diphosphate kinase 1 RPH3A - rabphilin 3a homolog (mouse) PGD - phosphogluconate dehydrogenase GOT1 - glutamic-oxaloacetic transaminase 1, soluble ATP1A4 - atpase, na+/k+ transporting, alpha 4 polypeptide STK32C - serine/threonine kinase 32c RAB6B - rab6b, member ras oncogene family MORC2 - morc family cw-type zinc finger 2 MYO19 - myosin xix SIRT6 - sirtuin 6 SIRT7 - sirtuin 7 CHKB - choline kinase beta SIRT4 - sirtuin 4 CNGB3 - cyclic nucleotide gated channel beta 3 MAP4K2 - mitogen-activated protein kinase kinase kinase kinase 2 ABCB9 - atp-binding cassette, sub-family b (mdr/tap), member 9 HAPLN4 - hyaluronan and proteoglycan link protein 4 ARL10 - adp-ribosylation factor-like 10 RAB3A - rab3a, member ras oncogene family SKIV2L - superkiller viralicidic activity 2-like (s. cerevisiae) HCN1 - hyperpolarization activated cyclic nucleotide-gated potassium channel 1 ATP6V1B1 - atpase, h+ transporting, lysosomal 56/58kda, v1 subunit b1 PIP5K1B - phosphatidylinositol-4-phosphate 5-kinase, type i, beta MATK - megakaryocyte-associated tyrosine kinase STYK1 - serine/threonine/tyrosine kinase 1 PIF1 - pif1 5'-to-3' dna helicase TUBG2 - tubulin, gamma 2 PFKP - phosphofructokinase, platelet RAC2 - ras-related c3 botulinum toxin substrate 2 (rho family, small gtp binding protein rac2) ATP2B4 - atpase, ca++ transporting, plasma membrane 4 ATP2B3 - atpase, ca++ transporting, plasma membrane 3 PFAS - phosphoribosylformylglycinamidine synthase EHD2 - eh-domain containing 2 MAP3K6 - mitogen-activated protein kinase kinase kinase 6 INO80 - ino80 complex subunit EHD3 - eh-domain containing 3 RAB27B - rab27b, member ras oncogene family DDX56 - dead (asp-glu-ala-asp) box helicase 56 ATP8B1 - atpase, aminophospholipid transporter, class i, type 8b, member 1 GRK6 - g protein-coupled receptor kinase 6 PAPSS2 - 3'-phosphoadenosine 5'-phosphosulfate synthase 2 PANK4 - pantothenate kinase 4 EGLN2 - egl-9 family hypoxia-inducible factor 2 GPT - glutamic-pyruvate transaminase (alanine aminotransferase) MICAL2 - microtubule associated monooxygenase, calponin and lim domain containing 2 PI4KA - phosphatidylinositol 4-kinase, catalytic, alpha CYP26B1 - cytochrome p450, family 26, subfamily b, polypeptide 1 TESK1 - testis-specific kinase 1 COL20A1 - collagen, type xx, alpha 1 DHCR7 - 7-dehydrocholesterol reductase TPK1 - thiamin pyrophosphokinase 1 MYH7B - myosin, heavy chain 7b, cardiac muscle, beta RASD1 - ras, dexamethasone-induced 1 SESN2 - sestrin 2 KIF1A - kinesin family member 1a FLAD1 - flavin adenine dinucleotide synthetase 1 BRSK2 - br serine/threonine kinase 2 LTK - leukocyte receptor tyrosine kinase CLK2 - cdc-like kinase 2 GRAMD1A - gram domain containing 1a RHOV - ras homolog family member v TWF2 - twinfilin actin-binding protein 2 ACAD8 - acyl-coa dehydrogenase family, member 8 ABCC10 - atp-binding cassette, sub-family c (cftr/mrp), member 10 CLCN7 - chloride channel, voltage-sensitive 7 ACOT7 - acyl-coa thioesterase 7 KALRN - kalirin, rhogef kinase ATAD3B - atpase family, aaa domain containing 3b PHKG2 - phosphorylase kinase, gamma 2 (testis) RPS6KA4 - ribosomal protein s6 kinase, 90kda, polypeptide 4 DHX16 - deah (asp-glu-ala-his) box polypeptide 16 PPIP5K1 - diphosphoinositol pentakisphosphate kinase 1 PIK3CD - phosphatidylinositol-4,5-bisphosphate 3-kinase, catalytic subunit delta CKMT1B - creatine kinase, mitochondrial 1b PIM1 - pim-1 oncogene DNM1 - dynamin 1 UBE2D4 - ubiquitin-conjugating enzyme e2d 4 (putative) DNAH5 - dynein, axonemal, heavy chain 5 TARS2 - threonyl-trna synthetase 2, mitochondrial (putative) GSK3A - glycogen synthase kinase 3 alpha NAV2 - neuron navigator 2 P4HA2 - prolyl 4-hydroxylase, alpha polypeptide ii RHOF - ras homolog family member f (in filopodia) |
| GO:0030594 | neurotransmitter receptor activity | 1.88E-5 | 1.8E-3 | 3.89 (16750,99,739,17) | GRIN2A - glutamate receptor, ionotropic, n-methyl d-aspartate 2a CHRNA7 - cholinergic receptor, nicotinic, alpha 7 (neuronal) GRIK3 - glutamate receptor, ionotropic, kainate 3 GRIK4 - glutamate receptor, ionotropic, kainate 4 GABRA1 - gamma-aminobutyric acid (gaba) a receptor, alpha 1 GLRA3 - glycine receptor, alpha 3 CHRM3 - cholinergic receptor, muscarinic 3 GABRA4 - gamma-aminobutyric acid (gaba) a receptor, alpha 4 GABBR2 - gamma-aminobutyric acid (gaba) b receptor, 2 GABRG3 - gamma-aminobutyric acid (gaba) a receptor, gamma 3 PTK2B - protein tyrosine kinase 2 beta HTR5A - 5-hydroxytryptamine (serotonin) receptor 5a, g protein-coupled HTR1A - 5-hydroxytryptamine (serotonin) receptor 1a, g protein-coupled GRIA4 - glutamate receptor, ionotropic, ampa 4 HTR2A - 5-hydroxytryptamine (serotonin) receptor 2a, g protein-coupled HTR1F - 5-hydroxytryptamine (serotonin) receptor 1f, g protein-coupled GRIN3A - glutamate receptor, ionotropic, n-methyl-d-aspartate 3a |
| GO:0004683 | calmodulin-dependent protein kinase activity | 2.57E-5 | 2.42E-3 | 10.63 (16750,28,394,7) | CAMKK1 - calcium/calmodulin-dependent protein kinase kinase 1, alpha CAMK1G - calcium/calmodulin-dependent protein kinase ig PTK2B - protein tyrosine kinase 2 beta PHKG2 - phosphorylase kinase, gamma 2 (testis) ITPKA - inositol-trisphosphate 3-kinase a CAMK2G - calcium/calmodulin-dependent protein kinase ii gamma CAMK2A - calcium/calmodulin-dependent protein kinase ii alpha |
| GO:0008066 | glutamate receptor activity | 3.1E-5 | 2.85E-3 | 5.42 (16750,22,1405,10) | GRIN2A - glutamate receptor, ionotropic, n-methyl d-aspartate 2a GRIK1 - glutamate receptor, ionotropic, kainate 1 PTK2B - protein tyrosine kinase 2 beta GRIK2 - glutamate receptor, ionotropic, kainate 2 GRIK3 - glutamate receptor, ionotropic, kainate 3 GRIK4 - glutamate receptor, ionotropic, kainate 4 GRIA4 - glutamate receptor, ionotropic, ampa 4 GRM2 - glutamate receptor, metabotropic 2 GRIA3 - glutamate receptor, ionotropic, ampa 3 GRIN3A - glutamate receptor, ionotropic, n-methyl-d-aspartate 3a |
| GO:0016740 | transferase activity | 3.73E-5 | 3.36E-3 | 1.30 (16750,2057,1615,258) | TRMT2B - trna methyltransferase 2 homolog b (s. cerevisiae) GFPT1 - glutamine--fructose-6-phosphate transaminase 1 MKRN1 - makorin ring finger protein 1 BCKDK - branched chain ketoacid dehydrogenase kinase LIMK1 - lim domain kinase 1 GCNT4 - glucosaminyl (n-acetyl) transferase 4, core 2 RET - ret proto-oncogene DPF1 - d4, zinc and double phd fingers family 1 TRIM7 - tripartite motif containing 7 HRASLS5 - hras-like suppressor family, member 5 CCNO - cyclin o KAT2A - k(lysine) acetyltransferase 2a GBA2 - glucosidase, beta (bile acid) 2 UAP1 - udp-n-acteylglucosamine pyrophosphorylase 1 NCOA3 - nuclear receptor coactivator 3 CDKL1 - cyclin-dependent kinase-like 1 (cdc2-related kinase) CHSY3 - chondroitin sulfate synthase 3 POLN - polymerase (dna directed) nu BRSK1 - br serine/threonine kinase 1 NEURL4 - neuralized homolog 4 (drosophila) PIP5KL1 - phosphatidylinositol-4-phosphate 5-kinase-like 1 SPHK2 - sphingosine kinase 2 FBXL20 - f-box and leucine-rich repeat protein 20 UBE2J2 - ubiquitin-conjugating enzyme e2, j2 MAGI3 - membrane associated guanylate kinase, ww and pdz domain containing 3 STK19 - serine/threonine kinase 19 RNF215 - ring finger protein 215 NME6 - nme/nm23 nucleoside diphosphate kinase 6 MAST1 - microtubule associated serine/threonine kinase 1 GGT7 - gamma-glutamyltransferase 7 ST8SIA5 - st8 alpha-n-acetyl-neuraminide alpha-2,8-sialyltransferase 5 CCNA1 - cyclin a1 PRDM8 - pr domain containing 8 MAST3 - microtubule associated serine/threonine kinase 3 GOLGA7B - golgin a7 family, member b PIM3 - pim-3 oncogene PDZRN3 - pdz domain containing ring finger 3 HEMK1 - hemk methyltransferase family member 1 EFNA3 - ephrin-a3 PDZRN4 - pdz domain containing ring finger 4 E4F1 - e4f transcription factor 1 FEM1A - fem-1 homolog a (c. elegans) PNCK - pregnancy up-regulated non-ubiquitously expressed cam kinase MED20 - mediator complex subunit 20 FTSJ2 - ftsj rna methyltransferase homolog 2 (e. coli) SMYD2 - set and mynd domain containing 2 TRIM37 - tripartite motif containing 37 ST6GALNAC5 - st6 (alpha-n-acetyl-neuraminyl-2,3-beta-galactosyl-1,3)-n-acetylgalactosaminide alpha-2,6-sialyltransferase 5 GMPPA - gdp-mannose pyrophosphorylase a POLD2 - polymerase (dna directed), delta 2, accessory subunit GMPPB - gdp-mannose pyrophosphorylase b POLD1 - polymerase (dna directed), delta 1, catalytic subunit TRAF3 - tnf receptor-associated factor 3 PGS1 - phosphatidylglycerophosphate synthase 1 NEURL3 - neuralized homolog 3 (drosophila) pseudogene HECW1 - hect, c2 and ww domain containing e3 ubiquitin protein ligase 1 POLR2H - polymerase (rna) ii (dna directed) polypeptide h MARCH9 - membrane-associated ring finger (c3hc4) 9 POLR2E - polymerase (rna) ii (dna directed) polypeptide e, 25kda PAK6 - p21 protein (cdc42/rac)-activated kinase 6 PRKAB1 - protein kinase, amp-activated, beta 1 non-catalytic subunit MCRS1 - microspherule protein 1 CAMK1D - calcium/calmodulin-dependent protein kinase id AGK - acylglycerol kinase ADRBK2 - adrenergic, beta, receptor kinase 2 CAMK2G - calcium/calmodulin-dependent protein kinase ii gamma ADRBK1 - adrenergic, beta, receptor kinase 1 HMBS - hydroxymethylbilane synthase CAMK2A - calcium/calmodulin-dependent protein kinase ii alpha KHK - ketohexokinase (fructokinase) PTK2B - protein tyrosine kinase 2 beta CIT - citron (rho-interacting, serine/threonine kinase 21) CUL9 - cullin 9 FASN - fatty acid synthase CAD - carbamoyl-phosphate synthetase 2, aspartate transcarbamylase, and dihydroorotase CSGALNACT1 - chondroitin sulfate n-acetylgalactosaminyltransferase 1 SPTSSB - serine palmitoyltransferase, small subunit b PINK1 - pten induced putative kinase 1 HK1 - hexokinase 1 TRIM17 - tripartite motif containing 17 LNX1 - ligand of numb-protein x 1, e3 ubiquitin protein ligase SPSB3 - spla/ryanodine receptor domain and socs box containing 3 UBE2E3 - ubiquitin-conjugating enzyme e2e 3 HERC6 - hect and rld domain containing e3 ubiquitin protein ligase family member 6 ADCK2 - aarf domain containing kinase 2 EXTL1 - exostosin-like glycosyltransferase 1 EXTL3 - exostosin-like glycosyltransferase 3 CAMKK1 - calcium/calmodulin-dependent protein kinase kinase 1, alpha STK25 - serine/threonine kinase 25 MED10 - mediator complex subunit 10 STK11 - serine/threonine kinase 11 ALAS1 - aminolevulinate, delta-, synthase 1 B3GALT2 - udp-gal:betaglcnac beta 1,3-galactosyltransferase, polypeptide 2 KANSL3 - kat8 regulatory nsl complex subunit 3 ADCK5 - aarf domain containing kinase 5 SULT4A1 - sulfotransferase family 4a, member 1 RNF165 - ring finger protein 165 B4GALNT1 - beta-1,4-n-acetyl-galactosaminyl transferase 1 AGPAT9 - 1-acylglycerol-3-phosphate o-acyltransferase 9 PRPS2 - phosphoribosyl pyrophosphate synthetase 2 GAK - cyclin g associated kinase MGAT4C - mannosyl (alpha-1,3-)-glycoprotein beta-1,4-n-acetylglucosaminyltransferase, isozyme c (putative) PRPS1 - phosphoribosyl pyrophosphate synthetase 1 CCNE1 - cyclin e1 NTMT1 - n-terminal xaa-pro-lys n-methyltransferase 1 CCNG2 - cyclin g2 PRKY - protein kinase, y-linked, pseudogene GFPT2 - glutamine-fructose-6-phosphate transaminase 2 CAMK1G - calcium/calmodulin-dependent protein kinase ig XYLB - xylulokinase homolog (h. influenzae) ZDHHC16 - zinc finger, dhhc-type containing 16 UCKL1 - uridine-cytidine kinase 1-like 1 FBXO6 - f-box protein 6 TYRO3 - tyro3 protein tyrosine kinase ITPKA - inositol-trisphosphate 3-kinase a TYK2 - tyrosine kinase 2 MAP2K1 - mitogen-activated protein kinase kinase 1 MAP2K2 - mitogen-activated protein kinase kinase 2 PHF20 - phd finger protein 20 MAPK10 - mitogen-activated protein kinase 10 MAPK11 - mitogen-activated protein kinase 11 EPHA3 - eph receptor a3 PKDCC - protein kinase domain containing, cytoplasmic MAPK3 - mitogen-activated protein kinase 3 HS3ST2 - heparan sulfate (glucosamine) 3-o-sulfotransferase 2 CCNI - cyclin i PRKG2 - protein kinase, cgmp-dependent, type ii GALNT11 - udp-n-acetyl-alpha-d-galactosamine:polypeptide n-acetylgalactosaminyltransferase 11 (galnac-t11) EPHB3 - eph receptor b3 EPHA5 - eph receptor a5 EPHA4 - eph receptor a4 HS3ST4 - heparan sulfate (glucosamine) 3-o-sulfotransferase 4 CSNK1E - casein kinase 1, epsilon DTX4 - deltex homolog 4 (drosophila) PRKCE - protein kinase c, epsilon EPHB6 - eph receptor b6 RNF123 - ring finger protein 123 CCND2 - cyclin d2 PRKCB - protein kinase c, beta ADCK1 - aarf domain containing kinase 1 ERCC2 - excision repair cross-complementing rodent repair deficiency, complementation group 2 PAK7 - p21 protein (cdc42/rac)-activated kinase 7 FBXL2 - f-box and leucine-rich repeat protein 2 CDIPT - cdp-diacylglycerol--inositol 3-phosphatidyltransferase MTPAP - mitochondrial poly(a) polymerase FN3KRP - fructosamine 3 kinase related protein RMND5A - required for meiotic nuclear division 5 homolog a (s. cerevisiae) FLT3 - fms-related tyrosine kinase 3 SETDB1 - set domain, bifurcated 1 DDB2 - damage-specific dna binding protein 2, 48kda COQ3 - coenzyme q3 methyltransferase RMND5B - required for meiotic nuclear division 5 homolog b (s. cerevisiae) MAP3K9 - mitogen-activated protein kinase kinase kinase 9 UBE3B - ubiquitin protein ligase e3b NAT8L - n-acetyltransferase 8-like (gcn5-related, putative) GSTT2 - glutathione s-transferase theta 2 POLL - polymerase (dna directed), lambda TRIM25 - tripartite motif containing 25 PRPF19 - pre-mrna processing factor 19 METTL21A - methyltransferase like 21a ZDHHC22 - zinc finger, dhhc-type containing 22 LRRK1 - leucine-rich repeat kinase 1 NLK - nemo-like kinase TRIM58 - tripartite motif containing 58 YRDC - yrdc domain containing (e. coli) FBXO40 - f-box protein 40 UBE2QL1 - ubiquitin-conjugating enzyme e2q family-like 1 TBRG4 - transforming growth factor beta regulator 4 NUAK1 - nuak family, snf1-like kinase, 1 PAK1 - p21 protein (cdc42/rac)-activated kinase 1 DGKZ - diacylglycerol kinase, zeta MARCH11 - membrane-associated ring finger (c3hc4) 11 RNF31 - ring finger protein 31 BRPF1 - bromodomain and phd finger containing, 1 FGFRL1 - fibroblast growth factor receptor-like 1 TAF5 - taf5 rna polymerase ii, tata box binding protein (tbp)-associated factor, 100kda CTU2 - cytosolic thiouridylase subunit 2 homolog (s. pombe) PDPK1 - 3-phosphoinositide dependent protein kinase-1 DPY19L1 - dpy-19-like 1 (c. elegans) FES - feline sarcoma oncogene RPAP1 - rna polymerase ii associated protein 1 A4GALT - alpha 1,4-galactosyltransferase ATP5SL - atp5s-like DCLK1 - doublecortin-like kinase 1 DAPK3 - death-associated protein kinase 3 CDK5 - cyclin-dependent kinase 5 PRDM2 - pr domain containing 2, with znf domain ZDHHC23 - zinc finger, dhhc-type containing 23 CDK9 - cyclin-dependent kinase 9 DGKA - diacylglycerol kinase, alpha 80kda MET - met proto-oncogene MAP3K13 - mitogen-activated protein kinase kinase kinase 13 LRSAM1 - leucine rich repeat and sterile alpha motif containing 1 ART3 - adp-ribosyltransferase 3 MAPK12 - mitogen-activated protein kinase 12 TNNI3K - tnni3 interacting kinase ZFP91 - zfp91 zinc finger protein RNF40 - ring finger protein 40, e3 ubiquitin protein ligase NEURL - neuralized homolog (drosophila) RNF208 - ring finger protein 208 ANAPC11 - anaphase promoting complex subunit 11 NME1 - nme/nm23 nucleoside diphosphate kinase 1 B4GALNT4 - beta-1,4-n-acetyl-galactosaminyl transferase 4 GOT1 - glutamic-oxaloacetic transaminase 1, soluble STK32C - serine/threonine kinase 32c DPAGT1 - dolichyl-phosphate (udp-n-acetylglucosamine) n-acetylglucosaminephosphotransferase 1 (glcnac-1-p transferase) SIRT6 - sirtuin 6 CHKB - choline kinase beta SIRT4 - sirtuin 4 PIGQ - phosphatidylinositol glycan anchor biosynthesis, class q PIP5K1B - phosphatidylinositol-4-phosphate 5-kinase, type i, beta MATK - megakaryocyte-associated tyrosine kinase STYK1 - serine/threonine/tyrosine kinase 1 TNFAIP1 - tumor necrosis factor, alpha-induced protein 1 (endothelial) PFKP - phosphofructokinase, platelet METTL21C - methyltransferase like 21c XYLT1 - xylosyltransferase i MAP3K6 - mitogen-activated protein kinase kinase kinase 6 DTX1 - deltex homolog 1 (drosophila) GRK6 - g protein-coupled receptor kinase 6 WDR82 - wd repeat domain 82 FUT9 - fucosyltransferase 9 (alpha (1,3) fucosyltransferase) PANK4 - pantothenate kinase 4 PGM2L1 - phosphoglucomutase 2-like 1 FBXL15 - f-box and leucine-rich repeat protein 15 TSPAN17 - tetraspanin 17 PI4KA - phosphatidylinositol 4-kinase, catalytic, alpha TERF2 - telomeric repeat binding factor 2 TESK1 - testis-specific kinase 1 ASB2 - ankyrin repeat and socs box containing 2 DHPS - deoxyhypusine synthase ST3GAL3 - st3 beta-galactoside alpha-2,3-sialyltransferase 3 GALNT9 - udp-n-acetyl-alpha-d-galactosamine:polypeptide n-acetylgalactosaminyltransferase 9 (galnac-t9) B3GNTL1 - udp-glcnac:betagal beta-1,3-n-acetylglucosaminyltransferase-like 1 TPK1 - thiamin pyrophosphokinase 1 ST3GAL1 - st3 beta-galactoside alpha-2,3-sialyltransferase 1 CDK5RAP1 - cdk5 regulatory subunit associated protein 1 FLAD1 - flavin adenine dinucleotide synthetase 1 BRSK2 - br serine/threonine kinase 2 LTK - leukocyte receptor tyrosine kinase CLK2 - cdc-like kinase 2 TRMT2A - trna methyltransferase 2 homolog a (s. cerevisiae) PIAS4 - protein inhibitor of activated stat, 4 FBXW2 - f-box and wd repeat domain containing 2 CHPF2 - chondroitin polymerizing factor 2 KALRN - kalirin, rhogef kinase PHKG2 - phosphorylase kinase, gamma 2 (testis) RPS6KA4 - ribosomal protein s6 kinase, 90kda, polypeptide 4 PPIP5K1 - diphosphoinositol pentakisphosphate kinase 1 CKMT1B - creatine kinase, mitochondrial 1b PIK3CD - phosphatidylinositol-4,5-bisphosphate 3-kinase, catalytic subunit delta PIM1 - pim-1 oncogene UBE2D4 - ubiquitin-conjugating enzyme e2d 4 (putative) TPST2 - tyrosylprotein sulfotransferase 2 SULT1A4 - sulfotransferase family, cytosolic, 1a, phenol-preferring, member 4 GSK3A - glycogen synthase kinase 3 alpha NAA50 - n(alpha)-acetyltransferase 50, nate catalytic subunit B4GALT7 - xylosylprotein beta 1,4-galactosyltransferase, polypeptide 7 |
| GO:0022824 | transmitter-gated ion channel activity | 4.12E-5 | 3.64E-3 | 5.36 (16750,48,716,11) | GRIN2A - glutamate receptor, ionotropic, n-methyl d-aspartate 2a GABRG3 - gamma-aminobutyric acid (gaba) a receptor, gamma 3 CHRNA7 - cholinergic receptor, nicotinic, alpha 7 (neuronal) PTK2B - protein tyrosine kinase 2 beta GRIK3 - glutamate receptor, ionotropic, kainate 3 GRIK4 - glutamate receptor, ionotropic, kainate 4 GABRA1 - gamma-aminobutyric acid (gaba) a receptor, alpha 1 GLRA3 - glycine receptor, alpha 3 GRIA4 - glutamate receptor, ionotropic, ampa 4 GABRA4 - gamma-aminobutyric acid (gaba) a receptor, alpha 4 GRIN3A - glutamate receptor, ionotropic, n-methyl-d-aspartate 3a |
| GO:0022835 | transmitter-gated channel activity | 4.12E-5 | 3.57E-3 | 5.36 (16750,48,716,11) | GRIN2A - glutamate receptor, ionotropic, n-methyl d-aspartate 2a GABRG3 - gamma-aminobutyric acid (gaba) a receptor, gamma 3 CHRNA7 - cholinergic receptor, nicotinic, alpha 7 (neuronal) PTK2B - protein tyrosine kinase 2 beta GRIK3 - glutamate receptor, ionotropic, kainate 3 GRIK4 - glutamate receptor, ionotropic, kainate 4 GABRA1 - gamma-aminobutyric acid (gaba) a receptor, alpha 1 GLRA3 - glycine receptor, alpha 3 GRIA4 - glutamate receptor, ionotropic, ampa 4 GABRA4 - gamma-aminobutyric acid (gaba) a receptor, alpha 4 GRIN3A - glutamate receptor, ionotropic, n-methyl-d-aspartate 3a |
| GO:0008092 | cytoskeletal protein binding | 5.2E-5 | 4.41E-3 | 1.44 (16750,872,1808,136) | EMD - emerin TAGLN3 - transgelin 3 CCDC64 - coiled-coil domain containing 64 LGMN - legumain SSH1 - slingshot protein phosphatase 1 KIFAP3 - kinesin-associated protein 3 SPTBN5 - spectrin, beta, non-erythrocytic 5 VILL - villin-like SOGA2 - soga family member 2 ENC1 - ectodermal-neural cortex 1 (with btb domain) SPAG5 - sperm associated antigen 5 BCL7B - b-cell cll/lymphoma 7b STMN3 - stathmin-like 3 ANXA6 - annexin a6 DCTN1 - dynactin 1 MYOZ2 - myozenin 2 FARP1 - ferm, rhogef (arhgef) and pleckstrin domain protein 1 (chondrocyte-derived) CDC42EP3 - cdc42 effector protein (rho gtpase binding) 3 MYRIP - myosin viia and rab interacting protein RAB3D - rab3d, member ras oncogene family BRSK1 - br serine/threonine kinase 1 NCALD - neurocalcin delta PLS1 - plastin 1 CCT5 - chaperonin containing tcp1, subunit 5 (epsilon) SNTG1 - syntrophin, gamma 1 MAGI1 - membrane associated guanylate kinase, ww and pdz domain containing 1 SNTB2 - syntrophin, beta 2 (dystrophin-associated protein a1, 59kda, basic component 2) MYO5B - myosin vb GAS2L1 - growth arrest-specific 2 like 1 SHROOM2 - shroom family member 2 PARVB - parvin, beta SNTA1 - syntrophin, alpha 1 HOOK2 - hook microtubule-tethering protein 2 CKAP5 - cytoskeleton associated protein 5 TPM3 - tropomyosin 3 TUBGCP4 - tubulin, gamma complex associated protein 4 BCAS3 - breast carcinoma amplified sequence 3 CNN1 - calponin 1, basic, smooth muscle KLHL17 - kelch-like family member 17 DCTN2 - dynactin 2 (p50) FMNL1 - formin-like 1 MYBPC1 - myosin binding protein c, slow type FES - feline sarcoma oncogene MYO16 - myosin xvi LRP8 - low density lipoprotein receptor-related protein 8, apolipoprotein e receptor MYBPC2 - myosin binding protein c, fast type CDK5 - cyclin-dependent kinase 5 CORO1A - coronin, actin binding protein, 1a ATCAY - ataxia, cerebellar, cayman type STMN1 - stathmin 1 ALKBH4 - alkb, alkylation repair homolog 4 (e. coli) MYOM2 - myomesin 2 TBCD - tubulin folding cofactor d KIF21B - kinesin family member 21b PTPN3 - protein tyrosine phosphatase, non-receptor type 3 TNNI3K - tnni3 interacting kinase CDH10 - cadherin 10, type 2 (t2-cadherin) ARL4C - adp-ribosylation factor-like 4c CDH9 - cadherin 9, type 2 (t1-cadherin) TUBGCP2 - tubulin, gamma complex associated protein 2 OBSL1 - obscurin-like 1 CDH13 - cadherin 13, h-cadherin (heart) CDH12 - cadherin 12, type 2 (n-cadherin 2) HTT - huntingtin CDH18 - cadherin 18, type 2 FHOD3 - formin homology 2 domain containing 3 MYO15A - myosin xva TUBGCP6 - tubulin, gamma complex associated protein 6 FABP3 - fatty acid binding protein 3, muscle and heart (mammary-derived growth inhibitor) ATP1A1 - atpase, na+/k+ transporting, alpha 1 polypeptide MICAL3 - microtubule associated monooxygenase, calponin and lim domain containing 3 NME1 - nme/nm23 nucleoside diphosphate kinase 1 KIF3C - kinesin family member 3c PTPRN - protein tyrosine phosphatase, receptor type, n KIF5A - kinesin family member 5a RAB6B - rab6b, member ras oncogene family PICK1 - protein interacting with prkca 1 MAPT - microtubule-associated protein tau MYO19 - myosin xix CLSTN1 - calsyntenin 1 KCNN2 - potassium intermediate/small conductance calcium-activated channel, subfamily n, member 2 MAP1A - microtubule-associated protein 1a PACSIN1 - protein kinase c and casein kinase substrate in neurons 1 PPP5C - protein phosphatase 5, catalytic subunit LMOD3 - leiomodin 3 (fetal) BAIAP2 - bai1-associated protein 2 KCNMA1 - potassium large conductance calcium-activated channel, subfamily m, alpha member 1 STRBP - spermatid perinuclear rna binding protein PEX14 - peroxisomal biogenesis factor 14 STX1A - syntaxin 1a (brain) RAB3A - rab3a, member ras oncogene family DCDC2 - doublecortin domain containing 2 RHCG - rh family, c glycoprotein MKL1 - megakaryoblastic leukemia (translocation) 1 KIFC2 - kinesin family member c2 KIF25 - kinesin family member 25 TNNC2 - troponin c type 2 (fast) RAB11FIP5 - rab11 family interacting protein 5 (class i) FMN1 - formin 1 TNNT3 - troponin t type 3 (skeletal, fast) INO80 - ino80 complex subunit TNNT2 - troponin t type 2 (cardiac) RAB27B - rab27b, member ras oncogene family PRC1 - protein regulator of cytokinesis 1 KCNA2 - potassium voltage-gated channel, shaker-related subfamily, member 2 CRK - v-crk avian sarcoma virus ct10 oncogene homolog VPS18 - vacuolar protein sorting 18 homolog (s. cerevisiae) SHROOM3 - shroom family member 3 CTNNAL1 - catenin (cadherin-associated protein), alpha-like 1 PIN1 - peptidylprolyl cis/trans isomerase, nima-interacting 1 NCK2 - nck adaptor protein 2 GPAA1 - glycosylphosphatidylinositol anchor attachment 1 MICAL2 - microtubule associated monooxygenase, calponin and lim domain containing 2 NEFM - neurofilament, medium polypeptide FSD1 - fibronectin type iii and spry domain containing 1 SYNPO2 - synaptopodin 2 AGBL5 - atp/gtp binding protein-like 5 PKD2L1 - polycystic kidney disease 2-like 1 MYH7B - myosin, heavy chain 7b, cardiac muscle, beta KIF1A - kinesin family member 1a BRSK2 - br serine/threonine kinase 2 SYNPO - synaptopodin PHACTR1 - phosphatase and actin regulator 1 TWF2 - twinfilin actin-binding protein 2 PLEKHM2 - pleckstrin homology domain containing, family m (with run domain) member 2 DYNC1I1 - dynein, cytoplasmic 1, intermediate chain 1 KCNJ11 - potassium inwardly-rectifying channel, subfamily j, member 11 TRIM54 - tripartite motif containing 54 AGBL4 - atp/gtp binding protein-like 4 PRKCE - protein kinase c, epsilon DNM1 - dynamin 1 PKNOX2 - pbx/knotted 1 homeobox 2 GAS8 - growth arrest-specific 8 KCNC1 - potassium voltage-gated channel, shaw-related subfamily, member 1 GSK3A - glycogen synthase kinase 3 alpha ANK1 - ankyrin 1, erythrocytic |
| GO:0005515 | protein binding | 7.01E-5 | 5.85E-3 | 1.08 (16750,10606,1749,1193) | TRMT2B - trna methyltransferase 2 homolog b (s. cerevisiae) DSN1 - dsn1, mis12 kinetochore complex component AGAP3 - arfgap with gtpase domain, ankyrin repeat and ph domain 3 LRRC16A - leucine rich repeat containing 16a SEPT5 - septin 5 GRHL2 - grainyhead-like 2 (drosophila) SH2D1B - sh2 domain containing 1b PDIA5 - protein disulfide isomerase family a, member 5 CHAF1B - chromatin assembly factor 1, subunit b (p60) PNOC - prepronociceptin LZTR1 - leucine-zipper-like transcription regulator 1 GDF1 - growth differentiation factor 1 MLLT11 - myeloid/lymphoid or mixed-lineage leukemia (trithorax homolog, drosophila); translocated to, 11 DPF1 - d4, zinc and double phd fingers family 1 ACOT2 - acyl-coa thioesterase 2 PMM1 - phosphomannomutase 1 GCKR - glucokinase (hexokinase 4) regulator KAT2A - k(lysine) acetyltransferase 2a NCOA3 - nuclear receptor coactivator 3 ASIC2 - acid-sensing (proton-gated) ion channel 2 GLA - galactosidase, alpha PLS1 - plastin 1 GADD45G - growth arrest and dna-damage-inducible, gamma TWIST2 - twist basic helix-loop-helix transcription factor 2 PLXNA2 - plexin a2 BTBD2 - btb (poz) domain containing 2 OLFM3 - olfactomedin 3 OTUB1 - otu domain, ubiquitin aldehyde binding 1 RAB37 - rab37, member ras oncogene family UBE2J2 - ubiquitin-conjugating enzyme e2, j2 PLCG1 - phospholipase c, gamma 1 GAN - gigaxonin GRASP - grp1 (general receptor for phosphoinositides 1)-associated scaffold protein GGT7 - gamma-glutamyltransferase 7 PPID - peptidylprolyl isomerase d NMU - neuromedin u PPEF1 - protein phosphatase, ef-hand calcium binding domain 1 PPIA - peptidylprolyl isomerase a (cyclophilin a) USP19 - ubiquitin specific peptidase 19 PPL - periplakin PARL - presenilin associated, rhomboid-like GLS - glutaminase PPM1B - protein phosphatase, mg2+/mn2+ dependent, 1b C1QL1 - complement component 1, q subcomponent-like 1 ZMYND19 - zinc finger, mynd-type containing 19 GLDC - glycine dehydrogenase (decarboxylating) PIDD - p53-induced death domain protein NPFFR2 - neuropeptide ff receptor 2 ADCY6 - adenylate cyclase 6 CPSF4 - cleavage and polyadenylation specific factor 4, 30kda RUNDC3A - run domain containing 3a BAP1 - brca1 associated protein-1 (ubiquitin carboxy-terminal hydrolase) AXIN2 - axin 2 NUP85 - nucleoporin 85kda GRIN3A - glutamate receptor, ionotropic, n-methyl-d-aspartate 3a ADCY2 - adenylate cyclase 2 (brain) GNG3 - guanine nucleotide binding protein (g protein), gamma 3 FEM1A - fem-1 homolog a (c. elegans) TNIP3 - tnfaip3 interacting protein 3 CCL27 - chemokine (c-c motif) ligand 27 LAGE3 - l antigen family, member 3 GNA15 - guanine nucleotide binding protein (g protein), alpha 15 (gq class) POLD2 - polymerase (dna directed), delta 2, accessory subunit GNA11 - guanine nucleotide binding protein (g protein), alpha 11 (gq class) SNAP47 - synaptosomal-associated protein, 47kda POLD1 - polymerase (dna directed), delta 1, catalytic subunit HES6 - hairy and enhancer of split 6 (drosophila) ADORA1 - adenosine a1 receptor F8A1 - coagulation factor viii-associated 1 TUBGCP2 - tubulin, gamma complex associated protein 2 PDZD7 - pdz domain containing 7 SYNDIG1 - synapse differentiation inducing 1 ADRA1B - adrenoceptor alpha 1b POLR2E - polymerase (rna) ii (dna directed) polypeptide e, 25kda ADRA1D - adrenoceptor alpha 1d RUVBL2 - ruvb-like 2 (e. coli) CNST - consortin, connexin sorting protein PRKAA2 - protein kinase, amp-activated, alpha 2 catalytic subunit AP1B1 - adaptor-related protein complex 1, beta 1 subunit PRKAB1 - protein kinase, amp-activated, beta 1 non-catalytic subunit ADRB1 - adrenoceptor beta 1 PREP - prolyl endopeptidase ADRBK1 - adrenergic, beta, receptor kinase 1 ADAMTS8 - adam metallopeptidase with thrombospondin type 1 motif, 8 PTPRT - protein tyrosine phosphatase, receptor type, t ARFGAP1 - adp-ribosylation factor gtpase activating protein 1 PPP3R1 - protein phosphatase 3, regulatory subunit b, alpha CIT - citron (rho-interacting, serine/threonine kinase 21) PPP5C - protein phosphatase 5, catalytic subunit ARHGEF25 - rho guanine nucleotide exchange factor (gef) 25 FZD3 - frizzled family receptor 3 LRIF1 - ligand dependent nuclear receptor interacting factor 1 PPP2R5B - protein phosphatase 2, regulatory subunit b', beta SPTSSB - serine palmitoyltransferase, small subunit b ZNF653 - zinc finger protein 653 PCID2 - pci domain containing 2 USP7 - ubiquitin specific peptidase 7 (herpes virus-associated) XRCC6 - x-ray repair complementing defective repair in chinese hamster cells 6 OGFR - opioid growth factor receptor GABRA3 - gamma-aminobutyric acid (gaba) a receptor, alpha 3 ALDH3A1 - aldehyde dehydrogenase 3 family, member a1 RER1 - rer1 retention in endoplasmic reticulum 1 homolog (s. cerevisiae) KLHDC3 - kelch domain containing 3 BAG6 - bcl2-associated athanogene 6 NUDT18 - nudix (nucleoside diphosphate linked moiety x)-type motif 18 IFT122 - intraflagellar transport 122 homolog (chlamydomonas) PPM1G - protein phosphatase, mg2+/mn2+ dependent, 1g RAPGEF4 - rap guanine nucleotide exchange factor (gef) 4 ALAS1 - aminolevulinate, delta-, synthase 1 TMEM115 - transmembrane protein 115 ABHD16A - abhydrolase domain containing 16a RABL6 - rab, member ras oncogene family-like 6 RNF165 - ring finger protein 165 GAK - cyclin g associated kinase PRPS2 - phosphoribosyl pyrophosphate synthetase 2 PRPS1 - phosphoribosyl pyrophosphate synthetase 1 GPRIN1 - g protein regulated inducer of neurite outgrowth 1 PNMA5 - paraneoplastic ma antigen family member 5 MAP2K1 - mitogen-activated protein kinase kinase 1 SORCS1 - sortilin-related vps10 domain containing receptor 1 MAP2K2 - mitogen-activated protein kinase kinase 2 GAD1 - glutamate decarboxylase 1 (brain, 67kda) MAPK10 - mitogen-activated protein kinase 10 FBXO32 - f-box protein 32 CEP72 - centrosomal protein 72kda MAPK11 - mitogen-activated protein kinase 11 KATNBL1 - katanin p80 subunit b-like 1 MAPK3 - mitogen-activated protein kinase 3 LMBR1L - limb development membrane protein 1-like CCNI - cyclin i PRKG2 - protein kinase, cgmp-dependent, type ii AMPH - amphiphysin PRKCE - protein kinase c, epsilon PRKCB - protein kinase c, beta GAP43 - growth associated protein 43 HPS6 - hermansky-pudlak syndrome 6 GAS8 - growth arrest-specific 8 SLC27A2 - solute carrier family 27 (fatty acid transporter), member 2 SLC25A5 - solute carrier family 25 (mitochondrial carrier; adenine nucleotide translocator), member 5 ANK1 - ankyrin 1, erythrocytic MTPAP - mitochondrial poly(a) polymerase C22orf29 - chromosome 22 open reading frame 29 GTF2F1 - general transcription factor iif, polypeptide 1, 74kda NXPH2 - neurexophilin 2 ZNF148 - zinc finger protein 148 BEND5 - ben domain containing 5 ANXA6 - annexin a6 NXPH3 - neurexophilin 3 NEIL1 - nei endonuclease viii-like 1 (e. coli) RMDN3 - regulator of microtubule dynamics 3 DCTN3 - dynactin 3 (p22) TRIM25 - tripartite motif containing 25 APBA2 - amyloid beta (a4) precursor protein-binding, family a, member 2 ZBTB16 - zinc finger and btb domain containing 16 HEXIM2 - hexamethylene bis-acetamide inducible 2 ZNF124 - zinc finger protein 124 LRRK1 - leucine-rich repeat kinase 1 GTF3C1 - general transcription factor iiic, polypeptide 1, alpha 220kda ZMAT4 - zinc finger, matrin-type 4 PAK1 - p21 protein (cdc42/rac)-activated kinase 1 SCAI - suppressor of cancer cell invasion SHROOM2 - shroom family member 2 ARNTL - aryl hydrocarbon receptor nuclear translocator-like C14orf79 - chromosome 14 open reading frame 79 RNF31 - ring finger protein 31 BRPF1 - bromodomain and phd finger containing, 1 NISCH - nischarin H2AFZ - h2a histone family, member z PKP3 - plakophilin 3 TUBA1A - tubulin, alpha 1a HIST1H1D - histone cluster 1, h1d IFT57 - intraflagellar transport 57 homolog (chlamydomonas) PDPK1 - 3-phosphoinositide dependent protein kinase-1 ARG1 - arginase 1 ANO1 - anoctamin 1, calcium activated chloride channel ANKRD9 - ankyrin repeat domain 9 HAGH - hydroxyacylglutathione hydrolase SERPINF1 - serpin peptidase inhibitor, clade f (alpha-2 antiplasmin, pigment epithelium derived factor), member 1 LRP8 - low density lipoprotein receptor-related protein 8, apolipoprotein e receptor HIST1H1A - histone cluster 1, h1a WDR60 - wd repeat domain 60 ASNA1 - arsa arsenite transporter, atp-binding, homolog 1 (bacterial) CORO1A - coronin, actin binding protein, 1a ACOT4 - acyl-coa thioesterase 4 SLC30A3 - solute carrier family 30 (zinc transporter), member 3 PCSK2 - proprotein convertase subtilisin/kexin type 2 CRTAC1 - cartilage acidic protein 1 ASS1 - argininosuccinate synthase 1 ZNF232 - zinc finger protein 232 C14orf1 - chromosome 14 open reading frame 1 PCSK1 - proprotein convertase subtilisin/kexin type 1 LARP1B - la ribonucleoprotein domain family, member 1b PDE1A - phosphodiesterase 1a, calmodulin-dependent PDE2A - phosphodiesterase 2a, cgmp-stimulated SNRNP25 - small nuclear ribonucleoprotein 25kda (u11/u12) HTT - huntingtin ASGR2 - asialoglycoprotein receptor 2 PDE4A - phosphodiesterase 4a, camp-specific TMEM132D - transmembrane protein 132d RHEBL1 - ras homolog enriched in brain like 1 ATP1A1 - atpase, na+/k+ transporting, alpha 1 polypeptide FBXW7 - f-box and wd repeat domain containing 7, e3 ubiquitin protein ligase CDK14 - cyclin-dependent kinase 14 RPH3A - rabphilin 3a homolog (mouse) WDR25 - wd repeat domain 25 WNT10B - wingless-type mmtv integration site family, member 10b MORC2 - morc family cw-type zinc finger 2 CLSTN1 - calsyntenin 1 GPM6A - glycoprotein m6a STRBP - spermatid perinuclear rna binding protein PEX14 - peroxisomal biogenesis factor 14 PEPD - peptidase d NTNG1 - netrin g1 C19orf66 - chromosome 19 open reading frame 66 BTBD11 - btb (poz) domain containing 11 PER1 - period circadian clock 1 WNT7A - wingless-type mmtv integration site family, member 7a STYK1 - serine/threonine/tyrosine kinase 1 VSNL1 - visinin-like 1 PFKP - phosphofructokinase, platelet ATP2B4 - atpase, ca++ transporting, plasma membrane 4 METTL21C - methyltransferase like 21c ATP2B3 - atpase, ca++ transporting, plasma membrane 3 ATP8B1 - atpase, aminophospholipid transporter, class i, type 8b, member 1 EFHD2 - ef-hand domain family, member d2 GRK6 - g protein-coupled receptor kinase 6 FBXL15 - f-box and leucine-rich repeat protein 15 GPX1 - glutathione peroxidase 1 BAD - bcl2-associated agonist of cell death PIN1 - peptidylprolyl cis/trans isomerase, nima-interacting 1 GPS1 - g protein pathway suppressor 1 LRRC20 - leucine rich repeat containing 20 PI4KA - phosphatidylinositol 4-kinase, catalytic, alpha FSD1 - fibronectin type iii and spry domain containing 1 PIK3R1 - phosphoinositide-3-kinase, regulatory subunit 1 (alpha) SYNPO2 - synaptopodin 2 AUP1 - ancient ubiquitous protein 1 ZFYVE27 - zinc finger, fyve domain containing 27 KIF1A - kinesin family member 1a PEF1 - penta-ef-hand domain containing 1 SYNPO - synaptopodin SLC38A7 - solute carrier family 38, member 7 RHOV - ras homolog family member v TWF2 - twinfilin actin-binding protein 2 IMP3 - imp3, u3 small nucleolar ribonucleoprotein, homolog (yeast) GRIN2A - glutamate receptor, ionotropic, n-methyl d-aspartate 2a ATXN7L3B - ataxin 7-like 3b VPS53 - vacuolar protein sorting 53 homolog (s. cerevisiae) MGLL - monoglyceride lipase GRIK2 - glutamate receptor, ionotropic, kainate 2 ACOT7 - acyl-coa thioesterase 7 GRM2 - glutamate receptor, metabotropic 2 PHKG2 - phosphorylase kinase, gamma 2 (testis) RPUSD3 - rna pseudouridylate synthase domain containing 3 PIK3CD - phosphatidylinositol-4,5-bisphosphate 3-kinase, catalytic subunit delta PIM1 - pim-1 oncogene GSK3A - glycogen synthase kinase 3 alpha PGAM5 - phosphoglycerate mutase family member 5 B4GALT7 - xylosylprotein beta 1,4-galactosyltransferase, polypeptide 7 RFC2 - replication factor c (activator 1) 2, 40kda LRRC45 - leucine rich repeat containing 45 TSHZ2 - teashirt zinc finger homeobox 2 SEMA4C - sema domain, immunoglobulin domain (ig), transmembrane domain (tm) and short cytoplasmic domain, (semaphorin) 4c RGR - retinal g protein coupled receptor BCKDK - branched chain ketoacid dehydrogenase kinase MKS1 - meckel syndrome, type 1 APBB3 - amyloid beta (a4) precursor protein-binding, family b, member 3 BTC - betacellulin HRG - histidine-rich glycoprotein FGF17 - fibroblast growth factor 17 RET - ret proto-oncogene PHF23 - phd finger protein 23 FGF18 - fibroblast growth factor 18 CCNO - cyclin o HPS1 - hermansky-pudlak syndrome 1 BDNF - brain-derived neurotrophic factor SPRED2 - sprouty-related, evh1 domain containing 2 HPCAL1 - hippocalcin-like 1 BID - bh3 interacting domain death agonist RGS7 - regulator of g-protein signaling 7 BMP2 - bone morphogenetic protein 2 PCGF3 - polycomb group ring finger 3 SYTL2 - synaptotagmin-like 2 RGS4 - regulator of g-protein signaling 4 OSTN - osteocrin BMP8B - bone morphogenetic protein 8b RHO - rhodopsin CTDSPL - ctd (carboxy-terminal domain, rna polymerase ii, polypeptide a) small phosphatase-like BCAS3 - breast carcinoma amplified sequence 3 FMNL1 - formin-like 1 C21orf2 - chromosome 21 open reading frame 2 MYBPHL - myosin binding protein h-like CCNA1 - cyclin a1 CA4 - carbonic anhydrase iv ZFYVE21 - zinc finger, fyve domain containing 21 HTR1A - 5-hydroxytryptamine (serotonin) receptor 1a, g protein-coupled HTR2A - 5-hydroxytryptamine (serotonin) receptor 2a, g protein-coupled EIF2B5 - eukaryotic translation initiation factor 2b, subunit 5 epsilon, 82kda TIMM17B - translocase of inner mitochondrial membrane 17 homolog b (yeast) PPP1R12C - protein phosphatase 1, regulatory subunit 12c POP7 - processing of precursor 7, ribonuclease p/mrp subunit (s. cerevisiae) RORB - rar-related orphan receptor b ALKBH4 - alkb, alkylation repair homolog 4 (e. coli) FOXH1 - forkhead box h1 TRPM4 - transient receptor potential cation channel, subfamily m, member 4 CACNA1G - calcium channel, voltage-dependent, t type, alpha 1g subunit LRFN4 - leucine rich repeat and fibronectin type iii domain containing 4 RAMP3 - receptor (g protein-coupled) activity modifying protein 3 AUNIP - aurora kinase a and ninein interacting protein DUSP26 - dual specificity phosphatase 26 (putative) SKAP2 - src kinase associated phosphoprotein 2 OSGIN2 - oxidative stress induced growth inhibitor family member 2 BAIAP3 - bai1-associated protein 3 CAPNS1 - calpain, small subunit 1 PPIE - peptidylprolyl isomerase e (cyclophilin e) MCRS1 - microspherule protein 1 CAMK2G - calcium/calmodulin-dependent protein kinase ii gamma C19orf47 - chromosome 19 open reading frame 47 OLFM1 - olfactomedin 1 PLEKHB2 - pleckstrin homology domain containing, family b (evectins) member 2 EPN3 - epsin 3 CAMK2A - calcium/calmodulin-dependent protein kinase ii alpha CHRD - chordin CASQ1 - calsequestrin 1 (fast-twitch, skeletal muscle) TIMM44 - translocase of inner mitochondrial membrane 44 homolog (yeast) FBXO34 - f-box protein 34 KLHDC9 - kelch domain containing 9 CARS - cysteinyl-trna synthetase AACS - acetoacetyl-coa synthetase RPA2 - replication protein a2, 32kda TNFAIP8L1 - tumor necrosis factor, alpha-induced protein 8-like 1 CAD - carbamoyl-phosphate synthetase 2, aspartate transcarbamylase, and dihydroorotase PINK1 - pten induced putative kinase 1 HK1 - hexokinase 1 CACNB4 - calcium channel, voltage-dependent, beta 4 subunit SLC26A6 - solute carrier family 26 (anion exchanger), member 6 NXF1 - nuclear rna export factor 1 UBE2E3 - ubiquitin-conjugating enzyme e2e 3 PIH1D1 - pih1 domain containing 1 STAC2 - sh3 and cysteine rich domain 2 ANKS6 - ankyrin repeat and sterile alpha motif domain containing 6 CALML3 - calmodulin-like 3 FBXO27 - f-box protein 27 NRG1 - neuregulin 1 BSCL2 - berardinelli-seip congenital lipodystrophy 2 (seipin) AURKAIP1 - aurora kinase a interacting protein 1 FMN1 - formin 1 STK25 - serine/threonine kinase 25 HIVEP1 - human immunodeficiency virus type i enhancer binding protein 1 CRTAP - cartilage associated protein C12orf43 - chromosome 12 open reading frame 43 ADCK5 - aarf domain containing kinase 5 CACNG2 - calcium channel, voltage-dependent, gamma subunit 2 CACNG3 - calcium channel, voltage-dependent, gamma subunit 3 CLN6 - ceroid-lipofuscinosis, neuronal 6, late infantile, variant TUBA1B - tubulin, alpha 1b SEMA3A - sema domain, immunoglobulin domain (ig), short basic domain, secreted, (semaphorin) 3a CCNF - cyclin f ADAM19 - adam metallopeptidase domain 19 CCNE1 - cyclin e1 CTNNAL1 - catenin (cadherin-associated protein), alpha-like 1 BEX5 - brain expressed, x-linked 5 GPAA1 - glycosylphosphatidylinositol anchor attachment 1 CPSF3L - cleavage and polyadenylation specific factor 3-like CCNG2 - cyclin g2 NPM2 - nucleophosmin/nucleoplasmin 2 GBF1 - golgi brefeldin a resistant guanine nucleotide exchange factor 1 TNFSF12 - tumor necrosis factor (ligand) superfamily, member 12 UCKL1 - uridine-cytidine kinase 1-like 1 FBXO6 - f-box protein 6 VPS33B - vacuolar protein sorting 33 homolog b (yeast) ADAM23 - adam metallopeptidase domain 23 CD3G - cd3g molecule, gamma (cd3-tcr complex) SYCP2 - synaptonemal complex protein 2 RSPO2 - r-spondin 2 KHDRBS2 - kh domain containing, rna binding, signal transduction associated 2 SLC41A3 - solute carrier family 41, member 3 TNFRSF14 - tumor necrosis factor receptor superfamily, member 14 TNFRSF6B - tumor necrosis factor receptor superfamily, member 6b, decoy EXD3 - exonuclease 3'-5' domain containing 3 RTN2 - reticulon 2 RXRB - retinoid x receptor, beta CCKBR - cholecystokinin b receptor TMEM161A - transmembrane protein 161a CCND2 - cyclin d2 RGS11 - regulator of g-protein signaling 11 RTN1 - reticulon 1 LHX6 - lim homeobox 6 SPON2 - spondin 2, extracellular matrix protein TNFRSF11A - tumor necrosis factor receptor superfamily, member 11a, nfkb activator UBAC1 - uba domain containing 1 CDIPT - cdp-diacylglycerol--inositol 3-phosphatidyltransferase CCK - cholecystokinin CDC6 - cell division cycle 6 PRSS3 - protease, serine, 3 RMND5A - required for meiotic nuclear division 5 homolog a (s. cerevisiae) LGMN - legumain SSH1 - slingshot protein phosphatase 1 MXD4 - max dimerization protein 4 IL12RB2 - interleukin 12 receptor, beta 2 ENC1 - ectodermal-neural cortex 1 (with btb domain) SPAG5 - sperm associated antigen 5 SGSM3 - small g protein signaling modulator 3 INCENP - inner centromere protein antigens 135/155kda FBXW5 - f-box and wd repeat domain containing 5 PSD - pleckstrin and sec7 domain containing PSEN2 - presenilin 2 (alzheimer disease 4) TOLLIP - toll interacting protein IMPA2 - inositol(myo)-1(or 4)-monophosphatase 2 CDC42EP3 - cdc42 effector protein (rho gtpase binding) 3 KCNMB4 - potassium large conductance calcium-activated channel, subfamily m, beta member 4 PPFIA4 - protein tyrosine phosphatase, receptor type, f polypeptide (ptprf), interacting protein (liprin), alpha 4 PRPF19 - pre-mrna processing factor 19 BHLHE40 - basic helix-loop-helix family, member e40 PCLO - piccolo presynaptic cytomatrix protein KHDRBS3 - kh domain containing, rna binding, signal transduction associated 3 SMAP2 - small arfgap2 SNTG1 - syntrophin, gamma 1 LMO4 - lim domain only 4 PPFIA3 - protein tyrosine phosphatase, receptor type, f polypeptide (ptprf), interacting protein (liprin), alpha 3 UBE2QL1 - ubiquitin-conjugating enzyme e2q family-like 1 YIPF1 - yip1 domain family, member 1 LSM11 - lsm11, u7 small nuclear rna associated GAS2L1 - growth arrest-specific 2 like 1 POSTN - periostin, osteoblast specific factor PSMD2 - proteasome (prosome, macropain) 26s subunit, non-atpase, 2 IL6R - interleukin 6 receptor DGKZ - diacylglycerol kinase, zeta PPARGC1B - peroxisome proliferator-activated receptor gamma, coactivator 1 beta ITGA9 - integrin, alpha 9 ATG7 - autophagy related 7 TUBGCP4 - tubulin, gamma complex associated protein 4 DCTN2 - dynactin 2 (p50) KLHL17 - kelch-like family member 17 IRS1 - insulin receptor substrate 1 CENPF - centromere protein f, 350/400kda RRP1 - ribosomal rna processing 1 PTGS2 - prostaglandin-endoperoxide synthase 2 (prostaglandin g/h synthase and cyclooxygenase) NARFL - nuclear prelamin a recognition factor-like GPKOW - g patch domain and kow motifs PTH2R - parathyroid hormone 2 receptor THOC5 - tho complex 5 CDK5 - cyclin-dependent kinase 5 RND1 - rho family gtpase 1 CDK9 - cyclin-dependent kinase 9 MRPL28 - mitochondrial ribosomal protein l28 MTMR14 - myotubularin related protein 14 CDKN3 - cyclin-dependent kinase inhibitor 3 CDKN2D - cyclin-dependent kinase inhibitor 2d (p19, inhibits cdk4) PTPN3 - protein tyrosine phosphatase, non-receptor type 3 CDKN2C - cyclin-dependent kinase inhibitor 2c (p18, inhibits cdk4) PSMG1 - proteasome (prosome, macropain) assembly chaperone 1 PTPN9 - protein tyrosine phosphatase, non-receptor type 9 IRF3 - interferon regulatory factor 3 CDH10 - cadherin 10, type 2 (t2-cadherin) CDH9 - cadherin 9, type 2 (t1-cadherin) CDH13 - cadherin 13, h-cadherin (heart) CDH12 - cadherin 12, type 2 (n-cadherin 2) ARHGAP9 - rho gtpase activating protein 9 CDH18 - cadherin 18, type 2 PTPRF - protein tyrosine phosphatase, receptor type, f RTP1 - receptor (chemosensory) transporter protein 1 PVALB - parvalbumin GTPBP2 - gtp binding protein 2 CHRNA7 - cholinergic receptor, nicotinic, alpha 7 (neuronal) BBC3 - bcl2 binding component 3 ARPP21 - camp-regulated phosphoprotein, 21kda PTPRR - protein tyrosine phosphatase, receptor type, r CHRM3 - cholinergic receptor, muscarinic 3 LNX2 - ligand of numb-protein x 2 WDR74 - wd repeat domain 74 SDCBP2 - syndecan binding protein (syntenin) 2 PTPRN - protein tyrosine phosphatase, receptor type, n MMS19 - mms19 nucleotide excision repair homolog (s. cerevisiae) CHML - choroideremia-like (rab escort protein 2) INPP5J - inositol polyphosphate-5-phosphatase j PEX5 - peroxisomal biogenesis factor 5 DKK2 - dickkopf wnt signaling pathway inhibitor 2 CHN1 - chimerin 1 CHL1 - cell adhesion molecule l1-like PDLIM2 - pdz and lim domain 2 (mystique) PVRL1 - poliovirus receptor-related 1 (herpesvirus entry mediator c) PVR - poliovirus receptor SLC6A20 - solute carrier family 6 (proline imino transporter), member 20 KCNH5 - potassium voltage-gated channel, subfamily h (eag-related), member 5 MLST8 - mtor associated protein, lst8 homolog (s. cerevisiae) PVRL2 - poliovirus receptor-related 2 (herpesvirus entry mediator b) ID2 - inhibitor of dna binding 2, dominant negative helix-loop-helix protein BRPF3 - bromodomain and phd finger containing, 3 MAP4K2 - mitogen-activated protein kinase kinase kinase kinase 2 CPNE4 - copine iv FRS3 - fibroblast growth factor receptor substrate 3 RAB3A - rab3a, member ras oncogene family GLS2 - glutaminase 2 (liver, mitochondrial) RGL2 - ral guanine nucleotide dissociation stimulator-like 2 PLA2G6 - phospholipase a2, group vi (cytosolic, calcium-independent) NOXA1 - nadph oxidase activator 1 PIP5K1B - phosphatidylinositol-4-phosphate 5-kinase, type i, beta IL36B - interleukin 36, beta RAD23A - rad23 homolog a (s. cerevisiae) HIST1H4L - histone cluster 1, h4l FOXK1 - forkhead box k1 MAGEF1 - melanoma antigen family f, 1 ZFAND2B - zinc finger, an1-type domain 2b RAC2 - ras-related c3 botulinum toxin substrate 2 (rho family, small gtp binding protein rac2) INO80 - ino80 complex subunit RAB27B - rab27b, member ras oncogene family NDFIP2 - nedd4 family interacting protein 2 VSTM2A - v-set and transmembrane domain containing 2a HYAL3 - hyaluronoglucosaminidase 3 RSPH9 - radial spoke head 9 homolog (chlamydomonas) CNPY3 - canopy fgf signaling regulator 3 LRRC4 - leucine rich repeat containing 4 NCK2 - nck adaptor protein 2 AKAP8L - a kinase (prka) anchor protein 8-like TRUB2 - trub pseudouridine (psi) synthase homolog 2 (e. coli) SGTB - small glutamine-rich tetratricopeptide repeat (tpr)-containing, beta RARB - retinoic acid receptor, beta CLTA - clathrin, light chain a LHPP - phospholysine phosphohistidine inorganic pyrophosphate phosphatase RBM24 - rna binding motif protein 24 PHACTR1 - phosphatase and actin regulator 1 CLK2 - cdc-like kinase 2 CLSPN - claspin NEUROD6 - neuronal differentiation 6 CLCN7 - chloride channel, voltage-sensitive 7 IGFBP2 - insulin-like growth factor binding protein 2, 36kda AP2S1 - adaptor-related protein complex 2, sigma 1 subunit SEMA7A - semaphorin 7a, gpi membrane anchor (john milton hagen blood group) HIRIP3 - hira interacting protein 3 RASGRF2 - ras protein-specific guanine nucleotide-releasing factor 2 RFPL3 - ret finger protein-like 3 DHX16 - deah (asp-glu-ala-his) box polypeptide 16 DOC2A - double c2-like domains, alpha ANKRD13D - ankyrin repeat domain 13 family, member d TPST2 - tyrosylprotein sulfotransferase 2 NECAB3 - n-terminal ef-hand calcium binding protein 3 CIDEA - cell death-inducing dffa-like effector a RHOF - ras homolog family member f (in filopodia) TBR1 - t-box, brain, 1 TRIB3 - tribbles homolog 3 (drosophila) SH3BP1 - sh3-domain binding protein 1 MKRN1 - makorin ring finger protein 1 SPTBN5 - spectrin, beta, non-erythrocytic 5 PRR16 - proline rich 16 LIMK1 - lim domain kinase 1 FAM73B - family with sequence similarity 73, member b SPINK2 - serine peptidase inhibitor, kazal type 2 (acrosin-trypsin inhibitor) LDLR - low density lipoprotein receptor SPG7 - spastic paraplegia 7 (pure and complicated autosomal recessive) LCP2 - lymphocyte cytosolic protein 2 (sh2 domain containing leukocyte protein of 76kda) CARTPT - cart prepropeptide REEP2 - receptor accessory protein 2 RAB11FIP4 - rab11 family interacting protein 4 (class ii) IQSEC3 - iq motif and sec7 domain 3 UAP1 - udp-n-acteylglucosamine pyrophosphorylase 1 CADM3 - cell adhesion molecule 3 L3MBTL3 - l(3)mbt-like 3 (drosophila) FAM69B - family with sequence similarity 69, member b BMP8A - bone morphogenetic protein 8a POLN - polymerase (dna directed) nu RAB3D - rab3d, member ras oncogene family BRSK1 - br serine/threonine kinase 1 SOX5 - sry (sex determining region y)-box 5 NEURL4 - neuralized homolog 4 (drosophila) LCOR - ligand dependent nuclear receptor corepressor IGSF21 - immunoglobin superfamily, member 21 CRHBP - corticotropin releasing hormone binding protein FBXL20 - f-box and leucine-rich repeat protein 20 GABBR2 - gamma-aminobutyric acid (gaba) b receptor, 2 ORC6 - origin recognition complex, subunit 6 LIPC - lipase, hepatic CRH - corticotropin releasing hormone MAGI3 - membrane associated guanylate kinase, ww and pdz domain containing 3 HOPX - hop homeobox NUDCD1 - nudc domain containing 1 SNTB2 - syntrophin, beta 2 (dystrophin-associated protein a1, 59kda, basic component 2) SMUG1 - single-strand-selective monofunctional uracil-dna glycosylase 1 SNTA1 - syntrophin, alpha 1 NR1D1 - nuclear receptor subfamily 1, group d, member 1 RHOXF2 - rhox homeobox family, member 2 KRT31 - keratin 31 KRT33B - keratin 33b FXR2 - fragile x mental retardation, autosomal homolog 2 CNN1 - calponin 1, basic, smooth muscle STXBP5L - syntaxin binding protein 5-like HPCAL4 - hippocalcin like 4 TMEM185A - transmembrane protein 185a KRT86 - keratin 86 SNRPA1 - small nuclear ribonucleoprotein polypeptide a' NGB - neuroglobin PIM3 - pim-3 oncogene KRT14 - keratin 14 UBL7 - ubiquitin-like 7 (bone marrow stromal cell-derived) STX19 - syntaxin 19 NT5C1A - 5'-nucleotidase, cytosolic ia HEMK1 - hemk methyltransferase family member 1 COL7A1 - collagen, type vii, alpha 1 SSBP3 - single stranded dna binding protein 3 IL27RA - interleukin 27 receptor, alpha STMN1 - stathmin 1 ATCAY - ataxia, cerebellar, cayman type THEMIS2 - thymocyte selection associated family member 2 NTNG2 - netrin g2 SMARCC2 - swi/snf related, matrix associated, actin dependent regulator of chromatin, subfamily c, member 2 PNCK - pregnancy up-regulated non-ubiquitously expressed cam kinase SLN - sarcolipin MED20 - mediator complex subunit 20 AFAP1L2 - actin filament associated protein 1-like 2 COL4A2 - collagen, type iv, alpha 2 DMRT3 - doublesex and mab-3 related transcription factor 3 SLIT1 - slit homolog 1 (drosophila) COL5A2 - collagen, type v, alpha 2 PLD3 - phospholipase d family, member 3 LCN1 - lipocalin 1 SLPI - secretory leukocyte peptidase inhibitor CABP1 - calcium binding protein 1 DACT1 - dishevelled-binding antagonist of beta-catenin 1 LAG3 - lymphocyte-activation gene 3 COL19A1 - collagen, type xix, alpha 1 AKAP5 - a kinase (prka) anchor protein 5 LAIR2 - leukocyte-associated immunoglobulin-like receptor 2 LAMB3 - laminin, beta 3 COL13A1 - collagen, type xiii, alpha 1 MAGED1 - melanoma antigen family d, 1 MYO15A - myosin xva TFIP11 - tuftelin interacting protein 11 NAGPA - n-acetylglucosamine-1-phosphodiester alpha-n-acetylglucosaminidase SYP - synaptophysin RXFP1 - relaxin/insulin-like family peptide receptor 1 EGFL7 - egf-like-domain, multiple 7 TAC3 - tachykinin 3 KIF3C - kinesin family member 3c SYT1 - synaptotagmin i KIF5A - kinesin family member 5a NTN4 - netrin 4 ZNF541 - zinc finger protein 541 ABCC8 - atp-binding cassette, sub-family c (cftr/mrp), member 8 PICK1 - protein interacting with prkca 1 KHK - ketohexokinase (fructokinase) KCNS1 - potassium voltage-gated channel, delayed-rectifier, subfamily s, member 1 HOMER1 - homer homolog 1 (drosophila) SPATA22 - spermatogenesis associated 22 KCNN2 - potassium intermediate/small conductance calcium-activated channel, subfamily n, member 2 KCNK3 - potassium channel, subfamily k, member 3 KCNMA1 - potassium large conductance calcium-activated channel, subfamily m, alpha member 1 MED22 - mediator complex subunit 22 KCNN1 - potassium intermediate/small conductance calcium-activated channel, subfamily n, member 1 SURF6 - surfeit 6 CTSK - cathepsin k TRIM17 - tripartite motif containing 17 CTSD - cathepsin d STX1A - syntaxin 1a (brain) STOML1 - stomatin (epb72)-like 1 LNX1 - ligand of numb-protein x 1, e3 ubiquitin protein ligase FLRT2 - fibronectin leucine rich transmembrane protein 2 FLRT3 - fibronectin leucine rich transmembrane protein 3 GABARAPL3 - gaba(a) receptors associated protein like 3, pseudogene WDR24 - wd repeat domain 24 NUDT14 - nudix (nucleoside diphosphate linked moiety x)-type motif 14 KIF25 - kinesin family member 25 AVPI1 - arginine vasopressin-induced 1 C20orf112 - chromosome 20 open reading frame 112 CYB561 - cytochrome b561 CAMKK1 - calcium/calmodulin-dependent protein kinase kinase 1, alpha APOL2 - apolipoprotein l, 2 FAM19A1 - family with sequence similarity 19 (chemokine (c-c motif)-like), member a1 MTCH1 - mitochondrial carrier 1 MED10 - mediator complex subunit 10 STK11 - serine/threonine kinase 11 CRIP2 - cysteine-rich protein 2 LONP1 - lon peptidase 1, mitochondrial KCNA2 - potassium voltage-gated channel, shaker-related subfamily, member 2 SULT4A1 - sulfotransferase family 4a, member 1 KCNA1 - potassium voltage-gated channel, shaker-related subfamily, member 1 (episodic ataxia with myokymia) NRSN1 - neurensin 1 RAB26 - rab26, member ras oncogene family STAT4 - signal transducer and activator of transcription 4 FBXW9 - f-box and wd repeat domain containing 9 SLIT2 - slit homolog 2 (drosophila) LHX2 - lim homeobox 2 YIPF3 - yip1 domain family, member 3 STAT6 - signal transducer and activator of transcription 6, interleukin-4 induced CRYBB1 - crystallin, beta b1 EIF1AD - eukaryotic translation initiation factor 1a domain containing AGBL5 - atp/gtp binding protein-like 5 TECPR1 - tectonin beta-propeller repeat containing 1 JAG2 - jagged 2 ZDHHC16 - zinc finger, dhhc-type containing 16 DCAF12 - ddb1 and cul4 associated factor 12 SSTR2 - somatostatin receptor 2 CRYGD - crystallin, gamma d PRADC1 - protease-associated domain containing 1 ITPKA - inositol-trisphosphate 3-kinase a RIC8A - ric8 guanine nucleotide exchange factor a ITPR1 - inositol 1,4,5-trisphosphate receptor, type 1 PHF20 - phd finger protein 20 CHCHD6 - coiled-coil-helix-coiled-coil-helix domain containing 6 GPATCH3 - g patch domain containing 3 KCNJ12 - potassium inwardly-rectifying channel, subfamily j, member 12 NGEF - neuronal guanine nucleotide exchange factor KCNJ11 - potassium inwardly-rectifying channel, subfamily j, member 11 KCNK1 - potassium channel, subfamily k, member 1 KCNJ4 - potassium inwardly-rectifying channel, subfamily j, member 4 KCNJ3 - potassium inwardly-rectifying channel, subfamily j, member 3 KLHL22 - kelch-like family member 22 GALNT11 - udp-n-acetyl-alpha-d-galactosamine:polypeptide n-acetylgalactosaminyltransferase 11 (galnac-t11) KCNJ9 - potassium inwardly-rectifying channel, subfamily j, member 9 KCNJ8 - potassium inwardly-rectifying channel, subfamily j, member 8 PTPN5 - protein tyrosine phosphatase, non-receptor type 5 (striatum-enriched) NREP - neuronal regeneration related protein LSM4 - lsm4 homolog, u6 small nuclear rna associated (s. cerevisiae) KCNJ6 - potassium inwardly-rectifying channel, subfamily j, member 6 CSNK1E - casein kinase 1, epsilon AGBL4 - atp/gtp binding protein-like 4 PKNOX2 - pbx/knotted 1 homeobox 2 KCNH1 - potassium voltage-gated channel, subfamily h (eag-related), member 1 BRMS1L - breast cancer metastasis-suppressor 1-like KCNB1 - potassium voltage-gated channel, shab-related subfamily, member 1 PLEKHO1 - pleckstrin homology domain containing, family o member 1 KCNC1 - potassium voltage-gated channel, shaw-related subfamily, member 1 NEGR1 - neuronal growth regulator 1 UBE2O - ubiquitin-conjugating enzyme e2o GTF3C3 - general transcription factor iiic, polypeptide 3, 102kda FBXL2 - f-box and leucine-rich repeat protein 2 KCNC2 - potassium voltage-gated channel, shaw-related subfamily, member 2 C9orf69 - chromosome 9 open reading frame 69 DHX8 - deah (asp-glu-ala-his) box polypeptide 8 PHLDB2 - pleckstrin homology-like domain, family b, member 2 HDAC10 - histone deacetylase 10 PVRL3 - poliovirus receptor-related 3 SYT17 - synaptotagmin xvii DDB2 - damage-specific dna binding protein 2, 48kda COQ3 - coenzyme q3 methyltransferase BCL7B - b-cell cll/lymphoma 7b ZNF304 - zinc finger protein 304 MAP3K9 - mitogen-activated protein kinase kinase kinase 9 DCTN1 - dynactin 1 MYOZ2 - myozenin 2 GPR61 - g protein-coupled receptor 61 CYTH3 - cytohesin 3 CNRIP1 - cannabinoid receptor interacting protein 1 BCL11A - b-cell cll/lymphoma 11a (zinc finger protein) MYRIP - myosin viia and rab interacting protein FRMPD2 - ferm and pdz domain containing 2 NUMBL - numb homolog (drosophila)-like CERS6 - ceramide synthase 6 CRLF1 - cytokine receptor-like factor 1 NCALD - neurocalcin delta MPP7 - membrane protein, palmitoylated 7 (maguk p55 subfamily member 7) MTHFD1L - methylenetetrahydrofolate dehydrogenase (nadp+ dependent) 1-like CCL4 - chemokine (c-c motif) ligand 4 NLK - nemo-like kinase TRIM58 - tripartite motif containing 58 DLGAP2 - discs, large (drosophila) homolog-associated protein 2 DLGAP1 - discs, large (drosophila) homolog-associated protein 1 MAGI1 - membrane associated guanylate kinase, ww and pdz domain containing 1 TBRG4 - transforming growth factor beta regulator 4 PNMA1 - paraneoplastic ma antigen 1 PLEKHG5 - pleckstrin homology domain containing, family g (with rhogef domain) member 5 WDR18 - wd repeat domain 18 FXYD6 - fxyd domain containing ion transport regulator 6 FGFRL1 - fibroblast growth factor receptor-like 1 ADAM11 - adam metallopeptidase domain 11 SCN1B - sodium channel, voltage-gated, type i, beta subunit SIPA1L1 - signal-induced proliferation-associated 1 like 1 CYP2E1 - cytochrome p450, family 2, subfamily e, polypeptide 1 CTU2 - cytosolic thiouridylase subunit 2 homolog (s. pombe) ZNF622 - zinc finger protein 622 IPCEF1 - interaction protein for cytohesin exchange factors 1 SETBP1 - set binding protein 1 CHD5 - chromodomain helicase dna binding protein 5 PRICKLE1 - prickle homolog 1 (drosophila) RAB24 - rab24, member ras oncogene family KIAA1239 - kiaa1239 MEF2C - myocyte enhancer factor 2c MKL2 - mkl/myocardin-like 2 OSBPL3 - oxysterol binding protein-like 3 COL24A1 - collagen, type xxiv, alpha 1 RPAP1 - rna polymerase ii associated protein 1 NSMF - nmda receptor synaptonuclear signaling and neuronal migration factor SCN8A - sodium channel, voltage gated, type viii, alpha subunit DAPK3 - death-associated protein kinase 3 C2CD3 - c2 calcium-dependent domain containing 3 TM6SF1 - transmembrane 6 superfamily member 1 HINFP - histone h4 transcription factor MYOM2 - myomesin 2 MET - met proto-oncogene SHC3 - shc (src homology 2 domain containing) transforming protein 3 LRSAM1 - leucine rich repeat and sterile alpha motif containing 1 MAP3K13 - mitogen-activated protein kinase kinase kinase 13 AP4M1 - adaptor-related protein complex 4, mu 1 subunit MFGE8 - milk fat globule-egf factor 8 protein MAPK12 - mitogen-activated protein kinase 12 SARS - seryl-trna synthetase DAB1 - dab, reelin signal transducer, homolog 1 (drosophila) SYNGR3 - synaptogyrin 3 SATB1 - satb homeobox 1 SYNGR1 - synaptogyrin 1 MBD3 - methyl-cpg binding domain protein 3 KITLG - kit ligand KCTD16 - potassium channel tetramerization domain containing 16 LYRM7 - lyr motif containing 7 RILP - rab interacting lysosomal protein TRPC4AP - transient receptor potential cation channel, subfamily c, member 4 associated protein TUBGCP6 - tubulin, gamma complex associated protein 6 ANAPC11 - anaphase promoting complex subunit 11 CXXC5 - cxxc finger protein 5 P2RX6 - purinergic receptor p2x, ligand-gated ion channel, 6 SLC8A2 - solute carrier family 8 (sodium/calcium exchanger), member 2 PDLIM1 - pdz and lim domain 1 RAB6B - rab6b, member ras oncogene family DPP4 - dipeptidyl-peptidase 4 MAPT - microtubule-associated protein tau LRFN5 - leucine rich repeat and fibronectin type iii domain containing 5 STAMBPL1 - stam binding protein-like 1 ATP6V0D1 - atpase, h+ transporting, lysosomal 38kda, v0 subunit d1 SIRT6 - sirtuin 6 MAP1A - microtubule-associated protein 1a DRD1 - dopamine receptor d1 STRIP1 - striatin interacting protein 1 SIRT7 - sirtuin 7 DPT - dermatopontin UNC119 - unc-119 homolog (c. elegans) DCDC2 - doublecortin domain containing 2 RHCG - rh family, c glycoprotein MKL1 - megakaryoblastic leukemia (translocation) 1 HCN1 - hyperpolarization activated cyclic nucleotide-gated potassium channel 1 DVL1 - dishevelled segment polarity protein 1 SYT13 - synaptotagmin xiii DVL3 - dishevelled segment polarity protein 3 MATK - megakaryocyte-associated tyrosine kinase CCM2 - cerebral cavernous malformation 2 LDB2 - lim domain binding 2 RAB11FIP5 - rab11 family interacting protein 5 (class i) RASSF5 - ras association (ralgds/af-6) domain family member 5 DUSP2 - dual specificity phosphatase 2 PITPNM2 - phosphatidylinositol transfer protein, membrane-associated 2 DECR2 - 2,4-dienoyl coa reductase 2, peroxisomal DTX1 - deltex homolog 1 (drosophila) PRC1 - protein regulator of cytokinesis 1 TSHZ3 - teashirt zinc finger homeobox 3 MRPS23 - mitochondrial ribosomal protein s23 CCDC64B - coiled-coil domain containing 64b L3MBTL2 - l(3)mbt-like 2 (drosophila) VPS18 - vacuolar protein sorting 18 homolog (s. cerevisiae) SHROOM3 - shroom family member 3 DLX1 - distal-less homeobox 1 DLX2 - distal-less homeobox 2 CABYR - calcium binding tyrosine-(y)-phosphorylation regulated TSPAN17 - tetraspanin 17 DPP10 - dipeptidyl-peptidase 10 (non-functional) TEKT5 - tektin 5 LCE3C - late cornified envelope 3c PKD2L1 - polycystic kidney disease 2-like 1 USP28 - ubiquitin specific peptidase 28 ASB2 - ankyrin repeat and socs box containing 2 DHPS - deoxyhypusine synthase TIAM2 - t-cell lymphoma invasion and metastasis 2 MYH7B - myosin, heavy chain 7b, cardiac muscle, beta SESN2 - sestrin 2 RASD1 - ras, dexamethasone-induced 1 CDK5RAP1 - cdk5 regulatory subunit associated protein 1 BRSK2 - br serine/threonine kinase 2 DIO2 - deiodinase, iodothyronine, type ii GRAMD1A - gram domain containing 1a LTK - leukocyte receptor tyrosine kinase PIAS4 - protein inhibitor of activated stat, 4 FBXW2 - f-box and wd repeat domain containing 2 RTN4RL1 - reticulon 4 receptor-like 1 KALRN - kalirin, rhogef kinase RSPH3 - radial spoke 3 homolog (chlamydomonas) UNC13C - unc-13 homolog c (c. elegans) RPS6KA4 - ribosomal protein s6 kinase, 90kda, polypeptide 4 STH - saitohin SYT16 - synaptotagmin xvi SGCG - sarcoglycan, gamma (35kda dystrophin-associated glycoprotein) ESYT3 - extended synaptotagmin-like protein 3 DNM1 - dynamin 1 UBE2D4 - ubiquitin-conjugating enzyme e2d 4 (putative) SGTA - small glutamine-rich tetratricopeptide repeat (tpr)-containing, alpha NAV2 - neuron navigator 2 H1FX - h1 histone family, member x SENP3 - sumo1/sentrin/smt3 specific peptidase 3 SH3GL1 - sh3-domain grb2-like 1 EMD - emerin EXOSC5 - exosome component 5 TAGLN3 - transgelin 3 EMX1 - empty spiracles homeobox 1 CCDC64 - coiled-coil domain containing 64 TSPAN14 - tetraspanin 14 KIFAP3 - kinesin-associated protein 3 SH3GLB2 - sh3-domain grb2-like endophilin b2 DHX30 - deah (asp-glu-ala-his) box helicase 30 IFT46 - intraflagellar transport 46 homolog (chlamydomonas) RALY - raly heterogeneous nuclear ribonucleoprotein ELL2 - elongation factor, rna polymerase ii, 2 ARRDC1 - arrestin domain containing 1 DKK1 - dickkopf wnt signaling pathway inhibitor 1 FARP1 - ferm, rhogef (arhgef) and pleckstrin domain protein 1 (chondrocyte-derived) C16orf80 - chromosome 16 open reading frame 80 TRIM7 - tripartite motif containing 7 C8orf4 - chromosome 8 open reading frame 4 NCAPH2 - non-smc condensin ii complex, subunit h2 MTDH - metadherin TUBA4A - tubulin, alpha 4a TPX2 - tpx2, microtubule-associated CDC42SE1 - cdc42 small effector 1 RBM5 - rna binding motif protein 5 VWC2L - von willebrand factor c domain containing protein 2-like CCT5 - chaperonin containing tcp1, subunit 5 (epsilon) TFPT - tcf3 (e2a) fusion partner (in childhood leukemia) NAB2 - ngfi-a binding protein 2 (egr1 binding protein 2) SPHK2 - sphingosine kinase 2 ELAVL2 - elav (embryonic lethal, abnormal vision, drosophila)-like 2 (hu antigen b) PDRG1 - p53 and dna-damage regulated 1 TSTA3 - tissue specific transplantation antigen p35b NME6 - nme/nm23 nucleoside diphosphate kinase 6 ABT1 - activator of basal transcription 1 MAST1 - microtubule associated serine/threonine kinase 1 MYO5B - myosin vb PARVB - parvin, beta HOOK2 - hook microtubule-tethering protein 2 ST8SIA5 - st8 alpha-n-acetyl-neuraminide alpha-2,8-sialyltransferase 5 TPM3 - tropomyosin 3 HILPDA - hypoxia inducible lipid droplet-associated IGSF8 - immunoglobulin superfamily, member 8 TSSC4 - tumor suppressing subtransferable candidate 4 EEF1A2 - eukaryotic translation elongation factor 1 alpha 2 SNUPN - snurportin 1 MYBPC1 - myosin binding protein c, slow type TOP3A - topoisomerase (dna) iii alpha PITPNM3 - pitpnm family member 3 ANAPC2 - anaphase promoting complex subunit 2 MEGF8 - multiple egf-like-domains 8 PRDM8 - pr domain containing 8 MAST3 - microtubule associated serine/threonine kinase 3 ATXN7L3 - ataxin 7-like 3 MYO16 - myosin xvi EFNB2 - ephrin-b2 UNC13A - unc-13 homolog a (c. elegans) EFNA5 - ephrin-a5 SYCE1 - synaptonemal complex central element protein 1 EFNA3 - ephrin-a3 SNRNP200 - small nuclear ribonucleoprotein 200kda (u5) MYBPC2 - myosin binding protein c, fast type MEPE - matrix extracellular phosphoglycoprotein SHD - src homology 2 domain containing transforming protein d HIST3H2A - histone cluster 3, h2a HSFX1 - heat shock transcription factor family, x linked 1 E4F1 - e4f transcription factor 1 TSPAN1 - tetraspanin 1 NRBP1 - nuclear receptor binding protein 1 KIF21B - kinesin family member 21b E2F4 - e2f transcription factor 4, p107/p130-binding SMYD2 - set and mynd domain containing 2 MVD - mevalonate (diphospho) decarboxylase TRIM37 - tripartite motif containing 37 EDNRA - endothelin receptor type a GMPPA - gdp-mannose pyrophosphorylase a SPRY4 - sprouty homolog 4 (drosophila) CPLX3 - complexin 3 GMPPB - gdp-mannose pyrophosphorylase b TIMM22 - translocase of inner mitochondrial membrane 22 homolog (yeast) TRAF3 - tnf receptor-associated factor 3 ARL4A - adp-ribosylation factor-like 4a ARL4C - adp-ribosylation factor-like 4c ECM1 - extracellular matrix protein 1 PAK6 - p21 protein (cdc42/rac)-activated kinase 6 TRAP1 - tnf receptor-associated protein 1 UQCRC1 - ubiquinol-cytochrome c reductase core protein i MTR - 5-methyltetrahydrofolate-homocysteine methyltransferase UROD - uroporphyrinogen decarboxylase CAMK1D - calcium/calmodulin-dependent protein kinase id FABP3 - fatty acid binding protein 3, muscle and heart (mammary-derived growth inhibitor) IQSEC2 - iq motif and sec7 domain 2 FSTL4 - follistatin-like 4 PTK2B - protein tyrosine kinase 2 beta RAP1GAP2 - rap1 gtpase activating protein 2 CUL9 - cullin 9 C12orf5 - chromosome 12 open reading frame 5 FARSA - phenylalanyl-trna synthetase, alpha subunit FASN - fatty acid synthase FAP - fibroblast activation protein, alpha NR1H2 - nuclear receptor subfamily 1, group h, member 2 CAMTA2 - calmodulin binding transcription activator 2 ETV4 - ets variant 4 TOM1L1 - target of myb1 (chicken)-like 1 SH2D3C - sh2 domain containing 3c PLXND1 - plexin d1 TOM1 - target of myb1 (chicken) SPSB3 - spla/ryanodine receptor domain and socs box containing 3 MED16 - mediator complex subunit 16 VIPR1 - vasoactive intestinal peptide receptor 1 CCDC24 - coiled-coil domain containing 24 VIP - vasoactive intestinal peptide TRIB2 - tribbles homolog 2 (drosophila) ADCK2 - aarf domain containing kinase 2 CHAF1A - chromatin assembly factor 1, subunit a (p150) KIFC2 - kinesin family member c2 SAE1 - sumo1 activating enzyme subunit 1 CREB3L1 - camp responsive element binding protein 3-like 1 VARS - valyl-trna synthetase CIC - capicua transcriptional repressor PDXP - pyridoxal (pyridoxine, vitamin b6) phosphatase JOSD1 - josephin domain containing 1 MCOLN1 - mucolipin 1 CITED1 - cbp/p300-interacting transactivator, with glu/asp-rich carboxy-terminal domain, 1 TYRP1 - tyrosinase-related protein 1 YTHDC1 - yth domain containing 1 MACROD1 - macro domain containing 1 COMMD5 - comm domain containing 5 NTMT1 - n-terminal xaa-pro-lys n-methyltransferase 1 PPP1R37 - protein phosphatase 1, regulatory subunit 37 GFPT2 - glutamine-fructose-6-phosphate transaminase 2 CAMK1G - calcium/calmodulin-dependent protein kinase ig HIRA - histone cell cycle regulator MOB2 - mob kinase activator 2 ACAD9 - acyl-coa dehydrogenase family, member 9 ETS2 - v-ets avian erythroblastosis virus e26 oncogene homolog 2 TYRO3 - tyro3 protein tyrosine kinase ETV1 - ets variant 1 TYK2 - tyrosine kinase 2 MAFB - v-maf avian musculoaponeurotic fibrosarcoma oncogene homolog b PLEKHM2 - pleckstrin homology domain containing, family m (with run domain) member 2 EPHA3 - eph receptor a3 UCN - urocortin COL23A1 - collagen, type xxiii, alpha 1 VSTM1 - v-set and transmembrane domain containing 1 TRIM54 - tripartite motif containing 54 XPO6 - exportin 6 EPHA4 - eph receptor a4 PLXDC1 - plexin domain containing 1 ADCK1 - aarf domain containing kinase 1 PAK7 - p21 protein (cdc42/rac)-activated kinase 7 ERCC2 - excision repair cross-complementing rodent repair deficiency, complementation group 2 MPG - n-methylpurine-dna glycosylase SYT12 - synaptotagmin xii VPS13A - vacuolar protein sorting 13 homolog a (s. cerevisiae) FLT3 - fms-related tyrosine kinase 3 SETDB1 - set domain, bifurcated 1 BRK1 - brick1, scar/wave actin-nucleating complex subunit VILL - villin-like TCF15 - transcription factor 15 (basic helix-loop-helix) PPP2R2D - protein phosphatase 2, regulatory subunit b, delta SOGA2 - soga family member 2 SEC24D - sec24 family, member d (s. cerevisiae) PARD6A - par-6 partitioning defective 6 homolog alpha (c. elegans) SLC22A18 - solute carrier family 22, member 18 STMN3 - stathmin-like 3 CDON - cell adhesion associated, oncogene regulated ZKSCAN7 - zinc finger with krab and scan domains 7 ADC - arginine decarboxylase PLCB1 - phospholipase c, beta 1 (phosphoinositide-specific) SHANK1 - sh3 and multiple ankyrin repeat domains 1 MB21D2 - mab-21 domain containing 2 PPP1R26 - protein phosphatase 1, regulatory subunit 26 RAB15 - rab15, member ras oncogene family SIN3B - sin3 transcription regulator family member b ZNF385B - zinc finger protein 385b TBC1D25 - tbc1 domain family, member 25 AHNAK2 - ahnak nucleoprotein 2 SV2B - synaptic vesicle glycoprotein 2b FOXJ2 - forkhead box j2 SATB2 - satb homeobox 2 OCA2 - oculocutaneous albinism ii METTL21A - methyltransferase like 21a SRGAP3 - slit-robo rho gtpase activating protein 3 SCN3B - sodium channel, voltage-gated, type iii, beta subunit CCNYL1 - cyclin y-like 1 VPS26B - vacuolar protein sorting 26 homolog b (s. pombe) ZDHHC22 - zinc finger, dhhc-type containing 22 UBAP2 - ubiquitin associated protein 2 DCAF11 - ddb1 and cul4 associated factor 11 KLHL18 - kelch-like family member 18 NUAK1 - nuak family, snf1-like kinase, 1 UBXN6 - ubx domain protein 6 DCAF6 - ddb1 and cul4 associated factor 6 TNFRSF11B - tumor necrosis factor receptor superfamily, member 11b CKAP5 - cytoskeleton associated protein 5 FGF14 - fibroblast growth factor 14 TAF6 - taf6 rna polymerase ii, tata box binding protein (tbp)-associated factor, 80kda TAF5 - taf5 rna polymerase ii, tata box binding protein (tbp)-associated factor, 100kda ESYT1 - extended synaptotagmin-like protein 1 FGF9 - fibroblast growth factor 9 NTHL1 - nth endonuclease iii-like 1 (e. coli) IST1 - increased sodium tolerance 1 homolog (yeast) BCLAF1 - bcl2-associated transcription factor 1 FES - feline sarcoma oncogene NTSR1 - neurotensin receptor 1 (high affinity) NOMO2 - nodal modulator 2 MAL2 - mal, t-cell differentiation protein 2 (gene/pseudogene) RIMS3 - regulating synaptic membrane exocytosis 3 SNX17 - sorting nexin 17 DHX38 - deah (asp-glu-ala-his) box polypeptide 38 MEMO1 - mediator of cell motility 1 NOSIP - nitric oxide synthase interacting protein NPW - neuropeptide w TBCD - tubulin folding cofactor d PYDC2 - pyrin domain containing 2 NPPA - natriuretic peptide a TNNI3K - tnni3 interacting kinase CNTN4 - contactin 4 RNF40 - ring finger protein 40, e3 ubiquitin protein ligase GAL - galanin/gmap prepropeptide TCF4 - transcription factor 4 OBSL1 - obscurin-like 1 FAM212B - family with sequence similarity 212, member b NRGN - neurogranin (protein kinase c substrate, rc3) CUL7 - cullin 7 RNF208 - ring finger protein 208 FHOD3 - formin homology 2 domain containing 3 KCNH3 - potassium voltage-gated channel, subfamily h (eag-related), member 3 CAMK2N2 - calcium/calmodulin-dependent protein kinase ii inhibitor 2 NOS2 - nitric oxide synthase 2, inducible CDIP1 - cell death-inducing p53 target 1 GRIP1 - glutamate receptor interacting protein 1 MUS81 - mus81 structure-specific endonuclease subunit NME1 - nme/nm23 nucleoside diphosphate kinase 1 ICAM5 - intercellular adhesion molecule 5, telencephalin FUZ - fuzzy planar cell polarity protein NOS1AP - nitric oxide synthase 1 (neuronal) adaptor protein SEMA3E - sema domain, immunoglobulin domain (ig), short basic domain, secreted, (semaphorin) 3e TMEM203 - transmembrane protein 203 TLE3 - transducin-like enhancer of split 3 (e(sp1) homolog, drosophila) NOV - nephroblastoma overexpressed MYO19 - myosin xix NPAS1 - neuronal pas domain protein 1 PILRB - paired immunoglobin-like type 2 receptor beta PACSIN1 - protein kinase c and casein kinase substrate in neurons 1 PPP6R2 - protein phosphatase 6, regulatory subunit 2 SIRT4 - sirtuin 4 CNOT3 - ccr4-not transcription complex, subunit 3 LMOD3 - leiomodin 3 (fetal) NPY - neuropeptide y KCNIP3 - kv channel interacting protein 3, calsenilin MICA - mhc class i polypeptide-related sequence a ABCB9 - atp-binding cassette, sub-family b (mdr/tap), member 9 TMPO - thymopoietin ZSCAN12 - zinc finger and scan domain containing 12 NFIX - nuclear factor i/x (ccaat-binding transcription factor) FOXP2 - forkhead box p2 FRMPD4 - ferm and pdz domain containing 4 PTGES2 - prostaglandin e synthase 2 NFKBIE - nuclear factor of kappa light polypeptide gene enhancer in b-cells inhibitor, epsilon TNFAIP1 - tumor necrosis factor, alpha-induced protein 1 (endothelial) TNNC2 - troponin c type 2 (fast) STOML2 - stomatin (epb72)-like 2 EHD2 - eh-domain containing 2 NINJ1 - ninjurin 1 TNNT3 - troponin t type 3 (skeletal, fast) EHD3 - eh-domain containing 3 TNNT2 - troponin t type 2 (cardiac) CCL28 - chemokine (c-c motif) ligand 28 EIF4ENIF1 - eukaryotic translation initiation factor 4e nuclear import factor 1 WDR82 - wd repeat domain 82 KCNQ5 - potassium voltage-gated channel, kqt-like subfamily, member 5 COL5A3 - collagen, type v, alpha 3 NEUROD1 - neuronal differentiation 1 NEUROD2 - neuronal differentiation 2 PRRT2 - proline-rich transmembrane protein 2 ULBP2 - ul16 binding protein 2 LRRC8B - leucine rich repeat containing 8 family, member b RPRM - reprimo, tp53 dependent g2 arrest mediator candidate NELL2 - nel-like 2 (chicken) EGLN2 - egl-9 family hypoxia-inducible factor 2 CCDC68 - coiled-coil domain containing 68 NELL1 - nel-like 1 (chicken) RCOR2 - rest corepressor 2 ABTB1 - ankyrin repeat and btb (poz) domain containing 1 NEDD9 - neural precursor cell expressed, developmentally down-regulated 9 TERF2 - telomeric repeat binding factor 2 MICAL2 - microtubule associated monooxygenase, calponin and lim domain containing 2 NEFM - neurofilament, medium polypeptide TESK1 - testis-specific kinase 1 PPM1F - protein phosphatase, mg2+/mn2+ dependent, 1f CYHR1 - cysteine/histidine-rich 1 CBX7 - chromobox homolog 7 PDGFD - platelet derived growth factor d TFR2 - transferrin receptor 2 FLAD1 - flavin adenine dinucleotide synthetase 1 SOHLH1 - spermatogenesis and oogenesis specific basic helix-loop-helix 1 RGS6 - regulator of g-protein signaling 6 THEMIS - thymocyte selection associated ARHGEF4 - rho guanine nucleotide exchange factor (gef) 4 CTNNBL1 - catenin, beta like 1 MORN3 - morn repeat containing 3 LRRC14 - leucine rich repeat containing 14 FOSL2 - fos-like antigen 2 TTI1 - telo2 interacting protein 1 TARS2 - threonyl-trna synthetase 2, mitochondrial (putative) NAA50 - n(alpha)-acetyltransferase 50, nate catalytic subunit THPO - thrombopoietin |
| GO:0015467 | G-protein activated inward rectifier potassium channel activity | 7.8E-5 | 6.38E-3 | 39.38 (16750,4,319,3) | KCNJ3 - potassium inwardly-rectifying channel, subfamily j, member 3 KCNJ9 - potassium inwardly-rectifying channel, subfamily j, member 9 KCNJ6 - potassium inwardly-rectifying channel, subfamily j, member 6 |
| GO:0005544 | calcium-dependent phospholipid binding | 1.03E-4 | 8.29E-3 | 3.92 (16750,40,1388,13) | CPNE4 - copine iv PCLO - piccolo presynaptic cytomatrix protein ESYT1 - extended synaptotagmin-like protein 1 SYT1 - synaptotagmin i RPH3A - rabphilin 3a homolog (mouse) SYT17 - synaptotagmin xvii SYT13 - synaptotagmin xiii CPNE9 - copine family member ix DOC2A - double c2-like domains, alpha ESYT3 - extended synaptotagmin-like protein 3 PLA2G4A - phospholipase a2, group iva (cytosolic, calcium-dependent) SYT12 - synaptotagmin xii CPNE8 - copine viii |
| GO:0140096 | catalytic activity, acting on a protein | 1.04E-4 | 8.23E-3 | 1.28 (16750,1971,1717,259) | MKRN1 - makorin ring finger protein 1 LIMK1 - lim domain kinase 1 BCKDK - branched chain ketoacid dehydrogenase kinase PDIA5 - protein disulfide isomerase family a, member 5 SPG7 - spastic paraplegia 7 (pure and complicated autosomal recessive) GCNT4 - glucosaminyl (n-acetyl) transferase 4, core 2 RET - ret proto-oncogene CCNO - cyclin o CDKL1 - cyclin-dependent kinase-like 1 (cdc2-related kinase) BRSK1 - br serine/threonine kinase 1 DUSP27 - dual specificity phosphatase 27 (putative) NEURL4 - neuralized homolog 4 (drosophila) FBXL20 - f-box and leucine-rich repeat protein 20 UBE2J2 - ubiquitin-conjugating enzyme e2, j2 OTUB1 - otu domain, ubiquitin aldehyde binding 1 STK19 - serine/threonine kinase 19 RNF215 - ring finger protein 215 MPND - mpn domain containing MAST1 - microtubule associated serine/threonine kinase 1 GGT7 - gamma-glutamyltransferase 7 PPID - peptidylprolyl isomerase d CTDSPL - ctd (carboxy-terminal domain, rna polymerase ii, polypeptide a) small phosphatase-like PPEF1 - protein phosphatase, ef-hand calcium binding domain 1 CTSA - cathepsin a PPIA - peptidylprolyl isomerase a (cyclophilin a) USP19 - ubiquitin specific peptidase 19 PARL - presenilin associated, rhomboid-like PPM1B - protein phosphatase, mg2+/mn2+ dependent, 1b ATG4D - autophagy related 4d, cysteine peptidase CCNA1 - cyclin a1 PIDD - p53-induced death domain protein MAST3 - microtubule associated serine/threonine kinase 3 GOLGA7B - golgin a7 family, member b PIM3 - pim-3 oncogene PDZRN3 - pdz domain containing ring finger 3 BAP1 - brca1 associated protein-1 (ubiquitin carboxy-terminal hydrolase) HEMK1 - hemk methyltransferase family member 1 EFNA3 - ephrin-a3 PDZRN4 - pdz domain containing ring finger 4 FEM1A - fem-1 homolog a (c. elegans) FIGNL2 - fidgetin-like 2 PNCK - pregnancy up-regulated non-ubiquitously expressed cam kinase MED20 - mediator complex subunit 20 SMYD2 - set and mynd domain containing 2 TRHDE - thyrotropin-releasing hormone degrading enzyme TRIM37 - tripartite motif containing 37 TRAF3 - tnf receptor-associated factor 3 NEURL3 - neuralized homolog 3 (drosophila) pseudogene HECW1 - hect, c2 and ww domain containing e3 ubiquitin protein ligase 1 DUSP26 - dual specificity phosphatase 26 (putative) PAK6 - p21 protein (cdc42/rac)-activated kinase 6 MBTPS2 - membrane-bound transcription factor peptidase, site 2 PRSS16 - protease, serine, 16 (thymus) PRKAA2 - protein kinase, amp-activated, alpha 2 catalytic subunit PRKAB1 - protein kinase, amp-activated, beta 1 non-catalytic subunit PPIE - peptidylprolyl isomerase e (cyclophilin e) CAPNS1 - calpain, small subunit 1 CAMK1D - calcium/calmodulin-dependent protein kinase id PREP - prolyl endopeptidase CAMK2G - calcium/calmodulin-dependent protein kinase ii gamma ADRBK2 - adrenergic, beta, receptor kinase 2 ADRBK1 - adrenergic, beta, receptor kinase 1 ADAMTS8 - adam metallopeptidase with thrombospondin type 1 motif, 8 CAMK2A - calcium/calmodulin-dependent protein kinase ii alpha PTPRT - protein tyrosine phosphatase, receptor type, t PTK2B - protein tyrosine kinase 2 beta PPP3R1 - protein phosphatase 3, regulatory subunit b, alpha CIT - citron (rho-interacting, serine/threonine kinase 21) PPP5C - protein phosphatase 5, catalytic subunit FAP - fibroblast activation protein, alpha CTSK - cathepsin k CAD - carbamoyl-phosphate synthetase 2, aspartate transcarbamylase, and dihydroorotase PINK1 - pten induced putative kinase 1 TRIM17 - tripartite motif containing 17 CTSD - cathepsin d LNX1 - ligand of numb-protein x 1, e3 ubiquitin protein ligase SPSB3 - spla/ryanodine receptor domain and socs box containing 3 USP7 - ubiquitin specific peptidase 7 (herpes virus-associated) UBE2E3 - ubiquitin-conjugating enzyme e2e 3 HERC6 - hect and rld domain containing e3 ubiquitin protein ligase family member 6 ADCK2 - aarf domain containing kinase 2 CAMKK1 - calcium/calmodulin-dependent protein kinase kinase 1, alpha STK25 - serine/threonine kinase 25 PPM1G - protein phosphatase, mg2+/mn2+ dependent, 1g MED10 - mediator complex subunit 10 STK11 - serine/threonine kinase 11 PDXP - pyridoxal (pyridoxine, vitamin b6) phosphatase JOSD1 - josephin domain containing 1 LONP1 - lon peptidase 1, mitochondrial ADCK5 - aarf domain containing kinase 5 RNF165 - ring finger protein 165 ST14 - suppression of tumorigenicity 14 (colon carcinoma) GAK - cyclin g associated kinase CTSF - cathepsin f MGAT4C - mannosyl (alpha-1,3-)-glycoprotein beta-1,4-n-acetylglucosaminyltransferase, isozyme c (putative) ADAM32 - adam metallopeptidase domain 32 ADAM19 - adam metallopeptidase domain 19 CCNE1 - cyclin e1 NTMT1 - n-terminal xaa-pro-lys n-methyltransferase 1 RHBDL3 - rhomboid, veinlet-like 3 (drosophila) GPAA1 - glycosylphosphatidylinositol anchor attachment 1 CCNG2 - cyclin g2 PRKY - protein kinase, y-linked, pseudogene AGBL5 - atp/gtp binding protein-like 5 CAMK1G - calcium/calmodulin-dependent protein kinase ig ZDHHC16 - zinc finger, dhhc-type containing 16 ASPHD2 - aspartate beta-hydroxylase domain containing 2 FBXO6 - f-box protein 6 TYRO3 - tyro3 protein tyrosine kinase ADAM23 - adam metallopeptidase domain 23 ITPKA - inositol-trisphosphate 3-kinase a TYK2 - tyrosine kinase 2 MAP2K1 - mitogen-activated protein kinase kinase 1 MAP2K2 - mitogen-activated protein kinase kinase 2 MAPK10 - mitogen-activated protein kinase 10 PMPCA - peptidase (mitochondrial processing) alpha MAPK11 - mitogen-activated protein kinase 11 EPHA3 - eph receptor a3 METAP2 - methionyl aminopeptidase 2 PKDCC - protein kinase domain containing, cytoplasmic MAPK3 - mitogen-activated protein kinase 3 CCNI - cyclin i PRKG2 - protein kinase, cgmp-dependent, type ii GALNT11 - udp-n-acetyl-alpha-d-galactosamine:polypeptide n-acetylgalactosaminyltransferase 11 (galnac-t11) EPHB3 - eph receptor b3 EPHA5 - eph receptor a5 EPHA4 - eph receptor a4 PTPN5 - protein tyrosine phosphatase, non-receptor type 5 (striatum-enriched) CSNK1E - casein kinase 1, epsilon DTX4 - deltex homolog 4 (drosophila) AGBL4 - atp/gtp binding protein-like 4 PRKCE - protein kinase c, epsilon EPHB6 - eph receptor b6 RNF123 - ring finger protein 123 CCND2 - cyclin d2 PRKCB - protein kinase c, beta BRMS1L - breast cancer metastasis-suppressor 1-like RNPEPL1 - arginyl aminopeptidase (aminopeptidase b)-like 1 ADCK1 - aarf domain containing kinase 1 UBE2O - ubiquitin-conjugating enzyme e2o ERCC2 - excision repair cross-complementing rodent repair deficiency, complementation group 2 PAK7 - p21 protein (cdc42/rac)-activated kinase 7 FBXL2 - f-box and leucine-rich repeat protein 2 PRSS3 - protease, serine, 3 RMND5A - required for meiotic nuclear division 5 homolog a (s. cerevisiae) LGMN - legumain SSH1 - slingshot protein phosphatase 1 PRSS1 - protease, serine, 1 (trypsin 1) HDAC10 - histone deacetylase 10 FLT3 - fms-related tyrosine kinase 3 SETDB1 - set domain, bifurcated 1 PPP2R2D - protein phosphatase 2, regulatory subunit b, delta KLK7 - kallikrein-related peptidase 7 DDB2 - damage-specific dna binding protein 2, 48kda RMND5B - required for meiotic nuclear division 5 homolog b (s. cerevisiae) MAP3K9 - mitogen-activated protein kinase kinase kinase 9 UBE3B - ubiquitin protein ligase e3b PSEN2 - presenilin 2 (alzheimer disease 4) PRPF19 - pre-mrna processing factor 19 TRIM25 - tripartite motif containing 25 PRSS12 - protease, serine, 12 (neurotrypsin, motopsin) SIN3B - sin3 transcription regulator family member b METTL21A - methyltransferase like 21a USP43 - ubiquitin specific peptidase 43 ZDHHC22 - zinc finger, dhhc-type containing 22 RNF148 - ring finger protein 148 LRRK1 - leucine-rich repeat kinase 1 HR - hair growth associated NLK - nemo-like kinase TRIM58 - tripartite motif containing 58 FBXO40 - f-box protein 40 UBE2QL1 - ubiquitin-conjugating enzyme e2q family-like 1 MMP16 - matrix metallopeptidase 16 (membrane-inserted) MMP17 - matrix metallopeptidase 17 (membrane-inserted) TBRG4 - transforming growth factor beta regulator 4 NUAK1 - nuak family, snf1-like kinase, 1 PAK1 - p21 protein (cdc42/rac)-activated kinase 1 PSMD2 - proteasome (prosome, macropain) 26s subunit, non-atpase, 2 RNF31 - ring finger protein 31 FGFRL1 - fibroblast growth factor receptor-like 1 LEPREL2 - leprecan-like 2 ADAM11 - adam metallopeptidase domain 11 PDPK1 - 3-phosphoinositide dependent protein kinase-1 FES - feline sarcoma oncogene ATP5SL - atp5s-like DCLK1 - doublecortin-like kinase 1 CDK5 - cyclin-dependent kinase 5 DAPK3 - death-associated protein kinase 3 NOSIP - nitric oxide synthase interacting protein PRDM2 - pr domain containing 2, with znf domain ZDHHC23 - zinc finger, dhhc-type containing 23 CDK9 - cyclin-dependent kinase 9 MTMR14 - myotubularin related protein 14 MET - met proto-oncogene PCSK2 - proprotein convertase subtilisin/kexin type 2 MAP3K13 - mitogen-activated protein kinase kinase kinase 13 LRSAM1 - leucine rich repeat and sterile alpha motif containing 1 CDKN3 - cyclin-dependent kinase inhibitor 3 PTPN3 - protein tyrosine phosphatase, non-receptor type 3 PCSK1 - proprotein convertase subtilisin/kexin type 1 PTPN9 - protein tyrosine phosphatase, non-receptor type 9 MAPK12 - mitogen-activated protein kinase 12 TNNI3K - tnni3 interacting kinase ZFP91 - zfp91 zinc finger protein RNF40 - ring finger protein 40, e3 ubiquitin protein ligase CAPN14 - calpain 14 NEURL - neuralized homolog (drosophila) PTPRF - protein tyrosine phosphatase, receptor type, f RNF208 - ring finger protein 208 ANAPC11 - anaphase promoting complex subunit 11 P4HTM - prolyl 4-hydroxylase, transmembrane (endoplasmic reticulum) PTPRR - protein tyrosine phosphatase, receptor type, r CDK14 - cyclin-dependent kinase 14 B4GALNT4 - beta-1,4-n-acetyl-galactosaminyl transferase 4 STK32C - serine/threonine kinase 32c DPP4 - dipeptidyl-peptidase 4 STAMBPL1 - stam binding protein-like 1 SIRT6 - sirtuin 6 SIRT7 - sirtuin 7 MAP4K2 - mitogen-activated protein kinase kinase kinase kinase 2 DUSP6 - dual specificity phosphatase 6 PEPD - peptidase d MATK - megakaryocyte-associated tyrosine kinase STYK1 - serine/threonine/tyrosine kinase 1 TNFAIP1 - tumor necrosis factor, alpha-induced protein 1 (endothelial) METTL21C - methyltransferase like 21c XYLT1 - xylosyltransferase i DUSP2 - dual specificity phosphatase 2 MAP3K6 - mitogen-activated protein kinase kinase kinase 6 GRK6 - g protein-coupled receptor kinase 6 WDR82 - wd repeat domain 82 FBXL15 - f-box and leucine-rich repeat protein 15 EGLN2 - egl-9 family hypoxia-inducible factor 2 PIN1 - peptidylprolyl cis/trans isomerase, nima-interacting 1 TSPAN17 - tetraspanin 17 DPP10 - dipeptidyl-peptidase 10 (non-functional) PPM1F - protein phosphatase, mg2+/mn2+ dependent, 1f TESK1 - testis-specific kinase 1 ASB2 - ankyrin repeat and socs box containing 2 USP28 - ubiquitin specific peptidase 28 GALNT9 - udp-n-acetyl-alpha-d-galactosamine:polypeptide n-acetylgalactosaminyltransferase 9 (galnac-t9) LHPP - phospholysine phosphohistidine inorganic pyrophosphate phosphatase BRSK2 - br serine/threonine kinase 2 LTK - leukocyte receptor tyrosine kinase CLK2 - cdc-like kinase 2 RHBDL1 - rhomboid, veinlet-like 1 (drosophila) PIAS4 - protein inhibitor of activated stat, 4 FBXW2 - f-box and wd repeat domain containing 2 KALRN - kalirin, rhogef kinase PHKG2 - phosphorylase kinase, gamma 2 (testis) RPS6KA4 - ribosomal protein s6 kinase, 90kda, polypeptide 4 PIM1 - pim-1 oncogene UBE2D4 - ubiquitin-conjugating enzyme e2d 4 (putative) TPST2 - tyrosylprotein sulfotransferase 2 GSK3A - glycogen synthase kinase 3 alpha PGAM5 - phosphoglycerate mutase family member 5 P4HA2 - prolyl 4-hydroxylase, alpha polypeptide ii B4GALT7 - xylosylprotein beta 1,4-galactosyltransferase, polypeptide 7 SENP3 - sumo1/sentrin/smt3 specific peptidase 3 |
| GO:0015633 | zinc-transporting ATPase activity | 1.19E-4 | 9.27E-3 | 8,375.00 (16750,1,2,1) | SLC30A3 - solute carrier family 30 (zinc transporter), member 3 |
| GO:0008022 | protein C-terminus binding | 1.35E-4 | 1.03E-2 | 2.01 (16750,169,1774,36) | CITED1 - cbp/p300-interacting transactivator, with glu/asp-rich carboxy-terminal domain, 1 GRIP1 - glutamate receptor interacting protein 1 CENPF - centromere protein f, 350/400kda HPCAL4 - hippocalcin like 4 IFT46 - intraflagellar transport 46 homolog (chlamydomonas) SYT1 - synaptotagmin i TERF2 - telomeric repeat binding factor 2 TESK1 - testis-specific kinase 1 SDCBP2 - syndecan binding protein (syntenin) 2 PICK1 - protein interacting with prkca 1 NEIL1 - nei endonuclease viii-like 1 (e. coli) PTK2B - protein tyrosine kinase 2 beta CNRIP1 - cannabinoid receptor interacting protein 1 SNX17 - sorting nexin 17 SHANK1 - sh3 and multiple ankyrin repeat domains 1 BAIAP2 - bai1-associated protein 2 KCNK3 - potassium channel, subfamily k, member 3 MAP2K1 - mitogen-activated protein kinase kinase 1 ZBTB16 - zinc finger and btb domain containing 16 DAPK3 - death-associated protein kinase 3 KCNJ11 - potassium inwardly-rectifying channel, subfamily j, member 11 CORO1A - coronin, actin binding protein, 1a PIAS4 - protein inhibitor of activated stat, 4 RAB3A - rab3a, member ras oncogene family USP7 - ubiquitin specific peptidase 7 (herpes virus-associated) XRCC6 - x-ray repair complementing defective repair in chinese hamster cells 6 SNTG1 - syntrophin, gamma 1 SAE1 - sumo1 activating enzyme subunit 1 TNNI3K - tnni3 interacting kinase DNM1 - dynamin 1 PCGF1 - polycomb group ring finger 1 MAGI1 - membrane associated guanylate kinase, ww and pdz domain containing 1 TCF4 - transcription factor 4 ECM1 - extracellular matrix protein 1 ERCC2 - excision repair cross-complementing rodent repair deficiency, complementation group 2 SH3GL1 - sh3-domain grb2-like 1 |
| GO:0004674 | protein serine/threonine kinase activity | 1.41E-4 | 1.06E-2 | 1.67 (16750,400,1651,66) | PRKAA2 - protein kinase, amp-activated, alpha 2 catalytic subunit PRKAB1 - protein kinase, amp-activated, beta 1 non-catalytic subunit CAMK1D - calcium/calmodulin-dependent protein kinase id LIMK1 - lim domain kinase 1 CDK14 - cyclin-dependent kinase 14 BCKDK - branched chain ketoacid dehydrogenase kinase ADRBK2 - adrenergic, beta, receptor kinase 2 CAMK2G - calcium/calmodulin-dependent protein kinase ii gamma ADRBK1 - adrenergic, beta, receptor kinase 1 STK32C - serine/threonine kinase 32c CAMK2A - calcium/calmodulin-dependent protein kinase ii alpha MAP3K9 - mitogen-activated protein kinase kinase kinase 9 PTK2B - protein tyrosine kinase 2 beta CIT - citron (rho-interacting, serine/threonine kinase 21) CDKL1 - cyclin-dependent kinase-like 1 (cdc2-related kinase) PINK1 - pten induced putative kinase 1 BRSK1 - br serine/threonine kinase 1 LRRK1 - leucine-rich repeat kinase 1 ADCK2 - aarf domain containing kinase 2 NLK - nemo-like kinase CAMKK1 - calcium/calmodulin-dependent protein kinase kinase 1, alpha STK19 - serine/threonine kinase 19 NUAK1 - nuak family, snf1-like kinase, 1 STK25 - serine/threonine kinase 25 PAK1 - p21 protein (cdc42/rac)-activated kinase 1 STK11 - serine/threonine kinase 11 MAP3K6 - mitogen-activated protein kinase kinase kinase 6 MAST1 - microtubule associated serine/threonine kinase 1 ADCK5 - aarf domain containing kinase 5 GRK6 - g protein-coupled receptor kinase 6 GAK - cyclin g associated kinase TESK1 - testis-specific kinase 1 PRKY - protein kinase, y-linked, pseudogene PDPK1 - 3-phosphoinositide dependent protein kinase-1 CAMK1G - calcium/calmodulin-dependent protein kinase ig MAST3 - microtubule associated serine/threonine kinase 3 PIM3 - pim-3 oncogene BRSK2 - br serine/threonine kinase 2 ITPKA - inositol-trisphosphate 3-kinase a CLK2 - cdc-like kinase 2 MAP2K1 - mitogen-activated protein kinase kinase 1 DCLK1 - doublecortin-like kinase 1 MAP2K2 - mitogen-activated protein kinase kinase 2 MAPK10 - mitogen-activated protein kinase 10 CDK5 - cyclin-dependent kinase 5 DAPK3 - death-associated protein kinase 3 MAPK11 - mitogen-activated protein kinase 11 CDK9 - cyclin-dependent kinase 9 MAPK3 - mitogen-activated protein kinase 3 PNCK - pregnancy up-regulated non-ubiquitously expressed cam kinase PRKG2 - protein kinase, cgmp-dependent, type ii MAP3K13 - mitogen-activated protein kinase kinase kinase 13 KALRN - kalirin, rhogef kinase PHKG2 - phosphorylase kinase, gamma 2 (testis) CSNK1E - casein kinase 1, epsilon RPS6KA4 - ribosomal protein s6 kinase, 90kda, polypeptide 4 MAPK12 - mitogen-activated protein kinase 12 TNNI3K - tnni3 interacting kinase PIM1 - pim-1 oncogene PRKCE - protein kinase c, epsilon PRKCB - protein kinase c, beta ADCK1 - aarf domain containing kinase 1 PAK7 - p21 protein (cdc42/rac)-activated kinase 7 ERCC2 - excision repair cross-complementing rodent repair deficiency, complementation group 2 GSK3A - glycogen synthase kinase 3 alpha PAK6 - p21 protein (cdc42/rac)-activated kinase 6 |
| GO:0015277 | kainate selective glutamate receptor activity | 1.41E-4 | 1.04E-2 | 11.92 (16750,4,1405,4) | GRIK1 - glutamate receptor, ionotropic, kainate 1 GRIK2 - glutamate receptor, ionotropic, kainate 2 GRIK3 - glutamate receptor, ionotropic, kainate 3 GRIK4 - glutamate receptor, ionotropic, kainate 4 |
| GO:0005251 | delayed rectifier potassium channel activity | 1.7E-4 | 1.24E-2 | 5.47 (16750,30,919,9) | KCNA2 - potassium voltage-gated channel, shaker-related subfamily, member 2 KCNQ5 - potassium voltage-gated channel, kqt-like subfamily, member 5 KCNA1 - potassium voltage-gated channel, shaker-related subfamily, member 1 (episodic ataxia with myokymia) KCNS1 - potassium voltage-gated channel, delayed-rectifier, subfamily s, member 1 KCNH1 - potassium voltage-gated channel, subfamily h (eag-related), member 1 KCNB1 - potassium voltage-gated channel, shab-related subfamily, member 1 KCNC1 - potassium voltage-gated channel, shaw-related subfamily, member 1 KCNC2 - potassium voltage-gated channel, shaw-related subfamily, member 2 KCNC3 - potassium voltage-gated channel, shaw-related subfamily, member 3 |
| GO:0015081 | sodium ion transmembrane transporter activity | 1.98E-4 | 1.41E-2 | 2.15 (16750,132,1713,29) | SCN1A - sodium channel, voltage-gated, type i, alpha subunit SLC4A10 - solute carrier family 4, sodium bicarbonate transporter, member 10 SLC6A7 - solute carrier family 6 (neurotransmitter transporter), member 7 SCN2B - sodium channel, voltage-gated, type ii, beta subunit SCN1B - sodium channel, voltage-gated, type i, beta subunit ATP12A - atpase, h+/k+ transporting, nongastric, alpha polypeptide ATP1A1 - atpase, na+/k+ transporting, alpha 1 polypeptide SLC8A2 - solute carrier family 8 (sodium/calcium exchanger), member 2 ATP1A4 - atpase, na+/k+ transporting, alpha 4 polypeptide SLC6A17 - solute carrier family 6 (neutral amino acid transporter), member 17 PKD2L1 - polycystic kidney disease 2-like 1 SLC9A5 - solute carrier family 9, subfamily a (nhe5, cation proton antiporter 5), member 5 TRPM2 - transient receptor potential cation channel, subfamily m, member 2 SCN8A - sodium channel, voltage gated, type viii, alpha subunit SLC6A20 - solute carrier family 6 (proline imino transporter), member 20 ASIC2 - acid-sensing (proton-gated) ion channel 2 GRIK1 - glutamate receptor, ionotropic, kainate 1 GRIK2 - glutamate receptor, ionotropic, kainate 2 KCNK1 - potassium channel, subfamily k, member 1 GRIK3 - glutamate receptor, ionotropic, kainate 3 GRIK4 - glutamate receptor, ionotropic, kainate 4 SLC1A6 - solute carrier family 1 (high affinity aspartate/glutamate transporter), member 6 SCN3B - sodium channel, voltage-gated, type iii, beta subunit SLC9A6 - solute carrier family 9, subfamily a (nhe6, cation proton antiporter 6), member 6 HCN1 - hyperpolarization activated cyclic nucleotide-gated potassium channel 1 SLC4A8 - solute carrier family 4, sodium bicarbonate cotransporter, member 8 SLC4A7 - solute carrier family 4, sodium bicarbonate cotransporter, member 7 SLC17A7 - solute carrier family 17 (vesicular glutamate transporter), member 7 SHROOM2 - shroom family member 2 |
| GO:0016247 | channel regulator activity | 2.31E-4 | 1.62E-2 | 3.91 (16750,133,387,12) | KCNIP3 - kv channel interacting protein 3, calsenilin CACNG3 - calcium channel, voltage-dependent, gamma subunit 3 FXYD6 - fxyd domain containing ion transport regulator 6 CHRNA7 - cholinergic receptor, nicotinic, alpha 7 (neuronal) STX1A - syntaxin 1a (brain) PRKCB - protein kinase c, beta KCNS1 - potassium voltage-gated channel, delayed-rectifier, subfamily s, member 1 SCN1B - sodium channel, voltage-gated, type i, beta subunit KCNV1 - potassium channel, subfamily v, member 1 KCNAB3 - potassium voltage-gated channel, shaker-related subfamily, beta member 3 PTPN3 - protein tyrosine phosphatase, non-receptor type 3 ITPR1 - inositol 1,4,5-trisphosphate receptor, type 1 |
| GO:0042165 | neurotransmitter binding | 2.32E-4 | 1.61E-2 | 4.93 (16750,46,739,10) | CRHBP - corticotropin releasing hormone binding protein GLDC - glycine dehydrogenase (decarboxylating) CHRNA7 - cholinergic receptor, nicotinic, alpha 7 (neuronal) HTR5A - 5-hydroxytryptamine (serotonin) receptor 5a, g protein-coupled HTR1A - 5-hydroxytryptamine (serotonin) receptor 1a, g protein-coupled GLRA3 - glycine receptor, alpha 3 HTR2A - 5-hydroxytryptamine (serotonin) receptor 2a, g protein-coupled CHRM3 - cholinergic receptor, muscarinic 3 HTR1F - 5-hydroxytryptamine (serotonin) receptor 1f, g protein-coupled GRIN3A - glutamate receptor, ionotropic, n-methyl-d-aspartate 3a |
| GO:0003824 | catalytic activity | 2.4E-4 | 1.64E-2 | 1.15 (16750,5134,1720,605) | TRMT2B - trna methyltransferase 2 homolog b (s. cerevisiae) GFPT1 - glutamine--fructose-6-phosphate transaminase 1 MKRN1 - makorin ring finger protein 1 AGAP3 - arfgap with gtpase domain, ankyrin repeat and ph domain 3 LIMK1 - lim domain kinase 1 SEPT5 - septin 5 PDIA5 - protein disulfide isomerase family a, member 5 GDA - guanine deaminase ABCA4 - atp-binding cassette, sub-family a (abc1), member 4 SPG7 - spastic paraplegia 7 (pure and complicated autosomal recessive) FOXRED1 - fad-dependent oxidoreductase domain containing 1 GCNT4 - glucosaminyl (n-acetyl) transferase 4, core 2 DPF1 - d4, zinc and double phd fingers family 1 ACOT2 - acyl-coa thioesterase 2 PMM1 - phosphomannomutase 1 KAT2A - k(lysine) acetyltransferase 2a HRASLS5 - hras-like suppressor family, member 5 NCOA3 - nuclear receptor coactivator 3 GBA2 - glucosidase, beta (bile acid) 2 UAP1 - udp-n-acteylglucosamine pyrophosphorylase 1 POLN - polymerase (dna directed) nu CHSY3 - chondroitin sulfate synthase 3 RAB3D - rab3d, member ras oncogene family BRSK1 - br serine/threonine kinase 1 STEAP2 - steap family member 2, metalloreductase NEURL4 - neuralized homolog 4 (drosophila) PIP5KL1 - phosphatidylinositol-4-phosphate 5-kinase-like 1 UBE2J2 - ubiquitin-conjugating enzyme e2, j2 FBXL20 - f-box and leucine-rich repeat protein 20 RAB37 - rab37, member ras oncogene family OTUB1 - otu domain, ubiquitin aldehyde binding 1 MAGI3 - membrane associated guanylate kinase, ww and pdz domain containing 3 LIPC - lipase, hepatic PXDNL - peroxidasin homolog (drosophila)-like TUBA4B - tubulin, alpha 4b (pseudogene) RAB40C - rab40c, member ras oncogene family PLCG1 - phospholipase c, gamma 1 MPND - mpn domain containing SMUG1 - single-strand-selective monofunctional uracil-dna glycosylase 1 GGT7 - gamma-glutamyltransferase 7 FADS3 - fatty acid desaturase 3 PPID - peptidylprolyl isomerase d PPEF1 - protein phosphatase, ef-hand calcium binding domain 1 CTSA - cathepsin a PPIA - peptidylprolyl isomerase a (cyclophilin a) USP19 - ubiquitin specific peptidase 19 GLS - glutaminase PARL - presenilin associated, rhomboid-like PPM1B - protein phosphatase, mg2+/mn2+ dependent, 1b ATG4D - autophagy related 4d, cysteine peptidase GLDC - glycine dehydrogenase (decarboxylating) PIDD - p53-induced death domain protein ADCY6 - adenylate cyclase 6 PIM3 - pim-3 oncogene BAP1 - brca1 associated protein-1 (ubiquitin carboxy-terminal hydrolase) NT5C1A - 5'-nucleotidase, cytosolic ia HEMK1 - hemk methyltransferase family member 1 ST6GAL2 - st6 beta-galactosamide alpha-2,6-sialyltranferase 2 ADCY2 - adenylate cyclase 2 (brain) GNG3 - guanine nucleotide binding protein (g protein), gamma 3 ATCAY - ataxia, cerebellar, cayman type FEM1A - fem-1 homolog a (c. elegans) PNCK - pregnancy up-regulated non-ubiquitously expressed cam kinase TMEM55A - transmembrane protein 55a MED20 - mediator complex subunit 20 CSAD - cysteine sulfinic acid decarboxylase PLD3 - phospholipase d family, member 3 GNA15 - guanine nucleotide binding protein (g protein), alpha 15 (gq class) POLD2 - polymerase (dna directed), delta 2, accessory subunit GNA11 - guanine nucleotide binding protein (g protein), alpha 11 (gq class) POLD1 - polymerase (dna directed), delta 1, catalytic subunit PGS1 - phosphatidylglycerophosphate synthase 1 POLR2H - polymerase (rna) ii (dna directed) polypeptide h POLR2E - polymerase (rna) ii (dna directed) polypeptide e, 25kda MBTPS2 - membrane-bound transcription factor peptidase, site 2 RUVBL2 - ruvb-like 2 (e. coli) PRKAA2 - protein kinase, amp-activated, alpha 2 catalytic subunit MYO15A - myosin xva NAGPA - n-acetylglucosamine-1-phosphodiester alpha-n-acetylglucosaminidase PRKAB1 - protein kinase, amp-activated, beta 1 non-catalytic subunit AGMAT - agmatine ureohydrolase (agmatinase) NAT6 - n-acetyltransferase 6 (gcn5-related) PREP - prolyl endopeptidase ADRBK2 - adrenergic, beta, receptor kinase 2 KIF3C - kinesin family member 3c AGK - acylglycerol kinase ADAMTS8 - adam metallopeptidase with thrombospondin type 1 motif, 8 ADRBK1 - adrenergic, beta, receptor kinase 1 KIF5A - kinesin family member 5a PTPRT - protein tyrosine phosphatase, receptor type, t ABCC8 - atp-binding cassette, sub-family c (cftr/mrp), member 8 KHK - ketohexokinase (fructokinase) PPP3R1 - protein phosphatase 3, regulatory subunit b, alpha CIT - citron (rho-interacting, serine/threonine kinase 21) PPP5C - protein phosphatase 5, catalytic subunit CTSK - cathepsin k CSGALNACT1 - chondroitin sulfate n-acetylgalactosaminyltransferase 1 SPTSSB - serine palmitoyltransferase, small subunit b CTSD - cathepsin d TRIM17 - tripartite motif containing 17 USP7 - ubiquitin specific peptidase 7 (herpes virus-associated) LNX1 - ligand of numb-protein x 1, e3 ubiquitin protein ligase XRCC6 - x-ray repair complementing defective repair in chinese hamster cells 6 NUDT14 - nudix (nucleoside diphosphate linked moiety x)-type motif 14 ABHD2 - abhydrolase domain containing 2 KIF25 - kinesin family member 25 DDX51 - dead (asp-glu-ala-asp) box polypeptide 51 CYB561 - cytochrome b561 CAMKK1 - calcium/calmodulin-dependent protein kinase kinase 1, alpha NUDT18 - nudix (nucleoside diphosphate linked moiety x)-type motif 18 PPM1G - protein phosphatase, mg2+/mn2+ dependent, 1g MED10 - mediator complex subunit 10 STK11 - serine/threonine kinase 11 ALAS1 - aminolevulinate, delta-, synthase 1 ABHD16A - abhydrolase domain containing 16a LONP1 - lon peptidase 1, mitochondrial KANSL3 - kat8 regulatory nsl complex subunit 3 RNF165 - ring finger protein 165 SULT4A1 - sulfotransferase family 4a, member 1 B4GALNT1 - beta-1,4-n-acetyl-galactosaminyl transferase 1 EEFSEC - eukaryotic elongation factor, selenocysteine-trna-specific ST14 - suppression of tumorigenicity 14 (colon carcinoma) AGPAT9 - 1-acylglycerol-3-phosphate o-acyltransferase 9 GAK - cyclin g associated kinase PRPS2 - phosphoribosyl pyrophosphate synthetase 2 MGAT4C - mannosyl (alpha-1,3-)-glycoprotein beta-1,4-n-acetylglucosaminyltransferase, isozyme c (putative) PRPS1 - phosphoribosyl pyrophosphate synthetase 1 CYP4X1 - cytochrome p450, family 4, subfamily x, polypeptide 1 RAB26 - rab26, member ras oncogene family PRODH - proline dehydrogenase (oxidase) 1 RHBDL3 - rhomboid, veinlet-like 3 (drosophila) PRKY - protein kinase, y-linked, pseudogene AGBL5 - atp/gtp binding protein-like 5 ZDHHC16 - zinc finger, dhhc-type containing 16 ADTRP - androgen-dependent tfpi-regulating protein ABHD14A - abhydrolase domain containing 14a ITPKA - inositol-trisphosphate 3-kinase a DHX35 - deah (asp-glu-ala-his) box polypeptide 35 MAP2K1 - mitogen-activated protein kinase kinase 1 MAP2K2 - mitogen-activated protein kinase kinase 2 GAD1 - glutamate decarboxylase 1 (brain, 67kda) PHF20 - phd finger protein 20 MAPK10 - mitogen-activated protein kinase 10 HSD11B1L - hydroxysteroid (11-beta) dehydrogenase 1-like MAPK11 - mitogen-activated protein kinase 11 METAP2 - methionyl aminopeptidase 2 MAPK3 - mitogen-activated protein kinase 3 CCNI - cyclin i PRKG2 - protein kinase, cgmp-dependent, type ii GALNT11 - udp-n-acetyl-alpha-d-galactosamine:polypeptide n-acetylgalactosaminyltransferase 11 (galnac-t11) PTPN5 - protein tyrosine phosphatase, non-receptor type 5 (striatum-enriched) CSNK1E - casein kinase 1, epsilon AGBL4 - atp/gtp binding protein-like 4 PRKCE - protein kinase c, epsilon RNF123 - ring finger protein 123 PRKCB - protein kinase c, beta BRMS1L - breast cancer metastasis-suppressor 1-like SLC27A2 - solute carrier family 27 (fatty acid transporter), member 2 SLC27A4 - solute carrier family 27 (fatty acid transporter), member 4 UBE2O - ubiquitin-conjugating enzyme e2o FBXL2 - f-box and leucine-rich repeat protein 2 C9orf69 - chromosome 9 open reading frame 69 DHX8 - deah (asp-glu-ala-his) box polypeptide 8 MTPAP - mitochondrial poly(a) polymerase FN3KRP - fructosamine 3 kinase related protein HDAC10 - histone deacetylase 10 DDB2 - damage-specific dna binding protein 2, 48kda COQ3 - coenzyme q3 methyltransferase MAP3K9 - mitogen-activated protein kinase kinase kinase 9 NEIL1 - nei endonuclease viii-like 1 (e. coli) UBE3B - ubiquitin protein ligase e3b GSTT2 - glutathione s-transferase theta 2 TRIM25 - tripartite motif containing 25 TUBA8 - tubulin, alpha 8 CERS6 - ceramide synthase 6 NUDT11 - nudix (nucleoside diphosphate linked moiety x)-type motif 11 USP43 - ubiquitin specific peptidase 43 MTHFD1L - methylenetetrahydrofolate dehydrogenase (nadp+ dependent) 1-like LRRK1 - leucine-rich repeat kinase 1 NLK - nemo-like kinase YRDC - yrdc domain containing (e. coli) TRIM58 - tripartite motif containing 58 FBXO40 - f-box protein 40 MMP16 - matrix metallopeptidase 16 (membrane-inserted) MMP17 - matrix metallopeptidase 17 (membrane-inserted) PAH - phenylalanine hydroxylase TBRG4 - transforming growth factor beta regulator 4 PAK1 - p21 protein (cdc42/rac)-activated kinase 1 OXR1 - oxidation resistance 1 MARCH11 - membrane-associated ring finger (c3hc4) 11 BRPF1 - bromodomain and phd finger containing, 1 RNF31 - ring finger protein 31 FGFRL1 - fibroblast growth factor receptor-like 1 ADAM11 - adam metallopeptidase domain 11 CTU2 - cytosolic thiouridylase subunit 2 homolog (s. pombe) TUBA1A - tubulin, alpha 1a CYP2E1 - cytochrome p450, family 2, subfamily e, polypeptide 1 IPCEF1 - interaction protein for cytohesin exchange factors 1 ABCB8 - atp-binding cassette, sub-family b (mdr/tap), member 8 CHD5 - chromodomain helicase dna binding protein 5 ABHD8 - abhydrolase domain containing 8 PDPK1 - 3-phosphoinositide dependent protein kinase-1 RAB24 - rab24, member ras oncogene family ARG1 - arginase 1 RPAP1 - rna polymerase ii associated protein 1 MBLAC1 - metallo-beta-lactamase domain containing 1 HAGH - hydroxyacylglutathione hydrolase A4GALT - alpha 1,4-galactosyltransferase ATP5SL - atp5s-like DCLK1 - doublecortin-like kinase 1 ARSJ - arylsulfatase family, member j DAPK3 - death-associated protein kinase 3 ASNA1 - arsa arsenite transporter, atp-binding, homolog 1 (bacterial) WDR60 - wd repeat domain 60 PRDM2 - pr domain containing 2, with znf domain ZDHHC23 - zinc finger, dhhc-type containing 23 ACOT4 - acyl-coa thioesterase 4 DBH - dopamine beta-hydroxylase (dopamine beta-monooxygenase) DGKA - diacylglycerol kinase, alpha 80kda MET - met proto-oncogene PCSK2 - proprotein convertase subtilisin/kexin type 2 MAP3K13 - mitogen-activated protein kinase kinase kinase 13 LRSAM1 - leucine rich repeat and sterile alpha motif containing 1 ASS1 - argininosuccinate synthase 1 PCSK1 - proprotein convertase subtilisin/kexin type 1 ART3 - adp-ribosyltransferase 3 MAPK12 - mitogen-activated protein kinase 12 PDE1A - phosphodiesterase 1a, calmodulin-dependent SARS - seryl-trna synthetase DHRS7B - dehydrogenase/reductase (sdr family) member 7b PDE2A - phosphodiesterase 2a, cgmp-stimulated CAPN14 - calpain 14 NEURL - neuralized homolog (drosophila) TUBB8 - tubulin, beta 8 class viii PGBD5 - piggybac transposable element derived 5 CYP26A1 - cytochrome p450, family 26, subfamily a, polypeptide 1 PDE4A - phosphodiesterase 4a, camp-specific ANAPC11 - anaphase promoting complex subunit 11 NUDT4P1 - nudix (nucleoside diphosphate linked moiety x)-type motif 4 pseudogene 1 RHEBL1 - ras homolog enriched in brain like 1 ATP12A - atpase, h+/k+ transporting, nongastric, alpha polypeptide ATP1A1 - atpase, na+/k+ transporting, alpha 1 polypeptide CDK14 - cyclin-dependent kinase 14 PGD - phosphogluconate dehydrogenase GOT1 - glutamic-oxaloacetic transaminase 1, soluble ATP1A4 - atpase, na+/k+ transporting, alpha 4 polypeptide DPP4 - dipeptidyl-peptidase 4 RAB6B - rab6b, member ras oncogene family MORC2 - morc family cw-type zinc finger 2 STAMBPL1 - stam binding protein-like 1 DPAGT1 - dolichyl-phosphate (udp-n-acetylglucosamine) n-acetylglucosaminephosphotransferase 1 (glcnac-1-p transferase) ATP6V0D1 - atpase, h+ transporting, lysosomal 38kda, v0 subunit d1 SIRT6 - sirtuin 6 SIRT7 - sirtuin 7 SKIV2L - superkiller viralicidic activity 2-like (s. cerevisiae) DUSP6 - dual specificity phosphatase 6 PIGQ - phosphatidylinositol glycan anchor biosynthesis, class q PEPD - peptidase d ATP6V1B1 - atpase, h+ transporting, lysosomal 56/58kda, v1 subunit b1 MATK - megakaryocyte-associated tyrosine kinase STYK1 - serine/threonine/tyrosine kinase 1 PFKP - phosphofructokinase, platelet ATP2B4 - atpase, ca++ transporting, plasma membrane 4 ATP2B3 - atpase, ca++ transporting, plasma membrane 3 METTL21C - methyltransferase like 21c PFAS - phosphoribosylformylglycinamidine synthase DUSP2 - dual specificity phosphatase 2 MAP3K6 - mitogen-activated protein kinase kinase kinase 6 DECR2 - 2,4-dienoyl coa reductase 2, peroxisomal DTX1 - deltex homolog 1 (drosophila) ATP8B1 - atpase, aminophospholipid transporter, class i, type 8b, member 1 GRK6 - g protein-coupled receptor kinase 6 PAPSS2 - 3'-phosphoadenosine 5'-phosphosulfate synthase 2 FBXL15 - f-box and leucine-rich repeat protein 15 PANK4 - pantothenate kinase 4 GPX1 - glutathione peroxidase 1 PIN1 - peptidylprolyl cis/trans isomerase, nima-interacting 1 TSPAN17 - tetraspanin 17 PI4KA - phosphatidylinositol 4-kinase, catalytic, alpha DPP10 - dipeptidyl-peptidase 10 (non-functional) DHCR7 - 7-dehydrocholesterol reductase USP28 - ubiquitin specific peptidase 28 ST3GAL3 - st3 beta-galactoside alpha-2,3-sialyltransferase 3 DHPS - deoxyhypusine synthase ASB2 - ankyrin repeat and socs box containing 2 MYH7B - myosin, heavy chain 7b, cardiac muscle, beta B3GNTL1 - udp-glcnac:betagal beta-1,3-n-acetylglucosaminyltransferase-like 1 PLA2G4A - phospholipase a2, group iva (cytosolic, calcium-dependent) ST3GAL1 - st3 beta-galactoside alpha-2,3-sialyltransferase 1 SESN2 - sestrin 2 RASD1 - ras, dexamethasone-induced 1 KIF1A - kinesin family member 1a CDK5RAP1 - cdk5 regulatory subunit associated protein 1 EPHX4 - epoxide hydrolase 4 BRSK2 - br serine/threonine kinase 2 DIO2 - deiodinase, iodothyronine, type ii LTK - leukocyte receptor tyrosine kinase RHBDL1 - rhomboid, veinlet-like 1 (drosophila) RHOV - ras homolog family member v BDH1 - 3-hydroxybutyrate dehydrogenase, type 1 ABCC10 - atp-binding cassette, sub-family c (cftr/mrp), member 10 MGLL - monoglyceride lipase FBXW2 - f-box and wd repeat domain containing 2 PIAS4 - protein inhibitor of activated stat, 4 ACOT7 - acyl-coa thioesterase 7 KALRN - kalirin, rhogef kinase PHKG2 - phosphorylase kinase, gamma 2 (testis) RPS6KA4 - ribosomal protein s6 kinase, 90kda, polypeptide 4 RPUSD3 - rna pseudouridylate synthase domain containing 3 PIK3CD - phosphatidylinositol-4,5-bisphosphate 3-kinase, catalytic subunit delta PIM1 - pim-1 oncogene DNM1 - dynamin 1 UBE2D4 - ubiquitin-conjugating enzyme e2d 4 (putative) NAV2 - neuron navigator 2 GSK3A - glycogen synthase kinase 3 alpha PGAM5 - phosphoglycerate mutase family member 5 P4HA2 - prolyl 4-hydroxylase, alpha polypeptide ii B4GALT7 - xylosylprotein beta 1,4-galactosyltransferase, polypeptide 7 SENP3 - sumo1/sentrin/smt3 specific peptidase 3 EXOSC5 - exosome component 5 RFC2 - replication factor c (activator 1) 2, 40kda DHX30 - deah (asp-glu-ala-his) box helicase 30 BCKDK - branched chain ketoacid dehydrogenase kinase HSD11B1 - hydroxysteroid (11-beta) dehydrogenase 1 DHRS11 - dehydrogenase/reductase (sdr family) member 11 DHRS7C - dehydrogenase/reductase (sdr family) member 7c RET - ret proto-oncogene TRIM7 - tripartite motif containing 7 CCNO - cyclin o CDKL1 - cyclin-dependent kinase-like 1 (cdc2-related kinase) TUBA4A - tubulin, alpha 4a TUBA3C - tubulin, alpha 3c DUSP27 - dual specificity phosphatase 27 (putative) STEAP1 - six transmembrane epithelial antigen of the prostate 1 SPHK2 - sphingosine kinase 2 RGS7 - regulator of g-protein signaling 7 BMP2 - bone morphogenetic protein 2 STK19 - serine/threonine kinase 19 RNF215 - ring finger protein 215 RGS4 - regulator of g-protein signaling 4 NME6 - nme/nm23 nucleoside diphosphate kinase 6 RIMKLA - ribosomal modification protein rimk-like family member a TSTA3 - tissue specific transplantation antigen p35b MAST1 - microtubule associated serine/threonine kinase 1 MYO5B - myosin vb ST8SIA5 - st8 alpha-n-acetyl-neuraminide alpha-2,8-sialyltransferase 5 CTDSPL - ctd (carboxy-terminal domain, rna polymerase ii, polypeptide a) small phosphatase-like EEF1A2 - eukaryotic translation elongation factor 1 alpha 2 MPPED1 - metallophosphoesterase domain containing 1 CCNA1 - cyclin a1 TOP3A - topoisomerase (dna) iii alpha CA4 - carbonic anhydrase iv PITPNM3 - pitpnm family member 3 PRDM8 - pr domain containing 8 CA7 - carbonic anhydrase vii MAST3 - microtubule associated serine/threonine kinase 3 GOLGA7B - golgin a7 family, member b MYO16 - myosin xvi PDZRN3 - pdz domain containing ring finger 3 EFNA3 - ephrin-a3 SNRNP200 - small nuclear ribonucleoprotein 200kda (u5) DIRAS2 - diras family, gtp-binding ras-like 2 PDZRN4 - pdz domain containing ring finger 4 POP7 - processing of precursor 7, ribonuclease p/mrp subunit (s. cerevisiae) E4F1 - e4f transcription factor 1 NT5M - 5',3'-nucleotidase, mitochondrial FIGNL2 - fidgetin-like 2 ALKBH4 - alkb, alkylation repair homolog 4 (e. coli) KIF21B - kinesin family member 21b FTSJ2 - ftsj rna methyltransferase homolog 2 (e. coli) SMYD2 - set and mynd domain containing 2 MVD - mevalonate (diphospho) decarboxylase TRHDE - thyrotropin-releasing hormone degrading enzyme TRIM37 - tripartite motif containing 37 EDNRA - endothelin receptor type a ST6GALNAC5 - st6 (alpha-n-acetyl-neuraminyl-2,3-beta-galactosyl-1,3)-n-acetylgalactosaminide alpha-2,6-sialyltransferase 5 GMPPA - gdp-mannose pyrophosphorylase a CA10 - carbonic anhydrase x GMPPB - gdp-mannose pyrophosphorylase b TRAF3 - tnf receptor-associated factor 3 TEX30 - testis expressed 30 ARL4C - adp-ribosylation factor-like 4c NEURL3 - neuralized homolog 3 (drosophila) pseudogene HECW1 - hect, c2 and ww domain containing e3 ubiquitin protein ligase 1 DUSP26 - dual specificity phosphatase 26 (putative) MARCH9 - membrane-associated ring finger (c3hc4) 9 PAK6 - p21 protein (cdc42/rac)-activated kinase 6 PRSS16 - protease, serine, 16 (thymus) UQCRC1 - ubiquinol-cytochrome c reductase core protein i MTR - 5-methyltetrahydrofolate-homocysteine methyltransferase CAPNS1 - calpain, small subunit 1 PPIE - peptidylprolyl isomerase e (cyclophilin e) UROD - uroporphyrinogen decarboxylase CAMK1D - calcium/calmodulin-dependent protein kinase id MCRS1 - microspherule protein 1 CAMK2G - calcium/calmodulin-dependent protein kinase ii gamma HMBS - hydroxymethylbilane synthase CAMK2A - calcium/calmodulin-dependent protein kinase ii alpha PTK2B - protein tyrosine kinase 2 beta CUL9 - cullin 9 C12orf5 - chromosome 12 open reading frame 5 FARSA - phenylalanyl-trna synthetase, alpha subunit CARS - cysteinyl-trna synthetase AACS - acetoacetyl-coa synthetase FASN - fatty acid synthase FAP - fibroblast activation protein, alpha CAD - carbamoyl-phosphate synthetase 2, aspartate transcarbamylase, and dihydroorotase PINK1 - pten induced putative kinase 1 HK1 - hexokinase 1 SPSB3 - spla/ryanodine receptor domain and socs box containing 3 MED16 - mediator complex subunit 16 UBE2E3 - ubiquitin-conjugating enzyme e2e 3 HERC6 - hect and rld domain containing e3 ubiquitin protein ligase family member 6 ADCK2 - aarf domain containing kinase 2 EXTL1 - exostosin-like glycosyltransferase 1 KIFC2 - kinesin family member c2 SAE1 - sumo1 activating enzyme subunit 1 EXTL3 - exostosin-like glycosyltransferase 3 VARS - valyl-trna synthetase STK25 - serine/threonine kinase 25 PDXP - pyridoxal (pyridoxine, vitamin b6) phosphatase B3GALT2 - udp-gal:betaglcnac beta 1,3-galactosyltransferase, polypeptide 2 JOSD1 - josephin domain containing 1 ADCK5 - aarf domain containing kinase 5 TYRP1 - tyrosinase-related protein 1 CTSF - cathepsin f TUBA1B - tubulin, alpha 1b ADAM19 - adam metallopeptidase domain 19 ADAM32 - adam metallopeptidase domain 32 MACROD1 - macro domain containing 1 CCNE1 - cyclin e1 NTMT1 - n-terminal xaa-pro-lys n-methyltransferase 1 GPAA1 - glycosylphosphatidylinositol anchor attachment 1 CPSF3L - cleavage and polyadenylation specific factor 3-like CCNG2 - cyclin g2 GFPT2 - glutamine-fructose-6-phosphate transaminase 2 CAMK1G - calcium/calmodulin-dependent protein kinase ig ZADH2 - zinc binding alcohol dehydrogenase domain containing 2 XYLB - xylulokinase homolog (h. influenzae) ASPHD2 - aspartate beta-hydroxylase domain containing 2 UCKL1 - uridine-cytidine kinase 1-like 1 FBXO6 - f-box protein 6 ACAD9 - acyl-coa dehydrogenase family, member 9 ADAM23 - adam metallopeptidase domain 23 TYRO3 - tyro3 protein tyrosine kinase TYK2 - tyrosine kinase 2 PMPCA - peptidase (mitochondrial processing) alpha EPHA3 - eph receptor a3 PKDCC - protein kinase domain containing, cytoplasmic HS3ST2 - heparan sulfate (glucosamine) 3-o-sulfotransferase 2 SLC25A42 - solute carrier family 25, member 42 EXD3 - exonuclease 3'-5' domain containing 3 EPHB3 - eph receptor b3 EPHA5 - eph receptor a5 EPHA4 - eph receptor a4 SPATA20 - spermatogenesis associated 20 HS3ST4 - heparan sulfate (glucosamine) 3-o-sulfotransferase 4 DTX4 - deltex homolog 4 (drosophila) ATP13A1 - atpase type 13a1 EPHB6 - eph receptor b6 RGS11 - regulator of g-protein signaling 11 CCND2 - cyclin d2 RNPEPL1 - arginyl aminopeptidase (aminopeptidase b)-like 1 ADCK1 - aarf domain containing kinase 1 PAK7 - p21 protein (cdc42/rac)-activated kinase 7 ERCC2 - excision repair cross-complementing rodent repair deficiency, complementation group 2 MAN1C1 - mannosidase, alpha, class 1c, member 1 MPG - n-methylpurine-dna glycosylase MPI - mannose phosphate isomerase APMAP - adipocyte plasma membrane associated protein CDIPT - cdp-diacylglycerol--inositol 3-phosphatidyltransferase PRSS3 - protease, serine, 3 RMND5A - required for meiotic nuclear division 5 homolog a (s. cerevisiae) LGMN - legumain KCNAB2 - potassium voltage-gated channel, shaker-related subfamily, beta member 2 SSH1 - slingshot protein phosphatase 1 PRSS1 - protease, serine, 1 (trypsin 1) FLT3 - fms-related tyrosine kinase 3 TUBA3D - tubulin, alpha 3d SETDB1 - set domain, bifurcated 1 PPP2R2D - protein phosphatase 2, regulatory subunit b, delta ADPRHL1 - adp-ribosylhydrolase like 1 KLK7 - kallikrein-related peptidase 7 RMND5B - required for meiotic nuclear division 5 homolog b (s. cerevisiae) PSEN2 - presenilin 2 (alzheimer disease 4) NAT8L - n-acetyltransferase 8-like (gcn5-related, putative) ADC - arginine decarboxylase IMPA2 - inositol(myo)-1(or 4)-monophosphatase 2 PLCB1 - phospholipase c, beta 1 (phosphoinositide-specific) POLL - polymerase (dna directed), lambda PRPF19 - pre-mrna processing factor 19 RAB15 - rab15, member ras oncogene family PRSS12 - protease, serine, 12 (neurotrypsin, motopsin) SIN3B - sin3 transcription regulator family member b ENTPD6 - ectonucleoside triphosphate diphosphohydrolase 6 (putative) METTL21A - methyltransferase like 21a ZDHHC22 - zinc finger, dhhc-type containing 22 RNF148 - ring finger protein 148 GLB1L3 - galactosidase, beta 1-like 3 RPUSD1 - rna pseudouridylate synthase domain containing 1 HR - hair growth associated UBE2QL1 - ubiquitin-conjugating enzyme e2q family-like 1 NUAK1 - nuak family, snf1-like kinase, 1 RASL10A - ras-like, family 10, member a PSMD2 - proteasome (prosome, macropain) 26s subunit, non-atpase, 2 DGKZ - diacylglycerol kinase, zeta ATG7 - autophagy related 7 LEPREL2 - leprecan-like 2 TAF5 - taf5 rna polymerase ii, tata box binding protein (tbp)-associated factor, 100kda DCTN2 - dynactin 2 (p50) NTHL1 - nth endonuclease iii-like 1 (e. coli) CES4A - carboxylesterase 4a DPY19L1 - dpy-19-like 1 (c. elegans) FES - feline sarcoma oncogene PTGS2 - prostaglandin-endoperoxide synthase 2 (prostaglandin g/h synthase and cyclooxygenase) DHX38 - deah (asp-glu-ala-his) box polypeptide 38 MBLAC2 - metallo-beta-lactamase domain containing 2 KMO - kynurenine 3-monooxygenase (kynurenine 3-hydroxylase) CDK5 - cyclin-dependent kinase 5 NOSIP - nitric oxide synthase interacting protein CDK9 - cyclin-dependent kinase 9 RND1 - rho family gtpase 1 MTMR14 - myotubularin related protein 14 PDE8B - phosphodiesterase 8b CDKN3 - cyclin-dependent kinase inhibitor 3 TAZ - tafazzin PTPN3 - protein tyrosine phosphatase, non-receptor type 3 PTPN9 - protein tyrosine phosphatase, non-receptor type 9 TNNI3K - tnni3 interacting kinase FADS6 - fatty acid desaturase 6 ZFP91 - zfp91 zinc finger protein RNF40 - ring finger protein 40, e3 ubiquitin protein ligase AGPAT2 - 1-acylglycerol-3-phosphate o-acyltransferase 2 RNF208 - ring finger protein 208 PTPRF - protein tyrosine phosphatase, receptor type, f GPN2 - gpn-loop gtpase 2 GTPBP2 - gtp binding protein 2 P4HTM - prolyl 4-hydroxylase, transmembrane (endoplasmic reticulum) NOS2 - nitric oxide synthase 2, inducible PTPRR - protein tyrosine phosphatase, receptor type, r MUS81 - mus81 structure-specific endonuclease subunit B4GALNT4 - beta-1,4-n-acetyl-galactosaminyl transferase 4 CHRM3 - cholinergic receptor, muscarinic 3 NME1 - nme/nm23 nucleoside diphosphate kinase 1 STK32C - serine/threonine kinase 32c MYO19 - myosin xix INPP5J - inositol polyphosphate-5-phosphatase j LYZL4 - lysozyme-like 4 CHKB - choline kinase beta SIRT4 - sirtuin 4 VKORC1L1 - vitamin k epoxide reductase complex, subunit 1-like 1 MAP4K2 - mitogen-activated protein kinase kinase kinase kinase 2 ARSK - arylsulfatase family, member k ABCB9 - atp-binding cassette, sub-family b (mdr/tap), member 9 RAB3A - rab3a, member ras oncogene family GLS2 - glutaminase 2 (liver, mitochondrial) PLA2G6 - phospholipase a2, group vi (cytosolic, calcium-independent) PIP5K1B - phosphatidylinositol-4-phosphate 5-kinase, type i, beta PTGES2 - prostaglandin e synthase 2 TNFAIP1 - tumor necrosis factor, alpha-induced protein 1 (endothelial) PIF1 - pif1 5'-to-3' dna helicase TUBG2 - tubulin, gamma 2 NIT1 - nitrilase 1 RAC2 - ras-related c3 botulinum toxin substrate 2 (rho family, small gtp binding protein rac2) RPP40 - ribonuclease p/mrp 40kda subunit XYLT1 - xylosyltransferase i TNNT3 - troponin t type 3 (skeletal, fast) INO80 - ino80 complex subunit EHD2 - eh-domain containing 2 TNNT2 - troponin t type 2 (cardiac) DDX56 - dead (asp-glu-ala-asp) box helicase 56 RAB27B - rab27b, member ras oncogene family HYAL3 - hyaluronoglucosaminidase 3 WDR82 - wd repeat domain 82 FUT9 - fucosyltransferase 9 (alpha (1,3) fucosyltransferase) PGM2L1 - phosphoglucomutase 2-like 1 EGLN2 - egl-9 family hypoxia-inducible factor 2 INPP5E - inositol polyphosphate-5-phosphatase, 72 kda MICAL2 - microtubule associated monooxygenase, calponin and lim domain containing 2 TERF2 - telomeric repeat binding factor 2 CYP26B1 - cytochrome p450, family 26, subfamily b, polypeptide 1 TRUB2 - trub pseudouridine (psi) synthase homolog 2 (e. coli) PPM1F - protein phosphatase, mg2+/mn2+ dependent, 1f TESK1 - testis-specific kinase 1 GALNT9 - udp-n-acetyl-alpha-d-galactosamine:polypeptide n-acetylgalactosaminyltransferase 9 (galnac-t9) TPK1 - thiamin pyrophosphokinase 1 LHPP - phospholysine phosphohistidine inorganic pyrophosphate phosphatase FLAD1 - flavin adenine dinucleotide synthetase 1 OTOGL - otogelin-like CLK2 - cdc-like kinase 2 RGS6 - regulator of g-protein signaling 6 ACAD8 - acyl-coa dehydrogenase family, member 8 TRMT2A - trna methyltransferase 2 homolog a (s. cerevisiae) CHPF2 - chondroitin polymerizing factor 2 NDUFB3 - nadh dehydrogenase (ubiquinone) 1 beta subcomplex, 3, 12kda DHX16 - deah (asp-glu-ala-his) box polypeptide 16 PPIP5K1 - diphosphoinositol pentakisphosphate kinase 1 CKMT1B - creatine kinase, mitochondrial 1b TPST2 - tyrosylprotein sulfotransferase 2 TARS2 - threonyl-trna synthetase 2, mitochondrial (putative) ADAT1 - adenosine deaminase, trna-specific 1 SULT1A4 - sulfotransferase family, cytosolic, 1a, phenol-preferring, member 4 RPUSD2 - rna pseudouridylate synthase domain containing 2 NAA50 - n(alpha)-acetyltransferase 50, nate catalytic subunit RHOF - ras homolog family member f (in filopodia) |
| GO:0099508 | voltage-gated ion channel activity involved in regulation of presynaptic membrane potential | 3.19E-4 | 2.14E-2 | 18.23 (16750,3,919,3) | KCNA1 - potassium voltage-gated channel, shaker-related subfamily, member 1 (episodic ataxia with myokymia) KCNC1 - potassium voltage-gated channel, shaw-related subfamily, member 1 KCNC2 - potassium voltage-gated channel, shaw-related subfamily, member 2 |
| GO:0044325 | ion channel binding | 3.97E-4 | 2.63E-2 | 3.58 (16750,110,595,14) | KCNIP3 - kv channel interacting protein 3, calsenilin KCNJ11 - potassium inwardly-rectifying channel, subfamily j, member 11 STX1A - syntaxin 1a (brain) SCN1B - sodium channel, voltage-gated, type i, beta subunit DPP10 - dipeptidyl-peptidase 10 (non-functional) ABCC8 - atp-binding cassette, sub-family c (cftr/mrp), member 8 KCNH1 - potassium voltage-gated channel, subfamily h (eag-related), member 1 ANKRD9 - ankyrin repeat domain 9 RIMS3 - regulating synaptic membrane exocytosis 3 KCNC1 - potassium voltage-gated channel, shaw-related subfamily, member 1 KCNH5 - potassium voltage-gated channel, subfamily h (eag-related), member 5 KCNC2 - potassium voltage-gated channel, shaw-related subfamily, member 2 SH3GL1 - sh3-domain grb2-like 1 ANK1 - ankyrin 1, erythrocytic |
| GO:0099529 | neurotransmitter receptor activity involved in regulation of postsynaptic membrane potential | 4.51E-4 | 2.95E-2 | 3.38 (16750,39,1652,13) | CHRNA7 - cholinergic receptor, nicotinic, alpha 7 (neuronal) GRIK1 - glutamate receptor, ionotropic, kainate 1 GRIK2 - glutamate receptor, ionotropic, kainate 2 GRIK3 - glutamate receptor, ionotropic, kainate 3 GRIK4 - glutamate receptor, ionotropic, kainate 4 ADRB1 - adrenoceptor beta 1 GABRA2 - gamma-aminobutyric acid (gaba) a receptor, alpha 2 GABRA1 - gamma-aminobutyric acid (gaba) a receptor, alpha 1 GABRA4 - gamma-aminobutyric acid (gaba) a receptor, alpha 4 GABRA3 - gamma-aminobutyric acid (gaba) a receptor, alpha 3 GABRG3 - gamma-aminobutyric acid (gaba) a receptor, gamma 3 GRIA3 - glutamate receptor, ionotropic, ampa 3 GRIN3A - glutamate receptor, ionotropic, n-methyl-d-aspartate 3a |
| GO:0017048 | Rho GTPase binding | 5.2E-4 | 3.34E-2 | 2.61 (16750,150,813,19) | DAPK3 - death-associated protein kinase 3 NGEF - neuronal guanine nucleotide exchange factor SH3BP1 - sh3-domain binding protein 1 SRGAP3 - slit-robo rho gtpase activating protein 3 KALRN - kalirin, rhogef kinase RASGRF2 - ras protein-specific guanine nucleotide-releasing factor 2 FMNL1 - formin-like 1 PARD6A - par-6 partitioning defective 6 homolog alpha (c. elegans) DVL1 - dishevelled segment polarity protein 1 NOXA1 - nadph oxidase activator 1 DVL3 - dishevelled segment polarity protein 3 TIAM2 - t-cell lymphoma invasion and metastasis 2 STRIP1 - striatin interacting protein 1 CDC42EP3 - cdc42 effector protein (rho gtpase binding) 3 PAK1 - p21 protein (cdc42/rac)-activated kinase 1 PAK7 - p21 protein (cdc42/rac)-activated kinase 7 ITPKA - inositol-trisphosphate 3-kinase a PAK6 - p21 protein (cdc42/rac)-activated kinase 6 ARHGEF25 - rho guanine nucleotide exchange factor (gef) 25 |
| GO:0098960 | postsynaptic neurotransmitter receptor activity | 7.75E-4 | 4.91E-2 | 3.21 (16750,41,1652,13) | CHRNA7 - cholinergic receptor, nicotinic, alpha 7 (neuronal) GRIK1 - glutamate receptor, ionotropic, kainate 1 GRIK2 - glutamate receptor, ionotropic, kainate 2 GRIK3 - glutamate receptor, ionotropic, kainate 3 GRIK4 - glutamate receptor, ionotropic, kainate 4 ADRB1 - adrenoceptor beta 1 GABRA2 - gamma-aminobutyric acid (gaba) a receptor, alpha 2 GABRA1 - gamma-aminobutyric acid (gaba) a receptor, alpha 1 GABRA4 - gamma-aminobutyric acid (gaba) a receptor, alpha 4 GABRA3 - gamma-aminobutyric acid (gaba) a receptor, alpha 3 GABRG3 - gamma-aminobutyric acid (gaba) a receptor, gamma 3 GRIA3 - glutamate receptor, ionotropic, ampa 3 GRIN3A - glutamate receptor, ionotropic, n-methyl-d-aspartate 3a |
| GO:0015319 | sodium:inorganic phosphate symporter activity | 7.76E-4 | 4.85E-2 | 1,288.46 (16750,1,13,1) | SLC17A7 - solute carrier family 17 (vesicular glutamate transporter), member 7 |
| GO:0008068 | extracellularly glutamate-gated chloride channel activity | 7.76E-4 | 4.79E-2 | 1,288.46 (16750,1,13,1) | SLC17A7 - solute carrier family 17 (vesicular glutamate transporter), member 7 |
| GO:1904315 | transmitter-gated ion channel activity involved in regulation of postsynaptic membrane potential | 7.89E-4 | 4.8E-2 | 3.38 (16750,36,1652,12) | GABRG3 - gamma-aminobutyric acid (gaba) a receptor, gamma 3 GRIK1 - glutamate receptor, ionotropic, kainate 1 CHRNA7 - cholinergic receptor, nicotinic, alpha 7 (neuronal) GRIK2 - glutamate receptor, ionotropic, kainate 2 GRIK3 - glutamate receptor, ionotropic, kainate 3 GRIK4 - glutamate receptor, ionotropic, kainate 4 GABRA2 - gamma-aminobutyric acid (gaba) a receptor, alpha 2 GABRA1 - gamma-aminobutyric acid (gaba) a receptor, alpha 1 GRIA3 - glutamate receptor, ionotropic, ampa 3 GABRA4 - gamma-aminobutyric acid (gaba) a receptor, alpha 4 GABRA3 - gamma-aminobutyric acid (gaba) a receptor, alpha 3 GRIN3A - glutamate receptor, ionotropic, n-methyl-d-aspartate 3a |
| GO:0043176 | amine binding | 8.11E-4 | 4.87E-2 | 11.33 (16750,8,739,4) | HTR5A - 5-hydroxytryptamine (serotonin) receptor 5a, g protein-coupled HTR1A - 5-hydroxytryptamine (serotonin) receptor 1a, g protein-coupled HTR2A - 5-hydroxytryptamine (serotonin) receptor 2a, g protein-coupled HTR1F - 5-hydroxytryptamine (serotonin) receptor 1f, g protein-coupled |
| GO:0051378 | serotonin binding | 8.11E-4 | 4.81E-2 | 11.33 (16750,8,739,4) | HTR5A - 5-hydroxytryptamine (serotonin) receptor 5a, g protein-coupled HTR1A - 5-hydroxytryptamine (serotonin) receptor 1a, g protein-coupled HTR2A - 5-hydroxytryptamine (serotonin) receptor 2a, g protein-coupled HTR1F - 5-hydroxytryptamine (serotonin) receptor 1f, g protein-coupled |
| GO:0004999 | vasoactive intestinal polypeptide receptor activity | 8.21E-4 | 4.8E-2 | 45.89 (16750,2,365,2) | VIPR1 - vasoactive intestinal peptide receptor 1 VIPR2 - vasoactive intestinal peptide receptor 2 |
| GO:0004713 | protein tyrosine kinase activity | 9.5E-4 | 5.49E-2 | 2.87 (16750,121,819,17) | EPHA3 - eph receptor a3 FGFRL1 - fibroblast growth factor receptor-like 1 PKDCC - protein kinase domain containing, cytoplasmic FLT3 - fms-related tyrosine kinase 3 EPHB3 - eph receptor b3 EPHA5 - eph receptor a5 EPHA4 - eph receptor a4 MATK - megakaryocyte-associated tyrosine kinase STYK1 - serine/threonine/tyrosine kinase 1 EPHB6 - eph receptor b6 FES - feline sarcoma oncogene PTK2B - protein tyrosine kinase 2 beta RET - ret proto-oncogene TYRO3 - tyro3 protein tyrosine kinase TYK2 - tyrosine kinase 2 MAP2K1 - mitogen-activated protein kinase kinase 1 LTK - leukocyte receptor tyrosine kinase |

b)

| **Description** | **P-value** | **FDR q-value** | **Enrichment (N, B, n, b)** |
| --- | --- | --- | --- |
| gated channel activity | 1.73E-14 | 7.8E-11 | 3.05 (16750,306,1114,62) |
| voltage-gated potassium channel activity | 4.88E-14 | 1.1E-10 | 14.24 (16750,80,250,17) |
| cation channel activity | 4.94E-14 | 7.42E-11 | 3.12 (16750,292,1067,58) |
| potassium channel activity | 7.68E-14 | 8.65E-11 | 4.74 (16750,116,1067,35) |
| ion gated channel activity | 1.14E-13 | 1.02E-10 | 3.14 (16750,297,1114,62) |
| voltage-gated cation channel activity | 1.64E-13 | 1.23E-10 | 10.19 (16750,126,261,20) |
| ion channel activity | 9.5E-13 | 6.11E-10 | 2.71 (16750,376,1084,66) |
| potassium ion transmembrane transporter activity | 1.05E-12 | 5.92E-10 | 6.01 (16750,143,507,26) |
| voltage-gated channel activity | 1.57E-12 | 7.88E-10 | 3.59 (16750,176,1114,42) |
| voltage-gated ion channel activity | 1.57E-12 | 7.09E-10 | 3.59 (16750,176,1114,42) |
| substrate-specific channel activity | 2.06E-12 | 8.41E-10 | 2.66 (16750,383,1084,66) |
| metal ion transmembrane transporter activity | 1.97E-11 | 7.41E-9 | 2.56 (16750,398,1067,65) |
| channel activity | 4.88E-11 | 1.69E-8 | 3.38 (16750,410,507,42) |
| passive transmembrane transporter activity | 5.07E-11 | 1.63E-8 | 3.38 (16750,411,507,42) |
| inorganic cation transmembrane transporter activity | 5.25E-10 | 1.58E-7 | 2.92 (16750,531,507,47) |
| cation transmembrane transporter activity | 5.98E-10 | 1.68E-7 | 2.27 (16750,575,924,72) |
| monovalent inorganic cation transmembrane transporter activity | 2.31E-9 | 6.12E-7 | 3.40 (16750,340,507,35) |
| ion transmembrane transporter activity | 3.2E-9 | 7.99E-7 | 1.95 (16750,782,1046,95) |
| ligand-gated channel activity | 3.22E-9 | 7.62E-7 | 4.53 (16750,120,770,25) |
| ligand-gated ion channel activity | 3.22E-9 | 7.24E-7 | 4.53 (16750,120,770,25) |
| transmembrane transporter activity | 4.54E-9 | 9.74E-7 | 1.68 (16750,937,1513,142) |
| inorganic molecular entity transmembrane transporter activity | 7.66E-9 | 1.57E-6 | 1.96 (16750,727,1046,89) |
| transferase activity, transferring phosphorus-containing groups | 9.74E-9 | 1.91E-6 | 1.68 (16750,821,1655,136) |
| kinase activity | 1.68E-8 | 3.14E-6 | 1.74 (16750,681,1655,117) |
| anion binding | 6.86E-8 | 1.24E-5 | 1.33 (16750,2495,1722,341) |
| ligand-gated cation channel activity | 7.83E-8 | 1.36E-5 | 4.29 (16750,93,924,22) |
| phosphotransferase activity, alcohol group as acceptor | 8.61E-8 | 1.44E-5 | 1.74 (16750,624,1655,107) |
| transporter activity | 1.05E-7 | 1.69E-5 | 1.56 (16750,1123,1513,158) |
| purine ribonucleotide binding | 2.8E-7 | 4.35E-5 | 1.40 (16750,1693,1722,243) |
| ribonucleotide binding | 3.75E-7 | 5.63E-5 | 1.39 (16750,1708,1722,244) |
| purine ribonucleoside triphosphate binding | 4.27E-7 | 6.2E-5 | 1.40 (16750,1635,1722,235) |
| purine nucleotide binding | 4.81E-7 | 6.77E-5 | 1.39 (16750,1704,1722,243) |
| protein kinase activity | 9.19E-7 | 1.25E-4 | 1.74 (16750,530,1651,91) |
| calmodulin binding | 1.72E-6 | 2.28E-4 | 4.74 (16750,184,307,16) |
| sodium channel activity | 3.36E-6 | 4.32E-4 | 4.77 (16750,35,1405,14) |
| adenyl ribonucleotide binding | 3.75E-6 | 4.69E-4 | 1.40 (16750,1383,1717,199) |
| ionotropic glutamate receptor activity | 4.01E-6 | 4.87E-4 | 7.15 (16750,15,1405,9) |
| ATP binding | 5.01E-6 | 5.94E-4 | 1.41 (16750,1331,1717,192) |
| drug binding | 5.55E-6 | 6.41E-4 | 1.38 (16750,1552,1668,213) |
| adenyl nucleotide binding | 6.02E-6 | 6.78E-4 | 1.39 (16750,1392,1717,199) |
| inward rectifier potassium channel activity | 7.15E-6 | 7.85E-4 | 16.58 (16750,19,319,6) |
| nucleotide binding | 7.54E-6 | 8.08E-4 | 1.33 (16750,1908,1722,260) |
| nucleoside phosphate binding | 8.33E-6 | 8.73E-4 | 1.32 (16750,1909,1722,260) |
| carbohydrate derivative binding | 9.57E-6 | 9.79E-4 | 1.31 (16750,1999,1744,273) |
| extracellular ligand-gated ion channel activity | 1.58E-5 | 1.58E-3 | 4.99 (16750,61,716,13) |
| small molecule binding | 1.76E-5 | 1.73E-3 | 1.28 (16750,2249,1807,310) |
| neurotransmitter receptor activity | 1.88E-5 | 1.8E-3 | 3.89 (16750,99,739,17) |
| calmodulin-dependent protein kinase activity | 2.57E-5 | 2.42E-3 | 10.63 (16750,28,394,7) |
| glutamate receptor activity | 3.1E-5 | 2.85E-3 | 5.42 (16750,22,1405,10) |
| transferase activity | 3.73E-5 | 3.36E-3 | 1.30 (16750,2057,1615,258) |
| transmitter-gated ion channel activity | 4.12E-5 | 3.64E-3 | 5.36 (16750,48,716,11) |
| transmitter-gated channel activity | 4.12E-5 | 3.57E-3 | 5.36 (16750,48,716,11) |
| cytoskeletal protein binding | 5.2E-5 | 4.41E-3 | 1.44 (16750,872,1808,136) |
| protein binding | 7.01E-5 | 5.85E-3 | 1.08 (16750,10606,1749,1193) |
| G-protein activated inward rectifier potassium channel activity | 7.8E-5 | 6.38E-3 | 39.38 (16750,4,319,3) |
| calcium-dependent phospholipid binding | 1.03E-4 | 8.29E-3 | 3.92 (16750,40,1388,13) |
| catalytic activity, acting on a protein | 1.04E-4 | 8.23E-3 | 1.28 (16750,1971,1717,259) |
| zinc-transporting ATPase activity | 1.19E-4 | 9.27E-3 | 8,375.00 (16750,1,2,1) |
| protein C-terminus binding | 1.35E-4 | 1.03E-2 | 2.01 (16750,169,1774,36) |
| protein serine/threonine kinase activity | 1.41E-4 | 1.06E-2 | 1.67 (16750,400,1651,66) |
| kainate selective glutamate receptor activity | 1.41E-4 | 1.04E-2 | 11.92 (16750,4,1405,4) |
| delayed rectifier potassium channel activity | 1.7E-4 | 1.24E-2 | 5.47 (16750,30,919,9) |
| sodium ion transmembrane transporter activity | 1.98E-4 | 1.41E-2 | 2.15 (16750,132,1713,29) |
| channel regulator activity | 2.31E-4 | 1.62E-2 | 3.91 (16750,133,387,12) |
| neurotransmitter binding | 2.32E-4 | 1.61E-2 | 4.93 (16750,46,739,10) |
| catalytic activity | 2.4E-4 | 1.64E-2 | 1.15 (16750,5134,1720,605) |
| voltage-gated ion channel activity involved in regulation of presynaptic membrane potential | 3.19E-4 | 2.14E-2 | 18.23 (16750,3,919,3) |
| ion channel binding | 3.97E-4 | 2.63E-2 | 3.58 (16750,110,595,14) |
| neurotransmitter receptor activity involved in regulation of postsynaptic membrane potential | 4.51E-4 | 2.95E-2 | 3.38 (16750,39,1652,13) |
| Rho GTPase binding | 5.2E-4 | 3.34E-2 | 2.61 (16750,150,813,19) |
| postsynaptic neurotransmitter receptor activity | 7.75E-4 | 4.91E-2 | 3.21 (16750,41,1652,13) |
| sodium:inorganic phosphate symporter activity | 7.76E-4 | 4.85E-2 | 1,288.46 (16750,1,13,1) |
| extracellularly glutamate-gated chloride channel activity | 7.76E-4 | 4.79E-2 | 1,288.46 (16750,1,13,1) |
| transmitter-gated ion channel activity involved in regulation of postsynaptic membrane potential | 7.89E-4 | 4.8E-2 | 3.38 (16750,36,1652,12) |
| amine binding | 8.11E-4 | 4.87E-2 | 11.33 (16750,8,739,4) |
| serotonin binding | 8.11E-4 | 4.81E-2 | 11.33 (16750,8,739,4) |
| vasoactive intestinal polypeptide receptor activity | 8.21E-4 | 4.8E-2 | 45.89 (16750,2,365,2) |
| protein tyrosine kinase activity | 9.5E-4 | 5.49E-2 | 2.87 (16750,121,819,17) |
