# Supplementary material for: Structural optimality and neurogenetic expression mediate functional dynamics in the human brain
Source: Hum Brain Mapp. 2020 Feb 6;41(8):2229–43. doi: 10.1002/hbm.24942 (PMC7267953; doi:10.1002/hbm.24942)
Supplement: Supplementary file 1 — Appendix S1: Supplementary Information [file HBM-41-2229-s001.zip › HBM_24942_Supplementary_File3.docx]

a)

| Subject | Release | Acquisition | Gender | Age |
| --- | --- | --- | --- | --- |
| 100307 | Q1 | Q01 | F | 26-30 |
| 100408 | Q3 | Q03 | M | 31-35 |
| 101107 | S500 | Q06 | M | 22-25 |
| 103111 | S500 | Q06 | M | 26-30 |
| 103414 | Q2 | Q02 | F | 22-25 |
| 105014 | S500 | Q05 | F | 26-30 |
| 105115 | Q2 | Q02 | M | 31-35 |
| 106016 | Q3 | Q04 | F | 31-35 |
| 108828 | S500 | Q05 | M | 31-35 |
| 110411 | Q2 | Q02 | M | 31-35 |
| 111312 | Q1 | Q01 | F | 31-35 |
| 113619 | Q2 | Q02 | F | 31-35 |
| 114419 | S500 | Q07 | M | 31-35 |
| 115320 | Q2 | Q02 | F | 31-35 |
| 116524 | S500 | Q05 | M | 26-30 |
| 118528 | S500 | Q04 | F | 26-30 |
| 118730 | Q2 | Q03 | M | 22-25 |
| 118932 | Q1 | Q02 | M | 26-30 |
| 120111 | Q3 | Q03 | F | 26-30 |
| 122317 | Q3 | Q04 | M | 31-35 |
| 122620 | S500 | Q05 | M | 26-30 |
| 123117 | Q2 | Q03 | M | 26-30 |
| 123925 | S500 | Q05 | F | 26-30 |
| 124422 | Q2 | Q03 | F | 31-35 |
| 125525 | Q1 | Q01 | F | 31-35 |
| 126325 | Q3 | Q03 | F | 26-30 |
| 127630 | S500 | Q06 | F | 22-25 |
| 127933 | Q3 | Q03 | M | 31-35 |
| 128127 | S500 | Q05 | F | 26-30 |
| 128632 | Q1 | Q02 | F | 31-35 |
| 129028 | Q2 | Q03 | M | 26-30 |
| 130013 | Q1 | Q01 | M | 26-30 |
| 130316 | Q3 | Q03 | F | 26-30 |
| 131217 | S500 | Q06 | F | 26-30 |
| 131722 | S500 | Q06 | F | 26-30 |
| 133019 | S500 | Q06 | F | 26-30 |
| 133928 | Q2 | Q03 | M | 26-30 |
| 135225 | S500 | Q06 | F | 22-25 |
| 135932 | Q2 | Q02 | F | 26-30 |
| 136833 | Q2 | Q02 | M | 31-35 |
| 138534 | Q3 | Q03 | M | 22-25 |
| 139637 | Q2 | Q03 | F | 31-35 |
| 140925 | S500 | Q04 | F | 22-25 |
| 144832 | S500 | Q06 | F | 22-25 |
| 146432 | S500 | Q06 | M | 31-35 |
| 147737 | S500 | Q06 | F | 31-35 |
| 148335 | Q3 | Q04 | F | 31-35 |
| 148840 | S500 | Q04 | F | 22-25 |
| 149337 | Q1 | Q02 | M | 31-35 |
| 149539 | Q2 | Q02 | M | 22-25 |

b)

Right Hemisphere Network membership Overlap

'lateralorbitofrontal_1' 'limbic' 0.55438

'lateralorbitofrontal_2' 'DMN' 0.31938

'lateralorbitofrontal_3' 'fronto-parietal' 0.37977

'lateralorbitofrontal_4' 'limbic' 0.34532

'parsorbitalis_1' 'DMN' 0.52595

'frontalpole_1' 'limbic' 0.57865

'medialorbitofrontal_1' 'DMN' 0.68189

'medialorbitofrontal_2' 'limbic' 0.84718

'medialorbitofrontal_3' 'limbic' 0.83333

'parstriangularis_1' 'ventral-attention' 0.51567

'parstriangularis_2' 'fronto-parietal' 0.5013

'parsopercularis_1 ‘'fronto-parietal' 0.51397

'parsopercularis_2' 'fronto-parietal' 0.37072

'rostralmiddlefrontal_1' 'fronto-parietal' 0.55

'rostralmiddlefrontal_2' 'fronto-parietal' 0.76931

'rostralmiddlefrontal_3' 'fronto-parietal' 0.72766

'rostralmiddlefrontal_4' 'fronto-parietal' 0.83796

'rostralmiddlefrontal_5' 'fronto-parietal' 0.45588

'rostralmiddlefrontal_6' 'fronto-parietal' 0.70379

'superiorfrontal_1' 'DMN' 0.8356

'superiorfrontal_2' 'DMN' 0.76767

'superiorfrontal_3' 'DMN' 0.46788

'superiorfrontal_4' 'DMN' 0.82695

'superiorfrontal_5' 'ventral-attention' 0.42059

'superiorfrontal_6' 'ventral-attention' 0.38519

'superiorfrontal_7' 'ventral-attention' 0.36127

'superiorfrontal_8' 'fronto-parietal' 0.47197

'caudalmiddlefrontal_1' 'fronto-parietal' 0.54766

'caudalmiddlefrontal_2' 'fronto-parietal' 0.78462

'caudalmiddlefrontal_3' 'fronto-parietal' 0.81452

'precentral_1' 'ventral-attention' 0.72683

'precentral_2' 'dorsal-attention' 0.53882

'precentral_3' 'somato-motor' 0.34834

'precentral_4' 'somato-motor' 0.28525

'precentral_5' 'somato-motor' 0.56938

'precentral_6' 'somato-motor' 0.67253

'paracentral_1' 'somato-motor' 0.80481

'paracentral_2' 'somato-motor' 0.80702

'paracentral_3' 'ventral-attention' 0.38062

'rostralanteriorcingulate_1' 'DMN' 0.65539

'caudalanteriorcingulate_1' 'ventral-attention' 0.46863

'posteriorcingulate_1' 'DMN' 0.46719

'posteriorcingulate_2' 'DMN' 0.34653

'isthmuscingulate_1' 'DMN' 0.51145

'postcentral_1' 'somato-motor' 0.78365

'postcentral_2' 'somato-motor' 0.81935

'postcentral_3' 'somato-motor' 0.79633

'postcentral_4' 'somato-motor' 0.55972

'postcentral_5' 'somato-motor' 0.86533

'supramarginal_1 ' 'dorsal-attention' 0.54517

'supramarginal_2 ' 'fronto-parietal' 0.43145

'supramarginal_3' 'ventral-attention' 0.65873

'supramarginal_4 ' 'somato-motor' 0.43985

'superiorparietal_1' 'dorsal-attention' 0.55401

'superiorparietal_2' 'dorsal-attention' 0.49026

'superiorparietal_3' 'dorsal-attention' 0.92234

'superiorparietal_4' 'dorsal-attention' 0.86084

'superiorparietal_5' 'dorsal-attention' 0.56375

'superiorparietal_6' 'visual' 0.66436

'superiorparietal_7' 'visual' 0.92857

'inferiorparietal_1' 'fronto-parietal' 0.61286

'inferiorparietal_2' 'DMN' 0.52863

'inferiorparietal_3' 'DMN' 0.56674

'inferiorparietal_4' 'DMN' 0.66866

'inferiorparietal_5' 'DMN' 0.65974

'inferiorparietal_6' 'dorsal-attention' 0.43216

'precuneus_1' 'visual' 0.71591

'precuneus_2' 'DMN' 0.66585

'precuneus_3' 'DMN' 0.66604

'precuneus_4' 'fronto-parietal' 0.31812

'precuneus_5' 'ventral-attention' 0.32244

'cuneus_1' 'visual' 0.93082

'cuneus_2' 'visual' 0.94896

'pericalcarine_1' 'visual' 0.73131

'pericalcarine_2' 'visual' 0.76104

'lateraloccipital_1' 'visual' 0.95871

'lateraloccipital_2' 'visual' 0.90205

'lateraloccipital_3' 'visual' 0.84601

'lateraloccipital_4' 'visual' 0.87977

'lateraloccipital_5' 'visual' 0.79323

'lingual_1' 'visual' 0.86017

'lingual_2' 'visual' 0.87273

'lingual_3' 'visual' 0.85831

'fusiform_1' 'visual' 0.85464

'fusiform_2' 'visual' 0.76199

'fusiform_3' 'visual' 0.69094

'fusiform_4' 'limbic' 0.81526

'parahippocampal_1' 'MTL' 0.50997

'entorhinal_1' 'MTL' 0.84566

'temporalpole_1' 'limbic' 0.80895

'inferiortemporal_1' 'limbic' 0.86963

'inferiortemporal_2' 'limbic' 0.55819

'inferiortemporal_3' 'fronto-parietal' 0.4436

'inferiortemporal_4' 'dorsal-attention' 0.75995

'middletemporal_1' 'fronto-parietal' 0.28681

'middletemporal_2' 'DMN' 0.75764

'middletemporal_3' 'DMN' 0.77883

'middletemporal_4' 'DMN' 0.79026

'bankssts_1' 'somato-motor' 0.37542

'superiortemporal_1' 'somato-motor' 0.81915

'superiortemporal_2' 'somato-motor' 0.72192

'superiortemporal_3' 'somato-motor' 0.71457

'superiortemporal_4' 'ventral-attention' 0.31504

'superiortemporal_5' 'DMN' 0.35759

'transversetemporal_1' 'somato-motor' 0.8254

'insula_1' 'somato-motor' 0.52381

'insula_2' 'ventral-attention' 0.60194

'insula_3' 'ventral-attention' 0.54486

'thalamusproper' 'Subcortical' 0

'caudate' 'Subcortical' 0

'putamen' 'Subcortical' 0

'pallidum' 'Subcortical' 0

'accumbensarea' 'Subcortical' 0.0060976

'hippocampus' 'MTL' 0.0032468

'amygdala' 'MTL' 0.090909

Left hemisphere

'lateralorbitofrontal_1' 'limbic' 0.75983

'lateralorbitofrontal_2' 'DMN' 0.45229

'lateralorbitofrontal_3' 'limbic' 0.42199

'lateralorbitofrontal_4' 'limbic' 0.75833

'parsorbitalis_1' 'DMN' 0.72103

'frontalpole_1' 'limbic' 0.48529

'medialorbitofrontal_1' 'DMN' 0.63043

'medialorbitofrontal_2' 'limbic' 0.89888

'parstriangularis_1' 'DMN' 0.41515

'parsopercularis_1' 'fronto-parietal' 0.53061

'parsopercularis_2' 'fronto-parietal' 0.44505

'rostralmiddlefrontal_1' 'fronto-parietal' 0.81356

'rostralmiddlefrontal_2' 'ventral-attention' 0.33806

'rostralmiddlefrontal_3' 'fronto-parietal' 0.54008

'rostralmiddlefrontal_4' 'fronto-parietal' 0.42274

'rostralmiddlefrontal_5' 'fronto-parietal' 0.47438

'rostralmiddlefrontal_6' 'DMN' 0.56226

'superiorfrontal_1' 'DMN' 0.86557

'superiorfrontal_2' 'DMN' 0.86979

'superiorfrontal_3' 'DMN' 0.82006

'superiorfrontal_4' 'fronto-parietal' 0.34429

'superiorfrontal_5' 'DMN' 0.52475

'superiorfrontal_6' 'DMN' 0.44301

'superiorfrontal_7' 'ventral-attention' 0.34985

'superiorfrontal_8' 'somato-motor' 0.34337

'superiorfrontal_9' 'dorsal-attention' 0.51885

'caudalmiddlefrontal_1' 'DMN' 0.81543

'caudalmiddlefrontal_2' 'fronto-parietal' 0.44991

'caudalmiddlefrontal_3' 'dorsal-attention' 0.33793

'precentral_1' 'somato-motor' 0.50392

'precentral_2' 'somato-motor' 0.57556

'precentral_3' 'somato-motor' 0.73697

'precentral_4' 'somato-motor' 0.4

'precentral_5' 'dorsal-attention' 0.41471

'precentral_6' 'somato-motor' 0.41414

'precentral_7' 'dorsal-attention' 0.48762

'precentral_8' 'ventral-attention' 0.81122

'paracentral_1' 'somato-motor' 0.85484

'paracentral_2' 'somato-motor' 0.67148

'rostralanteriorcingulate_1' 'DMN' 0.71516

'caudalanteriorcingulate_1' 'ventral-attention' 0.53613

'posteriorcingulate_1' 'DMN' 0.29024

'posteriorcingulate_2' 'DMN' 0.38583

'isthmuscingulate_1' 'DMN' 0.60311

'postcentral_1' 'somato-motor' 0.94643

'postcentral_2' 'somato-motor' 0.77835

'postcentral_3' 'somato-motor' 0.64937

'postcentral_4' 'somato-motor' 0.92424

'postcentral_5' 'somato-motor' 0.90062

'postcentral_6' 'somato-motor' 0.76817

'postcentral_7' 'somato-motor' 0.94545

'supramarginal_1' 'somato-motor' 0.45996

'supramarginal_2 ' 'ventral-attention' 0.68496

'supramarginal_3' 'ventral-attention' 0.58601

'supramarginal_4' ' fronto-parietal' 0.40164

'supramarginal_5' ' dorsal-attention' 0.75956

'superiorparietal_1' 'dorsal-attention' 0.46787

'superiorparietal_2' 'dorsal-attention' 0.5107

'superiorparietal_3' 'dorsal-attention' 0.45514

'superiorparietal_4' 'dorsal-attention' 0.86099

'superiorparietal_5' 'dorsal-attention' 0.58915

'superiorparietal_6' 'visual' 0.45721

'superiorparietal_7' 'visual' 0.91361

'inferiorparietal_1' 'visual' 0.45255

'inferiorparietal_2' 'DMN' 0.70989

'inferiorparietal_3' 'DMN' 0.67202

'inferiorparietal_4' 'fronto-parietal' 0.42336

'inferiorparietal_5' 'fronto-parietal' 0.52444

'precuneus_1' 'dorsal-attention' 0.33333

'precuneus_2' 'DMN' 0.76399

'precuneus_3' 'DMN' 0.65756

'precuneus_4' 'DMN' 0.78586

'precuneus_5' 'visual' 0.52155

'cuneus_1' 'visual' 0.961

'pericalcarine_1' 'visual' 0.80824

'lateraloccipital_1' 'visual' 0.93241

'lateraloccipital_2' 'visual' 0.94618

'lateraloccipital_3' 'visual' 0.78082

'lateraloccipital_4' 'visual' 0.72427

'lateraloccipital_5' 'visual' 0.82411

'lingual_1' 'visual' 0.87843

'lingual_2' 'visual' 0.91293

'lingual_3' 'visual' 0.83651

'lingual_4' 'visual' 0.85902

'fusiform_1' 'visual' 0.88426

'fusiform_2' 'visual' 0.5507

'fusiform_3' 'visual' 0.76065

'fusiform_4' 'limbic' 0.7565

'parahippocampal_1' 'MTL' 0.41823

'entorhinal_1' 'MTL' 0.86853

'temporalpole_1' 'limbic' 0.86029

'inferiortemporal_1' 'limbic' 0.71596

'inferiortemporal_2' 'limbic' 0.41524

'inferiortemporal_3' 'dorsal-attention' 0.4034

'inferiortemporal_4' 'dorsal-attention' 0.72065

'middletemporal_1' 'ventral-attention' 0.3234

'middletemporal_2' 'DMN' 0.91494

'middletemporal_3' 'DMN' 0.88049

'middletemporal_4' 'DMN' 0.79903

'bankssts_1' 'DMN' 0.52415

'bankssts_2' 'DMN' 0.51923

'superiortemporal_1' 'somato-motor' 0.61648

'superiortemporal_2' 'somato-motor' 0.70981

'superiortemporal_3' 'somato-motor' 0.46429

'superiortemporal_4' 'somato-motor' 0.36767

'superiortemporal_5' 'limbic' 0.52941

'transversetemporal_1' 'somato-motor' 0.82886

'insula_1' 'somato-motor' 0.35356

'insula_2' 'ventral-attention' 0.60215

'insula_3' 'ventral-attention' 0.48525

'insula_4' 'ventral-attention' 0.3876

'thalamus proper' 'Subcortical' 0.00057013

'caudate' ' Subcortical' 0

'putamen' 'Subcortical' 0

'pallidum' 'Subcortical' 0

'accumbens area' 'Subcortical' 0

'hippocampus' 'MTL' 0.0075676

'amygdala' 'MTL' 0.081851

'brainstem' 'Subcortical' 0
