# Supplementary material for: Structural optimality and neurogenetic expression mediate functional dynamics in the human brain
Source: Hum Brain Mapp. 2020 Feb 6;41(8):2229–43. doi: 10.1002/hbm.24942 (PMC7267953; doi:10.1002/hbm.24942)
Supplement: Supplementary file 1 — Appendix S1: Supplementary Information [file HBM-41-2229-s001.zip › HBM_24942_Supplementary_IP_proofread.docx]

Supplementary Information for: **Structural optimality and neurogenetic expression mediate functional dynamics in the human brain**

**Authors:** I. Pappas^1, 2, *,^†, M.M. Craig^1, 2, *,^†, D.K. Menon^1, 2^, E.A. Stamatakis^1, 2^

† These authors contributed equally to this work.

Contents

1. Supplementary Methods…………………………………………………..

2. Supplementary Figures…………………………………………………..

3. Supplementary Tables…………………………………………………..

4. Supplementary References…………………………………………………..

5. Data and code availability…………………………………………………..

6. Author contributions…………………………………………………..

1. Supplementary Methods

1.1 Data

Data were provided [in part] by the Human Connectome Project, WU-Minn Consortium (Principal Investigators: David Van Essen and Kamil Uğurbil; 1U54MH091657) funded by the 16 NIH Institutes and Centers that support the NIH Blueprint for Neuroscience Research; and by the McDonnell Center for Systems Neuroscience at Washington University. Data were downloaded from the Human Connectome Project (HCP) website: [*http://www.humanconnectome.org/*](http://www.humanconnectome.org/). Structural and functional magnetic resonance images from a total of 50 individuals were used in the analysis. Subjects were between the ages of 22 and 35 (**Supplementary File 3a**). Our methods are a combination of structural connectivity, functional connectivity, and gene expression in the human brain. Therefore to determine our sample size we focused on the reproducibility of the respective measures used (i.e. Fractional Anisotropy (FA), BOLD correlations, and gene expression). Specifically, previous work on the reproducibility of FA has shown that a sample size of 50 is sufficient to provide a power of 0.9 (De Santis, Drakesmith, Bells, Assaf, & Jones, 2014). Secondly, results on 50 simulated subjects showed that BOLD correlations are sensitive to capturing inter-subject connectivity strength variability (Smith et al., 2011). Finally, gene expression data comes from a unique dataset of 2 post-mortem brains from the Allen Institute for Brain Science and previous work has shown this data consists a transcriptional signature that is robust across individuals with ongoing work focusing on processing additional brains for consistency.

1.2 Whole-brain connectivity

*Structural connectivity*

Real structural connections were defined using High Angular Resolution Diffusion Imaging (HARDI). Using HARDI data is advantageous for identifying complex axonal pathways due to the fact the diffusion-weighted signal is acquired in more directions that usual Diffusion Tensor Imaging (DTI) sequences, thus providing increased accuracy in determining precise axonal morphology (Tuch, 2004). Data were acquired using a single shot single refocusing spin-echo, echo-planar imaging sequence with a multishell diffusion scheme of 90 diffusion weighted directions with b-values of 990, 1985, and 2985 $s/mm^{2}$ and spatial resolution of 1.25 x 1.25 x 1.25 $mm^{3}$. Preprocessing was conducted as part of the HCP pipeline and included eddy current and motion corrections, gradient non-linearity correction, and transformation to native structural space. The diffusion tensors were reconstructed in DSI-studio ([*http://dsi-studio.labsolver.org*](http://dsi-studio.labsolver.org)) using generalized q-sampling imaging (GQI). GQI is a model-free reconstruction procedure that quantifies the density of diffusion in different orientations, thus providing directional information regarding crossing fibres. DSI-studio uses the spin distribution function (SDF) that has greater sensitivity and specificity to white matter characteristics (Yeh, Van Jay, & Wen-Yih, 2010). The diffusion-sampling threshold for our data was set to 1.25 (Kuo, Chen, Wedeen, & Tseng, 2008). A deterministic fibre-tracking algorithm was used to obtain Generalized Fractional Anisotropy (GFA) values between brain regions (Cheng et al., 2015). This method uses spherical harmonics and Orientation Distribution Functions (ODF) to accurately characterize estimates of anisotropy. The anisotropy ratio threshold was 0.0518329, the angular threshold was set to 60 degrees, and the step size was 0.625 mm (half of the voxel size in one dimension).

T1-weighted data was also acquired with TR = 2400ms, TE =2.14ms, flip angle = 8 degrees, field of view 224 x 224mm, voxel size 0.7mm isotropic, bandwidth 219 Hz/pixel and ipat factor of 2. The T1-weighted data were segmented using *FreeSurfer*’s *recon-all* function (Fischl, Sereno, Tootell, & Dale, 1999). These were then parcellated using the Lausanne parcellation (Hagmann et al., 2008, [*http://github.com/mattcieslak/easy_lausanne*](http://github.com/mattcieslak/easy_lausanne)), at two different resolutions, 234 and 129 regions of interest (ROIs). Using the parcellation and the diffusion data we created a 234 x 234 or 129 x 129 connectivity matrix depending on the resolution of the parcellation used. Each entry $(i,j)$ contained either a positive number corresponding to the GFA when a non-zero GFA between each pair of brain regions was obtained or a 0 when a zero GFA value was obtained. The deterministic fibre-tracking algorithm also provided the number of streamlines between each pair of regions. In order to see our results were reproducible when streamlines were taken into consideration, separate structural connectivity matrices were constructed with each entry $(i,j)$ corresponding to the number of white matter streamlines connecting them (Gong, He, Concha, Lebel, Gross, Evans, & Beaulieu, 2009; Cohen-Adad, Descoteaux, Rossignol, Hog, & Deriche, 2008).

*From ROIs to large-scale networks*

To construct the networks of interest, we used the 7 masks from Yeo et al. (Yeo et al., 2011) In order to assign each ROI to one of the networks we calculated each ROI’s overlap with each network mask. The maximum overlap served as the criterion for assigning each ROI to a network. This assignment and the overlap numbers are included in the **Supplementary File 3b.** The number of connections within and between the different networks was defined using the real structural matrices from the HARDI data. These numbers were used as the denominator for inter- and intranetwork optimal connections (nominator) ratios used in **Fig. 2** of the main text in order to account for the different number of edges between and within networks.

*Resting-state fMRI data acquisition and functional connectivity*

Whole-brain echo-planar imaging (EPI) data was acquired with a 32-channel head coil using a 3T Siemens (Erlangen, Germany) Skyra scanner, modified for use in the HCP. The acquisition parameters were as follows: repetition time TR = 720ms, echo time TE = 33.1ms, flip angle = 52 degrees, bandwidth = 2290 Hz/pixel, in plane field of view = 208 x 180mm, slice number = 72, 2mm isotropic voxels and multiband acceleration factor = 8 (Smith et al., 2013). The first 13 volumes (corresponding to 10 seconds) were removed to eliminate saturation effects and achieve steady state magnetization. The HCP minimal preprocessing pipeline was used to preprocess functional data. This included motion correction, registration to structural T1-weighted scan, and non-linear registration into MNI152 space. Connectivity analysis was performed using the Conn functional connectivity toolbox (Whitfield-Gabrieli & Nieto-Castanon, 2012). Conn uses a strict preprocesing pipeline in order to deal with sources of noise in the data. First despiking was used for dealing with outlier data. Then physiological noise was regressed out using the anatomical *CompCor* (aCompCor) technique, which removes the top 5 principal components of the signal attributed to white matter and cerebrospinal fluid masks (Behzadi, Restom, Liau, & Liu, 2012). The motion parameters and their first-order temporal derivatives were also regressed out. Linear detrending was also used to remove linear drifts. Finally functional images were highpass filtered at 0.009 Hz to remove low frequency drifts due to scanner noise. Following preprocessing we computed Pearson’s correlations between each region in the Lausanne parcellation, resulting in a $234 x 234$ or 1$29 x 129$ functional connectivity matrices for each individual.

*Tasked-based fMRI data acquisition and functional connectivity*

Tasked-based fMRI was obtained using similar acquisition parameters to the resting-state data (Barch et al., 2013). In short, whole-brain EPI data was acquired with a 32-channel head coil using a 3T Siemens Skyra scanner, modified for use in the HCP. The acquisition parameters were as follows: TR = 720ms, TE = 33.1ms, flip angle = 52 degrees, bandwidth = 2290Hz/pixel, in-plane field of view = 208 x 180mm, slice number = 72, 2mm isotropic voxels and multiband acceleration factor = 8. A version of the n-back task was chosen in order to assess working memory. Two runs of each task were acquired, one with a right-to-left and the other with a left-to-right phase encoding. Details of the task are presented in Barch et al. (Barch et al., 2013). The resulting data obtained from the following process was averaged across the two runs.

Blocks of trials included the presentation of four types of stimulus (faces, places, tools and body parts) were presented). Within each run half of the trials were used a 2-back (2bk) working memory task and half were used for 0-back (0bk) task. Each run contained 8 blocks of trials each of them consisting of 10 trials (lasting 2.5 second each-total 25 seconds) and 4 fixation blocks (15 seconds each). On each trial, the stimulus was presented for 2s, followed by a 500ms inter-trial interval. Out of the 10 trials each block contained, 2 of them were targets, and 2-3 were non-target lures (e.g., repeated items in the wrong n-back position, either 1-back or 3-back). BOLD data for each region of interest and each task condition was obtained by using the Conn functional connectivity toolbox (Whitfield-Gabrieli & Nieto-Castanon, 2012). Preprocessing was similar to the resting-state data with the exception that we added in the confounding regressors the main effect of the task (Fair et al., 2007). To obtain task-specific functional connectivity data, the BOLD time series were first divided into block-specific scans as these come from the onsets and duration of each task. The delay in hemodynamic response was accounted for by convolving the block regressors for each task condition with a rectified hemodynamic response function. For each task condition, the scans associated with nonzero effects in the resulting time series were concatenated and weighted by the value of the corresponding time series (Vatansever, Menon, Manktelow, Sahakian, & Stamatakis, 2015). In turn, undirected and weighted functional connectivity matrices (only for the 234-resolution parcellation here) were constructed. For this work we restricted our analysis only to the data corresponding to 2bk face task.

1.2 Nash Equilibrium Network Game model (NNG)

The NNG model is the Nash equilibrium of a non-cooperative game played on networks where each player or node follows a strategy independently of others in order to maximize a certain payoff (Nisan, 2007). In the context of this work, each node’s optimal navigation vector/strategy relates to two distinct objectives: to maximize navigability with minimum number of edges.

We will provide formulas for deriving each node’s optimal navigation vector using graph-theoretic terms following closely the notation used by Gulyás et al. (Gulyás, Bíró, Kőrösi, Rétvári, & Krioukov, 2016). A triplet of coordinates $x,y,z$ represents the position in the Euclidean space of the nodes of interest $V$ (e.g. the ROI coordinates of the 234 or 129 parcellation resolutions). Distance between two nodes $p_{1}$ and $p_{2}$ with coordinates $x_{p1}, y_{p1}, z_{p1}$ and $x_{p2}, y_{p2}, z_{p2}$ respectively was defined as the Euclidean distance and was calculated as

$$dist\left( p_{1},p_{2} \right)=\sqrt{\left( x_{p_{1}}-x_{p_{2}} \right)^{2}+\left( y_{p_{1}}-y_{p_{2}} \right)^{2}+\left( z_{p_{1}}-z_{p_{2}} \right)^{2}}$$

For each pair of nodes $u\in V$ and$v\in V$, let$S_{v}^{u}=\left\{ w \right|dist\left( v,w \right)<dist(u,w)\}$. This represents the set of nodes that information can be navigated to from $u$, using $v$ as a first step.

The problem with finding an optimal navigation vector for a node $u\in V$ is formulated as follows. First, node $u\in V$ is associated with a collection of sets $S_{v}^{u}$, each one corresponding to the rest of the nodes $v\in V \backslash\{u\}$. In turn, the optimal navigation vector of node $u$ consists of constructing edges to those nodes $v’$ such that their $S_{v'}^{u}$ sets belong to the minimum cover set of the sets $S_{v}^{u}$. For a collection of sets, the minimum cover set problem refers to selecting the minimum number of sets such that their union includes all the elements appearing in the collection of sets. Formally, for each $v\in V \backslash\{u\}$ the problem is to find the binary values of the decision variables $d_{v}$ indicating whether the $S_{v}^{u}$ is going to be selected or not as part of the minimum cover set. To assist with the problem formulation we assign a variable $a_{v^{'}v}=1$ based on whether a node $v'$ belongs to a set $S_{v}^{u}$. The minimum cover set problem for node $u$ can then be written as the following optimization problem.

$$\min\sum d_{v}$$

$$subject to \sum a_{v^{'}v}d_{v}\geq1 v,v'\in V \backslash\{u\}$$

$$d_{v}=\{0,1\}$$

Instead of solving the integer form of the minimum cover set problem, we considered its linear relaxation by allowing the decision variables to be real non-negative numbers rather than integers, i.e., $d_{v}\geq0$. This relaxation transforms a hard linear integer programming problem into a related problem that can be solved in polynomial time where the solution of the latter is at least as good as the original problem (Schrijver, 1987).

The previous procedure results in a number of connections for node $u$ representing its optimal navigation vector. If this procedure is repeated independently for all nodes $u\in V$, it can be proved that the resulting network is the Nash Equilibrium of all the nodes’ optimal navigation vectors and is characterized by maximum navigability with minimum number of edges (Gulyás, Bíró, Kőrösi, Rétvári, & Krioukov, 2016). To obtain NNG models for each individual’s T1 parcellation we used the following methodology. For each individual’s ROIs’ coordinates we calculated the minimum cover set for each ROI $u$ by formulating and solving the corresponding problem using the *glpk* library ([*https://www.gnu.org/software/glpk*](https://www.gnu.org/software/glpk)). Edges between the ROI $u$ and the rest of the ROIs $v\in V \backslash\{u\}$ were constructed when the software’s output for $d_{v}$ was 1. This was repeated for all ROIs to produce one NNG model for each individual. Optimality results were obtained by comparing the NNG edges with the edges in the real structural matrix coming from the HARDI data and described in the *Structural Connectivity* section.

1.3 Using geodesic distance for calculating optimality

Optimality was also assessed using geodesic distances instead of Euclidean distances. To obtain the geodesic distances we used the publicly available package [*https://github.com/NeuroanatomyAndConnectivity/surfdist*](https://github.com/NeuroanatomyAndConnectivity/surfdist) on the *Freesurfer* output of each individual (Margulies, Falkiewicz, & Huntenburg, 2016). We used the function *dist_calc_matrix* to calculate the geodesic distance between each region of interest. Because geodesic distances are defined only within closed surfaces, results were obtained for each hemisphere separately. Thus we assessed optimality for the two hemispheres separately by taking the ratio of the true positive edges over the number of the NNG edges produced within each hemisphere.

1.4 Predicting functional from structural connectivity

*Formulation of the problem*

For each pair of functional and structural connectivity matrices we used a transformation as described in Becker et al. (Becker et al., 2018) Suppose that for an individual subject $j$ we obtained structural and functional connectivity matrices of dimensions$n x n$: $\boldsymbol{S}_{j}$ and $\boldsymbol{F}_{j}$ respectively. The first step consists of writing the predicted functional connectivity matrix as

$${\overbrace{\boldsymbol{F}}}_{j}\boldsymbol{= R}\left( \sum_{r=0}^{k} a_{r}\boldsymbol{S}_{j}^{r} \right) \boldsymbol{R}^{T}$$

The term $\sum_{r=0}^{k} {(a}_{r}\boldsymbol{S}_{j}^{r})$ represented a weighted sum of powers of $\boldsymbol{S}_{j}$ up to order $k$ (polynomial transformation of order$k$). Spectral graph theory states that powers of $k$ of the structural connectivity matrix are related to the paths (more precisely walks) of the graph (Chung, 1997). These are traversals from one vertex of the graph to another (with a potential repetition of the same vertices) using the edges of the graph. Therefore higher order transformations show how information can be communicated upon long walks in the graph-something that is potentially important for predicting functional connectivity. After the polynomial transformation, the rotation matrix $\boldsymbol{R}$ is used to transform the eigenvectors of the matrix$\boldsymbol{S}_{j}$ in order to align to those of $\boldsymbol{F}_{j}$. In the second step we solved the optimization problem that finds the best approximation ${\overbrace{\boldsymbol{F}}}_{j}$ to the real matrix $\boldsymbol{F}_{j}$, i.e., we solved the problem

$$\min_{\left\{ a_{r} \right\}_{r=0}^{k},\boldsymbol{R}} \left\| {\overbrace{\boldsymbol{F}}}_{j}-\boldsymbol{F}_{j} \right\|=\left\| \boldsymbol{R}\left( \sum_{r=0}^{k} a_{r}\boldsymbol{S}_{j}^{r} \right) \boldsymbol{R}^{T}-\boldsymbol{F}_{j} \right\| , \boldsymbol{R}^{T}\boldsymbol{R=R}\boldsymbol{R}^{T}\boldsymbol{=}\boldsymbol{I}_{\boldsymbol{n}},\det\boldsymbol{R}=1 (P)$$

where $\left\| . \right\|$ stands for the Frobenius norm and $\boldsymbol{I}_{\boldsymbol{n}}$ is the all-ones diagonal matrix of dimension $n$. The constrain guarantees that the matrix $\boldsymbol{R}$ is a rotation matrix. Prior to solving the problem we provide some notation. We write$\boldsymbol{v}_{i=1}^{n}$, $\lambda_{i=1}^{n}$ and$\boldsymbol{u}_{i=1}^{n}$, $\phi_{i=1}^{n}$ for the eigenvectors and eigenvalues of $\boldsymbol{S}_{j}$ and $\boldsymbol{F}_{j}$ respectively. Consider the vectors$\boldsymbol{\phi}=\left[ \phi_{1},\ldots, \phi_{n} \right]^{T}$, $\boldsymbol{\lambda}=\left[ \lambda_{1},\ldots, \lambda_{n} \right]^{T}$ and the matrices $\boldsymbol{V}=[v_{1}|\ldots|v_{n}]$ and $\boldsymbol{U}=[u_{1}|\ldots|u_{n}]$ for $\boldsymbol{S}$ and $\boldsymbol{F}$ respectively. We also consider the Vandermonde matrix

$$\boldsymbol{L}= \left[ \begin{matrix} 1 & \lambda_{1} & \cdots& \lambda_{1}^{k} \\ 1 & \lambda_{2} & \cdots& \lambda_{2}^{k} \\ \vdots& \vdots& \ddots& \vdots\\ 1 & \lambda_{n} & \cdots& \lambda_{n}^{k} \end{matrix} \right]$$

where $k$ is the order of the polynomial transformation. The minimization problem (P) can be decomposed into two problems that we solve in their generic form. The first problem is that of finding the optimal coefficients to fit the polynomial transformation of order $k$ to the matrix$\boldsymbol{S}$. Generally, for a matrix $\boldsymbol{g}$ and coefficient matrix $\boldsymbol{A}$, this problem can be written as

$\min_{\boldsymbol{x}}\left\| \boldsymbol{Ax}-\boldsymbol{g} \right\|$

where $A_{ij}=t_{i}^{j-1}$ can be written as the Vandermonde matrix

$$\left[ \begin{matrix} 1 & t_{1} & \cdots& t_{1}^{k} \\ 1 & t_{2} & \cdots& t_{2}^{k} \\ \vdots& \vdots& \ddots& \vdots\\ 1 & t_{n} & \cdots& t_{n}^{k} \end{matrix} \right]$$

The solution to this problem uses optimization techniques (Schönemann, 1968) and is given by

$$\boldsymbol{x}^{*}=\left( \boldsymbol{A}^{T}\boldsymbol{A} \right)^{-1}\boldsymbol{A}^{T}\boldsymbol{g}$$

The second problem rotates the eigenvectors of the weighted sum of powers of $\boldsymbol{S}_{j}$to fit the eigenvectors of $\boldsymbol{F}_{j}$. In general, for matrices $\boldsymbol{A}$ and $\boldsymbol{B}$ the problem pertains to finding a rotation matrix $\boldsymbol{\Omega}$ as follows.

$$\min_{\boldsymbol{\Omega}}\left\| \boldsymbol{\Omega}\boldsymbol{A}\boldsymbol{\Omega}^{T}-\boldsymbol{B} \right\|$$

$$\boldsymbol{\Omega}^{T}\boldsymbol{\Omega=\Omega}\boldsymbol{\Omega}^{T}=\boldsymbol{I}_{n},\det\boldsymbol{\Omega}=1$$

For real symmetric matrices this problem can be solved using the eigenvalue decomposition of the matrix $\boldsymbol{A}= \boldsymbol{U}_{A}\boldsymbol{\Sigma}_{A}{\boldsymbol{U}_{A}}^{-1}$ and the eigenvalue decomposition of $\boldsymbol{B}= \boldsymbol{U}_{B}\boldsymbol{\Sigma}_{B}{\boldsymbol{U}_{B}}^{-1}$ with the solution being the matrix $\boldsymbol{\Omega}^{*}=\boldsymbol{U}_{A}\boldsymbol{U}_{B}^{T}$ (Boyd & Vandenberghe, 2007).

Using these formulas for the prediction problem $(P)$ we obtained the pair of solutions

$$\left( a_{0}^{*}, \ldots, a_{k}^{*} \right)^{T}=\left( \boldsymbol{L}^{T}\boldsymbol{L} \right)^{-1}\boldsymbol{L}^{T}\phi, \boldsymbol{R}^{*}=\boldsymbol{U}\boldsymbol{V}^{T}$$

and thus ${\overbrace{\boldsymbol{F}}}_{j}$ is written as

$${\overbrace{\boldsymbol{F}}}_{j}= \boldsymbol{R}^{*}\left( \sum_{r=0}^{k} a_{r}^{*}\boldsymbol{S}_{\boldsymbol{j}}^{r} \right){\boldsymbol{(R}^{*})}^{T}$$

The algorithm for predicting ${\overbrace{\boldsymbol{F}}}_{j}$ was used in three scenarios that differed according to their structural connectivity matrix input 1) using the whole-brain structural connectivity matrix $\boldsymbol{S}_{j}$2) using the (sub) network of $\boldsymbol{S}_{j}$ consisting only of optimal connections 3) using the (sub) network of $\boldsymbol{S}_{j}$consisting only of non-optimal connections. To assess the importance of structural connections in each scenario, we used two different goodness-of-fit measures between the predicted ${\overbrace{\boldsymbol{F}}}_{j}$ and the real $\boldsymbol{F}_{j}$ functional connectivity matrices as described in the following section.

*Evaluation of prediction*

We quantified the goodness-of-fit between the predicted ${\overbrace{\boldsymbol{F}}}_{j}$ and the real $\boldsymbol{F}_{j}$functional connectivity matrices by employing two methods (Figure 3-Supplement 1):

1. We used correlation of the upper triangular entries obtained from the real matrix with those entries obtained from the predicted matrix. The three scores, corresponding to the three previously mentioned scenarios are depicted in Figure 3-Supplement 2 for different values of $k$ and for the two parcellation resolutions. We observed that predicting using optimal edges resulted in higher correlation between the predicted functional connectivity matrix and the real functional connectivity matrix compared to using all the structural connections or non-optimal connections.
2. We also used the proposed homology-based evaluation. This evaluation is based on comparing the number of connected components of the predicted and real functional connectivity matrices at different edge density levels $\lambda$(Betti numbers $\beta_{0}(\lambda)$ and $\hat{\beta_{0}(\lambda)}$ respectively)(Liang & Wang, 2017). Here edge density refers to the percentage of correlation entries in the connectivity matrix. Therefore zero density refers to an empty matrix whereas density of 1 indicates the existence of all correlation values in the matrix. $SSE_{\beta}$ was used to evaluate the goodness-of-fit for the prediction and was formulated as

$$SSE_{\beta}=\frac{1}{n^{2}}\int_{0}^{1} {(\beta_{0}\left( \lambda\right)-\hat{\beta_{0}(\lambda))}}^{2} d\lambda$$

where the integral spans all edge densities from 0 (no correlation entries) to 1 (all correlation entries) and $n$ is the dimension of the matrix. Intuitively, the smaller the score the better the fit of the predicted matrix to the real matrix as the number of different connected components is smaller. For the three different scenarios we observed that $SSE_{\beta}$ was smaller when optimal connections were used for prediction compared to using all the structural connections or non-optimal connections, thus indicating higher similarity between the predicted and the real functional connectivity matrices.

1.5 Synthetic network comparison

We further conducted comparison between the predictive ability of the NNG and distance-based synthetic networks. We identified synthetic networks that would fit each individual’s structural connectome. We followed closely the process presented in Betzel et al., 2016 (Betzel et al., 2016).

First we started with a core of structural connections across that were common across 50 participants. For each individual we created a synthetic network by adding edges to the common core until a number equal to the number of the NNG edges was added. Each connection was formed probabilistically with probability $P(u,v) = {dist(u,v)}^{-\eta}$ where the exponent η controls the connection length: η < 0 favours short-range while η > 0 favours longer connections. Assessing the fitness of the synthetic networks involved calculating the Kolmogorov-Smirnov statistic between the synthetic network and each individual’s real structural network. Because η is a random parameter, it could be the case that synthetic network does not resemble the topological properties of the real network that is compared against thus rendering this comparison spurious. To obtain synthetic networks that are closer fits to each individual’s network, additional steps were followed:

1. An initial stage in which parameters η were selected randomly and synthetic networks and their fitness was calculated.

2. A partitioning stage, in which the entire parameter space was partitioned according to a Voronoi tessellation. Then cells were chosen with probability inversely proportional to the fitness

P(C) ∝ EC^-a^, where EC is the energy of Voronoi cell, C, and P(C) is the relative probability of sampling from within that cell). Thus cells with better fitness were more likely to be chosen. The parameter a regulates the extent to which low energy cell will be chosen from the Voronoi tessellation.

3. New parameters were selected within the cell that was chosen in step 2 and their fitness was calculated.

The last two steps were repeated twice using parameter a= 2 allowing us to choose at each turn parameters η that would result in a better synthetic network fit for each individual. After the final repetition, synthetic networks with the closest fit to the real structural networks were chosen for further analysis. These networks were fed in the prediction algorithm to produce synthetic functional connectivity matrices. Predictive accuracy was conducted using the same method as the NNG (correlation and persistent homology metrics).

1.6 Gene expression analysis

*Gene expression sample collections and processing*

The Allen Human Brain Atlas is a publicly available online resource of microarray-based gene expression profiles for a set of predefined anatomical brain regions from the Allen Institute for Brain Science (AIBS, Hawrylycz et al., 2012). The atlas is based on post-mortem tissue from 6 donors with no known history of neurological or neuropsychiatric disease, who also passed a set of serology, toxicology, and RNA quality screens. The donors were a 24-year-old African American male (H0351.2001), a 39-year-old African American male (H0351.2002), a 57-year old Caucasian male (H0351.1009), a 31-year old Caucasian male (H0351.1012), a 49-year old Hispanic female (H0351.1015) and a 55-year old Caucasian male (H0351.1016). Each brain was cut into 0.5-1.0cm thick slabs that were frozen. Slabs were sectioned to allow tissue sampling representative for all structures throughout the brain. In turn, RNA was isolated for the different samples and microarray data were produced with expression levels for about 60,000 genes with 93% of known genes represented by at least 2 probes (see technical white paper at [*http://human.brain-map.org*](http://human.brain-map.org)). Because only two donors had bi-hemispheric coverage we focused our analysis only on data coming from two post mortem brains.

*Parcellation and AIBS data matching*

We spatially matched the centroids of each region of the 234-ROI Lausanne parcellation to the coordinates of the samples from the AIBS for each of the 2 donors that had whole-brain coverage. To do so, we converted the latter to voxel space and we investigated whether these would fall into the masks corresponding to each region of the parcellation. Using this procedure we encountered cases where more than one samples corresponded to one region. In those occasions we averaged the gene expression of the samples to obtain one expression for each region. On the other hand, some regions were not matched to any samples. This was the case for 45 bilateral regions in donor 1 and 52 bilateral regions in donor 2 (some regions were common to both donors). We preferred not to interpolate missing data but rather to exclude the unique intersection of the missing regions (a total of 81 regions) from further analysis (Arnatkeviciūtė, Fulcher, & Fornito, 2019). In order to discard probes belonging to the same gene or probes that did not match gene symbols in the AIBS data we used the gene list as presented in Whitaker et al. (Whitaker et al., 2016) Expression data were standardized for each donor using *zscore* function in MATLAB (The MathWorks, Inc., Natick, MA) and averaged across all samples from all donors across both hemispheres. Eventually, we obtained a matrix 153$x 20,737$ of whole-genome gene expression data for the remaining regions of the Lausanne parcellation.

*Partial Least Squares Analysis*

We calculated the optimal minus non-optimal degree scores (regional optimality-RO) for each region. To do so, we first considered only the network comprising the optimal edges and we calculated the degree of each region using the Brain Connectivity Toolbox ([*https://sites.google.com/site/bctnet/*](https://sites.google.com/site/bctnet/)). We repeated a similar analysis for the non-optimal degree by using the network comprising all the non-optimal edges. RO scores were normalized by the total number of connections of each region (normalized RO) for each subject and averaged across 50 subjects. We then used a Partial Least Squares (PLS) analysis to explain the variance in RO using linear combinations of the gene expression scores. We used the function *plsregress* in MATLAB (The MathWorks, Inc., Natick, MA). Unlike Principal Component Analysis (PCA), where the principal components capture only the characteristics of the predictor variables, PLS extracts components that take into consideration how each predictor variable is related to the predicted variables. Specifically, PLS uses both the variances of the predictor and predicted variables, as the components are chosen in order to explain as much of their covariance as possible (Hastie, T., Tibishirani, R., & Friedman, 2011). Thus PLS allows the extraction of more meaningful components compared to PCA especially in the cases where the predictor variables consist of multiple measurements with poorly understood variance. Since PLS is not scale invariant we standardized the predictor variable (expression data) so as to have mean=0 and standard deviation=1. The resulting components were ordered in descending order with top component being the one explaining the most variance in both X and Y. Significance was assessed based on a spatial permutation test. To do so we created a null hypothesis by shuffling 1,000 times the label assignment between the gene and RO data we re-ran the PLS analysis each time obtaining the variance explained by the top component (Váša et al., 2018). The original variance of the top component was compared against these values producing a p-value representing the confidence in rejecting the null hypothesis.

*Gene ontology enrichment analysis*

We used the online tool *GOrilla* ([*http://cbl-gorilla.cs.technion.ac.il*](http://cbl-gorilla.cs.technion.ac.il)) to search for enriched GO terms. This tool takes as input a list of genes in descending order (with the ranking based on loadings of the genes corresponding to the top PLS component) and produces gene ontology terms that appear significantly in the list of genes (Eden, Navon, Steinfeld, Lipson & Yakhini, 2009; Eden, Lipson, Yogev & Yakhini, 2007). As in Whitaker et al. work (Whitaker et al., 2016) for these analyses we unchecked the "Run GOrilla in fast mode" option and used the "P-value threshold 10-4" in the advanced parameter settings in order to best approximate FDR correction with$a = 0.05$. We used the “Function” ontology. Finally we used *REViGO* ([*http://revigo.irb.hr*](http://revigo.irb.hr)) to summarize the list of significant gene ontology terms by identifying redundant terms (Supek, Bošnjak, Škunca, Šmuc, 2011). A certain number of gene symbols were not recognized by the software and were discarded. The full list of genes and their associated terms can be found in **Supplementary File 1**.

2. Supplementary Figures

**Figure 3-Supplement 1**

**
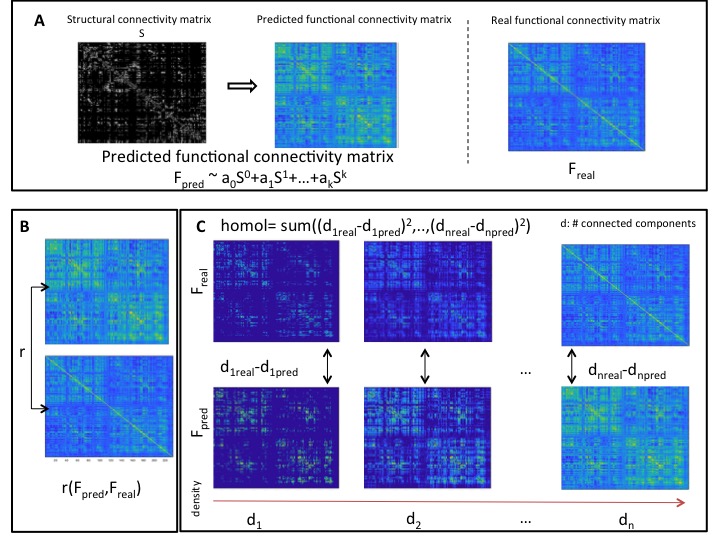
**

**Pipeline for predicting of functional connectivity from structural connectivity and evaluation of the prediction accuracy**. **(A)** First, a predicted functional connectivity matrix was constructed using a polynomial transformation of the structural connectivity matrix. Using optimization techniques the predicted functional connectivity matrix was derived to closely match the real functional connectivity matrix. In turn, we used two methods to evaluate the accuracy of the prediction. **(B)** First we used Pearson’s r correlation between the elements of the real and predicted functional connectivity matrices. Higher numbers imply higher prediction accuracy. **(C)** Second we used a homology measure that finds differences in the topology of the matrices at different levels of densities. Specifically starting from density 0 (zero elements) and going to density 1 (all entries in the matrix), at each step the difference between the number of connected components between the real and the predicted functional connectivity matrices was calculated. The sum of the squared difference represents the homology measure between the two matrices (this quantity was normalized by the square of the matrix dimension). If this measure is high then they have different number of connected components and, consequently, there is less prediction accuracy.

**Figure 3-Supplement 2**


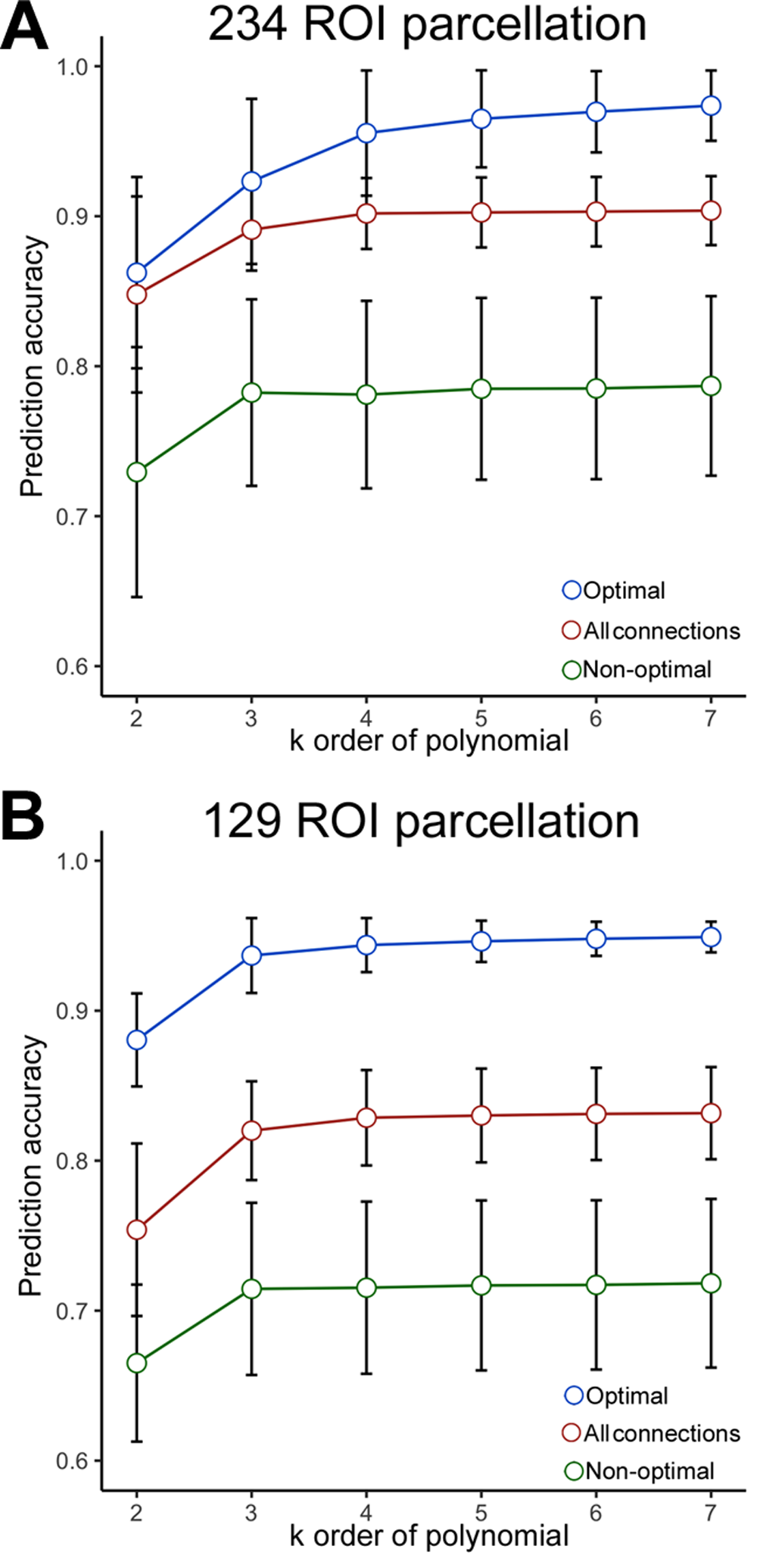


**Predicting functional connectivity from structural connectivity matrices using different orders of polynomial transformation.** The prediction algorithm uses a polynomial transformation (of order $k$) of the structural connectivity matrix to predict the functional connectivity matrix. **(A)** shows the prediction accuracy, as expressed by correlation of the predicted and real functional connectivity matrices, for the 234-ROI parcellation across different values of $k$. We observed that above $k=5$ the results plateaued showing that incorporating higher powers of polynomial transformation did not contribute further to the prediction. **(B)** shows similar results for the 129-ROI parcellation. Circles show average over n=50 individuals and lines show +- standard error of the mean.

**Figure 3-Supplement 3**


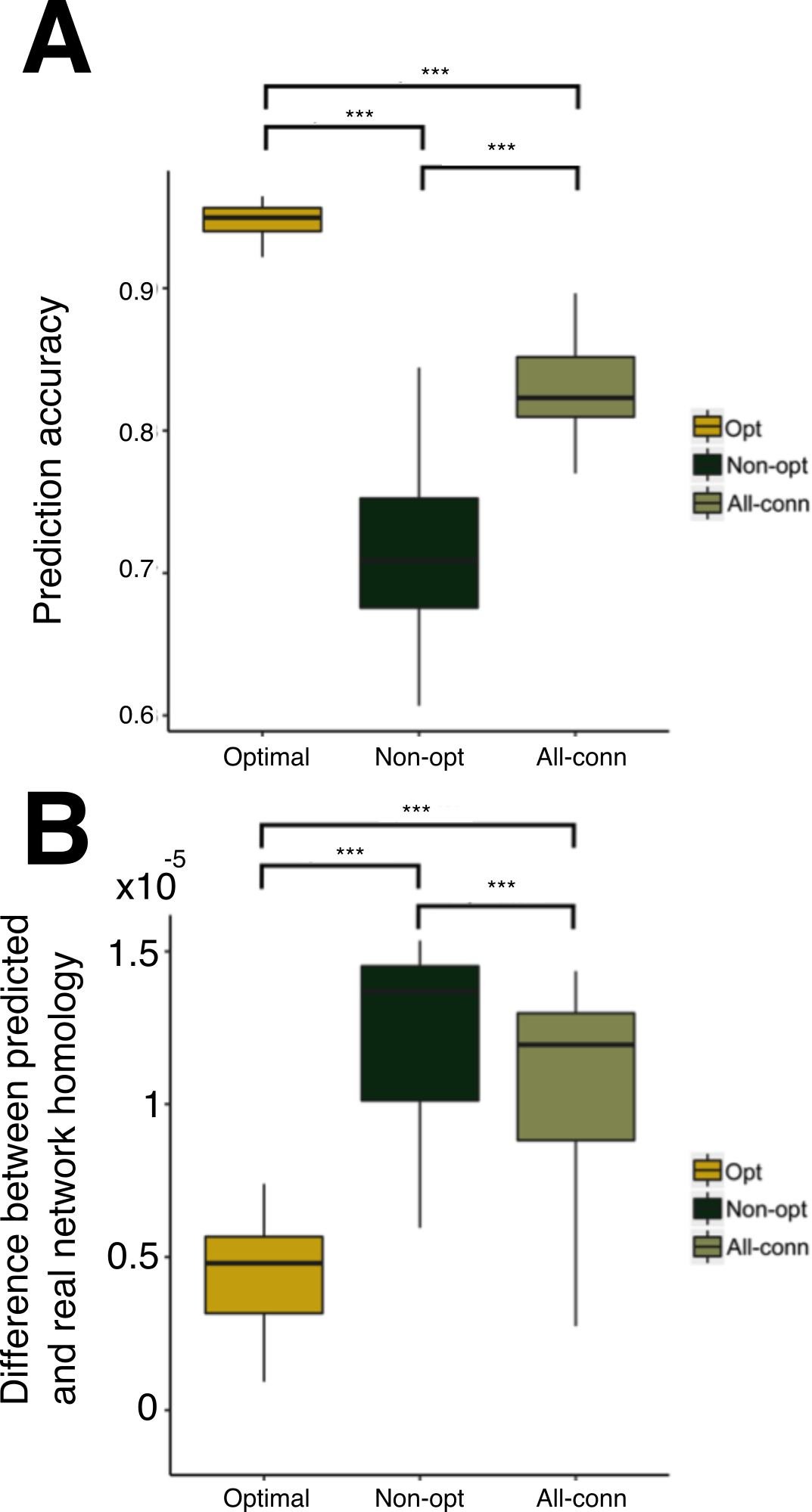


**Predicting functional connectivity from structural connectivity matrices at the 129-ROI resolution. (A)** Each box plot shows the variation of the prediction accuracy across n=50 individuals as expressed in terms of correlation between the real and predicted functional connectivity matrices (one-way repeated-measures ANOVA F(2,98) = 837, *P* < 0.0001, post-hoc tests were Bonferroni corrected for multiple comparisons). The predicted functional connectivity matrix was obtained using a polynomial order of $k =5$ of the structural connectivity matrix as in **Figure 3** of the main text. Higher scores show higher correlations between the real and the predicted functional connectivity matrices. **(B)** Here we used methods from homology theory to assess the similarities between predicted and real functional connectivity matrices (one-way repeated-measures ANOVA F(2,98) = 82.16, *P* < 0.0001, post-hoc tests were Bonferroni corrected for multiple comparisons). Lower scores reflect a smaller difference between the real and predicted functional connectivity matrices. Both prediction scores show that using only optimal connections was significantly more predictive of whole-brain functional connectivity than using all structural or non-optimal connections. For each box, thick lines show the median value for n=50 individuals while whiskers reflect the maximum and minimum values of the data. Triple asterisks (***) imply *post hoc* statistical significance P <0.001.

**Figure 3-Supplement 4**


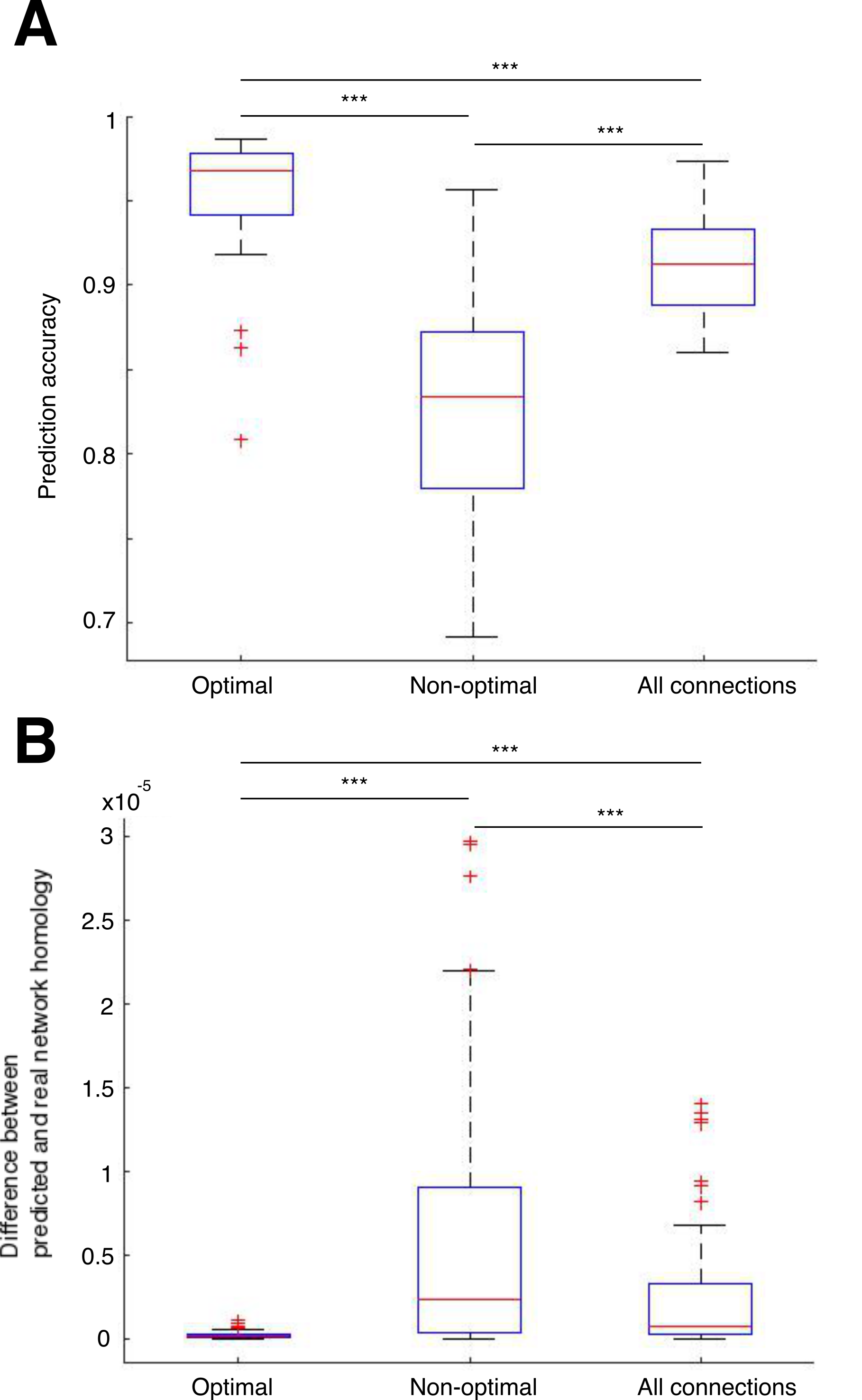


**Predicting functional connectivity from structural connectivity** **constructed using streamlines instead generalized fractional anisotropy for the 234-ROI parcellation** **(A)** We used optimal, non-optimal and all structural connections to predict functional connectivity. Prediction accuracy in terms of cross-matrix correlations between real and predicted matrices was higher using optimal connections (F(2,98)=118.63, P<0.001). **(B)** Prediction results are also presented using the homology method. Lower scores showed that the difference between the real and predicted functional networks was smaller using optimal connections. Predictions shown here are for polynomial order k=5. For each box, thick lines show the median value for n=50 individuals while whiskers reflect the 1.5 interquartile range of the data. Triple asterisks (***) imply *post hoc* statistical significance P <0.001.

**Figure 3-Supplement 5**

**
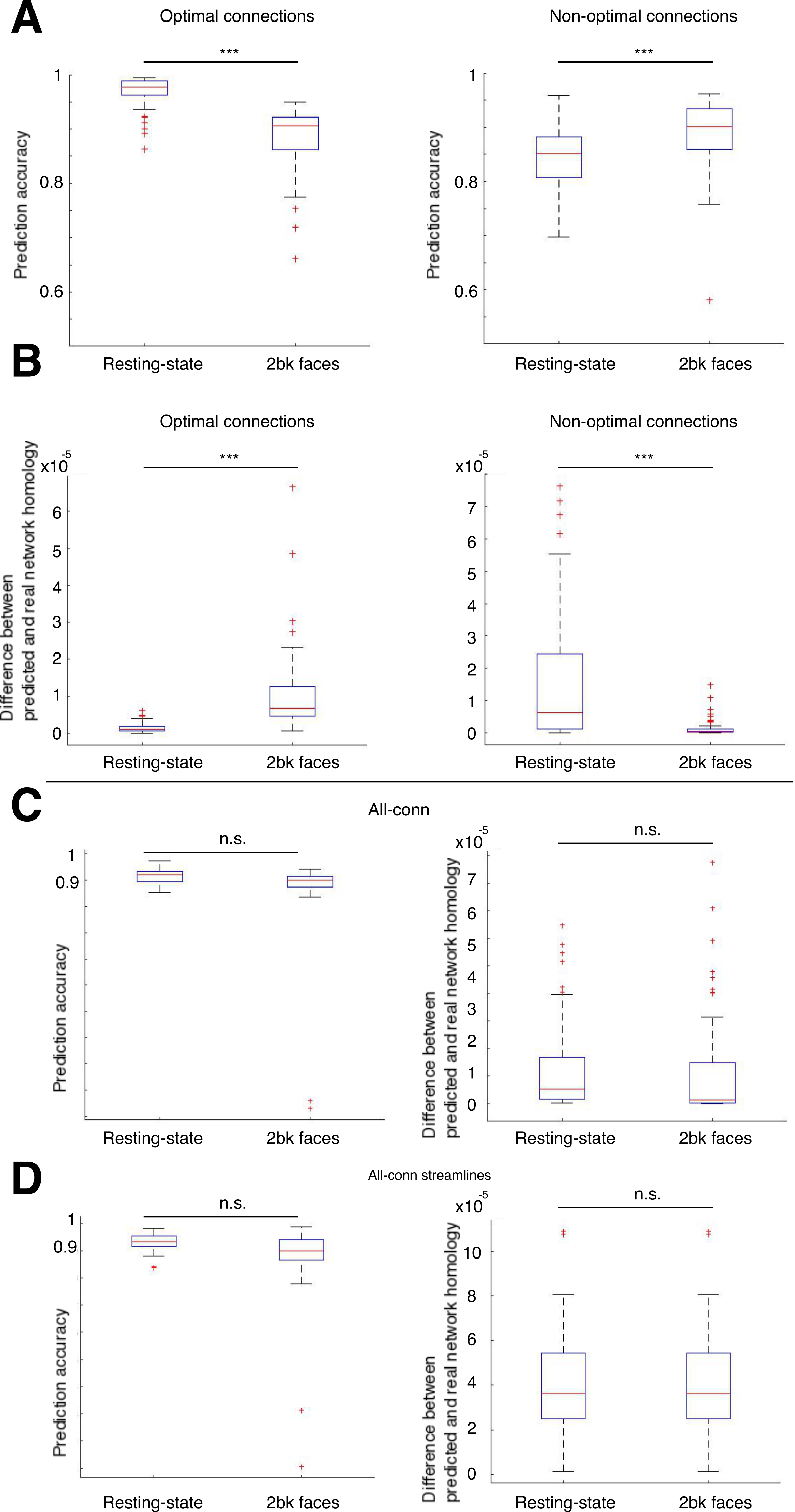
**

**Predicting task-based functional connectivity using the NNG model.** Here we used the functional connectivity of the 50 individuals during a 2bk faces working memory task and we assessed the predictive ability of the optimal and non-optimal connections. Panel **(A)** shows these in terms of cross-matrix correllation accuracy and how they compare with the predictive ability during resting state. Panel **(B)** shows the prediction accuracy in terms of the homology-based measure. In both cases we observed that the predictive ability of optimal connections was decreased during resting-state compared to the 2bk task, while the predictive ability of non-optimal connections increased. In panel (**C**) we also show predictions when using all structural conenctions. In both scores we observed no statistical difference when predicting resting-state and 2bk task functional connectivity. Similar results for all structural connections were observed when using streamlines instead of GFA (panel (**D**)). Predictions shown here are for polynomial order k=5. For each box, thick lines show the median value for n=50 individuals while whiskers reflect the 1.5 interquartile range of the data. Triple asterisks (***) imply statistical significance P <0.001.

**Figure 4-Supplement 1**

**
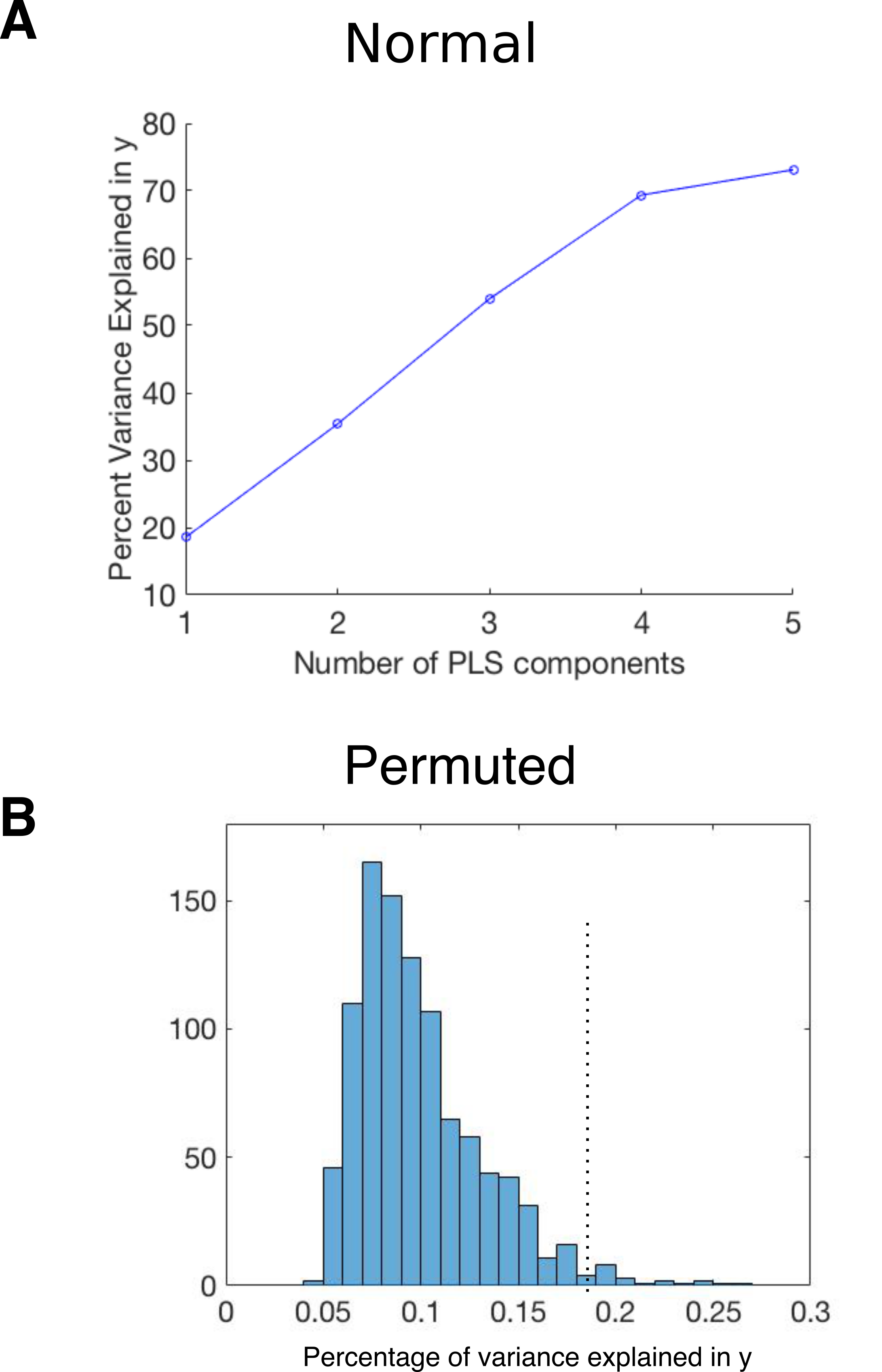
**

**Cummulative explanation in variance by the top 5 PLS components used for regressing genetic data to optimal degree.** **(A)** We used a PLS regression for identifying components in the genetic expression that drive the variance in the optimal minus non-optimal degree difference (RO). Here only the top 5 components are displayed; these cumulatively explained approximately 70% of the variance in the predictor variable (RO score). Out of these the top PLS component explained approximately 20% of the variance. Panel **(B)** shows the percentage of variance explained in y by the top PLS component when the assignment between the gene and the RO data was shuffled 1,000 times. The dotted line indicates the original variance explained in the RO with the non-permuted data.

3. Supplementary Files

**Supplementary File 1**


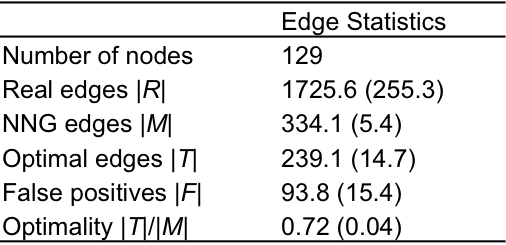


**Optimal edge statistics for the structural networks at the 129-ROI resolution.** Optimality was calculated by counting the number of optimal edges i.e. connections that existed in both the NNG and real networks $\left| T \right|$ divided by the number of total edges the NNG model ($|M|$) produced. False positives are also shown. Results are presented in the form of mean (stdev) over n=50 individuals.

**Supplementary File 2**

**Gene list and gene function ontology terms for the ranked scores of top PLS component**. a) This part of the table shows the significant gene ontology terms and genes as derived by applying a gene ontology analysis to the ranked list of genes coming from the top PLS component. $N$ is the total number of genes, $B$ is the total number of genes associated with specific gene ontology term, $n$ is the number of genes in the top of the selected input list, $b$is the number of genes in the intersection and enrichment is defined as Enrichment = $(b/n) / (B/N)$. b) The second part of the table also shows a condensed version of the significant terms found in the ranked gene list as it is derived from the gene ontology analysis.

**Supplementary File 3**

**List of brain regions and the networks they were assigned to and study demographics.** a) Table shows assignment of brain regions to canonical networks based on Yeo et al. (Yeo et al., 2011). Certain regions were manually labelled as ‘Subcortical’ or ‘Medial Temporal Lobe-MTL’ and were not used in the cortical network analysis presented in **Fig. 3** of the main text. We also present the overlap of each region with its assigned canonical network mask.

b) Table shows the subset of subjects from the Human Connectome Project that was chosen for this study. All subjects were between 21-35 years old (29 females; 21 males).

4. Supplementary References

1. De Santis, S., Drakesmith, M., Bells, S., Assaf, Y., & Jones, D. (2014) Why diffusion tensor MRI does well only some of the time: Variance and covariance of white matter tissue microstructure attributes in the living human brain. *Neuroimage* 89:35-44. doi: 10.1016/j.neuroimage.2013.12.003
2. Smith, S.M., Miller, K.L., Salimi-Khorshidi, G., Beckmann, C.F., Nichols, T.E., Ramsey, J.D., & Woolrich, M.W. (2011) Network modeling methods for fMRI. *Neuroimage* 54:875-891. doi: 10.1016/j.neuroimage.2010.08.063
3. Tuch, D.S. (2004) Q-ball imaging. *Magnetic Resonance in Medicine* 52:1358-1372. doi: 10.1002/mrm.20279
4. Yeh, F.C., Van Jay, W., & Wen-Yih, I.T. (2010) Generalized q-sampling imaging. Medical Imaging, *IEEE Transactions* 29:1626-1635. doi: 10.1109/TMI.2010.2045126
5. Kuo, L.W., Chen, J.H., Wedeen, V.J., & Tseng, W.Y. (2008) Optimization of diffusion spectrum imaging and q-ball imaging on clinical MRI system. *Neuroimage* 41:7-18. doi: 10.1016/j.neuroimage.2008.02.016
6. Chen, Y.J., Lo, Y.C., Hsu, Y.C., Fan, C.C., Hwang, T.J., Liu, C.M., Chien, Y.L., Hsieh, M.H., Liu, C.C., Hwu, H.G., & Tseng, W.Y. (2015) Automatic whole brain tract‐based analysis using predefined tracts in a diffusion spectrum imaging template and an accurate registration strategy. *Hum Brain Mapp* 36:3441-3458. doi: 10.1002/hbm.22854
7. Gong, G., He, Y., Concha, L., Lebel, C., Gross, D.W., Evans, A.C., & Beaulieu, C. (2009) Mapping anatomical connectivity patterns of human cerebral cortex using in vivo diffusion tensor imaging tractography. *Cerebral Cortex* 19:524-536. doi: 10.1093/cercor/bhn102
8. Cohen-Adad, J., Descoteaux, M., Rossignol, S., Hoge, R.D., & Deriche, R. (2008) Detection of multiple pathways in the spinal cord using q-ball imaging. *Neuroimage* 42:739-749. doi: 10.1016/j.neuroimage.2008.04.243
9. Smith, S.M., Beckmann, C.F., Andersson, J., Auerbach, E.J., Bijsterbosch, J., Douaud, G., Duff, E., Feinberg, D.A., Griffanti, L., Harms, M.P., Kelly, M., Laumann, T., Miller, K.L., Moeller, S., Petersen, S., Power, J., Salimi-Khorshidi, G., Snyder, A.Z., Vu, A.T., Woolrich, M.W., Xu, J., Yacoub, E., Uğurbil, K., Van Essen, D.C., Glasser, M.F., & WU-Minn HCP Consortium (2013) Resting-state fMRI in the Human Connectome Project. *Neuroimage* 80:144-168. doi: 10.1016/j.neuroimage.2013.05.039
10. Barch DM, Burgess GC, Harms MP, Petersen SE, Schlaggar BL, Corbetta M, Glasser MF, Curtiss S, Dixit S, Feldt C, Nolan D, Bryant E, Hartley T, Footer O, Bjork JM, Poldrack R, Smith S, Johansen-Berg H, Snyder AZ, Van Essen DC, & WU-Minn HCP Consortium (2013) Function in the human connectome: task-fMRI and individual differences in behavior. *Neuroimage* 80:169-189. doi: 10.1016/j.neuroimage.2013.05.033.
11. Fair, D.A., Schlaggar, B.L., Cohen, A.L., Miezin, F.M., Dosenbach, N.U., Wenger, K.K., Fox, M.D., Snyder, A.Z., Raichle, M.E., & Petersen, S.E. (2007) A method for using blocked and event-related fMRI data to study "resting state" functional connectivity. *Neuroimage* 35:396-405
12. Nisan, N. (2007) *Algorithmic Game Theory*(Cambridge University Press, New York). doi: 10.1017/CBO9780511800481.001
13. Schrijver, A. (1987) *Theory of linear and integer programming.* (John Wiley & Sons, Inc., New York)
14. Margulies, D.S., Falkiewicz, M., & Huntenburg, J.M. (2016) A cortical surface-based geodesic distance package for Python. *GigaScience* 5:s13742-016-0147-0-q. doi.org/10.1186/s13742-016-0147-0-q
15. Chung, F.R.K. (1997) Spectral graph theory. American Mathematical Society. doi: 10.1090/cbms/092
16. Boyd, S., & Vandenberghe, L. (2007) *Convex optimization* (Cambridge University Press, New York). doi: 10.1017/CBO9780511804441.001
17. Schönemann, P.H. (1968) On two-sided orthogonal Procrustes problems. *Psychometrika* 33. doi: 10.1007/BF02289673
18. Arnatkeviciūtė, A., Fulcher, B.D., & Fornito, A. (2019) A practical guide to linking brain-wide gene expression and neuroimaging data. *Neuroimage* 1;189:353-367. doi.org/10.1016/j.neuroimage.2019.01.011
19. Váša, F., Seidlitz, J., Romero-Garcia, R., Whitaker, K.J., Rosenthal, G., Vértes, P.E., Shinn, M., Alexander-Bloch, A., Fonagy, P., Dolan, R.J., Jones, P., Goodyer, I.M., the NSPN consortium, Sporns, O., & Bullmore E.T. (2018) Adolescent Tuning of Association Cortex in Human Structural Brain Networks. *Cereb Cortex* 28: 281-294. doi: 10.1093/cercor/bhx249
20. Eden, E., Navon, R., Steinfeld, I., Lipson, D., & Yakhini, Z. (2009) GOrilla: A tool for discovery and visualization of enriched GO terms in ranked gene lists. *BMC Bioinformatics* 10:48. doi: 10.1186/1471-2105-10-48
21. Eden, E., Lipson, D., Yogev, S., & Yakhini, Z. (2007) Discovering motifs in ranked lists of DNA sequences. *PLOS Computational Biology* 3:e39. doi: 10.1371/journal.pcbi.0030039
22. Supek, F., Bošnjak, M., Škunca N., & Šmuc, T. (2011) REVIGO summarizes and visualizes long lists of Gene Ontology terms. *PLOS ONE* 6:e21800. doi: 10.1371/journal.pone.0021800

5. Data and code availability

Data is open-access and can be downloaded from the Human Connectome Project website. Code was built using custom-made based scripts on top of publicly available code (cited when available). Code will be publicly available on a github repository after publication.

6. Author contributions

IP and MMC collated and processed the data.

IP and MMC conducted statistical and network analyses.

DKM and EAS provided advice on creation and interpretation of results.

IP, MMC, DKM, and EAS wrote the manuscript.

All authors critically appraised the manuscript.
