# Supplementary figures and images for: Structural optimality and neurogenetic expression mediate functional dynamics in the human brain
Source: Hum Brain Mapp. 2020 Feb 6;41(8):2229–43. doi: 10.1002/hbm.24942 (PMC7267953; doi:10.1002/hbm.24942)

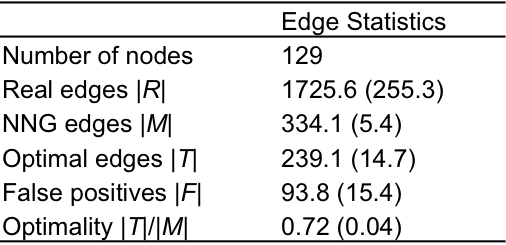

Supplement: Supplementary file 1 — Appendix S1: Supplementary Information [file HBM-41-2229-s001.zip › HBM_24942_Supplementary_File1.docx]
